# Synthesis of N-CD_3_ aryl amines *via* iron-catalysed site-selective aromatic C–H amination

**DOI:** 10.1039/d5sc03780h

**Published:** 2025-09-22

**Authors:** Meng-Meng Ren, Yin Yang, Fei Wang

**Affiliations:** a State Key Laboratory of Elemento-Organic Chemistry, Frontiers Science Center for New Organic Matter, College of Chemistry, Nankai University Weijin Rd. 94 Tianjin 300071 China fwang235@nankai.edu.cn

## Abstract

The strategic incorporation of deuterium atoms into pharmaceutical compounds can profoundly influence their pharmacokinetic profiles and metabolic stability. This is particularly relevant for the ubiquitous N-methyl motif in bioactive molecules, where metabolic oxidation of the methyl group often represents a major pathway. Despite this potential, synthetic methods for the direct introduction of the N-CD_3_ group through C–H functionalization remain elusive. We report herein an iron-catalysed protocol for the synthesis of N-CD_3_ anilines through site-selective aromatic C–H amination. An iron-aminyl radical is proposed as the key intermediate that facilitates site-selective homolytic aromatic substitution (HAS) through chelating with basic functional groups, including amides, urea and carbamate. The resulting *ortho*-amino products serve as versatile synthetic intermediates for valuable heterocycles. Importantly, the Weinreb amide proves effective as a directing group, offering the advantage of transforming into diverse carbonyl molecules.

## Abstract 2

An iron-catalysed protocol for site-selective aromatic C–H N-CD_3_ amination is reported. The iron-aminyl radical is proposed as the key intermediate that facilitates homolytic aromatic substitution through chelation with basic functional groups.

## Graphical abstract

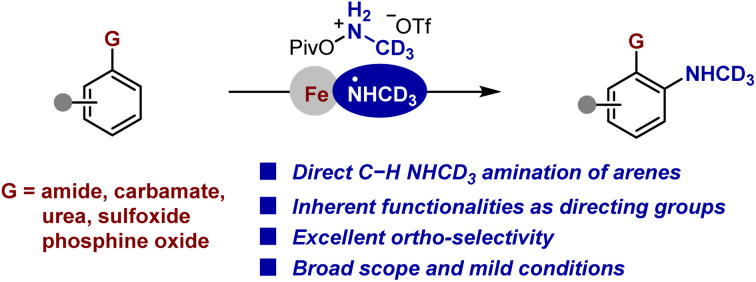

## Introduction

**Fig. 1:**
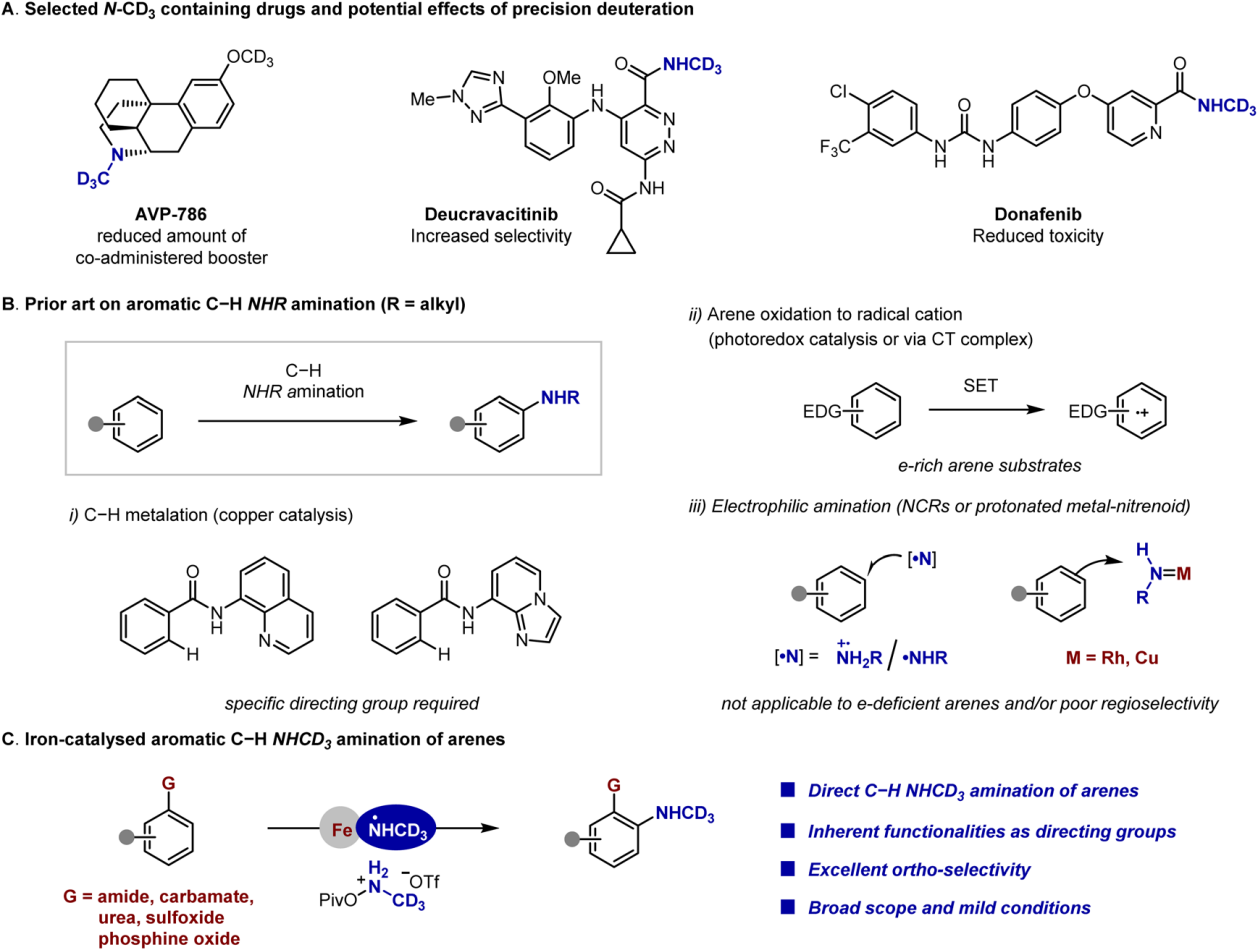
Context of this work. SET = single electron transfer, CT = charge transfer, EDG = electron-donating group.

While the substitution of hydrogen with deuterium represents the smallest possible structural modification in organic molecules, its impact on drug properties can be profound.^1^ As demonstrated by deutetrabenazine, the first FDA-approved deuterated drug, its superior pharmacokinetic profile over its non-deuterated counterpart enables reduced dose and dosing frequency.^2^ Consequently, synthetic methods for deuterium incorporation—whether during target molecule construction or *via* late-stage H/D exchange—have garnered increasing attention.^3^ This is especially pertinent for N-methyl (N-CH_3_) groups, which are ubiquitous in bioactive compounds yet often susceptible to oxidative metabolism.^1,4,5^ Replacing N-CH_3_ with N-CD_3_ offers a compelling strategy to attenuate such metabolic liabilities (Fig. 1A).^6^ Thus, the direct incorporation of the N-CD_3_ motif into organic molecules is highly demanded.

The prevalence of aryl amines in pharmaceuticals underscores the importance of developing efficient methods for their synthesis.^7^ Direct amination of aromatic C–H bonds represents an attractive strategy, combining the ready availability of arene substrates with the step-/atom-economy inherent to C–H functionalization processes.^8^ While significant progress has been achieved in this field, limited success has been achieved for the direct introduction of an NHR group (R = alkyl) to access secondary aryl amines (Fig. 1B).^9,10^ The pioneering works from Minisci and co-workers established the feasibility of NHR amination of arenes *via* the N-alkyl aminium radical cation, albeit under harsh conditions with low yields and limited substrate scope.^11^ Recently, significant advances have been made by Falck, Kürti, Ess, Nicewicz, Leonori, Morandi, Hashmi and Phipps through developing new catalytic systems and novel aminating reagents (Fig. 1B).^12–14^ For instance, Falck and co-workers developed dirhodium- and copper-catalysed systems using NH(alkyl)-*O*-(sulfonyl)hydroxylamines.^12*a*,*c*^ Morandi reported an iron-catalysed variant employing NH(methyl)-*O*-(sulfonyl)hydroxylamines·HOTf.^12*e*^ Hashmi presented a metal-free system using NH(alkyl)-*O*-(sulfonyl)hydroxylamines, in which a charge transfer (CT) complex between the arene substrate and hydroxylamine reagent was suggested.^12*f*^ Photoredox approaches have also emerged. Nicewicz and co-workers reported a photocatalytic amination of arenes with primary amines, wherein oxidation of an arene to an arene radical cation was proposed.^12*b*^ Alternatively, the group of Leonori employed alkyl amines and N-chlorosuccinimide (NCS) to generate N-chloroalkylamines *in situ*, which served as effective aminating reagents through photochemical generation of nitrogen centered radicals (NCRs).^12*c*^ However, these reactions work ineffectively for electron-deficient arenes and/or suffer from poor regioselectivity. Phipps and co-workers deployed the non-covalent interaction between the N-alkyl aminium radical cation and anionic arene substrates to enable highly *ortho*-selective amination of sulfamate derived from aniline.^15*a*^ More recently, they achieved an *ortho*-selective amination of arene carboxylic acids *via* an intramolecular rearrangement of acyl O-hydroxylamines.^15*c*^ Despite these important contributions, there is still a necessity for the development of complementary methods, which is applicable to readily available arene substrates and exerts excellent regioselectivity. Moreover, this field still lacks general methods for site-selective NH-CD_3_ amination of arene C–H bonds—a significant gap given the growing importance of deuterated pharmaceuticals.

With our continuous interest in radical-mediated selective amination reactions,^16^ we present here an iron-catalysed method for direct access to N-CD_3_ anilines through site-selective aromatic C–H functionalization (Fig. 1C). An iron-aminyl radical is invoked as the key intermediate that facilitates the site-selective homolytic aromatic substitution (HAS) *via* chelating with basic functional groups. Notably, the Weinreb amide proves effective as a directing group, allowing access to other carbonyl functionalities through established protocols. The *ortho*-amino benzamide products are valuable synthetic building blocks towards pharmacologically relevant heterocycles.

## Results and discussion

### Reaction development

**Fig. 2:**
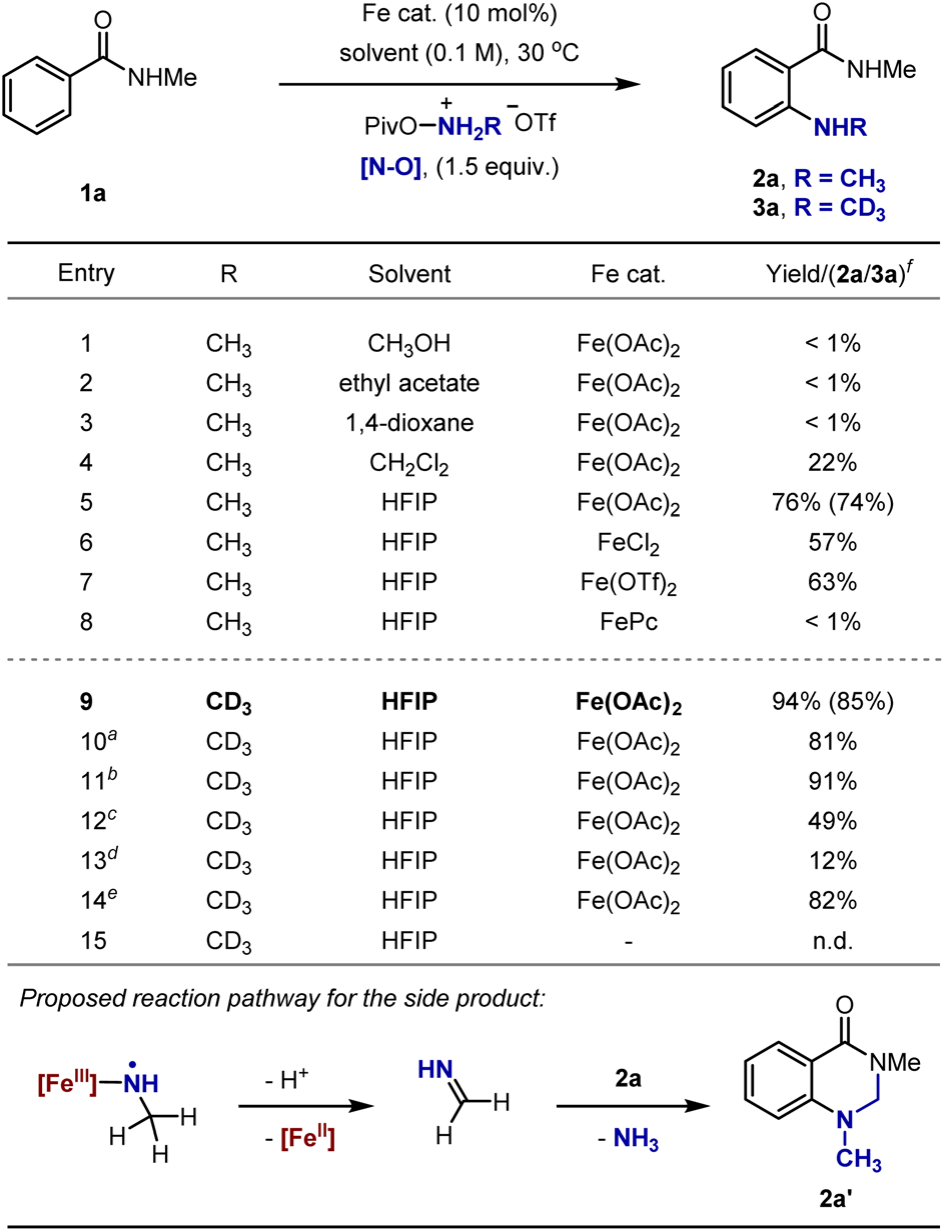
Reaction development. ^a^The reaction was performed at 0 °C. ^b^With H_2_O (1.0 equiv.). ^c^With H_2_O (5.0 equiv.). ^d^With H_2_O (10.0 equiv.). ^e^Under air. ^f^Only *ortho*-aminated products were formed. The yields were determined by 1H-NMR analysis with CH_2_Br_2_ as an internal standard. Isolated yields are shown in parentheses. n.d. = not detected.

Our experiments were initiated with the *ortho*-selective N-CH_3_ amination of benzamide 1a using NH(CH_3_)-*O*-(pivaloyl)hydroxylamines·HOTf as the electrophilic aminating reagent (see the SI for full reaction optimizations).^17^ No appreciable yield (<1%) was obtained when the reaction was conducted with Fe(OAc)_2_ as a catalyst in methanol, ethyl acetate or 1,4-dioxane, while the product was afforded in 22% yield using CH_2_Cl_2_ as a solvent (Fig. 2, entries 1–4). HFIP proved to be optimal, giving the desired product in 76% yield. The use of FeCl_2_ or Fe(OTf)_2_ in replacement of Fe(OAc)_2_ led to slightly decreased yield, while the reaction is completely shut down using iron(ii) phthalocyanine (Fig. 2, entries 5–8). Notably, dihydroquinazolinone side product 2a′ was observed in addition to 2a (see the SI for details). We attribute these results to the involvement of an imine or iminium, generated *via* deprotonation of the α-C–H bond of an iron-aminyl radical or free NCR^18^ and the subsequent single electron oxidation, followed by its condensation with 2a (Fig. 2, bottom). Strikingly, higher yield was obtained when using NH(CD_3_)-*O*-(pivaloyl)hydroxylamines·HOTf as the aminating reagent under otherwise identical conditions, with no *d*_5_-2a′ formed (Fig. 2, entry 9). These results might stem from the attenuated deprotonation of the deuterated NCRs thanks to the deuterium kinetic isotope effect.^19^ Running the reaction at lower temperature leads to decreased yield with 10% of 1a remaining (Fig. 2, entry 10). While adding one equivalent of water into the reaction mixture did not affect the reaction, high water loading significantly decreased the yield (Fig. 2, entries 11–13). We proposed that water might compete with 1a in coordinating with the iron-aminyl radical, shunting the desired radical addition to arenes. Alternatively, the iron-aminyl radical complex might be destroyed by water through coordination with iron. Slightly decreased yield was observed when the reaction was performed under air, implying its insensitivity to oxygen (Fig. 2, entry 14). Finally, no reaction occurred in the absence of the iron catalyst (Fig. 2, entry 15).

### Proposed reaction pathway

**Fig. 3:**
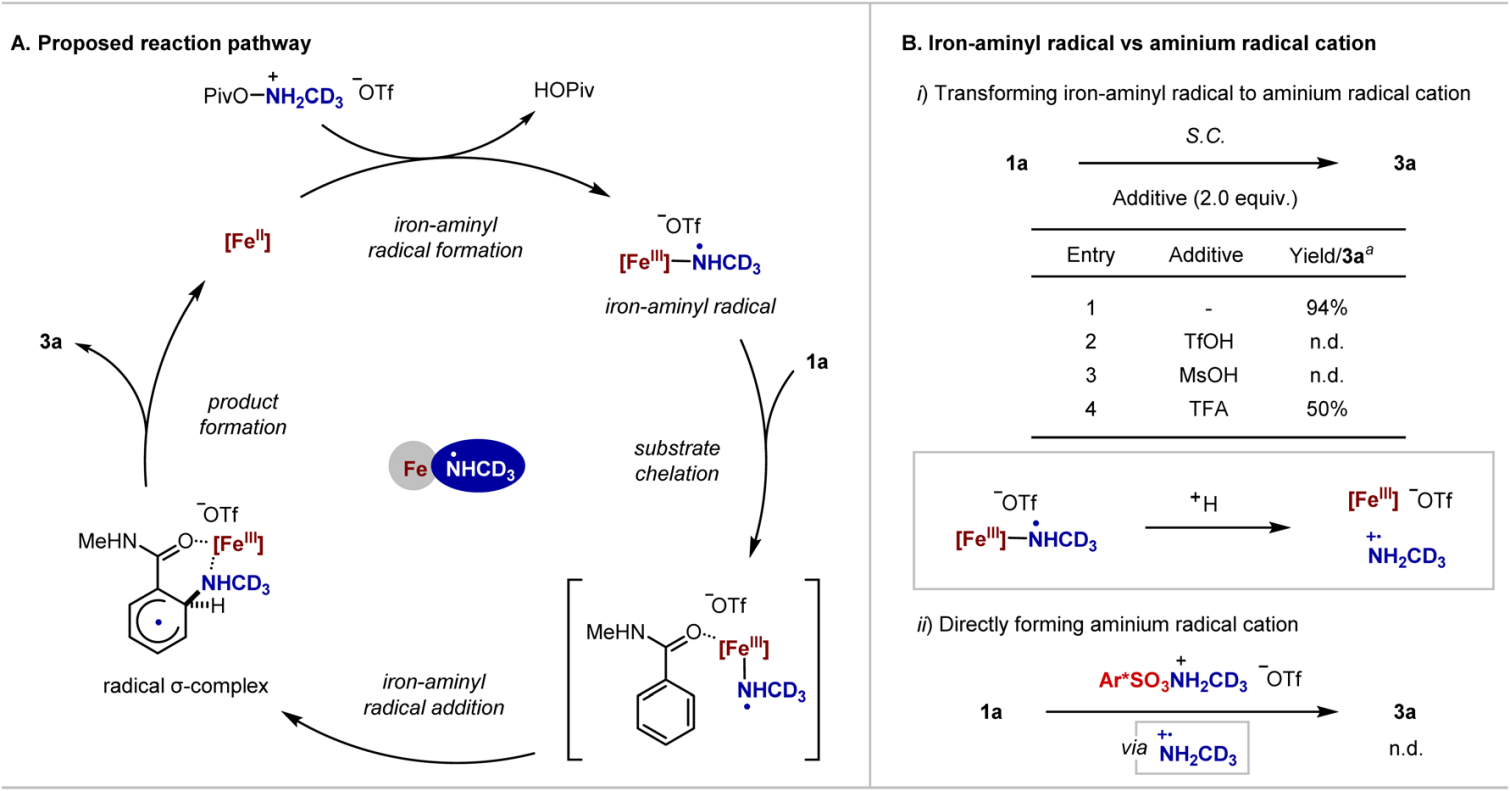
Proposed reaction pathway and control experiments. (A) The proposed reaction pathway involving an iron-aminyl radical. (B) Control experiments that preclude the involvement of an aminium radical cation. These reactions were analysed by ^1^H-NMR with CH_2_Br_2_ as an internal standard. ^a^Only *ortho*-aminated products were formed. Ar* = 4-NO_2_C_6_H_4_.

Building on our previous studies on iron-catalysed primary amination of arene C–H bonds^16*b*–*e*^ and related precedents in the literature,^20^ a proposed reaction pathway is illustrated in Fig. 3A. The reaction begins with single electron transfer between Fe(ii) and NH(CD_3_)-*O*-(pivaloyl)hydroxylamines·HOTf to afford the key iron-aminyl radical intermediate, along with formation of pivalic acid. Then, substrate chelation takes place to facilitate radical addition and dictate the regioselectivity, generating a σ-complex. Finally, the product was formed as an anilinium triflate, *via* electron transfer and proton transfer or proton coupled electron transfer, while the Fe(ii) catalyst was regenerated.

The involvement of NCR was evidenced by electron paramagnetic resonance (EPR) analysis of the reaction mixture (see the SI for details). The excellent *ortho*-selectivity suggested against the intermediacy of the free aminium radical cation because the amide anion species is unlikely formed under the present acidic conditions to facilitate the site-selective radical addition *via* electrostatic interaction.^15^ In addition, a tertiary amide is a suitable substrate, affording the associated product with excellent site-selectivity (*vide infra*). Moreover, no 3a was formed when a strong acid, such as TfOH or MsOH, was added into the reaction mixture, assuming the iron-aminyl radical is converted to the aminium radical cation through protonation (Fig. 3B). Meanwhile, using NH(CD_3_)-*O*-(sulfonyl)hydroxylamines·HOTf as an aminating reagent, previously proposed to generate an aminium radical cation upon reaction with Fe(ii), did not lead to any desired product.^12*e*,15*b*^ The lack of reactivity with the aminium radical cation might arise from the electron-deficient character of benzamide. These results highlight the unique reactivity and selectivity of the iron-aminyl radical intermediate, assisted by substrate chelation.

### Substrate scope

**Fig. 4:**
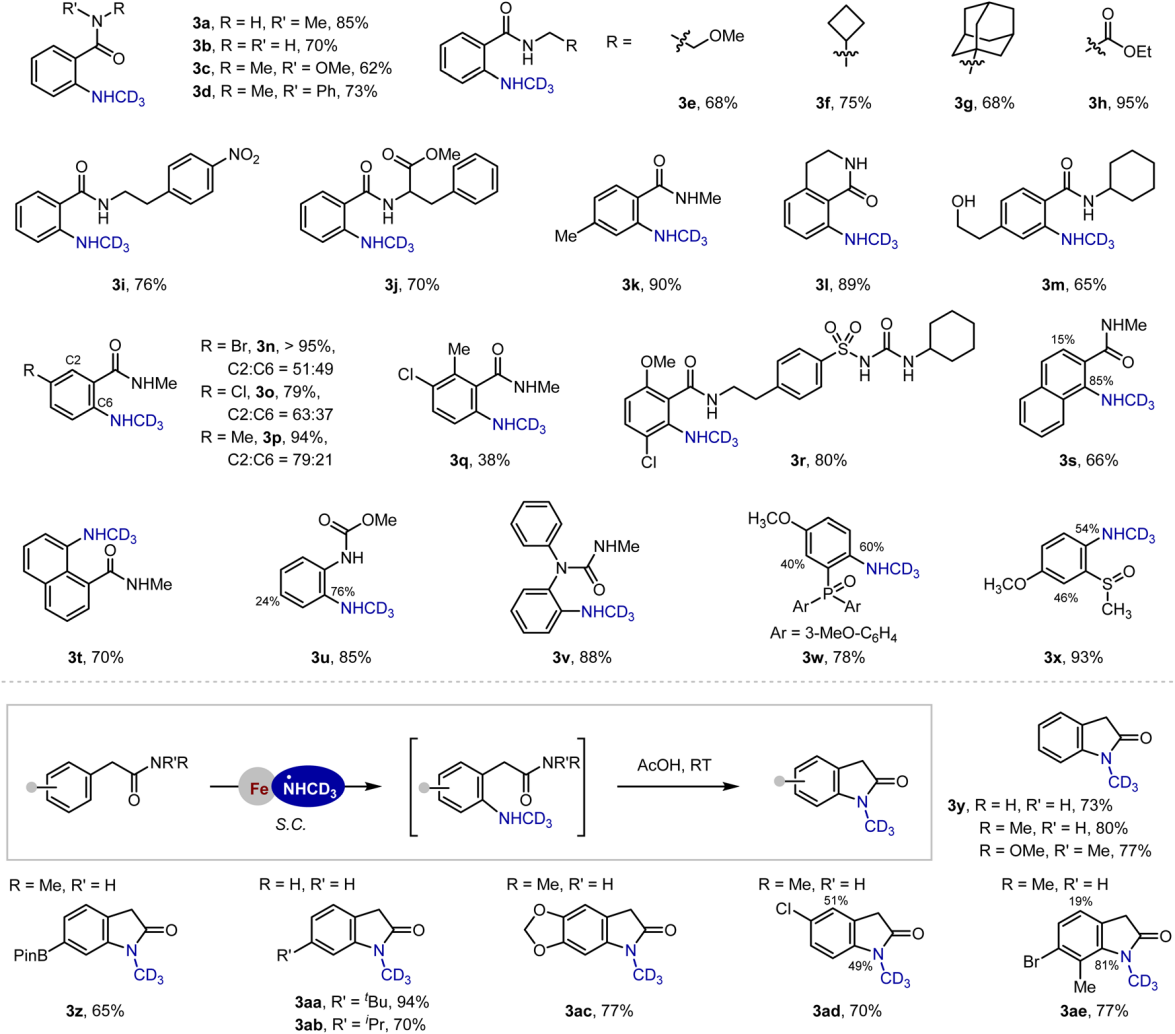
Substrate scope. Isolated yields, see the SI for details. Only *ortho*-aminated products were formed except for 3t and 3u.

**Fig. 5:**
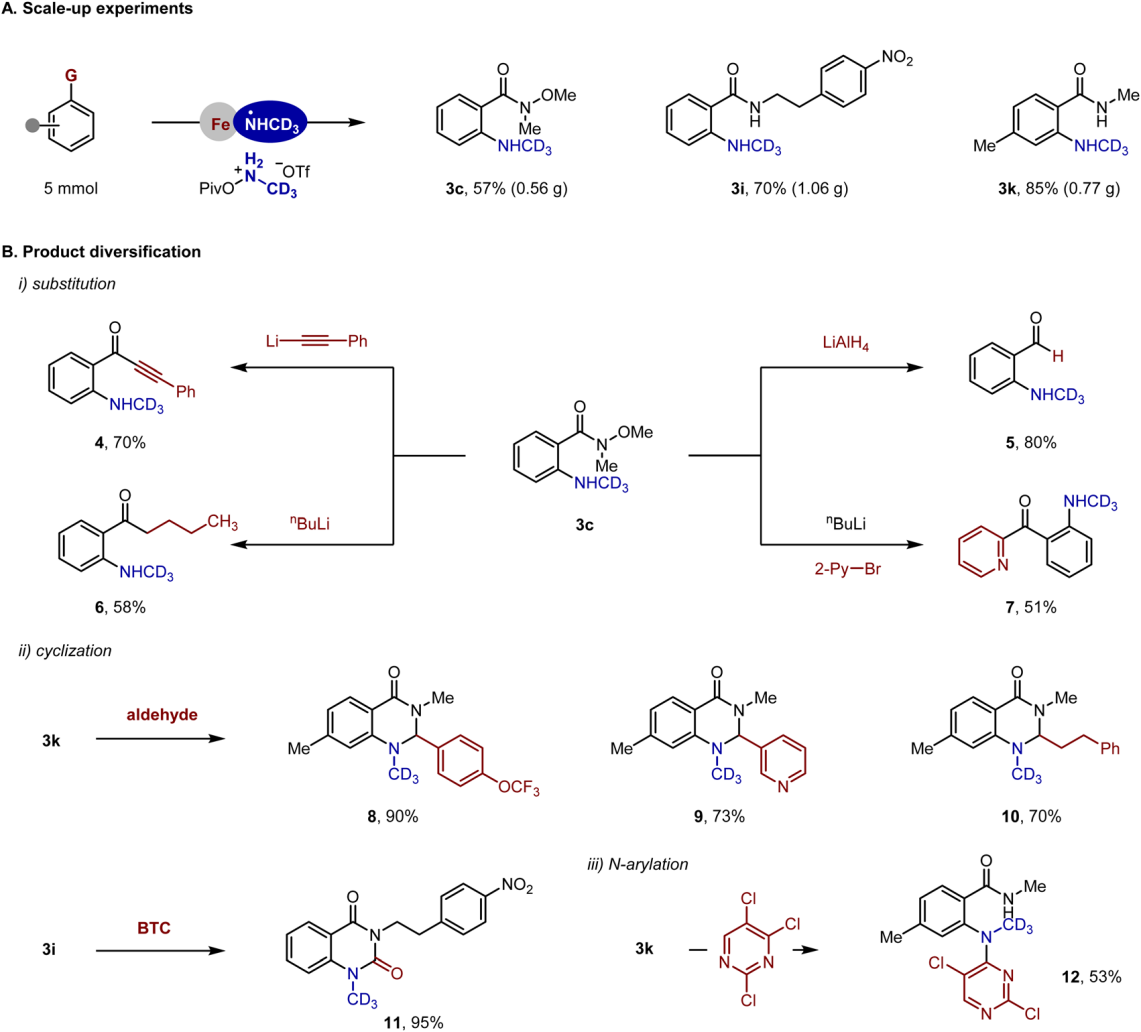
Gram-scale reaction and product diversification. Isolated yields, see the SI for details.

Efforts were then made to investigate the reaction scope (Fig. 4). The protocol demonstrated excellent compatibility with primary, secondary, and tertiary benzamides, consistently delivering *ortho*-NHCD_3_ products in good-to-excellent yields (3a–3d, 62–85%). The Weinreb amide proved particularly valuable, serving both as an effective directing group and as a versatile synthetic handle for subsequent transformations (*vide infra*). In addition to the exclusive *ortho*-selectivity, selective amination of the electron-deficient aromatic motif for 3d is noteworthy, underscoring the unique regiochemical control imparted by the iron-aminyl radical. Secondary benzamides derived from diverse primary alkylamines are amenable to this reaction (3e–3j), including biologically relevant substrates like phenylalanine derivatives (3j). Substituted benzamides bearing halide, alkyl or hydroxyl groups all participated effectively in the present amination reaction, affording the desired products in good-to-excellent yields (3k–3q). Notably, the protocol enabled late-stage C–H amination of glibenclamide, an oral hypoglycemic agent for the treatment of diabetes, with the site-selectivity exclusively dictated by amide chelation, overriding the inherent electronic effect of the methoxyl substituent (3r). Naphthalene derivatives exhibited interesting regiochemical outcomes: while 2-naphthalenecarboxamide reacted conventionally (3s, 66%), the 1-isomer showed preferential amination at the C8 position (3t, 70%, C8 : C2 > 20 : 1). The reaction with phenylcarbamate mainly afforded the *ortho*-aminated product, while the *para*-product was also observed due to the electron-rich character of the aromatic ring and the non-directed HAS pathway (3u). In contrast, the urea substrate gave an exclusive *ortho*-selectivity (3v). However, aryl phosphine oxide and sulfoxide could only work for substrates bearing an electron-donating group (3w and 3x), *e.g.* methoxyl group, possibly due to the decreased electrophilicity and accordingly the reactivity of the proposed Fe-·NHCD_3_ than that of Fe-·NH_2_ (see the SI for unsuccessful substrates). In addition, the methodology extended effectively to 2-phenylacetamides, providing direct access to 2-(2-aminophenyl)acetamides that could be cyclized to 2-indolinones under mild acidic conditions (3y–3ae, 65–94%). Both primary and secondary amides performed well, and functionality including boronic esters (3z) and bromides (3ae) remained intact, offering valuable handles for further derivatization. Of note, the reaction is easily to scale-up without diminishing the reaction efficiency and regioselectivity (Fig. 5A, 3c, 3i and 3k).

### Product diversification

The versatility of Weinreb amide allowed us to access a variety of carbonyl molecules through convenient nucleophilic substitution reactions (Fig. 5B, see the SI for details). For example, 3c was converted to an ynone product (4) in 70% yield using (phenylethynyl)lithium as a nucleophile. 2-Aminobenzaldehyde (5) was obtained in 80% yield through treatment of 3c with LiAlH_4_. Other reagents such as BuLi and 2-PyLi are suitable nucleophiles that afford structurally diverse carbonyl products in moderate yields (6 and 7). In addition, the 2-aminobenzamide products were transformed to a number of pharmaceutically relevant heterocycles. For instance, 3k was condensed with aldehydes, generating dihydroquinazolinones (8–10) in good yields; cyclization of 3i with bis(trichloromethyl) carbonate (BTC) took place effectively to give 11 in 95% yield. Finally, 3k was subjected to an S_N_Ar reaction, generating 12 in a yield of 53%.

## Conclusions

In conclusion, we have developed an electrophilic N-CD_3_ aminating reagent, NH(CD_3_)-*O*-(pivaloyl)hydroxylamines·HOTf, and demonstrated its utility in iron-catalysed, *ortho*-selective C–H amination of arenes. This transformation exhibits broad substrate scope, accommodating primary, secondary and tertiary benzamides, phenyl carbamate, phenyl urea and 2-phenylacetamides, while maintaining good yield and regioselectivity for late-stage C–H amination of complex molecules. The unique regiochemical control imparted by an iron-aminyl radical through substrate chelation supersedes the inherent substituent directing effects. Moreover, the amenability of using the Weinreb amide as a directing group enables subsequent product diversification; *ortho*-amino benzamides are valuable synthetic intermediates to build heterocycles. Current investigations are focused on exploring applications of this electrophilic N-CD_3_ reagent in other selective transformations.

## Author contributions

M.-M. R. conducted all the experiments and characterized all the new compounds. The EPR experiments were performed by Y. Y. with assistance from M.-M. R. M.-M. R. and F. W. designed the experiments and wrote the manuscript.

## Conflicts of interest

There are no conflicts to declare.

## Supplementary Material

SC-OLF-D5SC03780H-s001

## Data Availability

Supplementary information: experimental procedures and analytical data (NMR, HRMS and EPR). See DOI: https://doi.org/10.1039/d5sc03780h.
