# Supplementary material for: Synthesis of N-CD3 aryl amines via iron-catalysed site-selective aromatic C–H amination
Source: Chem Sci. 2025 Sep 22;16(41):19350–6. doi: 10.1039/d5sc03780h (PMC12452166; doi:10.1039/d5sc03780h)
Supplement: SC-016-D5SC03780H-s001 [file SC-016-D5SC03780H-s001.pdf]

Supplementary Information (SI) for Chemical Science.
This journal is © The Royal Society of Chemistry 2025

Supplementary Information for

Synthesis of N-CD_3 aryl amines via iron-catalysed site-selective aromatic C−H amination

Meng-Meng Ren, Yin Yang, and Fei Wang*

*Correspondence to: fwang235@nankai.edu.cn.

Contents:

1. General Information 2
2. General Procedures 2
3. Synthesis of the Aminating Reagents 3
4. Reaction Conditions Optimization 8
5. Spin Trap Experiments 13
6. Reactions with Other N-Alkyl Aminating Reagents. 14
7. Comparison between Aminium Radical Cation and Iron-Aminyl Radical 16
8. Experimental Procedures and Characterization Data 17
9. Product Derivatizations 49
10. Unsuccessful Substrates 55
11. NMR Spectra of Substrates and Products 56
12. References 145

1. General Information

The commercial starting materials were purchased from J&K Chemical, Adamas-Beta, Innochem, Macklin, MERYER, Bide Pharmatech, Energy Chemical and Heowns, and used as received without further purification unless otherwise noted.

The silica gel (particle size 40-63 μm) used for flash column chromatography was supplied by Innochem. Analytical thin layer chromatography (TLC) silica gel plates (250 μm thickness) with F254 indicator were purchased from Tansoole. Visualization was accomplished with UV light (254 nm), the potassium permanganate stain solution or iodine vapor.

The ^{1}H NMR, ^{13}C NMR, and ^{19}F NMR spectra were recorded on Bruker DRX-400 (400 MHz) spectrometer and chemical shifts were reported in ppm. The following abbreviations (and their combinations) are used to label the multiplicities: s (singlet), d (doublet), t (triplet), q (quartet), m (multiplet) and brs (broad singlet). ^{13}C NMR spectra were recorded with complete proton decoupling. Chemical shifts of the NMR spectra were calibrated by the literature values of the solvent residual peaks (Fulmer, G. R. *et al. Organometallics* **2010**, *29*, 2176-2179).

High-resolution mass spectra were obtained using a Thermo Q Exactive Focus LC/MS.

EPR experiment was performed with a Bruker super-high Q resonator (ER 4122 SHQ).

2. General Procedures

General Procedure A: *ortho*-selective C−H N-CH_3 amination

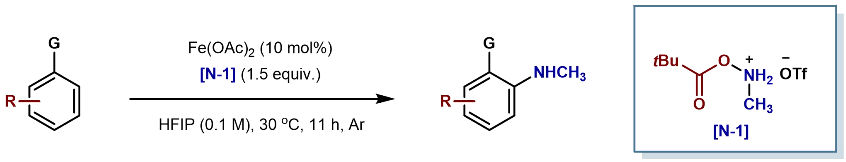

In a glovebox, a 10 mL glass vial was charged with $Fe(OAc)_2$ (0.04 mmol, 10 mol%), aminating reagent **[N-1]** (0.60 mmol, 168.6 mg, 1.5 equiv.) and HFIP (4 mL, 0.1 M), followed by adding arene substrate (0.4 mmol, 1.0 equiv.). The vial was removed from the glovebox upon sealed with a rubber septum or screwed access cap, and the reaction mixture was vigorously stirred at 30 °C for 11 hours. The vial was then cooled to room temperature. The reaction mixture was added with saturated EDTA solution (disodium edetate dihydrate, 2.0 mL) and then solid $NaHCO_3$ (slow addition) until it became basic. After stirring for an additional hour, the reaction

mixture was extracted with DCM (5 mL) for three times. The combined organic phase was dried over Na_2SO_4, filtered, and concentrated under reduced pressure. The crude mixture was then subjected to ^1H NMR analysis using CH_2Br_2 as the internal standard. followed by purification by flash column chromatography with silica gel to afford the desired products.

General Procedure B: *ortho*-selective C−H N-CD_3 amination

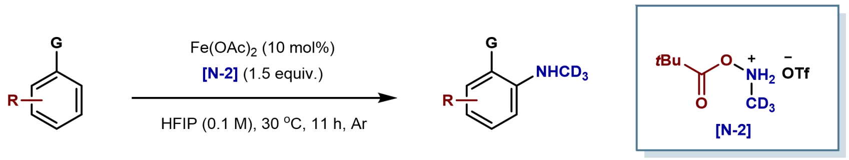

In a glovebox, a 10 mL glass vial was charged with $Fe(OAc)_2$ (0.04 mmol, 10 mol%), aminating reagent [N-2] (0.60 mmol, 170.4 mg, 1.5 equiv.) and HFIP (4 mL, 0.1 M), followed by adding the arene substrate (0.4 mmol, 1.0 equiv.). The vial was removed from the glovebox upon sealed with a rubber septum or screwed access cap, and the reaction mixture was vigorously stirred at 30 °C for 11 hours. The vial was then cooled to room temperature. The reaction mixture was added with saturated EDTA solution (disodium edetate dihydrate, 2 mL) and then solid $NaHCO_3$ (slow addition) until it became basic. After stirring for an additional hour, the reaction mixture was extracted with DCM (5 mL) for three times. The combined organic phase was dried over Na_2SO_4, filtered, and concentrated under reduced pressure. The crude mixture was then subjected to ^1H NMR analysis using CH_2Br_2 as the internal standard. followed by purification by flash column chromatography with silica gel to afford the desired products.

3. Synthesis of the Aminating Reagents

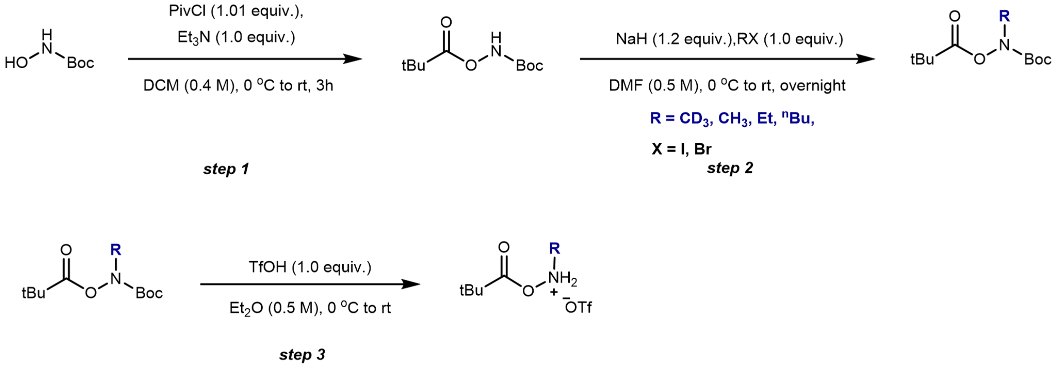

These reactions were performed following the procedure in the literature.[1]

Step 1: N-Boc hydroxylamine (30.0 g, 225.0 mmol, 1.0 equiv.) was dissolved in DCM (0.4 L). The resulting solution was cooled to 0 °C, then triethylamine (31.3 mL, 225.0 mmol, 1.0 equiv.) and pivaloyl chloride (28.0 mL, 228.0 mmol, 1.05 equiv.)

were added sequentially during vigorously stirring. The reaction mixture was allowed to warm to room temperature and was stirred for 2 hours. The precipitate was filtered off and washed with DCM (3 x 30 mL). The filtrate was washed with water (200 mL), sat. aq. $NaHCO_3$ (200 mL) and brine. The organic layer was dried over anhydrous Na_2SO_4 and concentrated under reduced pressure. The title compound was obtained as a white solid in 94.3% yield (46.0 g, 212.0 mmol) and was used without further purification.

Step 2: NaH (60% in mineral oil, 1.2 equiv.) was added portion wise to a stirred solution of *tert*-butyl (pivaloyloxy)carbamate (1.0 equiv.) in anhydrous DMF (0.5 M) at 0 °C. The reaction mixture was stirred at 0 °C for additional 30 min. Then, haloalkanes (1.0 equiv., CH_3I or CD_3I or C_2H_5I or $^nC_4H_9Br$) was added slowly and the reaction mixture was stirred at room temperature for 16 hours. After that, the reaction mixture was quenched by slow addition of water (100 mmol per 50 mL) at 0 °C. The aqueous reaction mixture was extracted with EA (three times). The combined organic layers were washed with brine, dried over anhydrous Na_2SO_4 and concentrated under reduced pressure. The crude product was purified by flash column chromatography.

Step 3: *tert*-Butyl alkyl(pivaloyloxy)carbamate (50.0 mmol, 1.0 equiv.) was suspended in diethyl ether (200 mL). The resulting suspension was cooled to 0 °C and triflic acid (4.4 mL, 50.0 mmol, 1.0 equiv.) was added dropwise under vigorous stirring. After the addition was complete, the reaction mixture was allowed to warm to room temperature and was stirred for 2 hours. The precipitate was filtered off, washed with diethyl ether (3 × 30 mL) and dried under reduced pressure.

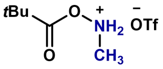

[N-1]

Name: N-methyl-O-pivaloylhydroxylammonium trifluoromethanesulfonate [N-1]

Yield: 87% for step 2, 79% for step 3.

Physical property: white solid

^{1}H NMR (400 MHz, CD_3CN) δ 10.50 (brs, 2H), 3.12 (s, 3H), 1.29 (s, 9H).

^{13}C NMR (151 MHz, CD_3CN) δ 175.2, 121.7 (q, $J_{C\text{-}F}$ = 319.5 Hz), 39.0, 36.7, 26.7.

^{19}F NMR (376 MHz, CD_3CN) δ -79.4 ppm.

The data is available in the literature.[1]

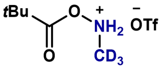

[N-2]

Name: N-(methyl-d$_3$)-O-pivaloylhydroxylammonium trifluoromethanesulfonate [N-2]

Yield: 64% for step 2, 56% for step 3.

Physical property: white solid

^{1}H NMR (400 MHz, $CDCl_3$) δ 9.98 (brs, 2H), 1.31 (s, 9H).

^{13}C NMR (151 MHz, $CDCl_3$) δ 174.3, 119.8 (q, J_{C-F} = 318.1 Hz), 38.4, 35.8 (hept, J_{C-D} = 22.3 Hz), 26.5.

^{19}F NMR (376 MHz, $CDCl_3$) δ -78.4 ppm.

HRMS (ESI) calculated for $C_6H_{11}D_3NO_2S^+$ $[M\text{-}OTf]^+$ 135.1207, found 135.1206.

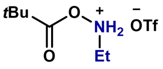

[N-3]

Name: N-ethyl-O-pivaloylhydroxylammonium trifluoromethanesulfonate [N-3]

Yield: 73% for step 2, 72% for step 3.

Physical property: white solid

^{1}H NMR (400 MHz, CD_3CN) δ 10.69 (brs, 2H), 3.53 (q, J = 7.3 Hz, 2H), 1.34 (t, J = 7.3 Hz, 3H), 1.30 (s, 9H).

^{13}C NMR (151 MHz, CD_3CN) δ 175.3, 121.6 (q, J_{C-F} = 319.3 Hz), 46.9, 39.2, 26.7, 9.0.

^{19}F NMR (376 MHz, CD_3CN) δ -79.4 ppm.

The data is available in the literature.[1]

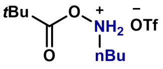

[N-4]

Name: N-butyl-O-pivaloylhydroxylammonium trifluoromethanesulfonate [N-4]

Yield: 82% for step 2, 71% for step 3.

Physical property: white solid

^{1}H NMR (400 MHz, $CDCl_3$) δ 11.38 (brs, 2H), 3.66 – 3.33 (m, 2H), 1.93 – 1.72 (m, 2H), 1.54 – 1.38 (m, 2H), 1.32 (s, 9H), 0.98 (t, J = 7.3 Hz, 3H).

^{13}C NMR (151 MHz, $CDCl_3$) δ 174.5, 119.8 (q, J_{C-F} = 318.1 Hz), 50.1, 38.5, 26.5, 25.3, 19.5.

^{19}F NMR (376 MHz, $CDCl_3$) δ -78.4 ppm.

HRMS (ESI) calculated for $C_9H_{20}NO_2S^+$ $[M\text{-}OTf]^+$ 174.1489, found 174.1487.

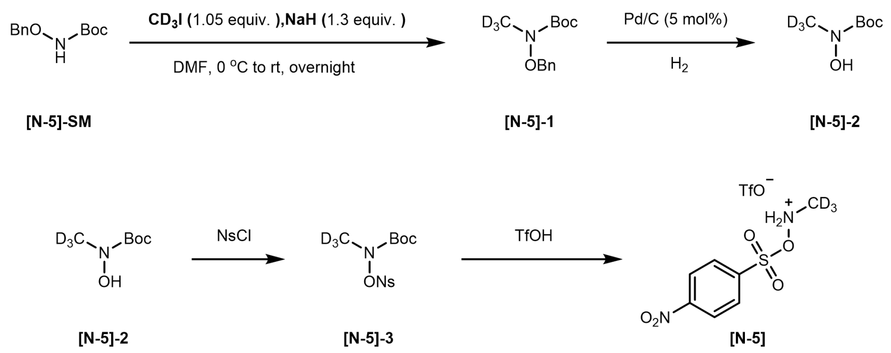

[N-5] was synthesized according to the literature with slight modifications.[2]

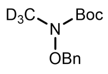

[N-5]-1

Name: *tert*-Butyl (benzyloxy)(methyl-d$_3$)carbamate [N-5]-1

NaH (60% in mineral oil; 4.3 g, 108.0 mmol, 1.3 equiv.) was added portion wise to a stirred solution of **[N-5]-SM** (11.2 g, 50.0 mmol, 1.0 equiv.) in anhydrous DMF (200 mL) at 0 °C. The reaction mixture was stirred at 0 °C for 30 min. After that, CD_3I (3.3 mL, 52.5 mmol, 1.05 equiv.) was added slowly and the reaction mixture was stirred at room temperature for 16 hours. The reaction mixture was quenched by slow addition of water (100 mL) at 0 °C. The aqueous reaction mixture was extracted with EA (3 × 100 mL). The combined organic layers were washed with brine, dried over anhydrous Na_2SO_4 and concentrated under reduced pressure. The crude product was purified by flash column chromatography (SiO_2; Hex/EtOAc=20:1 to 10:1, v/v). The title compound was obtained as a yellow oil in > 95% yield (12.0 g, 50.0 mmol).

^{1}H NMR (400 MHz, $CDCl_3$) δ 7.47–7.29 (m, 5H), 4.83 (s, 2H), 1.50 (s, 9H).

^{13}C NMR (151 MHz, $CDCl_3$) δ 157.0, 135.6, 129.4, 128.44, 128.37, 81.2, 76.5, 36.1 (hept, $J_{C\text{-}D}$ = 21.2 Hz), 28.2.

HRMS (ESI) calculated for $C_{13}H_{16}D_3NO_3Na^+$ $[M+Na]^+$ 263.1445, found 263.1449.

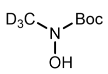

[N-5]-2

Name: *tert*-Butyl hydroxy(methyl-d$_3$)carbamate [N-5]-2

[N-5]-1 (3.6 g, 50.0 mmol, 1.0 equiv.) was dissolved in MeOH (300 mL), followed by addition of Pd/C (5 wt%). The reaction was stirred for 5 hours under a hydrogen atmosphere, after which TLC confirmed the complete conversion of starting material. The crude product was purified by flash column chromatography (SiO_2;

Hex/EtOAc=10:1 to 5:1, v/v). The title compound was obtained as a colorless oil in 90% yield (13.5 g, 13.5 mmol).

^{1}H NMR (400 MHz, $CDCl_3$) δ 7.75 (s, 1H), 1.45 (s, 9H).

^{13}C NMR (151 MHz, $CDCl_3$) δ 157.7, 81.6, 37.2 (hept, $J_{C\text{-}D}$ = 21.3 Hz), 28.2.

HRMS (ESI) calculated for $C_6H_{10}D_3NO_3Na^+$ $[M+Na]^+$ 173.0976, found 173.0981.

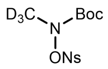

[N-5]-3

Name: *tert*-Butyl (methyl-d$_3$)(((4-nitrophenyl)sulfonyl)oxy)carbamate [N-5]-3

[N-5]-2 (13.5 g, 13.5 mmol, 1.0 equiv.) was dissolved in DCM (100 mL). The resulting solution was cooled to 0 °C, then triethylamine (1.9 mL, 13.5 mmol, 1.0 equiv.) and 4-nitrobenzenesulfonyl chloride (3.0 g, 13.5 mmol, 1.0 equiv.) were added sequentially during stirring. The reaction mixture was allowed to warm to room temperature and stirred for 16 hours. After that, the reaction mixture was washed with water (50 mL), sat. aq. $NaHCO_3$ (50 mL) and brine. The organic layer was dried over anhydrous Na_2SO_4 and concentrated under reduced pressure. The crude product was purified by flash column chromatography (SiO_2; Hex/EtOAc=8:1, v/v). The title compound was obtained as an off-white solid in 74% yield (3.4 g, 10.0 mmol).

^{1}H NMR (400 MHz, $CDCl_3$) δ 8.38 (d, J = 8.8 Hz, 2H), 8.19 (d, J = 8.7 Hz, 2H), 1.21 (s, 9H).

^{13}C NMR (151 MHz, $CDCl_3$) δ 155.6, 151.1, 139.8, 131.1, 123.9, 84.1, 40.0 (hept, $J_{C\text{-}D}$ = 32.5 Hz), 27.6.

HRMS (ESI) calculated for $C_{12}H_{13}D_3N_2O_7SNa^+$ $[M+Na]^+$ 358.0759, found 358.0762.

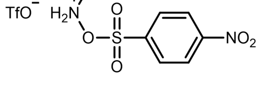

[N-5]-4

Name: N-(methyl-d$_3$)-O-((4-nitrophenyl)sulfonyl)hydroxylammonium trifluoromethanesulfonate [N-5]-4

[N-5]-3 (3.4 g, 10.0 mmol, 1.0 equiv.) was suspended in DCM (150 mL), which was cooled to 0 °C, followed by dropwise addition of triflic acid (0.9 mL, 10.0 mmol, 1.0 equiv.) under vigorous stirring. After that, the reaction mixture was allowed to warm to room temperature and stirred for 2 hours. The precipitate was filtered off, washed with diethyl ether (3 × 20 mL) and dried under reduced pressure. The title compound was obtained as a white solid in 53% yield (2.1 g, 5.3 mmol) and was used without further purification.

^{1}**H NMR** (400 MHz, CD_3CN) δ 10.87 (brs, 2H), 8.51 – 8.42 (m, 2H), 8.31 – 8.22 (m, 2H).

^{13}**C NMR** (151 MHz, CD_3CN) δ 153.2, 139.4, 131.9, 126.0, 121.3 (q, $J_{C\text{-}F}$ = 319.4 Hz), 38.5 (hept, $J_{C\text{-}D}$ = 22.4 Hz).

^{19}**F NMR** (376 MHz, CD_3CN) δ -79.4 ppm.

HRMS (ESI) calculated for $C_7H_6D_3N_2O_5S^+$ $[M\text{-}OTf]^+$ 236.0415, found 236.0420.

4. Reaction Conditions Optimization

4.1 Iron-catalyzed *ortho*-selective C−H N-CH_3 amination

These reactions were performed following the ***General Procedure A*** *in a 0.2 mmol scale.*

Table S1[a]

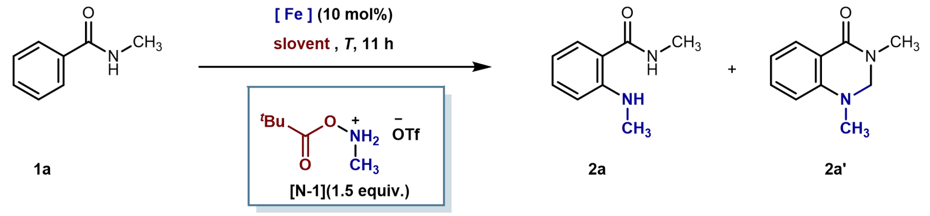

Entry	Solvent	Temp. (°C)	[Fe]	Conversion (%)	Product **2a** (%)	Product **2a'** (%)
1	EA	30	$Fe(OAc)_2$	14	n.d.	n.d.
2	MeOH	30	$Fe(OAc)_2$	-	n.d.	n.d.
3	PhF	30	$Fe(OAc)_2$	12	n.d.	5
4	DCM	30	$Fe(OAc)_2$	29	22	14
**5**	**HFIP**	**30**	**$Fe(OAc)_2$**	**85**	**76(74)**[b]	**7(6)**[b]
6	1,4-Dioxane	30	$Fe(OAc)_2$	10	n.d.	n.d.
7	CH_3CN	30	$Fe(OAc)_2$	45	7	19
8	HFIP	30	$FeCl_2$	84	57	11
9	HFIP	30	$FeBr_2$	84	72	11
10	HFIP	30	$Fe(OTf)_2$	80	63	13
11	HFIP	30	$Fe(acac)_2$	83	62	12
12	HFIP	30	FePc	4	n.d.	n.d.
13	HFIP	30	FeGly	88	53	13
14	HFIP	30	$FeCl_2·4H_2O$	87	55	9

[a]Reaction yields were monitored by ^{1}H NMR spectroscopy with CH_2Br_2 as the external standard. [b]Isolated yields are given in parentheses.

We attribute the formation of **2a'** to the involement of imine or iminium, generated via deprotonation of *α*-C−H bond of iron-aminyl radical and the

subsequent single electron oxidation. **2a’** was generated via condensation of imine with **2a** (Fig. S1). In addition, **2a”** was also observed in trace amount under certain conditions, which we ascribed to the iron-catalyzed directed C−H amination of **2a’** (see below).

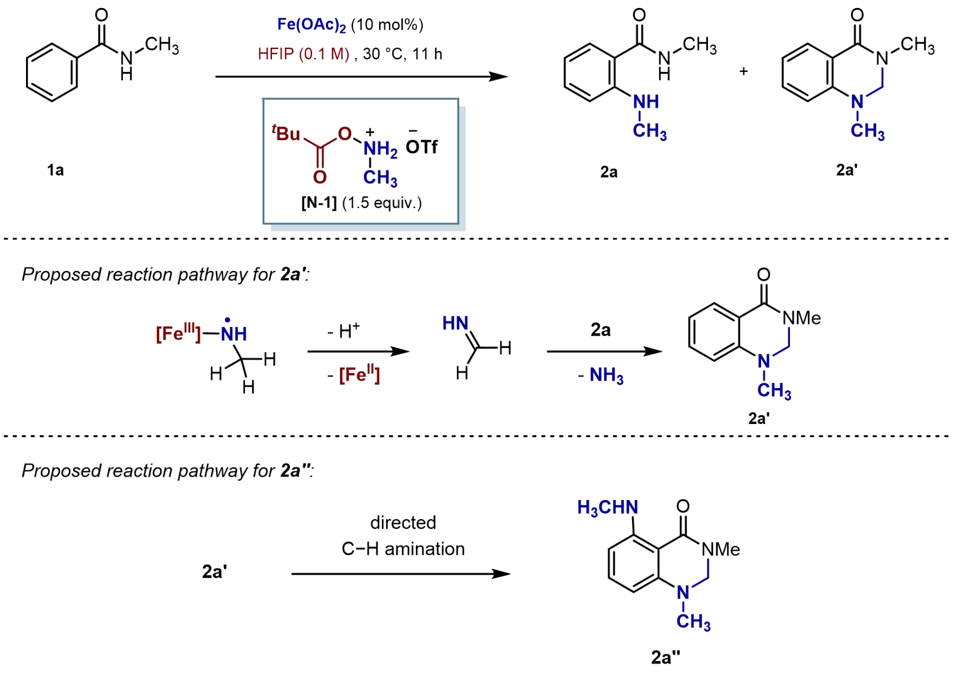

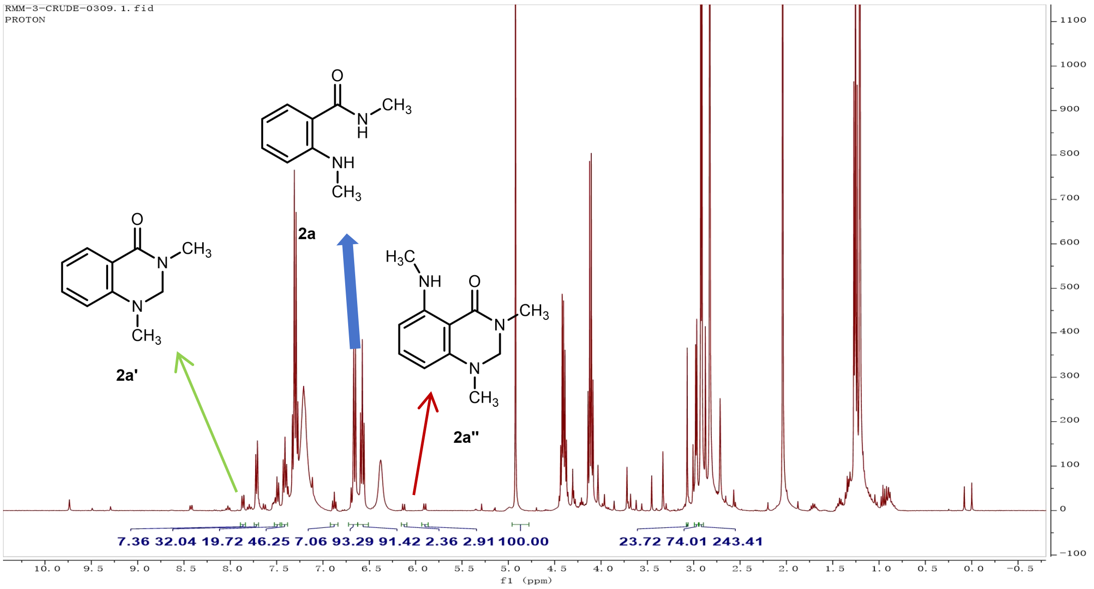

Figure S1. Mechanistic proposal for the formation of side products and the crude ^{1}H-NMR spectrum of the reaction mixture

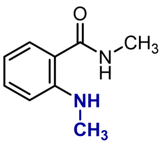

2a

Name: N-methyl-2-(methylamino)benzamide (2a)

Procedure: The reaction was conducted following the **General Procedure A** on a 0.8 mmol scale. The reaction mixture was purified by flash column chromatography with (PE/EA = 5:1 to 2:1, v/v) as elute to afford 97.1 mg of **2a**.

Isolated Yield: 74%

Physical Property: colorless oil

^{1}H NMR (400 MHz, $CDCl_3$) δ 7.47 (brs, 1H), 7.35 – 7.27 (m, 1H), 6.67 (d, J = 8.3 Hz, 1H), 6.57 (t, J = 7.5 Hz, 1H), 6.06 (brs, 1H), 2.95 (d, J = 4.9 Hz, 3H), 2.85 (s, 3H).

^{13}C NMR (151 MHz, $CDCl_3$) δ 170.5, 150.3, 132.7, 127.0, 115.3, 114.5, 111.1, 29.7, 26.5.

The data is available in the literature.[3]

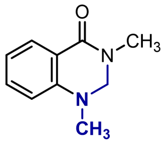

2a'

Name: 1,3-dimethyl-2,3-dihydroquinazolin-4(1H)-one(2a')

Procedure: The reaction was conducted following the **General Procedure A** on a 0.8 mmol scale. The reaction mixture was purified by flash column chromatography with (PE/EA = 5:1 to 2:1, v/v) as elute to afford 8.5 mg of **2a'**.

Isolated Yield: 6%

Physical Property: colorless oil

^{1}H NMR (400 MHz, DMSO-d_6) δ 7.72 (d, J = 7.6 Hz, 1H), 7.39 (t, J = 7.8 Hz, 1H), 6.91 – 6.71 (m, 2H), 4.45 (s, 2H), 2.95 (s, 3H), 2.83 (s, 3H).

^{13}C NMR (151 MHz, DMSO-d_6) δ 162.8, 149.5, 133.2, 127.8, 118.0, 116.9, 112.2, 67.1, 35.1, 31.9.

The data is available in the literature.[4]

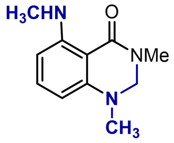

2a''

Name: 1,3-dimethyl-5-(methylamino)-2,3-dihydroquinazolin-4(1H)-one (2a'')

Procedure: The reaction was conducted following the **General Procedure A** on a 0.8

mmol scale. The reaction mixture was purified by flash column chromatography with (PE/EA = 5:1 to 2:1, v/v) as elute to afford 3 mg of **2a''**.

Isolated Yield: 2%

Physical Property: white solid

^{1}H NMR (400 MHz, $CDCl_3$) δ 8.59 (brs, 1H), 7.18 (t, J = 8.2 Hz, 1H), 6.13 (d, J = 8.4 Hz, 1H), 5.89 (d, J = 8.0 Hz, 1H), 4.31 (s, 2H), 3.03 (s, 3H), 2.85 (d, J = 5.0 Hz, 3H), 2.83 (s, 3H).

^{13}C NMR (151 MHz, $CDCl_3$) δ 167.0, 152.6, 151.0, 134.2, 101.9, 99.4, 98.4, 68.2, 36.5, 32.2, 29.5.

HRMS (ESI) calculated for $C_{11}H_{15}N_3OH^+$ $[M+H]^+$ 206.1288, found 206.1287.

4.2 Iron-catalyzed *ortho*-selective C−H N-CD$_3$ amination

*These reactions were performed following the **General Procedure B** in a 0.2 mmol scale.*

Table S2[a]

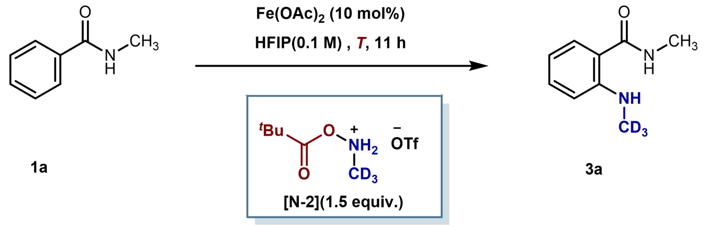

Entry	Solvent	Temp[*T*] (°C)	**additive**	Conversion (%)	Product **3a** (%)
1	HFIP	30	1.0 equiv. H_2O	91	91
2	HFIP	30	5.0 equiv. H_2O	62	49
3	HFIP	30	10.0 equiv. H_2O	24	12
4	HFIP	30	20.0 equiv. H_2O	11	0
5	HFIP	0	-	90	81
6	HFIP	10	-	94	85
7	HFIP	20	-	93	84
**8**	**HFIP**	**30**	**-**	**94**	**94**
9[b]	HFIP	30	-	0	n.d.
10[c]	HFIP	30	-	82	82

[a]Reaction yields were monitored by ^{1}H NMR spectroscopy with CH_2Br_2 as the external standard. [b]Without $Fe(OAc)_2$. [c]Under air.

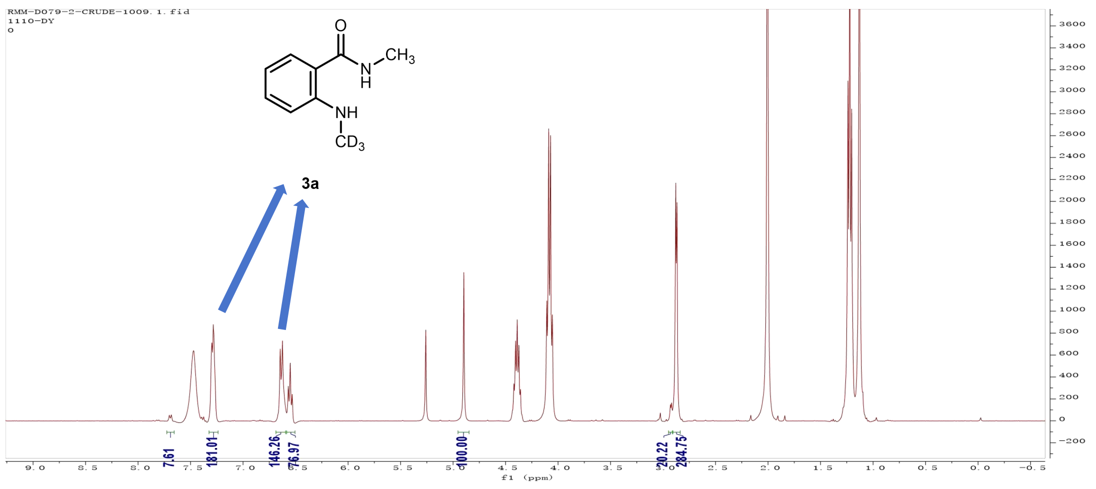

Figure S2. The crude ^{1}H-NMR spectrum of the reaction mixture

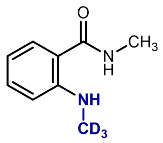

3a

Name: N-methyl-2-((methyl-d$_3$)amino)benzamide (3a)

Procedure: The reaction was conducted following the **General Procedure B** on a 0.4 mmol scale. The reaction mixture was purified by flash column chromatography with (PE/EA/DCM = 1:1:1, v/v/v) as elute to afford 56.8 mg of **3a**.

Isolated Yield: 85%

Physical Property: colorless oil

^{1}H NMR (400 MHz, $CDCl_3$) δ 7.39 (brs, 1H), 7.34 – 7.26 (m, 1H), 6.65 (d, J = 8.8 Hz, 1H), 6.56 (t, J = 7.5 Hz, 1H), 6.13 (brs, 1H), 2.93 (d, J = 4.8 Hz, 3H).

^{13}C NMR (151 MHz, $CDCl_3$) δ 170.5, 150.4, 132.7, 127.1, 115.3, 114.4, 111.0, 28.9 (hept, $J_{C\text{-}D}$ = 20.6 Hz), 26.4.

HRMS (ESI) calculated for $C_9H_9D_3N_2OH^+$ $[M+H]^+$ 168.1211 found 168.1207.

5. Spin Trap Experiments

Continuous-wave (CW) electron paramagnetic resonance (EPR) measurements were performed under argon atmosphere at room temperature with an Elexsys E 580 X-band spectrometer at microwave frequencies of about 9.88 GHz, a microwave power of 15 mW and a modulation amplitude of 0.05 mT in order to avoid line broadening by saturation or overmodulation.

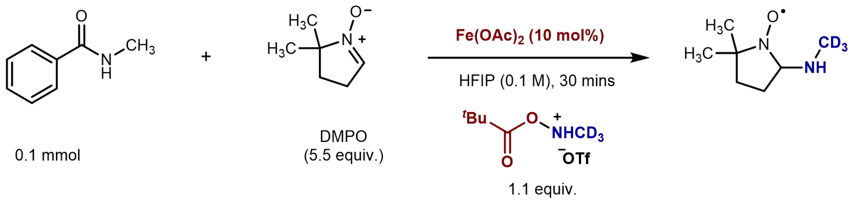

The reaction was conducted following the **General Procedure B** in a 0.1 mmol scale, albeit with DMPO as additive. In the glovebox, a 10 mL glass vial was charged with $Fe(OAc)_2$ (0.01 mmol, 10 mol%), aminating reagent **[N-2]** (0.11 mmol, 31.2 mg, 1.1 equiv.), arene substrate (0.1 mmol, 1.0 equiv.) and HFIP (1 mL, 0.1 M). Then, DMPO (0.55 mmol, 62.2 mg, 5.5 equiv.) was added into the reaction mixture, which was further stirred for 0.5 h. An aliquot (cat. 10.0 μL) of reaction mixture was then taken out for EPR analysis.

EPR spectrum of the radical generated upon mixing $Fe(OAc)_2$, [$PivONHCD_3HOTf$], DMPO and SM at room temperature (a) and the corresponding theoretical simulation (b) obtained with the following spectroscopic parameters: g = 2.0063, A_N = 13.67 G, A_H = 18.57 G, A_N = 3.28 G.

The signals of c-e refer to EPR spectrum of control experiments. 'Conditions of a, but without iron catalyst' (c), '**[N-2]** and DMPO' (d) and 'SM and **[N-2]**' (e) at room temperature.

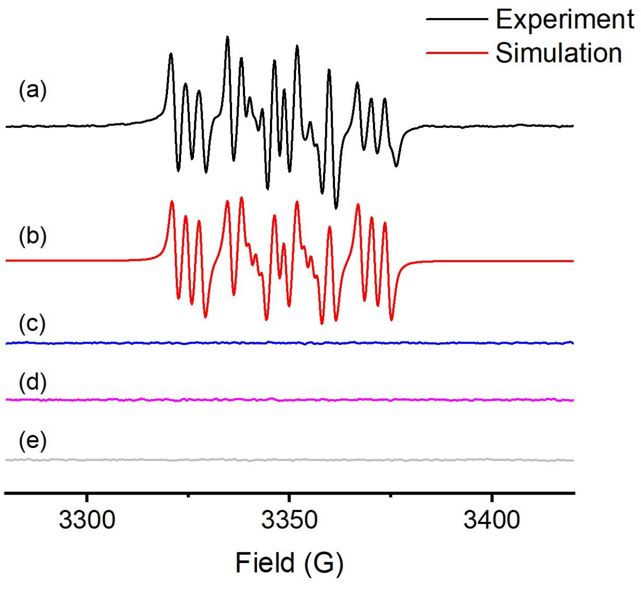

Figure S3. EPR spectrum of the spin trap experiment

6. Reactions with Other N-Alkyl Aminating Reagents.

6.1 Reaction with [N-3]

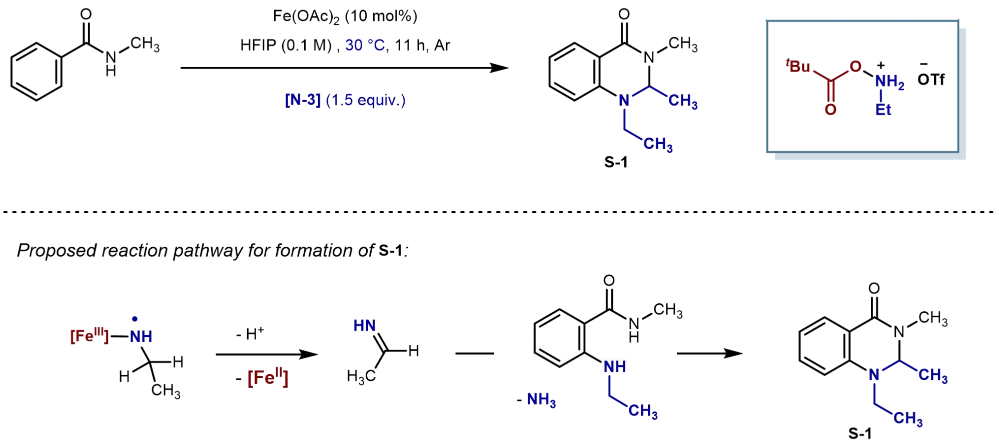

Figure S4. Reaction of C−H N-ethyl amination and mechanistic proposal

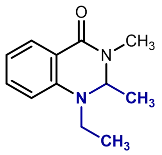

Name: 1-ethyl-2,3-dimethyl-2,3-dihydroquinazolin-4(1H)-one (S-1)

Procedure: The reaction was conducted following the **General Procedure B** on a 0.4 mmol scale with **[N-3]** as the aminating reagent. The reaction mixture was purified by flash column chromatography with (PE/EA = 2:1, v/v) as elute to afford 20.4 mg of **S-1**.

Isolated Yield: 25%

Physical Property: colorless oil

^{1}H NMR (400 MHz, $CDCl_3$) δ 7.93 (d, *J* = 7.8 Hz, 1H), 7.33 (t, *J* = 7.8 Hz, 1H), 6.80 (t, *J* = 7.5 Hz, 1H), 6.67 (d, *J* = 8.3 Hz, 1H), 4.62 (q, *J* = 6.0 Hz, 1H), 3.51 – 3.37 (m, 1H), 3.18 – 3.08 (m, 1H), 3.06 (s, 3H), 1.249 (t, *J* = 7.1 Hz, 3H), 1.248 (d, *J* = 5.9 Hz, 3H).

^{13}C NMR (151 MHz, $CDCl_3$) δ 162.7, 145.4, 133.2, 128.6, 117.9, 117.2, 113.1, 72.4, 43.7, 32.2, 15.8, 13.3.

HRMS (ESI) calculated for $C_{12}H_{16}N_2OH^+$ $[M+H]^+$ 205.1335, found 205.1341.

6.2 Reaction with [N-4]

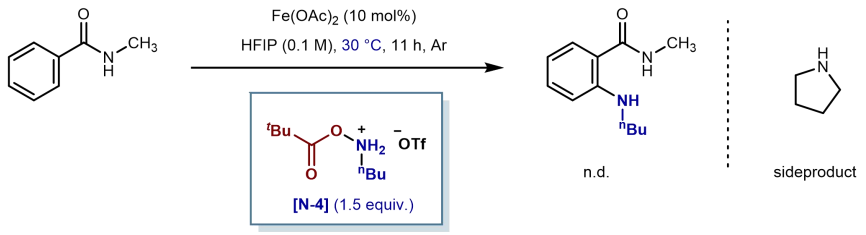

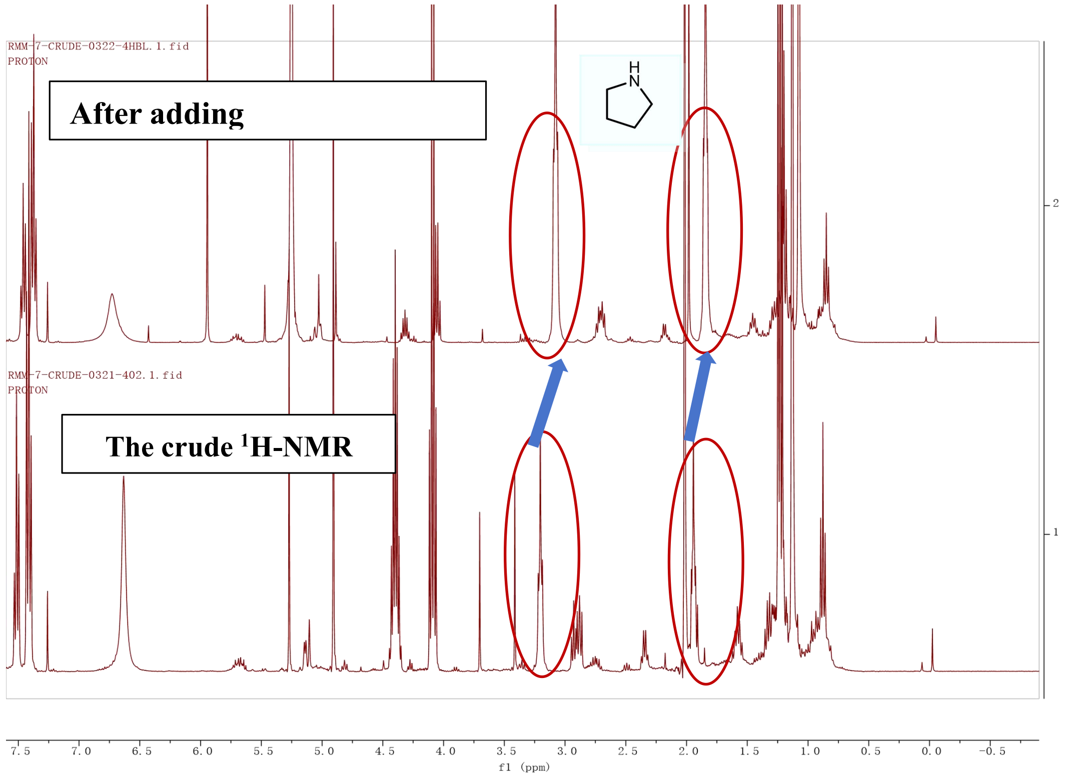

Figure S5. The crude ^{1}H-NMR spectrum of reaction with **[N-4]**

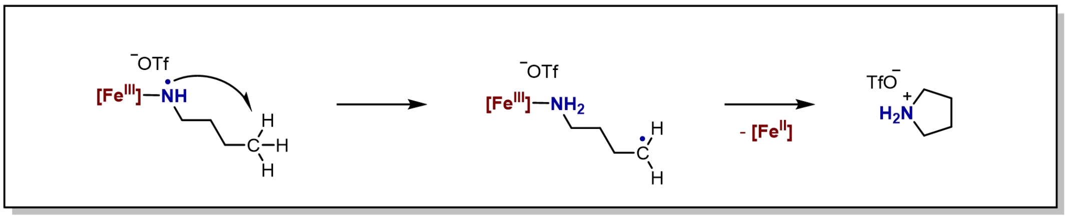

Figure S6. Proposed reaction pathway for tetrahydropyrrole formation

7. Comparison between Aminium Radical Cation and Iron-Aminyl Radical

The reactions were conducted following the **General Procedure B** in a 0.2 mmol scale.

Table S3[a]

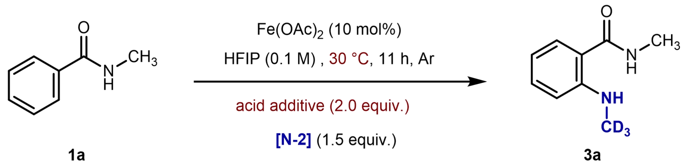

Entry	Solvent	Temp. (°C)	additive	Conversion (%)	Product **3a** (%)
1	HFIP	30	none	94	94
2	HFIP	30	$MeSO_3H$	0	n.d.
3	HFIP	30	TfOH	0	n.d.
4	HFIP	30	TFA	70	50
5	HFIP	30	CCl_3COOH	59	31
6[b]	HFIP	30	-	0	n.d.

[a]Reaction yields were monitored by ^{1}H NMR spectroscopy with CH_2Br_2 as the external standard. [b]Using **[N-5]** as the aminating reagent.

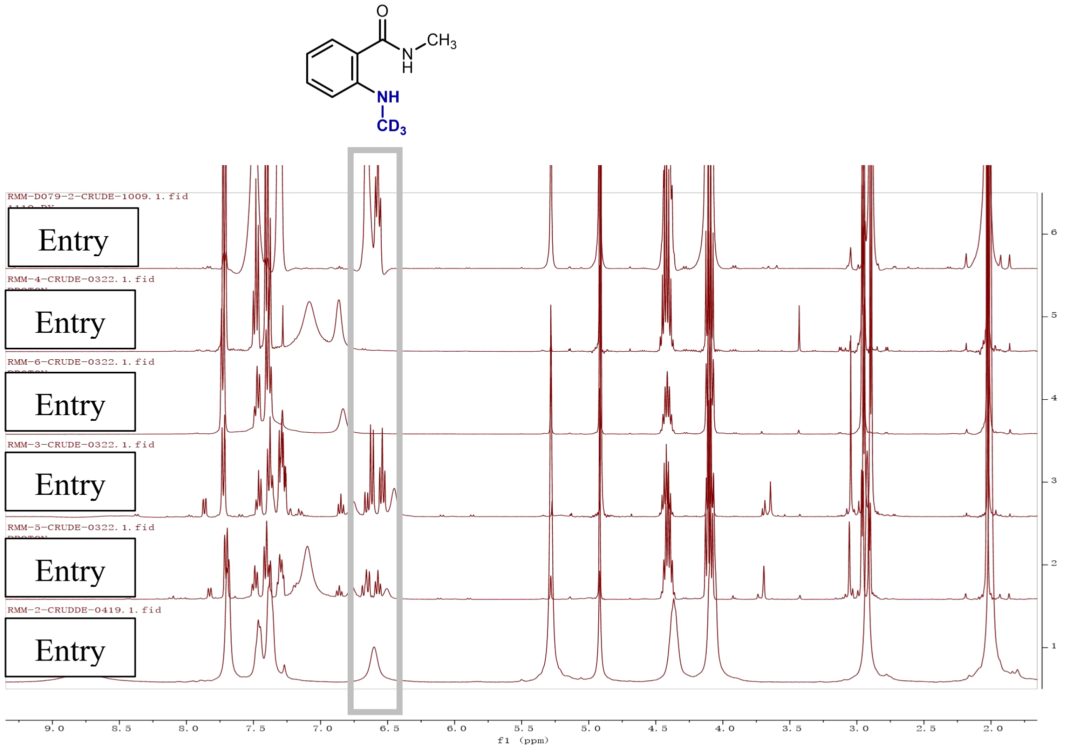

Figure S7. The crude ^{1}H-NMR spectrum of reactions shown in Table S3.

These results indicated that the iron-aminyl radical intermediate may undergo protonation to form an ammonium radical cation, thereby diverting the catalytic cycle. When using **[N-5]** as the aminating reagent, no product was observed, implying that aminium radical cation failed to undergo addition to the electron-deficient substrate.

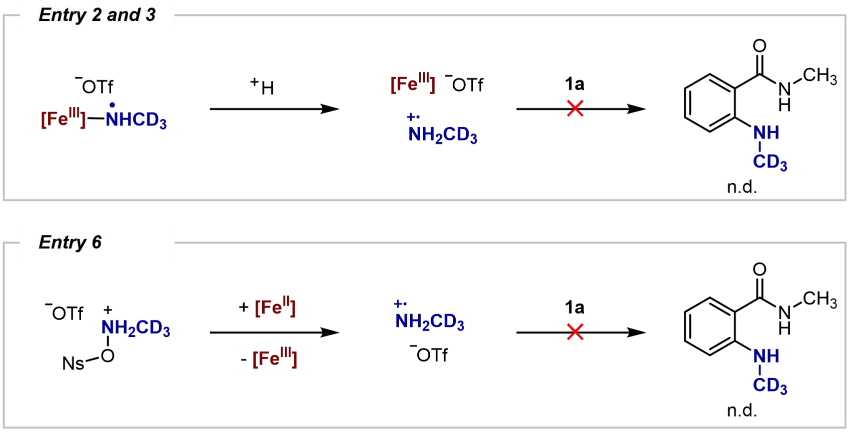

Figure S8. The lack of reactivity with aminium radical cation

8. Experimental Procedures and Characterization Data

Substrates:

A Summary of Commercially Available Substrates

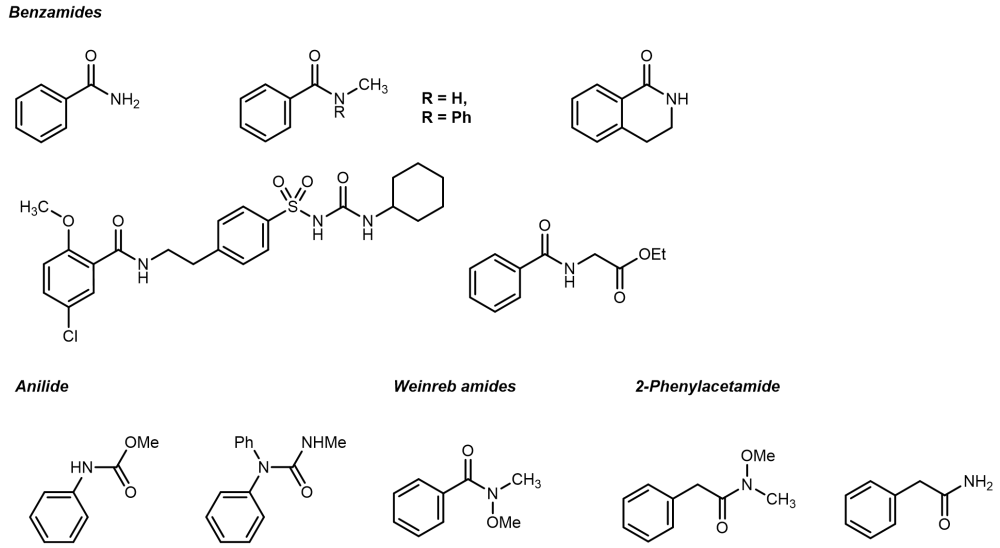

A Summary of Synthesized Substrates

Benzamides

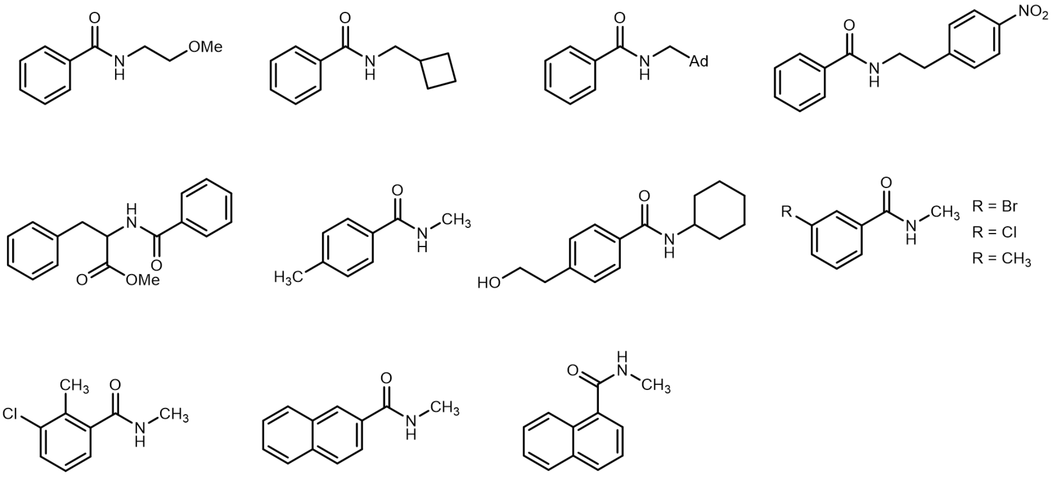

2-Phenylacetamide

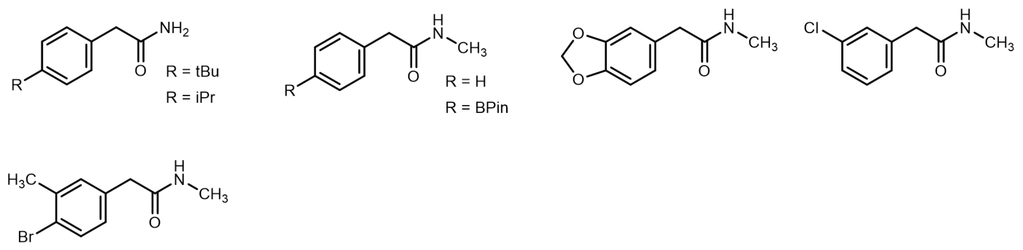

P(V)=O

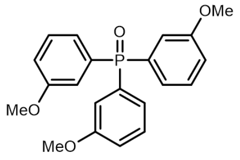

Aryl sulfoxides

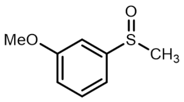

General Procedure C

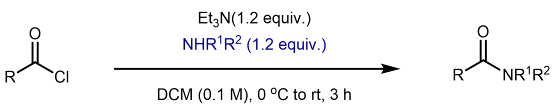

To a solution of alkylamines (1.20 equiv.) and Et_3N (1.20 equiv.) in DCM (100 mL) was added acyl chloride (10.0 mmol) at 0 °C. The reaction mixture was stirred at room temperature about 3 hours at which point TLC analysis indicated the complete conversion of acyl chloride. And then the resulting suspension was washed with 10% critic acid in water and saturated aqueous $NaHCO_3$, dried over Na_2SO_4.The solvent was then removed under reduced pressure, the title compound was obtained without further purification.

General Procedure D

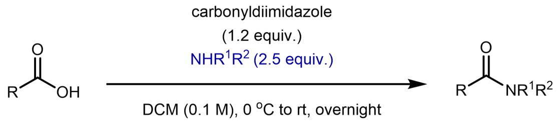

To a solution of carboxylic acid (1.0 equiv.) in DCM (100 mL per 10 mmol of acid) at 0 °C was added carbonyldiimidazole (1.2 equiv.). The resulting solution was stirred for 1 hour at 0 °C. Then, to this solution was added amine (2.5 equiv.) and the reaction was stirred overnight at room temperature, at which point TLC analysis indicated the complete conversion of the acid. The resulting suspension was washed with 10% critic acid in water and saturated aqueous $NaHCO_3$, dried over Na_2SO_4. The solvent was then removed under reduced pressure, the title compound was obtained without further purification.

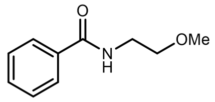

Name: N-(2-methoxyethyl)benzamide (1e)

Procedure: The reaction was conducted following the **General Procedure C** on a 10.0 mmol scale. The title compound was obtained as a white solid in 92% yield (1.6 g, 9.2 mmol).

Isolated Yield: 92%

Physical Property: yellow oil

^{1}H NMR (400 MHz, $CDCl_3$) δ 7.83 – 7.71 (m, 2H), 7.54 – 7.46 (m, 1H), 7.47 – 7.38 (m, 2H), 6.56 (brs, 1H), 3.65 (q, J = 5.2 Hz, 2H), 3.56 (t, J = 5.0 Hz, 1H), 3.38 (s, 3H).

^{13}C NMR (151 MHz, $CDCl_3$) δ 167.4, 134.5, 131.4, 128.5, 126.9, 71.2, 58.8, 39.6.

The data is available in the literature.[5]

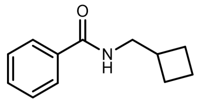

Name: N-(cyclobutylmethyl)benzamide (1f)

Procedure: The reaction was conducted following the **General Procedure C** on a 10.0 mmol scale. The title compound was obtained as a white solid in 95% yield (1.8 g, 9.5 mmol).

Isolated Yield: 95%

Physical Property: yellow solid

^{1}H NMR (400 MHz, $CDCl_3$) δ 7.81 – 7.71 (m, 2H), 7.54 – 7.45 (m, 1H), 7.41 (dd, J =

8.4, 6.9 Hz, 2H), 6.15 (brs, 1H), 3.48 (dd, J = 7.3, 5.8 Hz, 1H), 2.58 (hept, J = 7.6 Hz, 1H), 2.20 – 2.02 (m, 2H), 2.01 – 1.84 (m, 2H), 1.84 – 1.63 (m, 2H).

^{13}C NMR (151 MHz, $CDCl_3$) δ 167.7, 134.8, 131.2, 128.4, 126.9, 45.2, 35.0, 25.7, 18.3.

The data is available in the literature.[6]

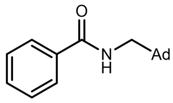

Name: N-(((3r,5r,7r)-adamantan-1-yl)methyl)benzamide (1g)

Procedure: The reaction was conducted following the **General Procedure C** on a 10.0 mmol scale. The title compound was obtained as a white solid in 82% yield (2.2 g, 8.2 mmol).

Isolated Yield: 82%

Physical Property: light yellow solid

^{1}H NMR (400 MHz, $CDCl_3$) δ 7.81 – 7.73 (m, 2H), 7.53 – 7.46 (m, 1H), 7.46 – 7.39 (m, 2H), 6.20 (brs, 1H), 3.16 (d, J = 6.3 Hz, 2H), 2.04 – 1.95 (m, 3H), 1.77 – 1.68 (m, 3H), 1.68 – 1.60 (m, 3H), 1.60 – 1.52 (m, 6H).

^{13}C NMR (151 MHz, $CDCl_3$) δ 167.7, 135.1, 131.3, 128.5, 126.8, 51.3, 40.3, 36.9, 34.0, 28.2.

HRMS (ESI) calculated for $C_{18}H_{23}NONa^+$ $[M+Na]^+$ 292.1672, found 292.1672.

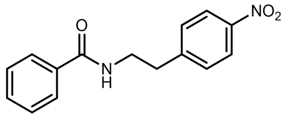

Name: N-(4-nitrophenethyl)benzamide (1i)

Procedure: The reaction was conducted following the **General Procedure C** on a 10.0 mmol scale. The residue was purified through flash column chromatography with PE/EA (5:1, v/v) as elute to afford 2.1 g of the titled product.

Isolated Yield: 77%

Physical Property: white solid

^{1}H NMR (400 MHz, $CDCl_3$) δ 8.18 (d, J = 8.5 Hz, 2H), 7.70 (d, J = 7.4 Hz, 2H), 7.51 (t, J = 7.3 Hz, 1H), 7.46 – 7.37 (m, 4H), 6.22 (brs, 1H), 3.75 (q, J = 6.7 Hz, 2H), 3.07 (t, J = 7.0 Hz, 2H).

^{13}C NMR (151 MHz, $CDCl_3$) δ 167.6, 146.81, 146.77, 134.2, 131.7, 129.7, 128.6, 126.8, 123.8, 40.7, 35.7.

The data is available in the literature.[7]

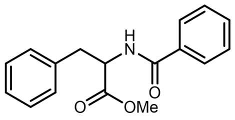

Name: methyl benzoylphenylalaninate (1j)

Procedure: The reaction was conducted following the **General Procedure D** on a 5.0 mmol scale. The residue was purified through flash column chromatography with PE/EA (1:1, v/v) as elute to afford 0.69 g of the titled product.

Isolated Yield: 49%

Physical Property: white solid

^{1}H NMR (400 MHz, $CDCl_3$) δ 7.83 – 7.65 (m, 2H), 7.50 (t, *J* = 7.4 Hz, 1H), 7.41 (t, *J* = 7.5 Hz, 2H), 7.33 – 7.23 (m, 4H), 7.18 – 7.09 (m, 1H), 6.59 (d, *J* = 7.6 Hz, 1H), 5.09 (q, *J* = 6.2 Hz, 1H), 3.76 (s, 3H), 3.29 (dd, *J* = 13.8, 5.7 Hz, 1H), 3.22 (dd, *J* = 13.8, 5.2 Hz, 1H)..

^{13}C NMR (151 MHz, $CDCl_3$) δ 172.0, 166.8, 135.8, 133.8, 131.7, 129.3, 128.6, 127.1, 126.9, 53.5, 52.4, 37.8.

The data is available in the literature.[8]

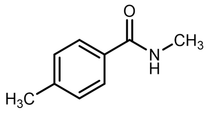

Name: N,4-dimethylbenzamide (1k)

Procedure: The reaction was conducted following the **General Procedure C** on a 10.0 mmol scale. The title compound was obtained as a white solid in 87% yield (1.3 g, 8.7 mmol).

Isolated Yield: 87%

Physical Property: white solid

^{1}H NMR (400 MHz, $CDCl_3$) δ 7.66 (d, *J* = 8.0 Hz, 2H), 7.17 (d, *J* = 7.9 Hz, 2H), 6.65 (brs, 1H), 2.94 (d, *J* = 4.8 Hz, 3H), 2.35 (s, 3H).

^{13}C NMR (151 MHz, $CDCl_3$) δ 168.2, 141.5, 131.6, 129.0, 126.8, 26.7, 21.3.

The data is available in the literature.[9]

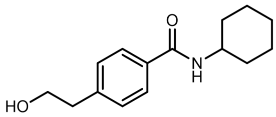

Name:N-cyclohexyl-4-(2-hydroxyethyl)benzamide (1m)

Procedure: The reaction was conducted following the **General Procedure D** on a 10.0 mmol scale. The title compound was obtained as a white solid in 76% yield (1.9 g, 7.6 mmol).

Isolated Yield: 76%

Physical Property: white solid

^{1}H NMR (400 MHz, $CDCl_3$) δ 7.72 – 7.64 (m, 2H), 7.29 – 7.25 (m, 2H), 5.92 (brs, 1H), 4.00 – 3.90 (m, 1H), 3.85 (t, *J* = 6.6 Hz, 2H), 2.89 (t, *J* = 6.7 Hz, 2H), 2.08 – 1.90 (m, 2H), 1.80 – 1.69 (m, 2H), 1.68 – 1.58 (m, 1H), 1.47 – 1.33 (m, 2H), 1.29 – 1.13 (m, 3H).

^{13}C NMR (151 MHz, $CDCl_3$) δ 166.5, 142.3, 133.2, 129.1, 127.0, 63.3, 48.6, 38.9, 33.2, 25.5, 24.9.

HRMS (ESI) calculated for $C_{15}H_{21}NO_2Na^+$ $[M+Na]^+$ 270.1463, found 270.1464.

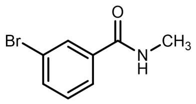

Name: 3-bromo-N-methylbenzamide (1n)

Procedure: The reaction was conducted following the **General Procedure D** on a 10.0 mmol scale. The title compound was obtained as a white solid in 66% yield (1.4 g, 6.6 mmol).

Isolated Yield: 66%

Physical Property: white solid

^{1}H NMR (400 MHz, $CDCl_3$) δ 7.90 (t, *J* = 1.8 Hz, 1H), 7.67 (d, *J* = 7.8 Hz, 1H), 7.64 – 7.57 (m, 1H), 7.29 (t, *J* = 7.9 Hz, 1H), 6.23 (brs, 1H), 3.01 (d, *J* = 4.8 Hz, 3H).

^{13}C NMR (151 MHz, $CDCl_3$) δ 167.1, 136.2, 134.5, 131.2, 129.7, 127.2, 125.0, 26.8.

The data is available in the literature.[10]

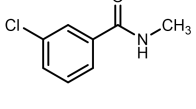

Name:3-chloro-N-methylbenzamide (1o)

Procedure: The reaction was conducted following the **General Procedure D** on a 10.0 mmol scale. The title compound was obtained as a white solid in 79% yield (1.3 g, 7.9 mmol).

Isolated Yield: 79%

Physical Property: white solid

^{1}**H NMR** (400 MHz, $CDCl_3$) δ 7.90 (t, *J* = 1.9 Hz, 1H), 7.67 (dt, *J* = 7.7, 1.4 Hz, 1H), 7.64 – 7.54 (m, 1H), 7.29 (td, *J* = 8.2, 2.6 Hz, 1H), 3.00 (d, *J* = 4.8 Hz, 3H).

^{13}**C NMR** (151 MHz, $CDCl_3$) δ 166.8, 136.6, 134.3, 130.1, 125.4, 122.7, 26.9.

The data is available in the literature.[10]

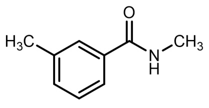

Name: N,3-dimethylbenzamide (1p)

Procedure: The reaction was conducted following the **General Procedure C** on a 10.0 mmol scale. The title compound was obtained as a white solid in 64% yield (0.95 g, 6.5 mmol).

Isolated Yield: 64%

Physical Property: white solid

^{1}**H NMR** (400 MHz, $CDCl_3$) δ 7.61 (s, 1H), 7.59 – 7.54 (m, 1H), 7.30 – 7.20 (m, 2H), 6.94 (brs, 1H), 2.95 (d, *J* = 4.5 Hz, 3H), 2.32 (s, 3H).

^{13}**C NMR** (151 MHz, $CDCl_3$) δ 168.5, 138.1, 134.4, 131.8, 128.1, 127.6, 123.8, 26.6, 21.1.

The data is available in the literature.[10]

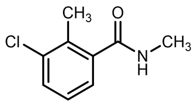

Name: 3-chloro-N,2-dimethylbenzamide (1q)

Procedure: The reaction was conducted following the **General Procedure D** on a 10.0 mmol scale. The resulting residue was purified through flash column chromatography with PE/EA (1:1, v/v) as elute, which was recrystallized in ethyl ether/n-hexane (1:1, v/v) to afford the titled product as a white solid in 15% yield (0.27 g, 1.5 mmol).

Isolated Yield: 15%

Physical Property: white solid

^{1}**H NMR** (400 MHz, $CDCl_3$) δ 7.69 (dd, *J* = 7.8, 1.4 Hz, 1H), 7.49 (dd, *J* = 8.0, 1.4 Hz, 1H), 7.16 (t, *J* = 7.9 Hz, 1H), 3.90 (s, 3H), 2.60 (s, 3H).

^{13}**C NMR** (101 MHz, $CDCl_3$) δ 167.8, 137.1, 136.0, 132.5, 128.5, 126.3, 52.2, 17.4.

The data is available in the literature.[11]

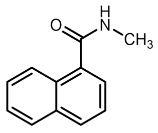

Name:N-methyl-1-naphthamide (1s)

Procedure: The reaction was conducted following the **General Procedure C** on a 10.0 mmol scale. The titled compound was obtained as a white solid in 80% yield (1.5 g, 8.0 mmol).

Isolated Yield: 80%

Physical Property: beige solid

^{1}H NMR (400 MHz, $CDCl_3$) δ 8.31 – 8.17 (m, 1H), 7.96 – 7.76 (m, 2H), 7.59 – 7.44 (m, 3H), 7.43 – 7.32 (m, 1H), 6.22 (brs, 1H), 2.99 (d, J = 4.8 Hz, 2H).

^{13}C NMR (151 MHz, $CDCl_3$) δ 170.2, 134.4, 133.6, 130.4, 130.0, 128.2, 126.9, 126.3, 125.4, 124.8, 124.6, 26.7.

The data is available in the literature.[12]

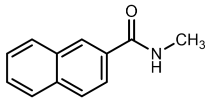

Name: N-methyl-2-naphthamide (1t)

Procedure: The reaction was conducted following the **General Procedure C** on a 10.0 mmol scale. The crude product was recrystallized in ethyl ether/n-hexane (1:1, v/v) to afford the titled compound as a white solid in 70% yield (1.3 g, 7.0 mmol).

Isolated Yield: 70%

Physical Property: white solid

^{1}H NMR (400 MHz, $CDCl_3$) δ 8.28 (s, 1H), 8.01 – 7.73 (m, 4H), 7.58 – 7.51 (m, 2H), 6.34 (brs, 1H), 3.08 (d, J = 4.8 Hz, 3H).

^{13}C NMR (151 MHz, $CDCl_3$) δ 168.4, 134.6, 132.6, 131.8, 128.8, 128.3, 127.7, 127.5, 127.3, 126.6, 123.5, 26.9.

The data is available in the literature.[13]

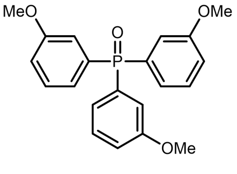

Name: tris(3-methoxyphenyl)phosphine oxide (1w)

Procedure: To a solution of tris(3-methoxyphenyl)phosphane (1.76 g, 5 mmol, 1

equiv.) in THF (4 mL per mmol) was added H_2O_2 (30% in water, 3 equiv.) slowly. The resulting mixture was then stirred at room temperature, which was monitored with TLC. After completion, the reaction was quenched with saturated $NaHCO_3$ and Na_2S2O_3, and stirred for a few more minutes. The reaction mixture was then separated, and the aqueous phase was extracted with DCM. The combined organic phases were combined and dried over Na_2SO_4 and volatiles were removed in vacuo to afford 1.7 g of the titled product, which was used without further purification.

Isolated Yield: 90%

Physical Property: white solid

^{1}H NMR (400 MHz, $CDCl_3$) δ 7.34 (dt, *J* = 7.9, 3.9 Hz, 3H), 7.30 – 7.21 (m, 3H), 7.18 – 7.09 (m, 3H), 7.08 – 7.02 (m, 3H), 3.79 (s,9H).

^{13}C NMR (151 MHz, $CDCl_3$) δ 159.6, 159.5, 134.0, 133.3, 129.7, 129.6, 124.35, 124.28, 118.22, 118.21, 116.7, 116.6, 55.4.

^{31}P NMR (162 MHz, $CDCl_3$) δ 29.9 ppm.

The data is available in the literature.[14]

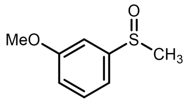

Name: 1-methoxy-3-(methylsulfinyl)benzene (1x)

Procedure: The (3-methoxyphenyl)(methyl)sulfane (1.0 equiv.) was re-dissolved in DCM (0.1 M) and cooled to 0 °C , to which was added slowly a solution of m-chloroperoxybenzoic acid (0.9 equiv.) in DCM (0.2 M). The resulting mixture was stirred for 1 h at the same temperature. After that, the reaction mixture was poured into saturated aqueous solution of $NaHCO_3$ (50 mL) with vigorous stirring and the layers were separated. The aqueous layer was extracted twice with DCM, and the combined organic phase was washed with saturated $NaHCO_3$, H_2O, brine, and dried over anhydrous Na_2SO_4. The solvent was removed under reduced pressure, and the residue was purified by flash column chromatography to give the titled product.

Isolated Yield: 87%

Physical Property: colorless oil

^{1}H NMR (400 MHz, $CDCl_3$) δ 7.39 (t, *J* = 8.0 Hz, 1H), 7.24 (s, 1H), 7.11 (d, *J* = 7.6 Hz, 1H), 7.00 (d, *J* = 8.3 Hz, 1H), 3.85 (s, 3H), 2.71 (s, 3H)..

^{13}C NMR (151 MHz, $CDCl_3$) δ 160.4, 147.1, 130.2, 117.4, 115.5, 107.8, 55.5, 43.9.

The data is available in the literature.[15]

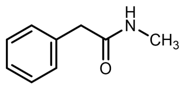

Name: N-methyl-2-phenylacetamide (1y)

Procedure: The reaction was conducted following the **General Procedure D** on a 10.0 mmol scale. The titled compound was obtained as a white solid in 74% yield (1.1 g, 7.4 mmol).

Isolated Yield: 74%

Physical Property: white solid

^{1}H NMR (400 MHz, $CDCl_3$) δ 7.39 – 7.31 (m, 2H), 7.31 – 7.27 (m, 1H), 7.27 – 7.21 (m, 2H), 5.70 (brs, 1H), 3.56 (s, 2H), 2.74 (d, J = 4.9 Hz, 2H).

^{13}C NMR (151 MHz, $CDCl_3$) δ 171.6, 134.9, 129.4, 128.9, 127.2, 43.6, 26.4.

The data is available in the literature.[16]

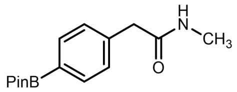

Name: N-methyl-2-(4-(4,4,5,5-tetramethyl-1,3,2-dioxaborolan-2-yl)phenyl)acetamide (1z)

Procedure: The reaction was conducted following the **General Procedure D** on a 5.0 mmol scale. The titled compound was obtained as a white solid in 72% yield (0.99 g, 3.6 mmol).

Isolated Yield: 72%

Physical Property: white solid

^{1}H NMR (400 MHz, $CDCl_3$) δ 7.97 – 7.75 (m, 2H), 7.38 – 7.03 (m, 2H), 5.37 (brs, 1H), 3.59 (d, J = 1.7 Hz, 2H), 2.73 (d, J = 4.9 Hz, 1H), 1.35 (s, 12H).

^{13}C NMR (151 MHz, $CDCl_3$) δ 171.2, 138.0, 135.4, 128.9, 83.8, 43.9, 26.4, 24.8.

^{11}B NMR (128 MHz, $CDCl_3$) δ 30.3 ppm.

The data is available in the literature.[17]

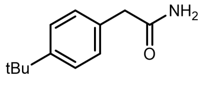

Name: 2-(4-(tert-butyl)phenyl)-N-methylacetamide (1aa)

Procedure: The reaction was conducted following the **General Procedure D** on a 5.0 mmol scale. The titled compound was obtained as a white solid in 76% yield (1.5 g,

7.6 mmol).

Isolated Yield: 76%

Physical Property: white solid

^{1}H NMR (400 MHz, $CDCl_3$) δ 7.48 – 7.32 (m, 2H), 7.24 – 7.10 (m, 2H), 5.63 (brs, 1H), 5.41 (brs, 1H), 3.56 (s, 2H), 1.32 (s, 9H).

^{13}C NMR (151 MHz, $CDCl_3$) δ 174.0, 150.3, 131.7, 129.0, 125.9, 42.8, 34.5, 31.3.

HRMS (ESI) calculated for $C_{12}H_{17}NONa^+$ $[M+Na]^+$ 214.1202, found 214.1204.

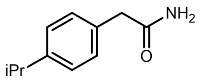

Name: 2-(4-isopropylphenyl)acetamide (1ab)

Procedure: The reaction was conducted following the **General Procedure D** on a 10.0 mmol scale. The titled compound was obtained as a white solid in 63% yield (1.1 g, 6.3 mmol).

Isolated Yield: 63%

Physical Property: white solid

^{1}H NMR (400 MHz, $CDCl_3$) δ 7.25 – 7.12 (m, 4H), 5.54 (brs, 1H), 5.39 (brs, 1H), 3.55 (s, 2H), 2.90 (hept, J = 7.0 Hz, 1H), 1.25 (d, J = 6.9, 0.8 Hz, 6H).

^{13}C NMR (151 MHz, $CDCl_3$) δ 173.9, 148.1, 132.1, 129.3, 127.1, 42.9, 33.7, 23.9.

HRMS (ESI) calculated for $C_{11}H_{15}NOH^+$ $[M+H]^+$ 178.1226, found 178.1231.

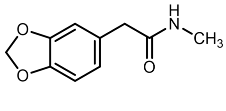

Name: 2-(benzo[d][1,3]dioxol-5-yl)-N-methylacetamide (1ac)

Procedure: The reaction was conducted following the **General Procedure D** on a 5.0 mmol scale. The titled compound was obtained as a white solid in 93% yield (1.8 g, 9.3 mmol).

Isolated Yield: 93%

Physical Property: white solid

^{1}H NMR (400 MHz, $CDCl_3$) δ 6.84 – 6.59 (m, 3H), 5.93 (s, 2H), 5.64 (brs, 1H), 3.45 (s, 2H), 2.74 (d, J = 4.8 Hz, 3H).

^{13}C NMR (151 MHz, $CDCl_3$) δ 171.7, 148.0, 146.8, 128.4, 122.6, 109.7, 108.6, 101.1, 43.2, 26.4.

The data is available in the literature.[18]

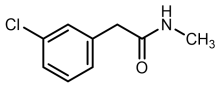

Name: 2-(3-chlorophenyl)-N-methylacetamide (1ad)

Procedure: The reaction was conducted following the **General Procedure D** on a 10.0 mmol scale. The titled compound was obtained as a white solid in 82% yield (1.5 g, 8.2 mmol).

Isolated Yield: 82%

Physical Property: white solid

^{1}H NMR (400 MHz, $CDCl_3$) δ 7.28 – 7.24 (m, 3H), 7.18 – 7.14 (m, 1H), 5.91 (brs, 1H), 3.51 (s, 2H), 2.76 (d, J = 4.8 Hz, 3H).

^{13}C NMR (151 MHz, $CDCl_3$) δ 170.8, 136.9, 134.5, 130.0, 129.4, 127.5, 127.3, 43.0, 26.4.

HRMS (ESI) calculated for $C_9H_{10}ClNOH^+$ $[M+H]^+$ 184.0524, found 184.0530.

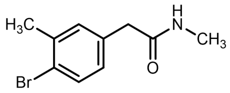

Name: 2-(4-bromo-3-methylphenyl)-N-methylacetamide (1ae)

Procedure: The reaction was conducted following the **General Procedure D** on a 10.0 mmol scale. The titled compound was obtained as a white solid in 55% yield (1.3 g, 5.5 mmol).

Isolated Yield: 55%

Physical Property: white solid

^{1}H NMR (400 MHz, $CDCl_3$) δ 7.47 (dd, J = 8.1, 2.5 Hz, 1H), 7.12 (s, 1H), 6.92 (d, J = 8.1 Hz, 1H), 5.63 (brs, 1H), 3.45 (s, 2H), 2.74 (d, J = 4.8 Hz, 3H), 2.36 (s, 3H).

^{13}C NMR (151 MHz, $CDCl_3$) δ 171.1, 138.4, 134.1, 132.7, 131.8, 128.3, 123.7, 42.9, 26.4, 22.8.

HRMS (ESI) calculated for $C_{10}H_{12}BrNOH^+$ $[M+H]^+$ 242.0175, found 242.0175.

Product:

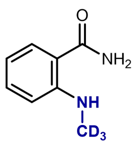

3b

Name: 2-((methyl-d$_3$)amino)benzamide (3b)

Procedure: The reaction was conducted following the **General Procedure B** on a 0.4 mmol scale. The reaction mixture was purified by flash column chromatography with (PE/EA/DCM = 1:1:1, v/v/v) as elute to afford 42.9 mg of **3b** (r.r. > 20:1).

Isolated Yield: 70%

Physical Property: white solid

^{1}H NMR (400 MHz, $CDCl_3$) δ 7.71 (brs, 1H), 7.45 – 7.30 (m, 2H), 6.68 (d, J = 8.4 Hz, 1H), 6.58 (t, J = 7.5 Hz, 1H), 5.73 (brs, 2H).

^{13}C NMR (151 MHz, $CDCl_3$) δ 172.2, 151.2, 133.6, 128.2, 114.2, 112.8, 111.3, 28.8 (hept, $J_{C\text{-}D}$ = 22.3 Hz).

HRMS (ESI) calculated for $C_8H_7D_3N_2OH^+$ $[M+H]^+$ 154.1054, found 154.1053.

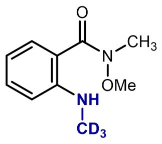

3c

Name: N-methoxy-N-methyl-2-((methyl-d$_3$)amino)benzamide (3c)

Procedure: The reaction was conducted following the **General Procedure B** on a 0.4 mmol scale. The reaction mixture was purified by flash column chromatography with (PE/EA/DCM = 1:1:1, v/v/v) as elute to afford 48.9 mg of **3c** (r.r. > 20:1).

Isolated Yield: 62%

Physical Property: dark oil

Procedure for gram-scale experiment: The reaction was conducted following the General Procedure B in a 5.0 mmol scale with HFIP (50 mL) as solvent and with [N-2] of 1.5 equiv. as aminating reagent. The reaction was stirred at 30 °C for 11 hours. The reaction mixture was purified by flash column chromatography with PE/EA (1:1) as elute to afford 0.56 g of **3c** (57% yield, r.r. > 20:1).

^{1}H NMR (400 MHz, $CDCl_3$) δ 7.35 (d, J = 7.7 Hz, 1H), 7.29 (t, J = 7.8 Hz, 1H), 6.70 – 6.57 (m, 2H), 5.72 (brs, 1H), 3.59 (s, 3H), 3.33 (s, 3H).

^{13}C NMR (151 MHz, $CDCl_3$) δ 170.5, 148.9, 131.8, 129.1, 116.2, 114.8, 110.7, 60.9, 34.5, 29.2 (hept, $J_{C\text{-}D}$ = 20.7 Hz).

HRMS (ESI) calculated for $C_{10}H_{11}D_3N_2O_2Na^+$ $[M+Na]^+$ 220.1136, found 220.1141.

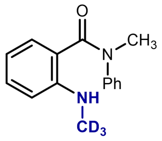

3d

Name: N-methyl-2-((methyl-d$_3$)amino)-N-phenylbenzamide (3d)

Procedure: The reaction was conducted following the **General Procedure B** on a 0.4 mmol scale. The reaction mixture was purified by flash column chromatography with (PE/EA = 2:1, v/v/v) as elute to afford 71.0 mg of **3d** (r.r. > 20:1).

Isolated Yield: 73%

Physical Property: green oil

^{1}H NMR (400 MHz, $CDCl_3$) δ 7.21 (t, J = 7.7 Hz, 2H), 7.15 – 6.99 (m, 4H), 6.73 (dd, J = 7.7, 1.6 Hz, 1H), 6.64–6.56 (m, 1H), 6.26 (td, J = 7.5, 1.1 Hz, 1H), 5.74 (brs, 1H), 3.47 (s, 3H).

^{13}C NMR (151 MHz, $CDCl_3$) δ 171.5, 149.1, 145.2, 130.8, 129.8, 129.0, 126.2, 126.1, 118.8, 114.7, 110.4, 37.9, 29.3 (hept, $J_{C\text{-}D}$ = 20.7 Hz).

HRMS (ESI) calculated for $C_{15}H_{13}D_3N_2ONa^+$ $[M+Na]^+$ 266.1343, found 266.1347.

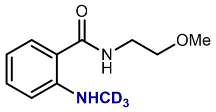

3e

Name: N-(2-methoxyethyl)-2-((methyl-d$_3$)amino)benzamide (3e)

Procedure: The reaction was conducted following the **General Procedure B** on a 0.2 mmol scale. The reaction mixture was purified by flash column chromatography with (PE/EA = 2:1, v/v) as elute to afford 28.7 mg of **3e** (r.r. > 20:1).

Isolated Yield: 68%

Physical Property: colorless oil

^{1}H NMR (400 MHz, $CDCl_3$) δ 7.45 (brs, 1H), 7.38 – 7.27 (m, 2H), 6.65 (d, J = 8.4 Hz, 1H), 6.58 (t, J = 7.6 Hz, 1H), 6.44 (brs, 1H), 3.62 – 3.56 (m, 2H), 3.56 – 3.50 (m, 2H), 3.37 (s, 3H).

^{13}C NMR (151 MHz, $CDCl_3$) δ 169.8, 150.5, 132.8, 127.3, 115.0, 114.4, 111.0, 71.2, 58.8, 39.2, 28.9 (hept, $J_{C\text{-}D}$ = 20.4 Hz).

HRMS (ESI) calculated for $C_{11}H_{13}D_3N_2O_2Na^+$ $[M+Na]^+$ 234.1292, found 234.1302.

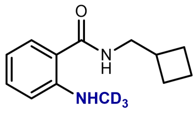

3f

Name: N-(cyclobutylmethyl)-2-((methyl-d$_3$)amino)benzamide (3f)

Procedure: The reaction was conducted following the **General Procedure B** on a 0.2 mmol scale. The reaction mixture was purified by flash column chromatography with (PE/EA = 2:1, v/v) as elute to afford 33.2 mg of **3f** (r.r. > 20:1).

Isolated Yield: 75%

Physical Property: white solid

^{1}H NMR (400 MHz, $CDCl_3$) δ 7.39 (brs, 1H), 7.32 – 7.21 (m, 2H), 6.65 (d, J = 8.3 Hz, 1H), 6.57 (t, J = 7.5 Hz, 1H), 5.97 (brs, 1H), 3.42 (t, J = 6.6 Hz, 2H), 2.56 (hept, J = 7.6 Hz, 1H), 2.18 – 2.02 (m, 2H), 2.00 – 1.84 (m, 2H), 1.82 – 1.66 (m, 2H).

^{13}C NMR (151 MHz, $CDCl_3$) δ 169.9, 150.5, 132.7, 127.0, 115.5, 114.4, 111.1, 44.8, 35.1, 29.0 (hept, J_{C-D} = 20.2 Hz). 25.7, 18.4.

HRMS (ESI) calculated for $C_{13}H_{15}D_3N_2ONa^+$ $[M+Na]^+$ 244.1500, found 244.1506.

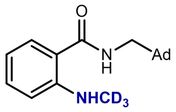

3g

Name: N-(((3r,5r,7r)-adamantan-1-yl)methyl)-2-((methyl-d$_3$)amino)benzamide (3g)

Procedure: The reaction was conducted following the **General Procedure B** on a 0.4 mmol scale. The reaction mixture was purified by flash column chromatography with (PE/EA = 2:1, v/v) as elute to afford 81.9 mg of **3g** (r.r. > 20:1).

Isolated Yield: 68%

Physical Property: white solid

^{1}H NMR (400 MHz, $CDCl_3$) δ 7.41 (brs, 1H), 7.52 – 7.30 (m, 2H), 6.67 (d, J = 8.3 Hz, 1H), 6.60 (t, J = 7.5 Hz, 1H), 6.08 (brs, 1H), 3.11 (d, J = 6.3 Hz, 2H), 2.06 – 1.91 (m, 3H), 1.78 – 1.69 (m, 3H), 1.69 – 1.60 (m, 3H), 1.59 – 1.51 (m, 6H).

^{13}C NMR (151 MHz, $CDCl_3$) δ 169.9, 150.4, 132.6, 126.9, 115.6, 114.3, 111.0, 50.8, 40.3, 36.9, 34.0, 28.9 (hept, J_{C-D} = 20.6 Hz), 28.2.

HRMS (ESI) calculated for $C_{19}H_{23}D_3N_2ONH^+$ $[M+H]^+$ 302.2306, found 302.2307.

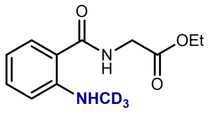

3h

Name: ethyl (2-((methyl-d$_3$)amino)benzoyl)glycinate (3h)

Procedure: The reaction was conducted following the **General Procedure B** on a 0.2 mmol scale. The reaction mixture was purified by flash column chromatography with (PE/EA = 2:1, v/v) as elute to afford 45.4 mg of **3h** (r.r. > 20:1).

Isolated Yield: 95%

Physical Property: green solid

^{1}H NMR (400 MHz, $CDCl_3$) δ 7.62 – 7.29 (m, 3H), 6.78 – 36.50 (m, 3H), 4.24 (q, J = 7.1 Hz, 2H), 4.15 (d, J = 5.1 Hz, 2H), 1.30 (t, J = 7.1 Hz, 3H).

^{13}C NMR (151 MHz, $CDCl_3$) δ 170.1, 169.7, 150.6, 133.1, 127.5, 114.4, 114.0, 111.1, 61.5, 41.5, 28.8 (hept, $J_{C\text{-}D}$ = 20.6 Hz), 14.1.

HRMS (ESI) calculated for $C_{12}H_{13}D_3N_2O_3Na^+$ $[M+Na]^+$ 262.1241, found 262.1242.

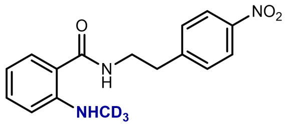

3i

Name: 2-((methyl-d$_3$)amino)-N-(4-nitrophenethyl)benzamide (3i)

Procedure: The reaction was conducted following the **General Procedure B** on a 0.2 mmol scale. The reaction mixture was purified by flash column chromatography with (PE/EA = 2:1, v/v) as elute to afford 45.9 mg of **3i** (r.r. > 20:1).

Isolated Yield: 76%

Physical Property: yellow solid

Procedure for gram-scale experiment: The reaction was conducted following the General Procedure B in a 5.0 mmol scale with HFIP (50 mL) as solvent and with [N-2] of 1.5 equiv. as aminating reagent. The reaction was stirred at 30 °C for 11 hours. The reaction mixture was purified by flash column chromatography with PE/EA (5:1 to 2:1) as elute to afford 1.06 g of **3i** (70% yield, r.r. > 20:1).

^{1}H NMR (400 MHz, $CDCl_3$) δ 8.17 (d, J = 8.3 Hz, 2H), 7.40 (d, J = 8.3 Hz, 2H), 7.36 – 7.28 (m, 1H), 7.19 (d, J = 7.9 Hz, 1H), 6.67 (d, J = 8.4 Hz, 1H), 6.55 (t, J = 7.5 Hz, 1H), 6.11 (s, 1H), 3.69 (q, J = 6.7 Hz, 2H), 3.03 (t, J = 7.0 Hz, 2H).

^{13}C NMR (151 MHz, $CDCl_3$) δ 170.0, 150.6, 147.0, 146.8, 133.1, 129.7, 127.0, 123.9, 114.7, 114.5, 111.3, 40.5, 35.8, 28.9 (hept, $J_{C\text{-}D}$ = 20.6 Hz).

HRMS (ESI) calculated for $C_{16}H_{14}D_3N_3O_3NH^+$ $[M+H]^+$ 303.1531, found 303.1540.

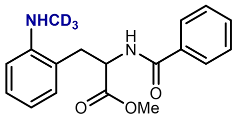

3j

Name: methyl 2-benzamido-3-(2-((methyl-d$_3$)amino)phenyl)propanoate (3j)

Procedure: The reaction was conducted following the **General Procedure B** on a 0.2 mmol scale. The reaction mixture was purified by flash column chromatography with (PE/EA = 2:1, v/v) as elute to afford 44.1 mg of **3j** (r.r. > 20:1).

Isolated Yield: 70%

Physical Property: yellow oil

^{1}H NMR (400 MHz, $CDCl_3$) δ 7.42 (brs, 1H), 7.34 – 7.20 (m, 5H), 7.17 – 7.10 (m, 2H), 6.65 (d, J = 8.4 Hz, 1H), 6.55 (t, J = 7.5 Hz, 1H), 6.47 (brs, 1H), 5.00 (dt, J = 7.5, 5.8 Hz, 1H), 3.75 (s, 3H), 3.25 (dd, J = 13.8, 5.7 Hz, 1H), 3.18 (dd, J = 13.8, 5.9 Hz, 1H).

^{13}C NMR (151 MHz, $CDCl_3$) δ 172.1, 169.1, 150.7, 135.9, 133.2, 129.3, 128.6, 127.4, 127.1, 114.5, 114.1, 111.1, 53.3, 52.3, 37.9, 28.9 (hept, $J_{C\text{-}D}$ = 19.2 Hz).

HRMS (ESI) calculated for $C_{18}H_{17}D_3N_2O_3Na^+$ $[M+Na]^+$ 338.1554, found 338.1554.

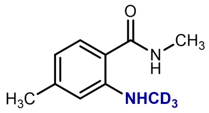

3k

Name: N,4-dimethyl-2-((methyl-d$_3$)amino)benzamide (3k)

Procedure: The reaction was conducted following the **General Procedure B** on a 0.4 mmol scale. The reaction mixture was purified by flash column chromatography with (PE/EA = 10:1 to 5:1, v/v) as elute to afford 65.2 mg of **3k** (r.r. > 20:1).

Isolated Yield: 90%

Physical Property: colorless oil

Procedure for gram-scale experiment: The reaction was conducted following the General Procedure B in a 5.0 mmol scale with HFIP (50 mL) as solvent and with [N-2] of 1.5 equiv. as aminating reagent. The reaction was stirred at 30 °C for 11 hours. The reaction mixture was purified by flash column chromatography with PE/EA (20:1) as elute to afford 0.77 g of **3k** (85% yield, r.r. > 20:1).

^{1}H NMR (400 MHz, $CDCl_3$) δ 7.48 (brs, 1H), 7.19 (d, J = 7.9 Hz, 1H), 6.45 (s, 1H), 6.38 (d, J = 8.0 Hz, 1H), 6.07 (brs, 1H), 2.92 (d, J = 4.9 Hz, 3H), 2.30 (s, 3H).

^{13}C NMR (151 MHz, $CDCl_3$) δ 170.5, 150.5, 143.1, 127.1, 115.5, 112.5, 111.4, 28.8

(hept, J_{C-D} = 20.7 Hz),, 26.4, 21.9.

HRMS (ESI) calculated for $C_{10}H_{11}D_3N_2ONa^+$ $[M+Na]^+$ 204.1187, found 204.1184.

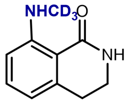

3l

Name: 8-((methyl-d3)amino)-3,4-dihydroisoquinolin-1(2H)-one (3l)

Procedure: The reaction was conducted following the **General Procedure B** on a 0.2 mmol scale. The reaction mixture was purified by flash column chromatography with (PE/EA = 10:1 to 5:1, v/v) as elute to afford 31.9 mg of **3l** (r.r. > 20:1).

Isolated Yield: 89%

Physical Property: white solid

^{1}H NMR (400 MHz, $CDCl_3$) δ 8.36 (brs, 1H), 7.23 (t, J = 7.9 Hz, 1H), 6.52 (d, J = 8.5 Hz, 1H), 6.37 (d, J = 7.2 Hz, 1H), 6.31 (brs, 1H), 3.46 (td, J = 6.6, 2.9 Hz, 2H), 2.88 (t, J = 6.6 Hz, 2H).

^{13}C NMR (101 MHz, $CDCl_3$) δ 169.7, 151.9, 140.6, 133.2, 113.4, 109.1, 109.0, 40.0, 29.5, 28.7 (hept, J_{C-D} = 31.0 Hz).

HRMS (ESI) calculated for $C_{10}H_9D_3N_2OH^+$ $[M+H]^+$ 180.1211, found 180.1209.

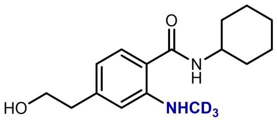

3m

Name: N-cyclohexyl-4-(2-hydroxyethyl)-2-((methyl-d3)amino)benzamide (3m)

Procedure: The reaction was conducted following the **General Procedure B** on a 0.4 mmol scale. The reaction mixture was purified by flash column chromatography with (PE/EA = 5:1, v/v) as elute to afford 72.6 mg of **3m** (r.r. > 20:1).

Isolated Yield: 65%

Physical Property: white solid

^{1}H NMR (400 MHz, $CDCl_3$) δ 7.47 (brs, 1H), 7.35 – 7.13 (m, 1H), 6.49 (s, 1H), 6.43 (d, J = 7.9 Hz, 1H), 5.92 (d, J = 8.1 Hz, 1H), 3.99 – 3.75 (m, 3H), 2.81 (t, J = 6.5 Hz, 2H), 2.10 – 1.84 (m, 2H), 1.86 – 1.57 (m, 4H), 1.49 – 1.33 (m, 2H), 1.31 – 1.07 (m, 3H).

^{13}C NMR (151 MHz, $CDCl_3$) δ 168.9, 150.8, 143.6, 127.4, 115.1, 113.9, 111.4, 63.4, 48.3, 39.6, 33.3, 28.9 (hept, J_{C-D} = 20.8 Hz), 25.6, 24.9.

HRMS (ESI) calculated for $C_{16}H_{21}D_3N_2O_2H^+$ $[M+H]^+$ 280.2099, found 280.2107.

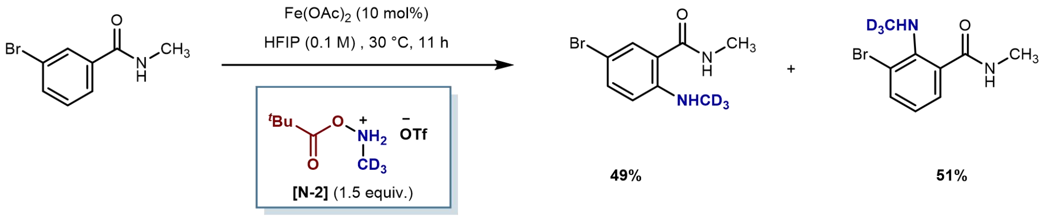

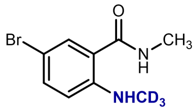

3n

Name: 5-bromo-N-methyl-2-((methyl-d3)amino)benzamide (3n)

Procedure: The reaction was conducted following the **General Procedure B** on a 0.4 mmol scale. The reaction mixture was purified by flash column chromatography with (PE/EA = 10:1 to 1:1, v/v) as elute to afford 48.0 mg of **3n** and 50.0 mg of **3n'**.

Isolated Yield: 49%

Physical Property: white solid

^{1}H NMR (400 MHz, $CDCl_3$) δ 7.30 (brs, 1H), 7.26 (d, J = 2.3 Hz, 1H), 7.23 (dd, J = 8.8, 2.5 Hz, 1H), 6.57 (d, J = 8.8 Hz, 1H), 6.10 (brs, 1H), 2.93 (d, J = 4.8 Hz, 2H).

^{13}C NMR (151 MHz, $CDCl_3$) δ 169.4, 148.9, 132.3, 126.8, 118.9, 116.3, 112.3, 29.0 (hept, $J_{C\text{-}D}$ = 20.6 Hz), 26.5.

HRMS (ESI) calculated for $C_9H_8D_3BrN_2OH^+$ $[M+H]^+$ 246.0316, found 246.0322.

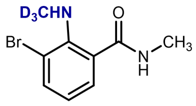

3n'

Name: 3-bromo-N-methyl-2-((methyl-d3)amino)benzamide (3n')

Isolated Yield: 51%

Physical Property: white solid

^{1}H NMR (400 MHz, $CDCl_3$) δ 7.89 (brs, 1H), 7.70 (d, J = 7.9, 1.5 Hz, 1H), 7.39 (dd, J = 8.0, 1.5 Hz, 1H), 6.93 (t, J = 7.8 Hz, 1H), 4.39 (brs, 1H), 3.01 (d, J = 4.9 Hz, 3H).

^{13}C NMR (151 MHz, $CDCl_3$) δ 167.6, 145.0, 131.7, 129.2, 126.8, 126.4, 122.0, 35.2 (hept, $J_{C\text{-}D}$ = 21.1 Hz), 26.5.

HRMS (ESI) calculated for $C_9H_8D_3BrN_2OH^+$ $[M+H]^+$ 246.0316, found 246.0328.

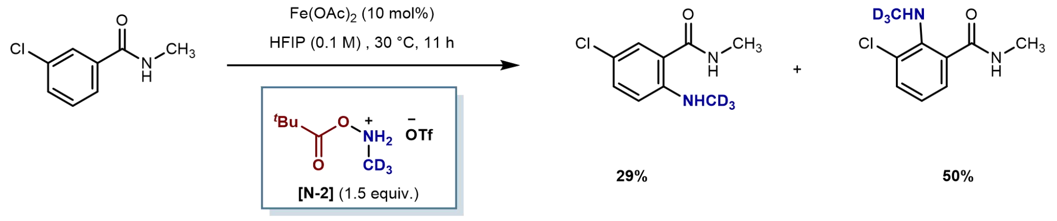

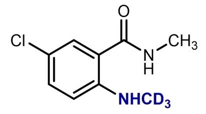

3o

Name: 5-chloro-N-methyl-2-((methyl-d3)amino)benzamide (3o)

Procedure: The reaction was conducted following the **General Procedure B** on a 0.4 mmol scale. The reaction mixture was purified by flash column chromatography with (PE/EA = 10:1 to 1:1, v/v) as elute to afford 23.3 mg of **3o** and 40.2 mg of **3o'**.

Isolated Yield: 29%

Physical Property: white solid

^{1}H NMR (400 MHz, $CDCl_3$) δ 7.53 – 7.29 (m, 2H), 6.52 (d, J = 8.9, 1.9 Hz, 1H), 6.09 (brs, 1H), 2.93 (d, 3H).

^{13}C NMR (151 MHz, $CDCl_3$) δ 169.3, 149.2, 135.1, 129.6, 116.9, 112.8, 105.6, 28.9 (hept, $J_{C\text{-}D}$ = 20.7 Hz),, 26.5.

HRMS (ESI) calculated for $C_9H_8D_3ClN_2OH^+$ $[M+H]^+$ 202.0821, found 202.0820.

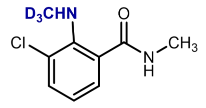

3o'

Name: 3-chloro-N-methyl-2-((methyl-d3)amino)benzamide (3o')

Isolated Yield: 50%

Physical Property: white solid

^{1}H NMR (400 MHz, $CDCl_3$) δ 8.06 (brs, 1H), 7.76 (dd, J = 7.8, 1.6 Hz, 1H), 7.55 (dd, J = 7.9, 1.6 Hz, 1H), 6.87 (t, J = 7.8 Hz, 1H), 4.19 (brs, 1H), 2.99 (d, J = 4.9 Hz, 3H).

^{13}C NMR (151 MHz, $CDCl_3$) δ 167.3, 145.8, 134.9, 130.2, 127.2, 122.9, 117.7, 35.4 (hept, $J_{C\text{-}D}$ = 20.9 Hz), 26.5.

HRMS (ESI) calculated for $C_9H_8D_3ClN_2OH^+$ $[M+H]^+$ 202.0821, found 202.0816.

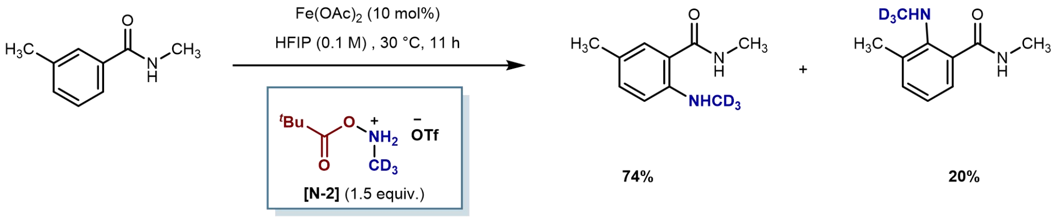

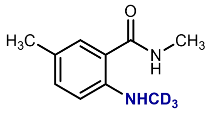

3p

Name: N,5-dimethyl-2-((methyl-d$_3$)amino)benzamide (3p)

Procedure: The reaction was conducted following the **General Procedure B** on a 0.4 mmol scale. The reaction mixture was purified by flash column chromatography with (PE/EA = 10:1 to 5:1, v/v) as elute to afford 53.6 mg of **3p** and 14.5 mg of **3p'**.

Isolated Yield: 74%

Physical Property: white solid

^{1}**H NMR** (400 MHz, $CDCl_3$) δ 7.21 – 7.03 (m, 3H), 6.58 (d, J = 8.2 Hz, 1H), 6.08 (brs, 1H), 2.93 (d, J = 4.8 Hz, 3H), 2.22 (s, 3H).

^{13}**C NMR** (151 MHz, $CDCl_3$) δ 170.5, 148.3, 133.4, 127.4, 123.4, 115.4, 111.2, 29.1 (hept, $J_{C\text{-}D}$ = 20.7 Hz), 26.4, 20.2.

HRMS (ESI) calculated for $C_{10}H_{11}D_3N_2ONa^+$ $[M+Na]^+$ 204.1187, found 204.1192.

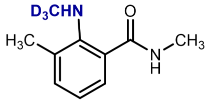

3p'

Name: N,3-dimethyl-2-((methyl-d$_3$)amino)benzamide (3p')

Isolated Yield: 20%

Physical Property: brown solid

^{1}**H NMR** (400 MHz, $CDCl_3$) δ 7.66 (brs, 1H), 7.57 (dd, J = 7.9, 1.7 Hz, 1H), 7.19 (dd, J = 7.4, 1.7 Hz, 1H), 6.89 (t, J = 7.6 Hz, 1H), 4.48 (brs, 1H), 2.99 (d, J = 4.8 Hz, 3H), 2.31 (s, 3H).

^{13}**C NMR** (151 MHz, $CDCl_3$) δ 169.2, 148.1, 133.6, 129.6, 127.5, 125.2, 121.3, 35.2 (hept, $J_{C\text{-}D}$ = 20.8 Hz), 26.5, 18.4.

HRMS (ESI) calculated for $C_{10}H_{11}D_3N_2OH^+$ $[M+H]^+$ 182.1367, found 182.1366.

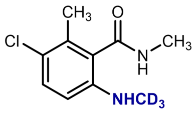

3q

Name: 3-chloro-N,2-dimethyl-6-((methyl-d3)amino)benzamide (3q)

Procedure: The reaction was conducted following the **General Procedure B** on a 0.3 mmol scale. The reaction mixture was purified by flash column chromatography with (PE/EA = 5:1 to 1:1, v/v) as elute to afford 24.5 mg of **3q** (r.r. > 20:1).

Isolated Yield: 38%

Physical Property: orange solid

^{1}H NMR (400 MHz, $CDCl_3$) δ 7.20 (d, J = 8.8 Hz, 1H), 6.41 (d, J = 8.7 Hz, 1H), 5.81 (brs, 1H), 4.54 (brs, 1H), 2.98 (d, J = 5.0 Hz, 3H), 2.29 (s, 3H).

^{13}C NMR (151 MHz, $CDCl_3$) δ 169.6, 145.5, 132.3, 130.5, 124.1, 122.6, 109.2, 29.7 (hept, $J_{C\text{-}D}$ = 20.6 Hz), 26.5, 17.8.

HRMS (ESI) calculated for $C_{10}H_{10}D_3ClN_2ONa^+$ $[M+Na]^+$ 238.0797, found 238.0800.

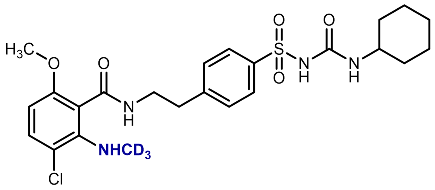

3r

Name:
3-chloro-N-(4-(N-(cyclohexylcarbamoyl)sulfamoyl)phenethyl)-6-methoxy-2-((methyl-d3)amino)benzamide (3r)

Procedure: The reaction was conducted following the **General Procedure B** on a 0.2 mmol scale. The reaction mixture was purified by flash column chromatography with (DCM:MeOH = 10:1 to 2:1, v/v) as elute to afford 84.0 mg of **3r** (r.r. > 20:1).

Isolated Yield: 80%

Physical Property: white solid

^{1}H NMR (400 MHz, $CDCl_3$) δ 7.83 (d, J = 8.0 Hz, 2H), 7.42 (d, J = 8.0 Hz, 2H), 7.19 (d, J = 8.7 Hz, 1H), 6.52 – 6.29 (m, 2H), 6.24 (d, J = 8.8 Hz, 1H), 3.80 – 3.63 (m, 5H), 3.63 – 3.46 (m, 1H), 3.03 (t, J = 7.0 Hz, 2H), 1.89 – 1.71 (m, 2H), 1.73 – 1.48 (m, 3H), 1.38 – 1.23 (m, 2H), 1.23 – 1.03 (m, 3H).

^{13}C NMR (151 MHz, DMSO-d_6) δ 166.1, 156.4, 150.6, 145.4, 142.8, 138.1, 129.2, 129.1, 127.1, 113.7, 112.5, 100.0, 55.6, 48.1, 34.2, 32.3, 30.2 (hept, $J_{C\text{-}D}$ = 21.1 Hz), 25.0, 24.2.

HRMS (ESI) calculated for $C_{24}H_{28}D_3ClN_4O_5Na^+$ $[M+Na]^+$ 526.1965, found

526.1970.

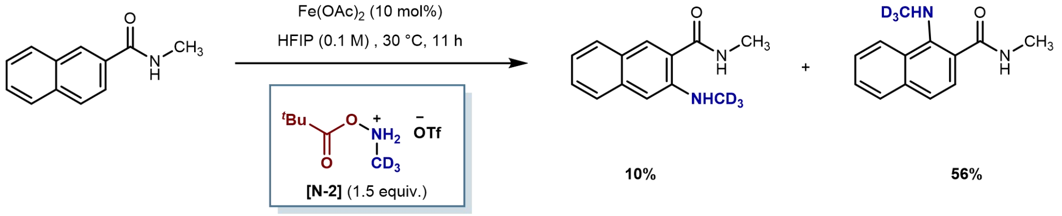

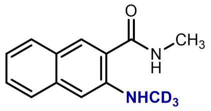

3s

Name: N-methyl-3-((methyl-d$_3$)amino)-2-naphthamide (3s)

Procedure: The reaction was conducted following the **General Procedure B** on a 0.4 mmol scale. The reaction mixture was purified by flash column chromatography with (PE:EA = 10:1 to 1:1, v/v) as elute to afford 8.7 mg of **3s** and 48.6 mg of **3s'**.

Isolated Yield: 10%

Physical Property: yellow solid

^{1}H NMR (400 MHz, $CDCl_3$) δ 7.79 (s, 1H), 7.66 – 7.56 (m, 2H), 7.43 – 7.36 (m, 1H), 7.21 – 7.12 (m, 1H),, 6.79 (s, 1H), 6.71 (brs, 1H), 6.24 (brs, 1H), 3.01 (d, J = 4.9 Hz, 3H).

^{13}C NMR (151 MHz, $CDCl_3$) δ 170.3, 146.4, 136.6, 128.2, 127.9, 127.8, 125.6, 125.2, 122.1, 121.5, 104.3, 29.4 (hept, $J_{C\text{-}D}$ = 21.0 Hz), 26.7.

HRMS (ESI) calculated for $C_{13}H_{11}D_3N_2ONa^+$ $[M+Na]^+$ 240.1187, found 240.1195.

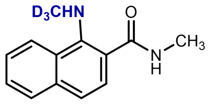

3s'

Name: N-methyl-1-((methyl-d$_3$)amino)-2-naphthamide (3s')

Isolated Yield: 56%

Physical Property: brown solid

^{1}H NMR (400 MHz, $CDCl_3$) δ 8.21 – 8.13 (m, 1H), 7.83 – 7.74 (m, 1H), 7.63 (d, J = 8.6 Hz, 1H), 7.55 – 7.45 (m, 2H), 7.40 (d, J = 8.6 Hz, 1H), 7.28 (brs, 1H), 6.16 (brs, 1H), 3.04 (d, J = 4.8 Hz, 3H).

^{13}C NMR (151 MHz, $CDCl_3$) δ 169.7, 149.0, 136.0, 128.4, 127.7, 127.3, 125.4, 124.7, 124.4, 121.1, 118.9, 29.4 (hept, $J_{C\text{-}D}$ = 21.1 Hz), 26.6.

HRMS (ESI) calculated for $C_{13}H_{11}D_3N_2ONa^+$ $[M+Na]^+$ 240.1187, found 240.1192.

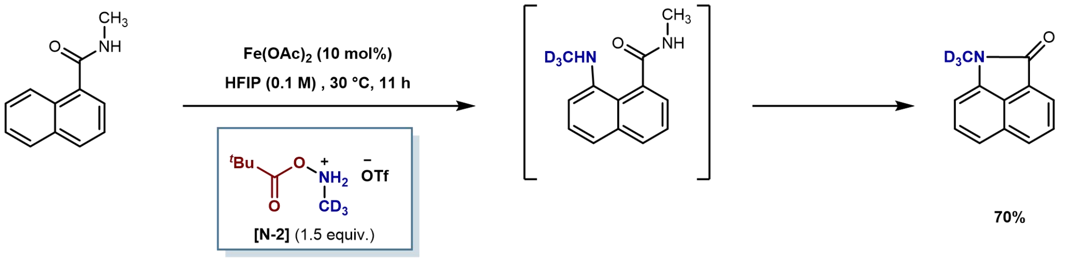

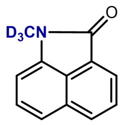

3t

Name: 1-(methyl-d$_3$)benzo[cd]indol-2(1H)-one (3t)

Procedure: The reaction was conducted following the **General Procedure B** on a 0.4 mmol scale. The reaction mixture was purified by flash column chromatography with (PE:EA = 10:1 to 1:1, v/v) as elute to afford 52.1 mg of **3t** (r.r. > 20:1).

Isolated Yield: 70%

Physical Property: green solid

^{1}H NMR (400 MHz, $CDCl_3$) δ 8.05 (d, J = 7.0 Hz, 1H), 7.99 (d, J = 8.1 Hz, 1H), 7.75–7.63 (m, 1H), 7.52 (d, J = 8.4 Hz, 1H), 7.46 (dd, J = 8.5, 6.9 Hz, 1H), 6.88 (d, J = 6.9 Hz, 1H).

^{13}C NMR (151 MHz, $CDCl_3$) δ 168.2, 140.0, 130.6, 128.9, 128.6, 128.4, 126.7, 125.0, 124.1, 120.2, 104.6, 25.6 (hept, $J_{C\text{-}D}$ = 21.2 Hz).

HRMS (ESI) calculated for $C_{12}H_6D_3NOH^+$ $[M+H]^+$ 187.0952, found 187.0945.

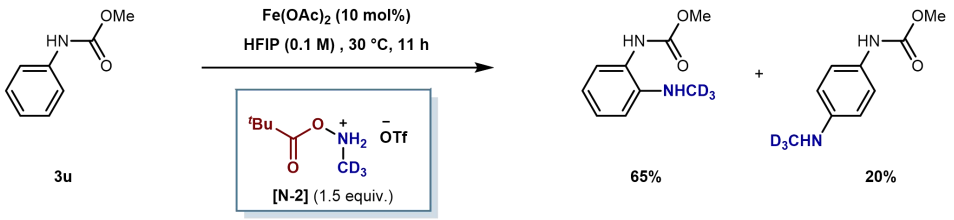

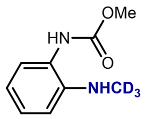

3u

Name: methyl (2-((methyl-d$_3$)amino)phenyl)carbamate (3u)

Procedure: The reaction was conducted following the **General Procedure B** on a 0.4 mmol scale. The reaction mixture was purified by flash column chromatography with

(PE:EA = 1:1, v/v) as elute to afford 47.6 mg of **3u** and 14.6 mg of **3u'**.

Isolated Yield: 65%

Physical Property: cyan solid

^{1}H NMR (400 MHz, $CDCl_3$) δ 7.25 – 7.21 (m, 1H), 7.20 – 7.08 (m, 1H), 6.81 – 6.66 (m, 2H), 6.22 (brs, 1H), 3.83 (brs, 1H), 3.76 (s, 3H).

^{13}C NMR (151 MHz, CD_3CN) δ 156.8, 146.1, 128.5, 127.8, 123.9, 117.0, 111.5, 52.8, 29.7 (hept, $J_{C\text{-}D}$ = 19.7 Hz).

HRMS (ESI) calculated for $C_9H_9D_3N_2O_2H^+$ $[M+H]^+$ 183.1165, found 183.1164.

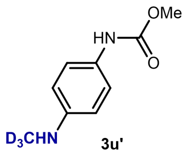

3u'

Name: methyl (2-methyl-4-((methyl-d$_3$)amino)phenyl)carbamate (3u')

Isolated Yield: 20%

Physical Property: white solid

^{1}H NMR (400 MHz, CD_3CN) δ 7.23 – 7.02 (m, 2H), 6.83 (brs, 1H), 6.70 – 6.56 (m, 2H), 4.40 (brs, 1H), 3.68 (s, 3H).

^{13}C NMR (151 MHz, CD_3CN) δ 155.8, 147.7, 129.1, 122.2, 113.1, 52.4, 30.2 (hept, J = 20.6 Hz).

HRMS (ESI) calculated for $C_9H_9D_3N_2O_2H^+$ $[M+H]^+$ 183.1165, found 183.1160.

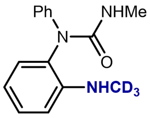

3v

Name: 3-methyl-1-(2-((methyl-d$_3$)amino)phenyl)-1-phenylurea (3v)

Procedure: The reaction was conducted following the **General Procedure B** on a 0.4 mmol scale. The reaction mixture was purified by flash column chromatography with (PE:EA = 1:1, v/v) as elute to afford 90.9 mg of **3v** (r.r. > 20:1).

Isolated Yield: 88%

Physical Property: white solid

^{1}H NMR (400 MHz, $CDCl_3$) δ 7.33 – 7.20 (m, 5H), 7.13 (dd, J = 8.0, 1.6 Hz, 1H), 7.09 – 7.02 (m, 1H), 6.76 – 6.68 (m, 2H), 4.73 – 4.52 (m, 1H), 4.21 (brs, 1H), 2.78 (d, J = 4.8 Hz, 3H).

^{13}C NMR (151 MHz, $CDCl_3$) δ 156.6, 146.3, 142.2, 130.2, 129.8, 128.6, 125.9, 124.2,

123.7, 117.1, 111.3, 29.3 (hept, $J_{C\text{-}D}$ = 20.6 Hz), 27.4.

HRMS (ESI) calculated for $C_{15}H_{14}D_3N_3ONa^+$ $[M+Na]^+$ 281.1452, found 281.1457.

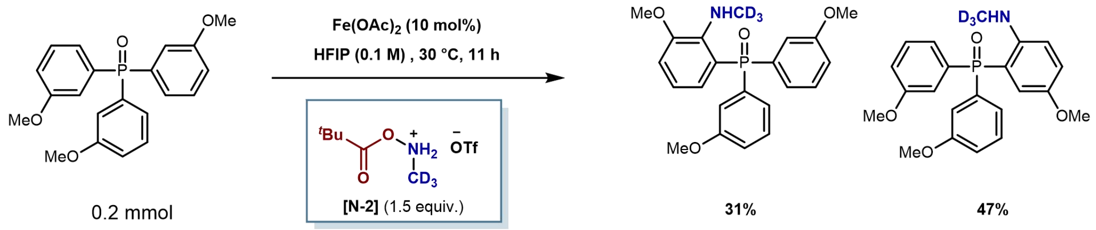

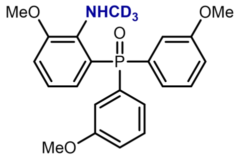

3w

Name: (3-methoxy-2-((methyl-d3)amino)phenyl)bis(3-methoxyphenyl)phosphine oxide (3w)

Procedure: The reaction was conducted following the **General Procedure B** on a 0.2 mmol scale. The reaction mixture was purified by flash column chromatography with (PE:EA:DCM = 2:1:1, v/v/v) as elute to afford 24.8 mg of **3w** and 37.6 mg of **3w'**.

Isolated Yield: 31%

Physical Property: yellow oil

^{1}H NMR (400 MHz, $CDCl_3$) δ 7.34 (td, J = 7.9, 3.8 Hz, 2H), 7.25 (ddd, J = 13.3, 2.7, 1.3 Hz, 2H), 7.14 – 7.02 (m, 4H), 6.96 (d, J = 7.3 Hz, 1H), 6.68 (td, J = 7.9, 3.6 Hz, 1H), 6.44 (ddd, J = 13.9, 7.8, 1.4 Hz, 1H), 5.89 (s, 1H), 3.83 (s, 3H), 3.79 (s, 6H).

^{13}C NMR (151 MHz, $CDCl_3$) δ 159.6, 159.5, 151.72, 151.65, 145.83, 145.80, 134.4, 133.7, 129.6, 129.5, 125.5, 125.4, 124.35, 124.29, 118.7, 118.57, 118.53, 118.17, 118.16, 117.8, 116.7, 116.6, 116.2, 55.9, 55.4, 33.3 (hept, $J_{C\text{-}D}$ = 20.8 Hz).

^{31}P NMR (162 MHz, $CDCl_3$) δ 35.2 ppm.

HRMS (ESI) calculated for $C_{22}H_{21}D_3NO_4PH+$ [M+H]+ 401.1704, found 401.1696.

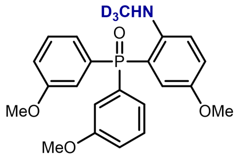

3w'

Name: (5-methoxy-2-((methyl-d3)amino)phenyl)bis(3-methoxyphenyl)phosphine oxide (3w')

Isolated Yield: 47%

Physical Property: colorless oil

^{1}H NMR (400 MHz, $CDCl_3$) δ 7.35 (td, J = 7.9, 3.9 Hz, 2H), 7.26 – 7.19 (m, 2H), 7.12 – 7.03 (m, 4H), 7.00 (dd, J = 9.0, 2.2 Hz, 1H), 6.62 (dd, J = 9.0, 5.8 Hz, 1H), 6.38 (dd, J = 15.8, 3.0 Hz, 1H), 6.34 (s, 1H), 3.79 (s, 6H), 3.59 (s, 6H).

^{13}C NMR (151 MHz, $CDCl_3$) δ 159.6, 159.5, 149.6, 149.5, 149.10, 149.08, 133.8, 133.1, 129.7, 129.6, 124.4, 124.3, 119.6, 119.5, 119.4, 118.28, 118.27, 116.73, 116.65, 112.1, 111.8, 111.7, 111.4, 55.9, 55.4, 29.9 (hept, $J_{C\text{-}D}$ = 29.0 Hz).

^{31}P NMR (162 MHz, $CDCl_3$) δ 36.3 ppm.

HRMS (ESI) calculated for $C_{22}H_{21}D_3NO_4PH^+$ [M+H]+ 401.1704, found 401.1701.

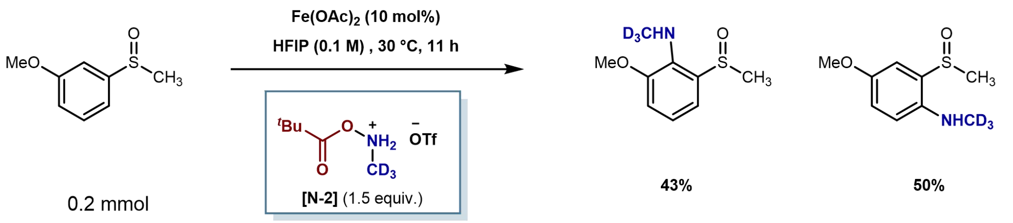

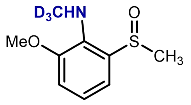

3x

Name: 2-methoxy-N-(methyl-d$_3$)-6-(methylsulfinyl)aniline (3x)

Procedure: The reaction was conducted following the **General Procedure B** on a 0.2 mmol scale. The reaction mixture was purified by flash column chromatography with (PE:EA:DCM = 1:1:1, v/v/v) as elute to afford 17.4 mg of **3x** and 20.2 mg of **3x'**.

Isolated Yield: 43%

Physical Property: white soild

^{1}H NMR (400 MHz, $CDCl_3$) δ 7.39 (d, J = 8.0 Hz, 1H), 6.99 (t, J = 8.0 Hz, 1H), 6.90 (d, J = 8.0 Hz, 1H), 4.49 (brs, 1H), 3.85 (s, 3H), 2.74 (s, 3H).

^{13}C NMR (151 MHz, $CDCl_3$) δ 150.0, 137.3, 132.9, 120.5, 116.1, 112.6, 56.0, 40.4, 34.5 (hept, $J_{C\text{-}D}$= 20.4 Hz).

HRMS (ESI) calculated for $C_9H_{10}D_3NO_2SH^+$ [M+H]+ 203.0928, found 203.0925.

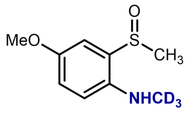

3x'

Name: 4-methoxy-N-(methyl-d$_3$)-2-(methylsulfinyl)aniline (3x')

Isolated Yield: 50%

Physical Property: white soild

^{1}H NMR (400 MHz, $CDCl_3$) δ 6.94 (d, J = 8.8 Hz, 1H), 6.86 (s, 1H), 6.66 (d, J = 9.0 Hz, 1H), 5.63 (brs, 1H), 3.74 (s, 3H), 2.88 (s, 3H).

^{13}C NMR (151 MHz, $CDCl_3$) δ 150.8, 143.6, 123.9, 119.0, 112.7, 111.3, 55.9, 38.0, 29.5 (hept, $J_{C\text{-}D}$ = 20.7 Hz).

HRMS (ESI) calculated for $C_9H_{10}D_3NO_2SH$+ [M+H]+ 203.0928, found 203.0929.

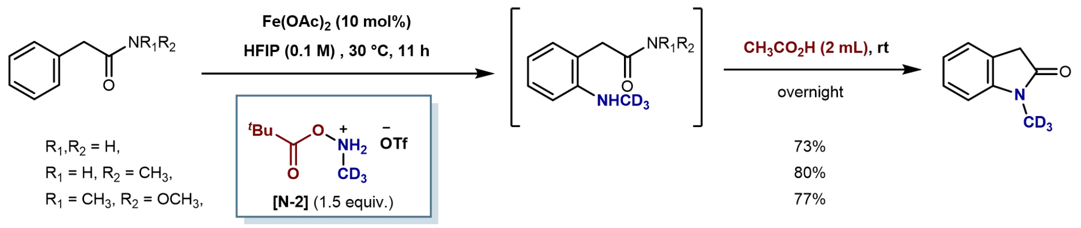

General Procedure B':

Step-1: The reaction was conducted following the **General Procedure B**.

Step-2: After the amination reaction, the solvent was removed under reduced pressure. The resulting mixture was re-dissolved into acetic acid (2.0 mL) and stirred overnight at room temperature. The reaction mixture was then purified by flash column chromatography to afford the titled product.

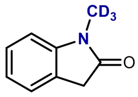

3y

Name: 1-(methyl-d$_3$)indolin-2-one (3y)

Procedure: The reaction was conducted following the **General Procedure B'** on a 0.4 mmol scale. The reaction mixture was purified by flash column chromatography with (PE:EA:DCM = 1:1:1, v/v/v) as elute to afford 43.8 mg of **3y** (r.r. > 20:1).

Isolated Yield: 73%

Physical Property: white solid

^{1}H NMR (400 MHz, $CDCl_3$) δ 7.31 – 7.22 (m, 2H), 7.04 (t, J = 7.5, 1.0 Hz, 1H), 6.82 (d, J = 7.8 Hz, 1H), 3.52 (s, 2H).

^{13}C NMR (151 MHz, $CDCl_3$) δ 175.0, 145.1, 127.8, 124.4, 124.2, 122.2, 108.0, 35.7, 25.4 (hept, $J_{C\text{-}D}$ = 21.2 Hz).

HRMS (ESI) calculated for $C_9H_6D_3NOH^+$ $[M+H]^+$ 151.0945, found 151.0951.

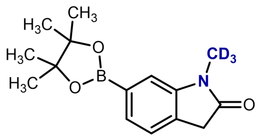

3z

Name: 1-(methyl-d3)-6-(4,4,5,5-tetramethyl-1,3,2-dioxaborolan-2-yl)indolin-2-one (3z)

Procedure: The reaction was conducted following the **General Procedure B'** on a 0.4 mmol scale. The reaction mixture was purified by flash column chromatography with (PE:EA = 10:1, v/v/v) as elute to afford 71.8 mg of **3z** (r.r. > 20:1).

Isolated Yield: 65%

Physical Property: yellow solid

^{1}H NMR (400 MHz, $CDCl_3$) δ 7.54 (d, J = 7.3 Hz, 1H), 7.30 – 7.20 (m, 2H), 3.54 (s, 2H), 1.38 (s, 12H).

^{13}C NMR (151 MHz, $CDCl_3$) δ 174.8, 144.8, 129.4, 128.0, 123.7, 113.4, 84.0, 35.9, 25.6 (hept, $J_{C\text{-}D}$ = 21.3 Hz), 24.8.

^{11}B NMR (128 MHz, $CDCl_3$) δ 30.8 ppm.

HRMS (ESI) calculated for $C_{15}H_{17}D_3BNO_3Na^+$ $[M+Na]^+$ 299.1622, found 299.1617.

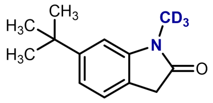

3aa

Name: 6-(*tert*-Butyl)-1-(methyl-d3)indolin-2-one (3aa)

Procedure: The reaction was conducted following the **General Procedure B'** on a 0.4 mmol scale. The reaction mixture was purified by flash column chromatography with (PE:EA = 10:1, v/v/v) as elute to afford 77.5 mg of **3aa** (r.r. > 20:1).

Isolated Yield: 94%

Physical Property: white solid

^{1}H NMR (400 MHz, $CDCl_3$) δ 7.17 (d, J = 7.8 Hz, 1H), 7.07 (dd, J = 7.8, 1.7 Hz, 1H), 6.83 (d, J = 1.7 Hz, 1H), 3.47 (s, 2H), 1.35 (s, 9H).

^{13}C NMR (151 MHz, $CDCl_3$) δ 175.5, 151.6, 145.1, 123.8, 121.6, 119.1, 105.4, 35.4, 34.9, 31.4, 25.4 (hept, $J_{C\text{-}D}$ = 21.2 Hz).

HRMS (ESI) calculated for $C_{13}H_{14}D_3NOH^+$ $[M+H]^+$ 207.1571, found 207.1577.

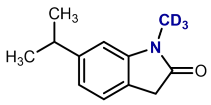

3ab

Name: 6-*isopropyl*-1-(methyl-d$_3$)indolin-2-one (3ab)

Procedure: The reaction was conducted following the **General Procedure B'** on a 0.4 mmol scale. The reaction mixture was purified by flash column chromatography with (PE:EA = 10:1, v/v/v) as elute to afford 53.8 mg of **3ab** (r.r. > 20:1).

Isolated Yield: 70%

Physical Property: orange solid

^{1}H NMR (400 MHz, $CDCl_3$) δ 7.15 (d, J = 7.6 Hz, 1H), 6.90 (dd, J = 7.5, 1.6 Hz, 1H), 6.68 (d, J = 1.6 Hz, 1H), 3.46 (s, 2H), 2.93 (hept, J = 6.9 Hz, 1H), 1.27 (d, J = 7.0 Hz, 6H).

^{13}C NMR (151 MHz, $CDCl_3$) δ 175.4, 149.2, 145.2, 124.0, 121.8, 120.2, 106.3, 35.4, 34.4, 25.4 (hept, $J_{C\text{-}D}$ = 21.1 Hz), 24.1.

HRMS (ESI) calculated for $C_{12}H_{12}D_3NOH^+$ $[M+H]^+$ 193.1415, found 193.1420.

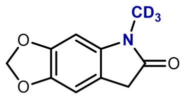

3ac

Name: 5-(methyl-d$_3$)-5,7-dihydro-6H-[1,3]dioxolo[4,5-f]indol-6-one (3ac)

Procedure: The reaction was conducted following the **General Procedure B**' on a 0.4 mmol scale. The reaction mixture was purified by flash column chromatography with (PE:EA = 10:1, v/v/v) as elute to afford 59.8 mg of **3ac** (r.r. > 20:1).

Isolated Yield: 77%

Physical Property: off-white solid

^{1}H NMR (400 MHz, $CDCl_3$) δ 6.74 (s, 1H), 6.40 (s, 1H), 5.90 (s, 2H), 3.41 (s, 2H).

^{13}C NMR (151 MHz, $CDCl_3$) δ 175.3, 147.1, 142.9, 139.3, 115.8, 105.9, 100.9, 92.0, 35.9, 25.6 (hept, $J_{C\text{-}D}$ = 21.3 Hz).

HRMS (ESI) calculated for $C_{10}H_6D_3NOH^+$ $[M+H]^+$ 195.0843, found 195.0849.

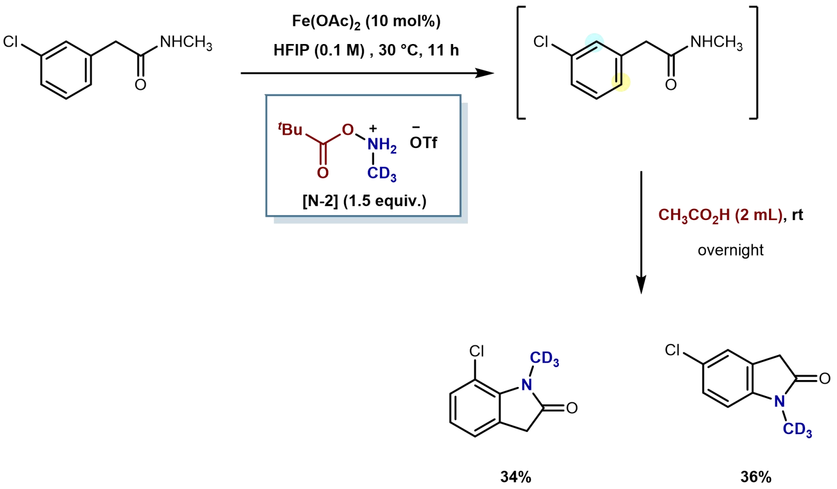

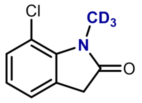

3ad

Name: 7-chloro-1-(methyl-d$_3$)indolin-2-one (3ad)

Procedure: The reaction was conducted following the **General Procedure B**' on a 0.4 mmol scale. The reaction mixture was purified by flash column chromatography with (PE:EA = 5:1, v/v/v) as elute to afford 25.0 mg of **3ad** and 26.5 mg of **3ad'**.

Isolated Yield: 34%

Physical Property: white solid

^{1}H NMR (400 MHz, $CDCl_3$) δ 7.19 (d, J = 7.9 Hz, 1H), 7.11 (d, J = 7.3 Hz, 1H), 6.92 (t, J = 7.8 Hz, 1H), 3.51 (s, 2H).

^{13}C NMR (151 MHz, $CDCl_3$) δ 175.1, 140.9, 130.2, 127.0, 123.0, 122.8, 115.4, 35.6, 28.9 (hept, $J_{C\text{-}D}$ = 21.6 Hz)..

HRMS (ESI) calculated for $C_9H_5D_3ClNOH^+$ $[M+H]^+$ 185.0555, found 185.0561.

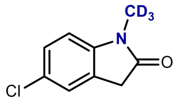

3ad'

Name: 5-chloro-1-(methyl-d$_3$)indolin-2-one (3ad')

Isolated Yield: 36%

Physical Property: pink solid

^{1}H NMR (400 MHz, $CDCl_3$) δ 7.52 – 7.08 (m, 2H), 6.73 (d, J = 8.3 Hz, 1H), 3.50 (s, 2H).

^{13}C NMR (151 MHz, $CDCl_3$) δ 174.4, 143.7, 127.7, 127.6, 126.0, 124.7, 108.8, 35.6, 25.7 (hept, $J_{C\text{-}D}$ = 21.6 Hz).

HRMS (ESI) calculated for $C_9H_5D_3ClNOH^+$ $[M+H]^+$ 185.0555, found 185.0559.

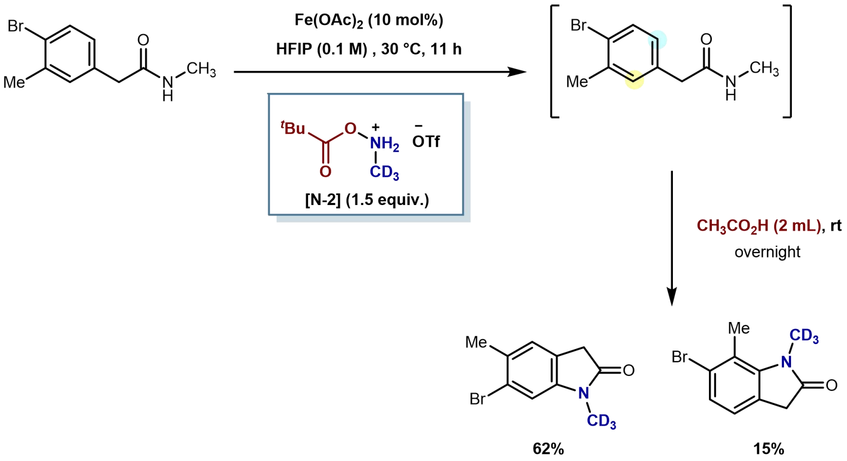

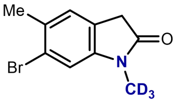

3ae

Name: 5-bromo-4-methyl-1-(methyl-d$_3$)indolin-2-one (3ae)

Procedure: The reaction was conducted following the **General Procedure B**' on a 0.4 mmol scale. The reaction mixture was purified by flash column chromatography with (PE:EA = 5:1, v/v/v) as elute to afford 60.0 mg of **3ae** and 14.5 mg of **3ae'**.

Isolated Yield: 62%

Physical Property: white solid

^{1}H NMR (400 MHz, $CDCl_3$) δ 7.09 (s, 1H), 6.97 (s, 1H), 3.42 (s, 2H), 2.35 (s, 3H).

^{13}C NMR (151 MHz, $CDCl_3$) δ 174.7, 144.2, 131.1, 126.4, 123.7, 123.2, 111.9, 35.3, 25.6 (hept, $J_{C\text{-}D}$ = 21.3 Hz), 22.5.

HRMS (ESI) calculated for $C_{10}H_7D_3BrNOH^+$ $[M+H]^+$ 243.0207, found 243.0209.

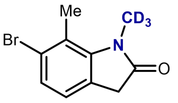

3ae'

Name: 6-bromo-5-methyl-1-(methyl-d$_3$)indolin-2-one (3ae')

Isolated Yield: 15%

Physical Property: white solid

^{1}H NMR (400 MHz, $CDCl_3$) δ 7.25 (d, J = 8.2 Hz, 1H), 6.91 (d, J = 7.9 Hz, 1H), 3.42 (s, 2H), 2.63 (s, 3H).

^{13}C NMR (151 MHz, $CDCl_3$) δ 175.7, 144.2, 126.5, 126.2, 124.2, 122.9, 119.9, 35.2, 29.7 (hept, $J_{C\text{-}D}$ = 21.2 Hz), 18.0.

HRMS (ESI) calculated for $C_{10}H_7D_3BrNOH^+$ $[M+H]^+$ 243.0207, found 243.0210.

9. Product Derivatizations

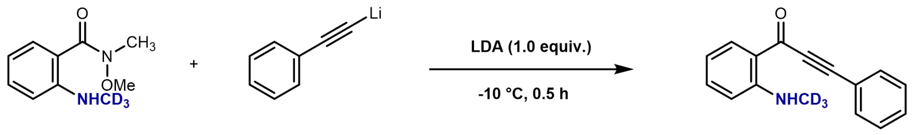

3c 4

Name: 1-(2-((methyl-d$_3$)amino)phenyl)-3-phenylprop-2-yn-1-one (4)

Procedure:

n-BuLi (1.0 mmol, 2.5 M in hexane) was added dropwise at -78 °C to a solution of phenylacetylene (1.0 mmol) in THF (3.0 mL) and the mixture was stirred at -78 °C for 0.5 h.[19]

To a solution of **3c** (78.4 mg, 0.4 mmol) in THF (3.0 mL) was added lithium diisopropylamide solution (2.0 M in THF, 0.2 mL, 0.4 mmol) at -10 °C·and stirred for 10 min. The pre-synthesized lithium phenylacetylide solution was added to the above solution at -10 °C. The reaction mixture was stirred for 0.5 h at -10 °C, which was then quenched with a saturated NH_4Cl solution (2.0 mL) and extracted with EA (3×5 mL). The organic layer was dried over anhydrous Na_2SO_4 and filtered. The reaction mixture was purified by flash column chromatography with PE as elute to afford 66.7 mg of **4**.[20]

Isolated Yield: 70%

Physical Property: orange solid

^{1}H NMR (400 MHz, $CDCl_3$) δ 8.76 (brs, 1H), 8.40 – 8.10 (m, 1H), 7.77 – 7.61 (m, 2H), 7.57 – 7.31 (m, 4H), 6.78 – 6.60 (m, 2H).

^{13}C NMR (151 MHz, $CDCl_3$) δ 179.3, 152.8, 136.0, 135.2, 132.7, 130.2, 128.5, 120.7, 118.4, 114.4, 111.0, 92.2, 87.2, 28.6 (hept, $J_{C\text{-}D}$ = 20.8 Hz).

HRMS (ESI) calculated for $C_{16}H_{10}D_3NOH^+$ $[M+H]^+$ 239.1258, found 239.1261.

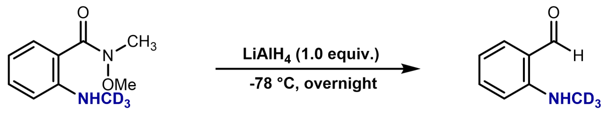

3c 5

Name: 2-((methyl-d$_3$)amino)benzaldehyde (5)

Procedure:[21] To a solution of **3c** (78.8 mg, 0.4 mmol) in THF (3.0 mL) was added

lithium aluminium tetrahydride (0.4 mmol) at -78 °C under Ar atmosphere and stirred overnight, at which point TLC analysis indicated the complete conversion of **3c**. The reaction mixture was then quenched with a saturated NH_4Cl solution (2.0 mL) and extracted with EA (3 × 5 mL). The organic layer was dried over anhydrous Na_2SO_4 and filtered. The reaction mixture was purified by flash column chromatography with PE as elute to afford 44.2 mg of **5**.

Isolated Yield: 80%

Physical Property: bright yellow liquid solid

^{1}H NMR (400 MHz, $CDCl_3$) δ 9.81 (s, 1H), 8.23 (brs, 1H), 7.55 – 7.33 (m, 2H), 6.73 – 6.63 (m, 2H).

^{13}C NMR (151 MHz, $CDCl_3$) δ 193.9, 151.6, 136.6, 135.8, 118.4, 114.7, 110.4, 28.4 (hept, $J_{C\text{-}D}$ = 20.8 Hz).

HRMS (ESI) calculated for $C_8H_6D_3NOH^+$ $[M+H]^+$ 139.0945, found 139.0950.

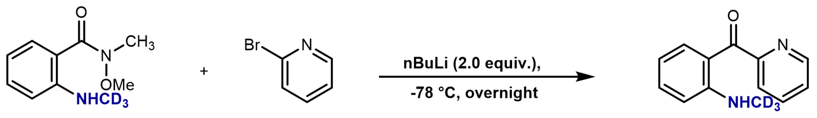

3c 2.0 equiv. 6

Name: (2-((methyl-d$_3$)amino)phenyl)(pyridin-2-yl)methanone (6)

Procedure:[21] To a solution of 2-bromopyridine (62.8 mg, 0.4 mmol) in THF (3.0 mL) was added dropwise n-BuLi (0.4 mmol, 2.5 M in hexane) at -78 ℃ under Ar atmosphere and stirred for 30 min, then **3c** (39.4 mg, 0.2 mmol) was added dropwise and stirred overnight at which point TLC analysis indicated the complete conversion of **3c**. The reaction mixture was quenched with a saturated NH_4Cl solution (2.0 mL) and extracted with EA (3×5 mL). The organic layer was dried over anhydrous Na_2SO_4 and filtered. The reaction mixture was purified by flash column chromatography with (PE:EA=8:1, v/v) as elute to afford 21.9 mg of **6**.

Isolated Yield: 51%

Physical Property: yellow oil

3ac': ^{1}H NMR (400 MHz, $CDCl_3$) δ 8.72 (brs, 1H), 8.68 (dt, J = 4.9, 1.3 Hz, 1H), 7.84 (td, J = 7.7, 1.8 Hz, 1H), 7.69 (d, J = 7.8 Hz, 1H), 7.63 (dd, J = 8.1, 1.6 Hz, 1H), 7.47 – 7.34 (m, 2H), 6.75 (d, J = 8.6 Hz, 1H), 6.54 (t, J = 7.6 Hz, 1H).

^{13}C NMR (151 MHz, $CDCl_3$) δ 196.1, 157.9, 153.4, 148.4, 136.7, 135.7, 135.5, 124.6, 123.7, 115.9, 113.8, 111.1, 28.7 (hept, $J_{C\text{-}D}$ = 21.0 Hz).

HRMS (ESI) calculated for $C_{13}H_9D_3N_2OH^+$ $[M+H]^+$ 216.1211, found 216.1216.

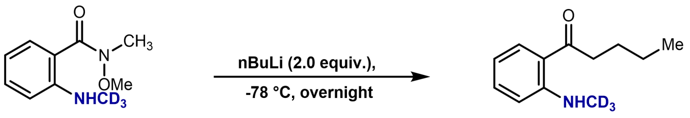

Name: N-butyl-2-((methyl-d$_3$)amino)benzamide (7)

Procedure:[21] To a solution of **3c** (78.8 mg, 0.4 mmol) in THF (3.0 mL) was added dropwise with n-BuLi (0.8 mmol, 2.5 M in hexane) at -78 °C under Ar atmosphere and stirred for 30 min. The reaction mixture was quenched with a saturated NH_4Cl solution (2.0 mL) and extracted with EA (3×5 mL). The organic layer was dried over anhydrous Na_2SO_4 and filtered. The reaction mixture was purified by flash column chromatography with (PE:EA=20:1, v/v) as elute to afford 45.0 mg of **7**.

Isolated Yield: 58%

Physical Property: yellow oil

^{1}H NMR (400 MHz, $CDCl_3$) δ 8.83 (brs, 1H), 7.78 (dd, *J* = 8.1, 1.6 Hz, 1H), 7.49 – 7.31 (m, 1H), 6.69 (d, *J* = 8.1 Hz, 1H), 6.59 (t, *J* = 7.1 Hz, 1H), 2.94 (t, *J* = 7.5 Hz, 2H), 1.86 – 1.56 (m, 2H), 1.47 – 1.31 (m, 2H), 0.95 (t, *J* = 7.3 Hz, 3H).

^{13}C NMR (151 MHz, $CDCl_3$) δ 203.3, 152.0, 134.8, 131.8, 117.2, 113.7, 111.3, 38.9, 28.5 (hept, $J_{C\text{-}D}$ = 20.6 Hz), 27.4, 22.6, 13.9.

HRMS (ESI) calculated for $C_{12}H_{14}D_3NOH^+$ $[M+H]^+$ 195.1571, found 195.1576.

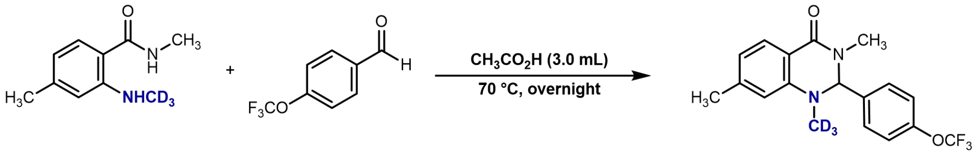

Name:
3,7-dimethyl-1-(methyl-d$_3$)-2-(4-(trifluoromethoxy)phenyl)-2,3-dihydroquinazolin-4(1H)-one (8)

Procedure:[22] To a solution of **3k** (72.5 mg, 0.4 mmol) in CH_3CO_2H (3.0 mL) was added with 4-(trifluoromethoxy)benzaldehyde (76.0 mg, 0.4 mmol) at 70 °C. The resulting solution was stirred overnight, at which point TLC analysis indicated the complete conversion of **3k**. After that, the reaction mixture was cooled to room temperature, which was washed with saturated aqueous $NaHCO_3$, brine, and dried over anhydrous Na_2SO_4. The solvent was then removed under reduced pressure. The reaction mixture was purified by flash column chromatography with (PE:EA=10:1 to 5:1, v/v) as elute to afford 127.1 mg of **8**.

Isolated Yield: 90%

Physical Property: colorless oil

^{1}**H NMR** (400 MHz, $CDCl_3$) δ 7.88 (d, J = 7.8 Hz, 1H), 7.26 – 7.20 (m, 2H), 7.17 – 7.07 (m, 2H), 6.67 (d, J = 7.9 Hz, 1H), 6.29 (s, 1H), 5.40 (s, 1H), 2.98 (s, 3H), 2.30 (s, 3H).

^{13}**C NMR** (151 MHz, $CDCl_3$) δ 162.4, 149.6, 145.8, 144.6, 135.6, 128.5, 127.8, 121.1, 120.3 (q, $J_{C\text{-}F}$ = 257.7 Hz), 119.4, 113.5, 112.1, 80.0, 34.6 (hept, $J_{C\text{-}D}$ = 21.0 Hz), 32.3, 22.1.

19F NMR (376 MHz, $CDCl_3$) δ -57.8 ppm.

HRMS (ESI) calculated for $C_{18}H_{14}D_3F_3N_2O_2H^+$ $[M+H]^+$ 354.1503, found 354.1510.

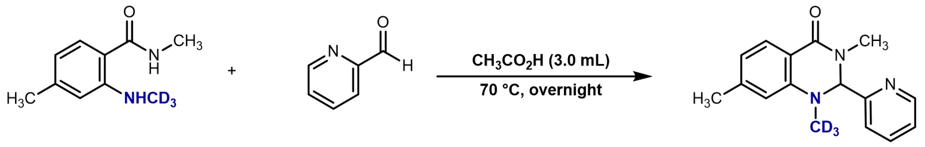

3k 1.0 equiv. 9

Name:
3,7-dimethyl-1-(methyl-d3)-2-(pyridin-2-yl)-2,3-dihydroquinazolin-4(1H)-one (9)

Procedure:[22] To a solution of **3k** (72.5 mg, 0.4 mmol) in CH_3CO_2H (3.0 mL) was added with picolinaldehyde (42.8 mg, 0.4 mmol) at 70 °C. The resulting solution was stirred overnight, at which point TLC analysis indicated the complete conversion of **3k**. After that, the reaction mixture was cooled to room temperature, which was washed with saturated aqueous $NaHCO_3$, brine, and dried over anhydrous Na_2SO_4. The solvent was then removed under reduced pressure. The reaction mixture was purified by flash column chromatography with (PE:EA=2:1 to 1:1, v/v) as elute to afford 78.9 mg of **9**.

Isolated Yield: 73%

Physical Property: colorless oil

^{1}**H NMR** (400 MHz, $CDCl_3$) δ 8.69 – 8.45 (m, 1H), 7.87 (d, J = 7.8 Hz, 1H), 7.56 (t, J = 7.7 Hz, 1H), 7.25 – 7.18 (m, 1H), 7.13 (d, J = 7.9 Hz, 1H), 6.64 (d, J = 7.9 Hz, 1H), 6.30 (s, 1H), 5.55 (s, 1H), 3.06 (s, 3H), 2.30 (s, 3H).

^{13}**C NMR** (151 MHz, $CDCl_3$) δ 162.7, 156.9, 149.1, 146.0, 144.5, 137.3, 128.6, 123.6, 120.2, 119.1, 113.6, 111.9, 81.5, 35.1 (hept, $J_{C\text{-}D}$ = 20.9 Hz), 32.6, 22.1.

HRMS (ESI) calculated for $C_{16}H_{14}D_3N_3OH^+$ $[M+H]^+$ 271.1633, found 271.1633.

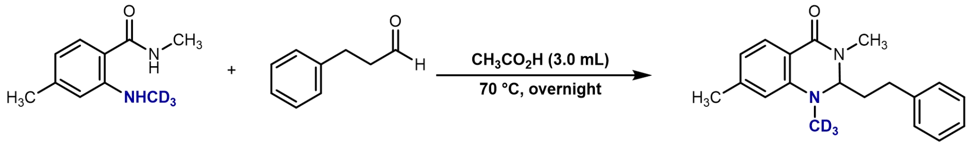

3k 1.0 equiv. 10

Name: 3,7-dimethyl-1-(methyl-d$_3$)-2-phenethyl-2,3-dihydroquinazolin-4(1H)-one (10)

Procedure:[22] To a solution of **3k** (72.5 mg, 0.4 mmol) in CH_3CO_2H (3.0 mL) was added with 3-phenylpropanal (53.6 mg, 0.4 mmol) at 70 °C. The resulting reaction mixture was stirred overnight, at which point TLC analysis indicated the complete conversion of 3k. After that, the reaction mixture was cooled to room temperature, which was washed with saturated aqueous $NaHCO_3$, brine, and dried over anhydrous Na_2SO_4. The solvent was then removed under reduced pressure. The reaction mixture was purified by flash column chromatography with (PE:EA=10:1 to 5:1, v/v) as elute to afford 83.2 mg of **10**.

Isolated Yield: 70%

Physical Property: colorless oil

^{1}H NMR (400 MHz, $CDCl_3$) δ 7.85 (d, J = 7.8 Hz, 1H), 7.33 – 7.27 (m, 2H), 7.23 – 7.17 (m, 1H), 7.16 – 7.09 (m, 2H), 6.69 (d, J = 8.0 Hz, 1H), 6.44 (s, 1H), 4.50 (t, J = 5.9 Hz, 1H), 3.15 (s, 3H), 2.64 (t, J = 8.0 Hz, 2H), 2.36 (s, 3H), 2.13 – 1.97 (m, 2H).

^{13}C NMR (151 MHz, $CDCl_3$) δ 162.8, 146.7, 144.1, 140.6, 128.5, 128.4, 128.1, 126.1, 119.5, 114.9, 113.3, 77.9, 36.7 (hept, $J_{C\text{-}D}$ = 20.9 Hz), 33.9, 33.0, 31.2, 22.0.

HRMS (ESI) calculated for $C_{19}H_{19}D_3N_2OH^+$ $[M+H]^+$ 298.1993, found 298.2001.

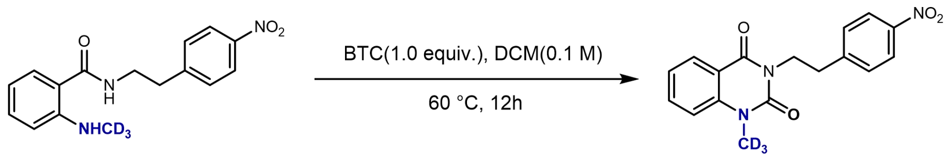

3i

11

Name: 1-(methyl-d$_3$)-3-(4-nitrophenethyl)quinazoline-2,4(1H,3H)-dione (11)

Procedure:[23] A solution of triphosgene (118.7 g, 0.4 mmol, 1.0 equiv.) in DCM was added dropwise to the solution of **3i** (120.8 g, 0.4 mmol, 1.0 equiv.). After stirring at 50 °C for 12 hours, water (2 ml) was added to the reaction solution. The organic layer was washed with saturated aqueous sodium hydrogen carbonate and brine. This solution was dried over anhydrous Na_2SO_4. The filtrate was concentrated under reduced presure. The residue was purified by flash column chromatography with (PE:EA=1:1, v/v) as elute to afford 124.7 mg of **11**.

Isolated Yield: 95%

Physical Property: white solid

^{1}H NMR (400 MHz, $CDCl_3$) δ 8.12 (d, J = 8.2 Hz, 2H), 8.02 (d, J = 7.8 Hz, 1H), 7.53 (t, J = 7.9 Hz, 1H), 7.44 (d, J = 8.3 Hz, 2H), 7.17 (t, J = 7.6 Hz, 1H), 7.01 (d, J = 8.3 Hz, 1H), 3.86 (t, J = 6.9 Hz, 2H), 3.07 (t, J = 6.9 Hz, 2H).

^{13}**C NMR** (151 MHz, $CDCl_3$) δ 148.3, 147.8, 146.4, 145.8, 138.8, 133.4, 129.8, 127.2, 123.8, 123.4, 114.6, 113.3, 46.8, 36.7, 30.8 (hept, $J_{C\text{-}D}$ = 21.5 Hz).

HRMS (ESI) calculated for $C_{17}H_{12}D_3N_3O_4H^+$ $[M+H]^+$ 329.1324, found 329.1332.

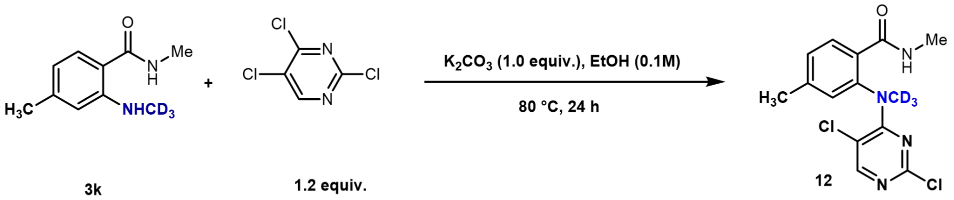

Name: 2-((2,5-dichloropyrimidin-4-yl)(methyl-d$_3$)amino)-N,4-dimethylbenzamide (12)

Procedure:[24] 2,4,5-Trichloropyrimidine (87.3 mg, 0.48 mmol)) was added into a solution of **3k** (72.5 mg, 0.4 mmol) and K_2CO_3 (55.3 g, 0.4 mmol) in EtOH (4.0 mL). The resulting mixture was stirred at 80 °C for 24 hours. The mixture was then concentrated and the resulting solid was partitioned between EtOAc (5 mL) and water (5 mL). The organic layer was washed with brine and dried over anhydrous Na_2SO_4. The solvent was then removed under reduced pressure, and the residue was purified by flash column chromatography with (PE:EA=2:1, v/v) as elute to afford 69.3 mg of **12**.

Isolated Yield: 53%

Physical Property: yellow oil

^{1}**H NMR** (400 MHz, $CDCl_3$) δ 8.00 (s, 1H), 7.62 (d, J = 7.8 Hz, 1H), 7.15 (d, J = 7.8 Hz, 1H), 6.80 (s, 1H), 6.46 (brs, 1H), 2.88 (d, J = 5.1 Hz, 3H), 2.31 (s, 3H).

^{13}**C NMR** (151 MHz, $CDCl_3$) δ 167.0, 159.7, 157.7, 157.6, 142.2, 142.1, 129.8, 128.7, 127.6, 115.3, 41.3 (hept, $J_{C\text{-}D}$ = 21.5 Hz), 26.8, 21.0.

HRMS (ESI) calculated for $C_{14}H_{11}D_3Cl_2N_4OH^+$ $[M+H]^+$ 328.0806, found 328.0812.

10. UnsuccessfulSubstrates

Substrates with Low Yields for N-CD_3 Amination Reaction of Arene

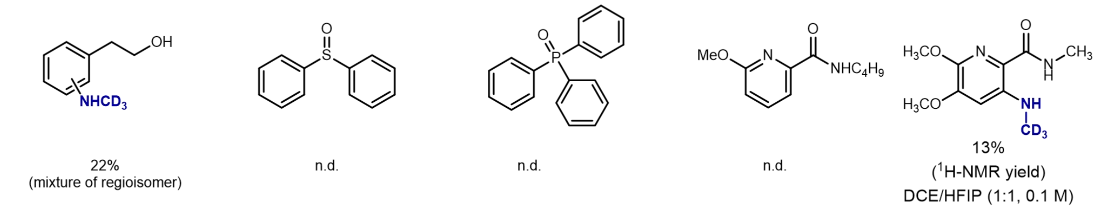

Reactions with *N,N*-Dimethyl Reagent

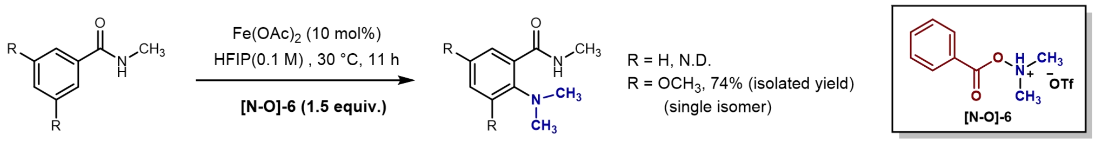

Observation of Aminative Defunctionalization

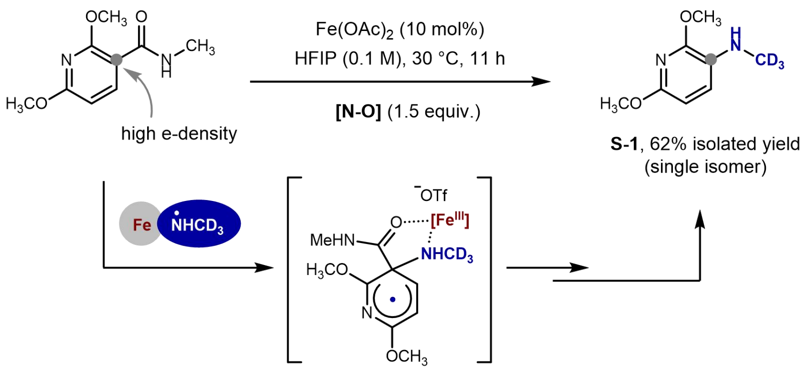

11. NMR Spectra of Substrates and Products

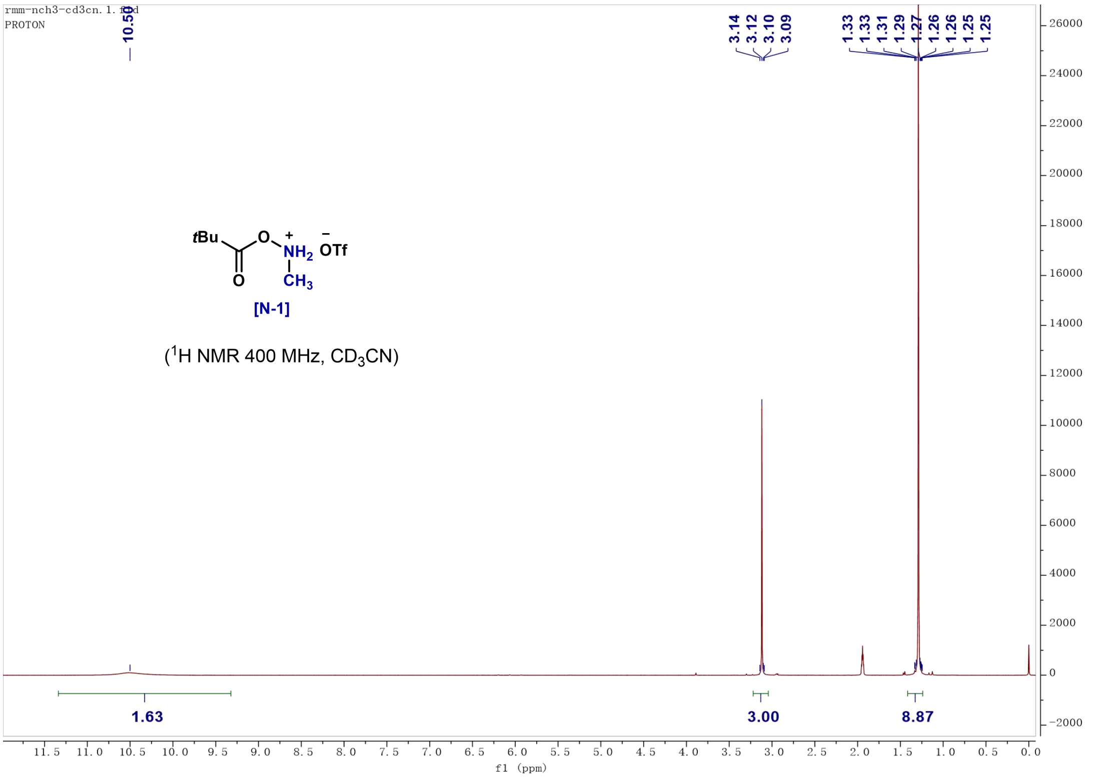

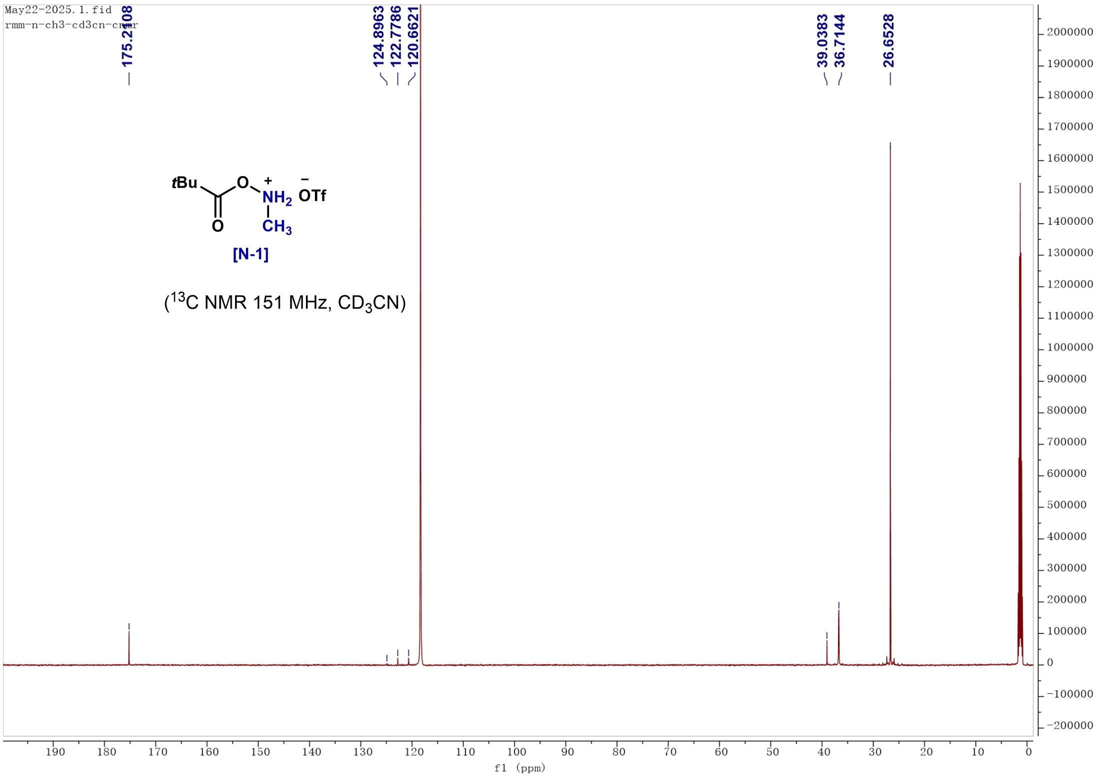

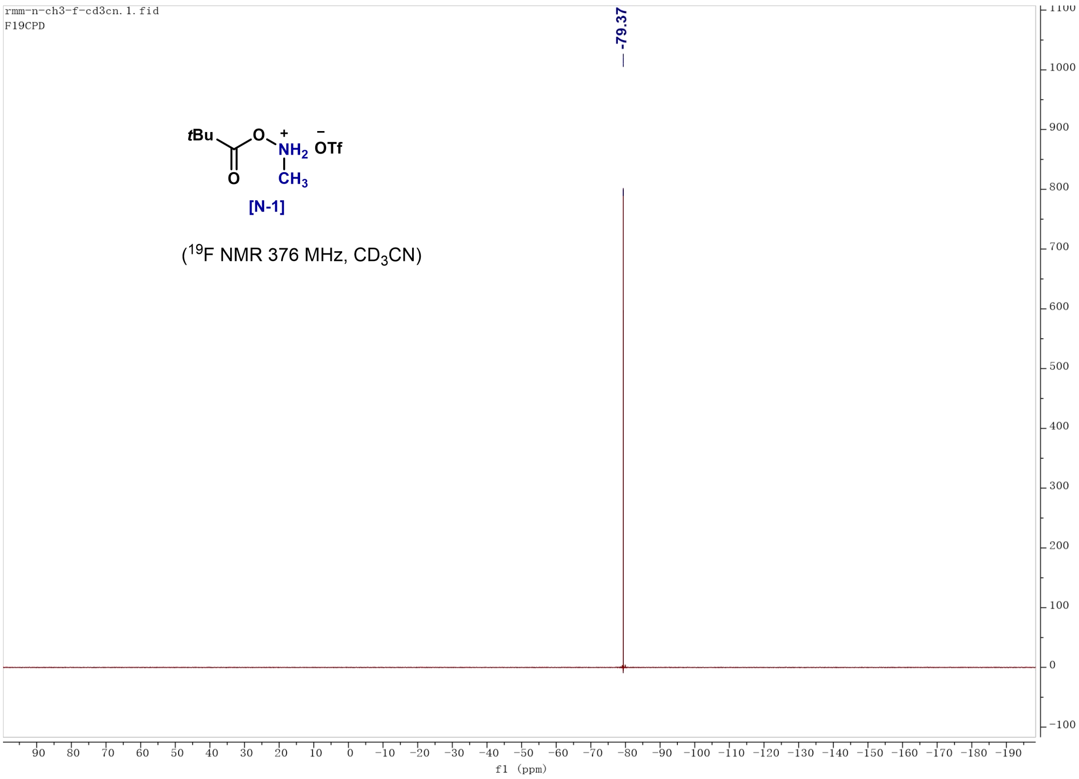
rmm-n-ch3-f-cd3cn.1.fid
F19CPD
-79.37
tBu
O
NH2
OTf
CH3
[N-1]
(19F NMR 376 MHz, CD3CN)
f1 (ppm)

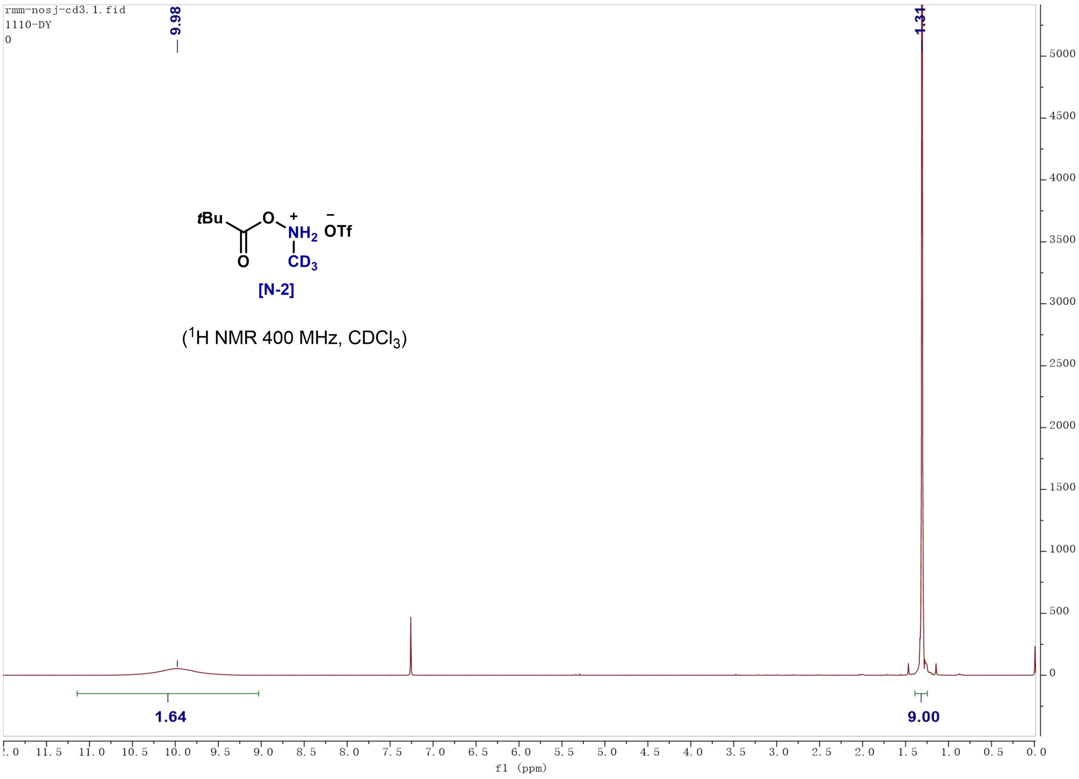
rmm-nosj-cd3.1.fid
1110-DY
0
9.98
1.34
tBu
O
NH2
OTf
CD3
[N-2]
(1H NMR 400 MHz, CDCl3)
1.64
9.00
f1 (ppm)

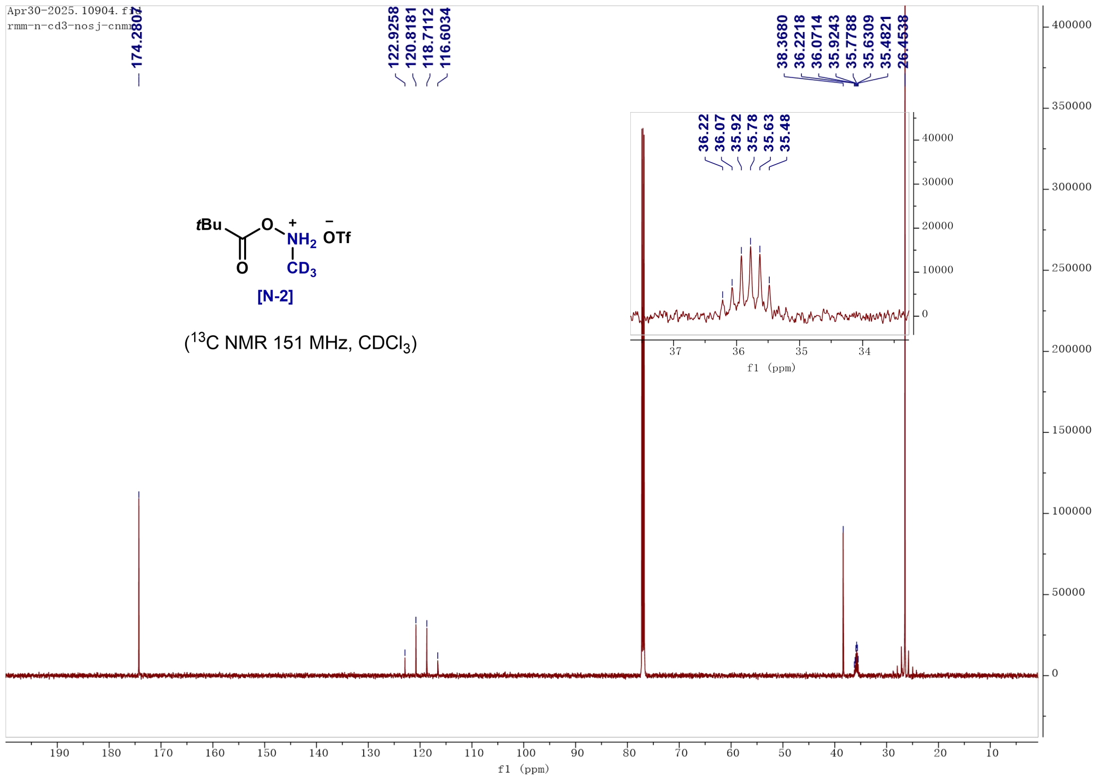
Apr30-2025.10904.fid
rmm-n-cd3-nosj-cnmr
174.2807
122.9258
120.8181
118.7112
116.6034
38.3680
36.2218
36.0714
35.9243
35.7788
35.6309
35.4821
26.4538
36.22
36.07
35.92
35.78
35.63
35.48
tBu
O
NH2
OTf
O
CD3
[N-2]
(13C NMR 151 MHz, CDCl3)
f1 (ppm)

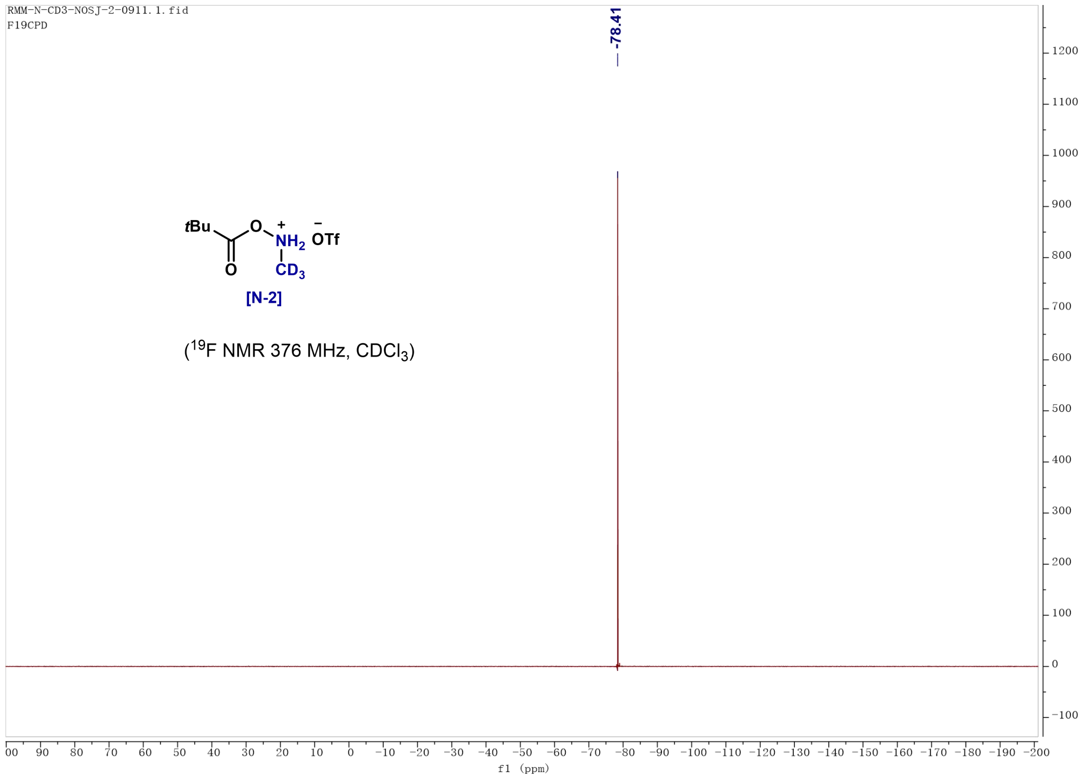
RMM-N-CD3-NOSJ-2-0911.1.fid
F19CPD
-78.41
tBu
O
NH2
OTf
O
CD3
[N-2]
(19F NMR 376 MHz, CDCl3)
f1 (ppm)

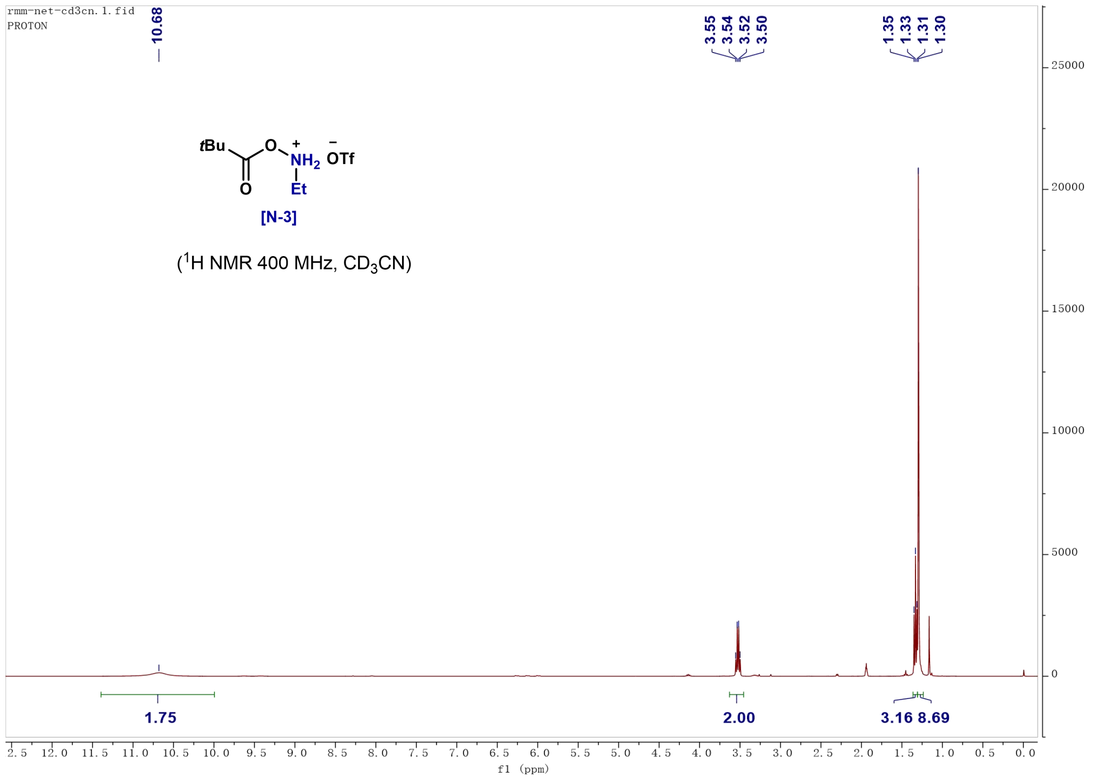
rmm-net-cd3cn.1.fid
PROTON
10.68
3.55
3.54
3.52
3.50
1.35
1.33
1.31
1.30
tBu
O
NH2
OTf
O
Et
[N-3]
(1H NMR 400 MHz, CD3CN)
1.75
2.00
3.16 8.69
f1 (ppm)

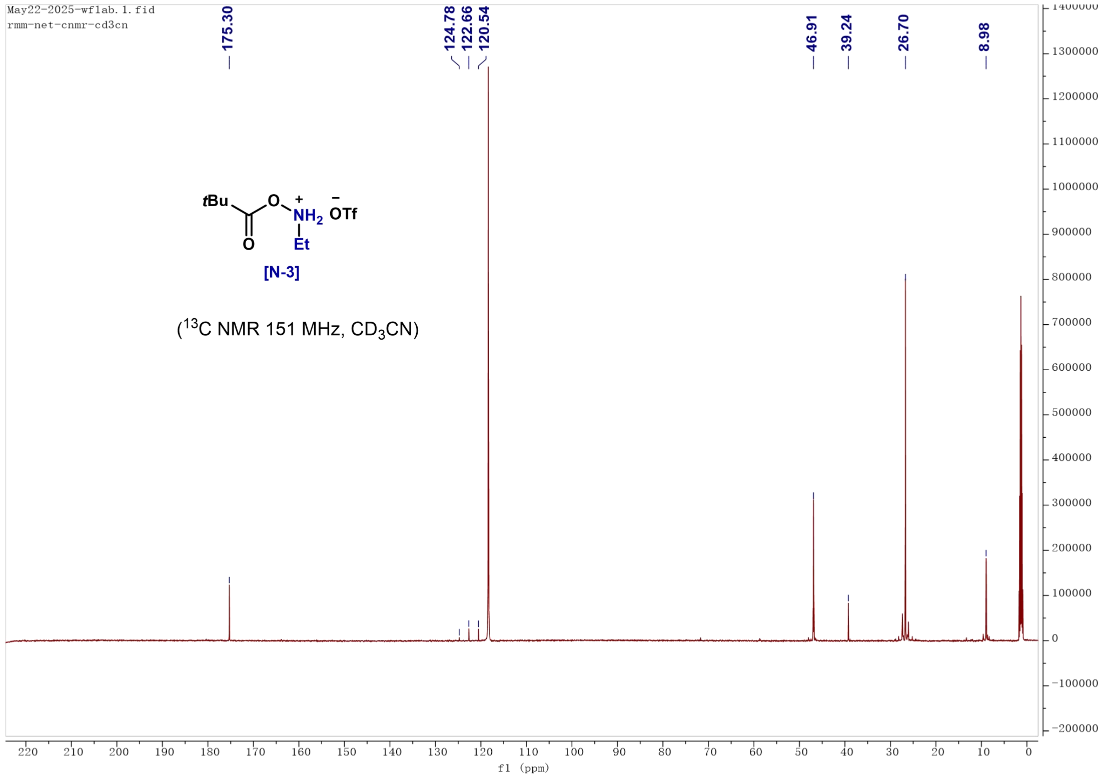
May22-2025-wf1ab.1.fid
rmm-net-cnmr-cd3cn
175.30
124.78
122.66
120.54
46.91
39.24
26.70
8.98
tBu
O
NH2
OTf
O
Et
[N-3]
(13C NMR 151 MHz, CD3CN)
f1 (ppm)

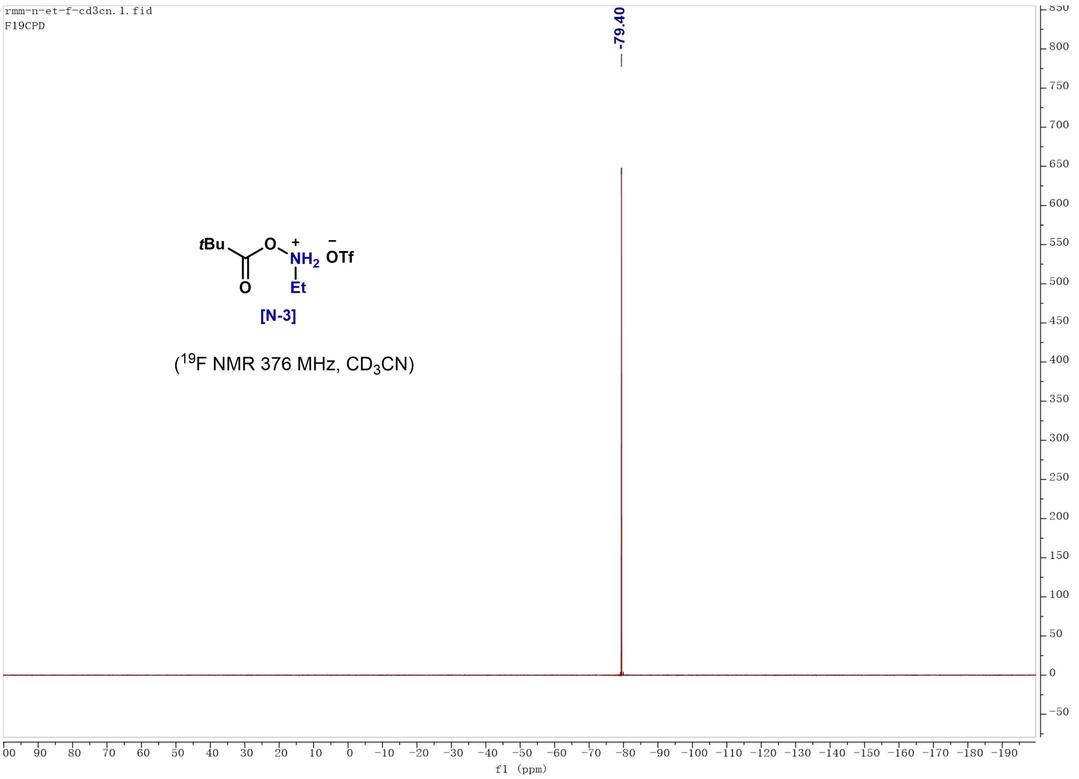
rmm-n-et-f-cd3cn.1.fid
F19CPD
-79.40
tBu
O
NH2
OTf
O
Et
[N-3]
(19F NMR 376 MHz, CD3CN)
f1 (ppm)

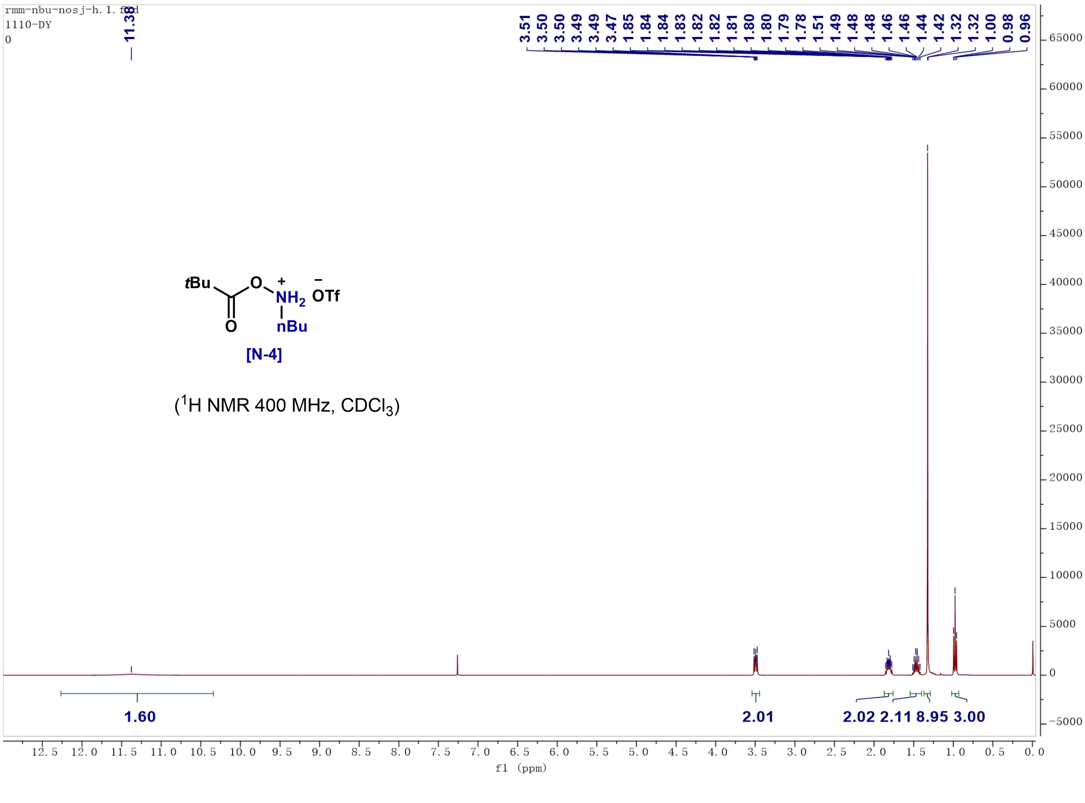
rmm-nbu-nosj-h.1.fid
1110-DY
0
11.38
3.51
3.50
3.50
3.49
3.49
3.47
1.85
1.84
1.83
1.83
1.82
1.81
1.80
1.80
1.79
1.78
1.51
1.49
1.48
1.48
1.46
1.46
1.44
1.42
1.32
1.32
1.00
0.98
0.96
tBu
O
NH2
OTf
O
nBu
[N-4]
(1H NMR 400 MHz, CDCl3)
1.60
2.01
2.02
2.11
8.95
3.00
f1 (ppm)

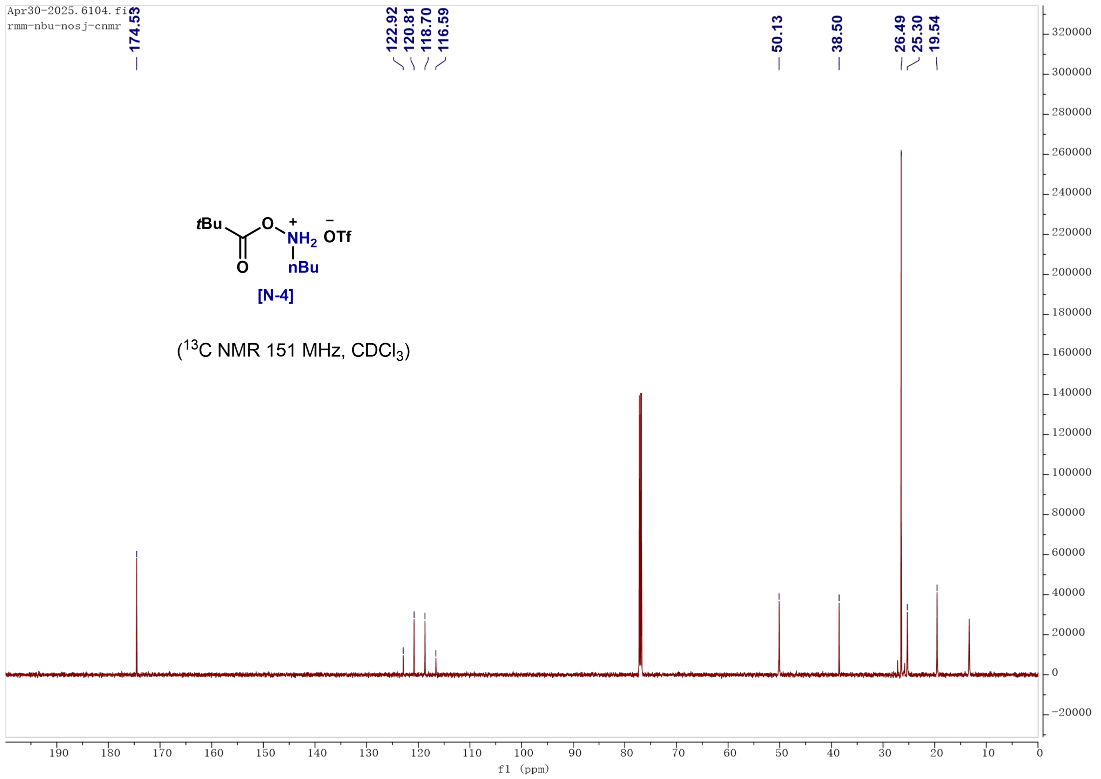

Apr30-2025.6104.fid
rmm-nbu-nosj-cnmr
174.53
122.92
120.81
118.70
116.59
50.13
38.50
26.49
25.30
19.54
tBu
O
NH2
OTf
O
nBu
[N-4]
(13C NMR 151 MHz, CDCl3)
f1 (ppm)

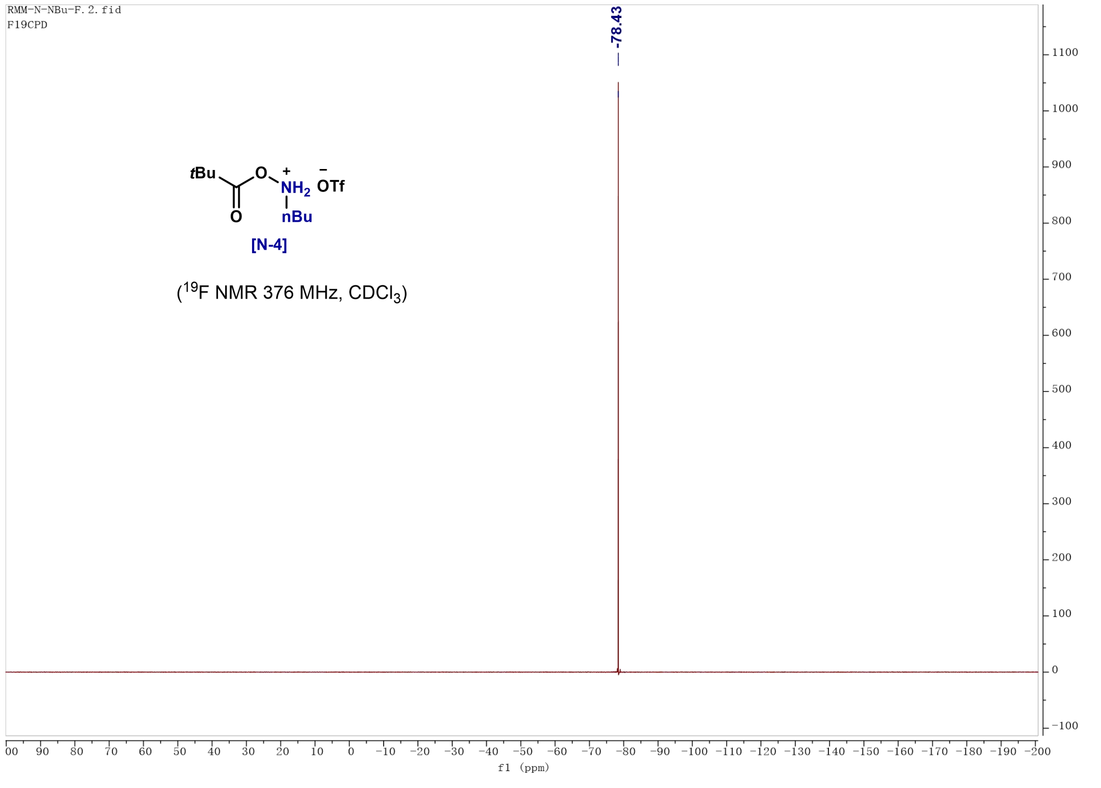

RMM-N-NBu-F.2.fid
F19CPD
-78.43
tBu
O
NH2
OTf
O
nBu
[N-4]
(19F NMR 376 MHz, CDCl3)
f1 (ppm)

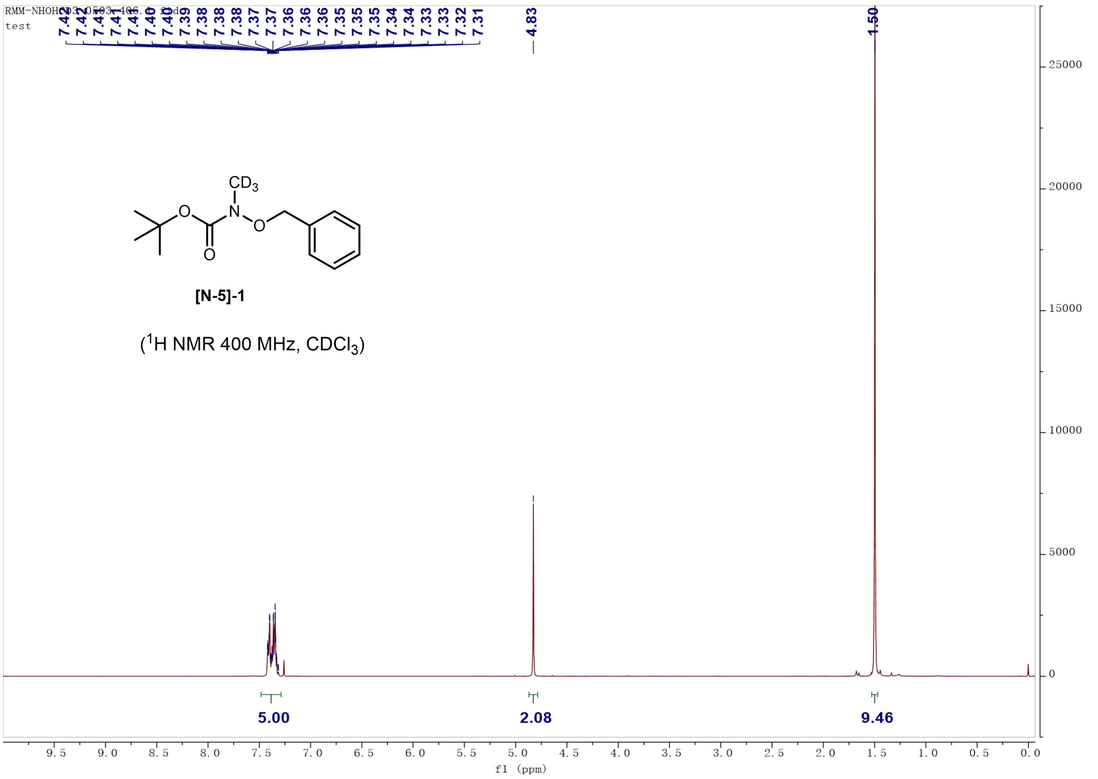
test
7.42
7.42
7.41
7.41
7.40
7.40
7.39
7.38
7.38
7.38
7.37
7.37
7.36
7.36
7.35
7.35
7.35
7.34
7.34
7.33
7.33
7.32
7.31
4.83
1.50
CD3
N
O
O
O
[N-5]-1
(1H NMR 400 MHz, CDCl3)
5.00
2.08
9.46
f1 (ppm)

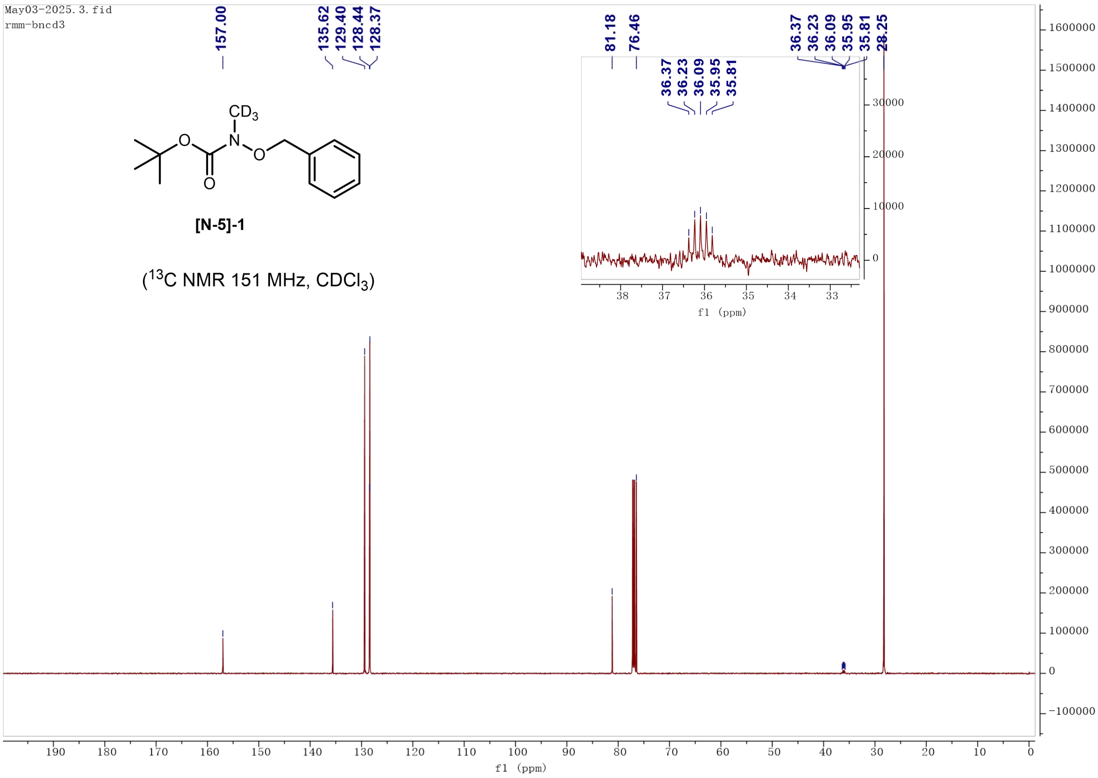
May03-2025.3.fid
rmm-bncd3
157.00
135.62
129.40
128.44
128.37
81.18
76.46
36.37
36.23
36.09
35.95
35.81
28.25
CD3
N
O
O
O
[N-5]-1
(13C NMR 151 MHz, CDCl3)
f1 (ppm)

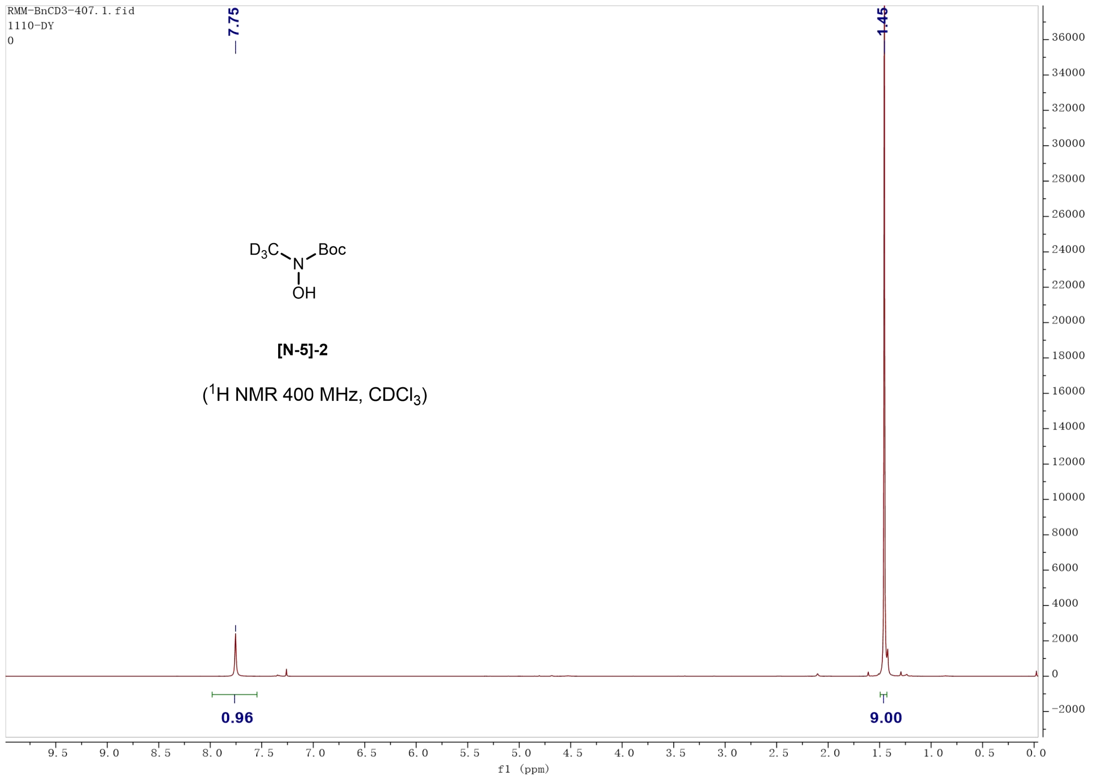

RMM-BnCD3-407.1.fid
1110-DY
0
7.75
1.45
D_3C N Boc
OH
[N-5]-2
(^{1}H NMR 400 MHz, $CDCl_3$)
0.96
9.00
f1 (ppm)

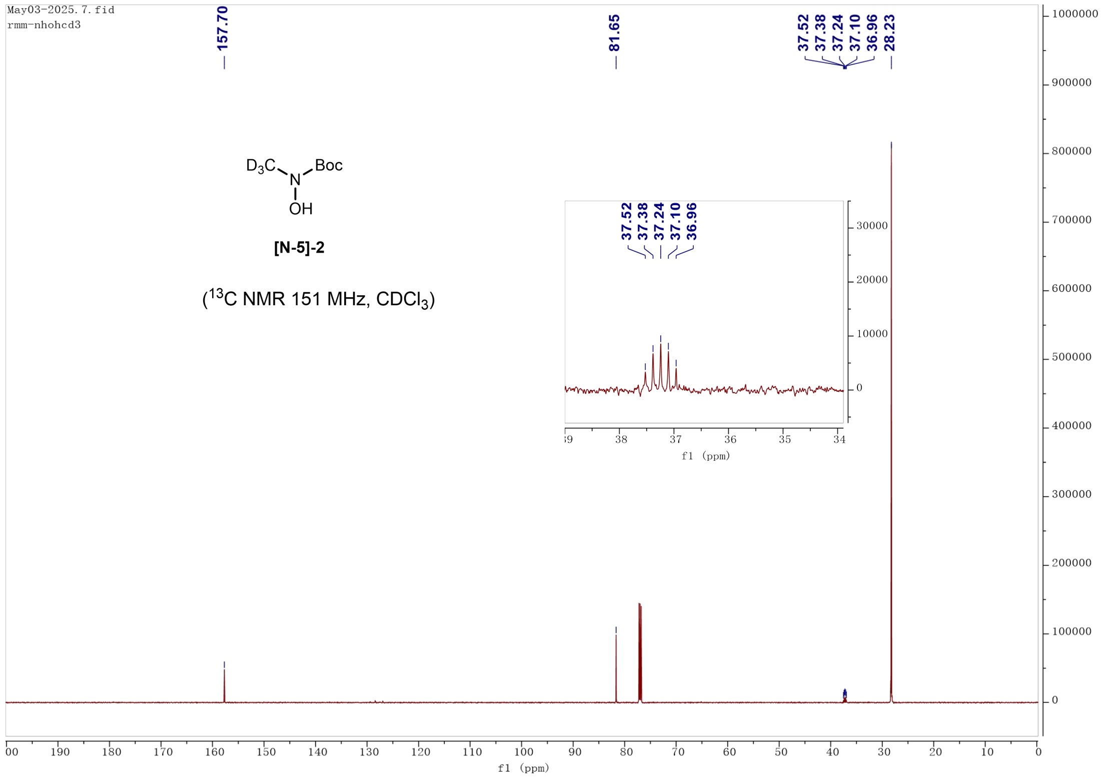

May03-2025.7.fid
rmm-nhohcd3
157.70
81.65
37.52
37.38
37.24
37.10
36.96
28.23
D_3C N Boc
OH
[N-5]-2
(^{13}C NMR 151 MHz, $CDCl_3$)
f1 (ppm)

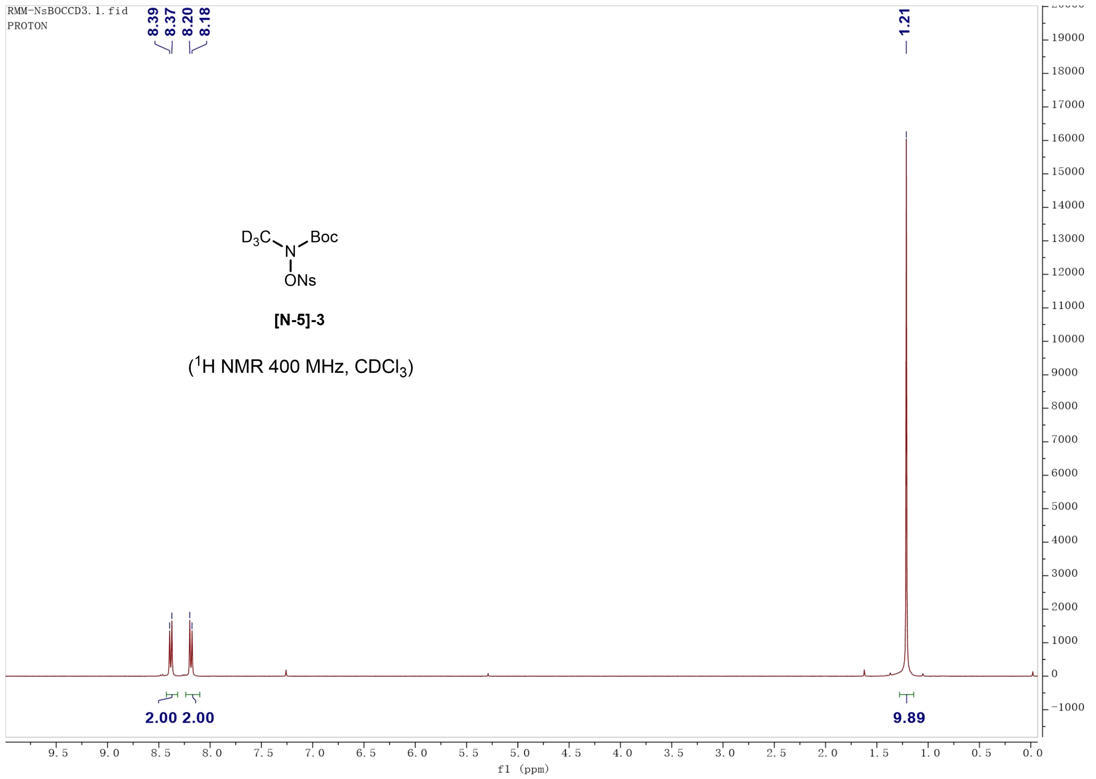
RMM-NsBOCCD3.1.fid
PROTON
8.39
8.37
8.20
8.18
1.21
D3C
N
Boc
ONs
[N-5]-3
(1H NMR 400 MHz, CDCl3)
2.00
2.00
9.89
f1 (ppm)

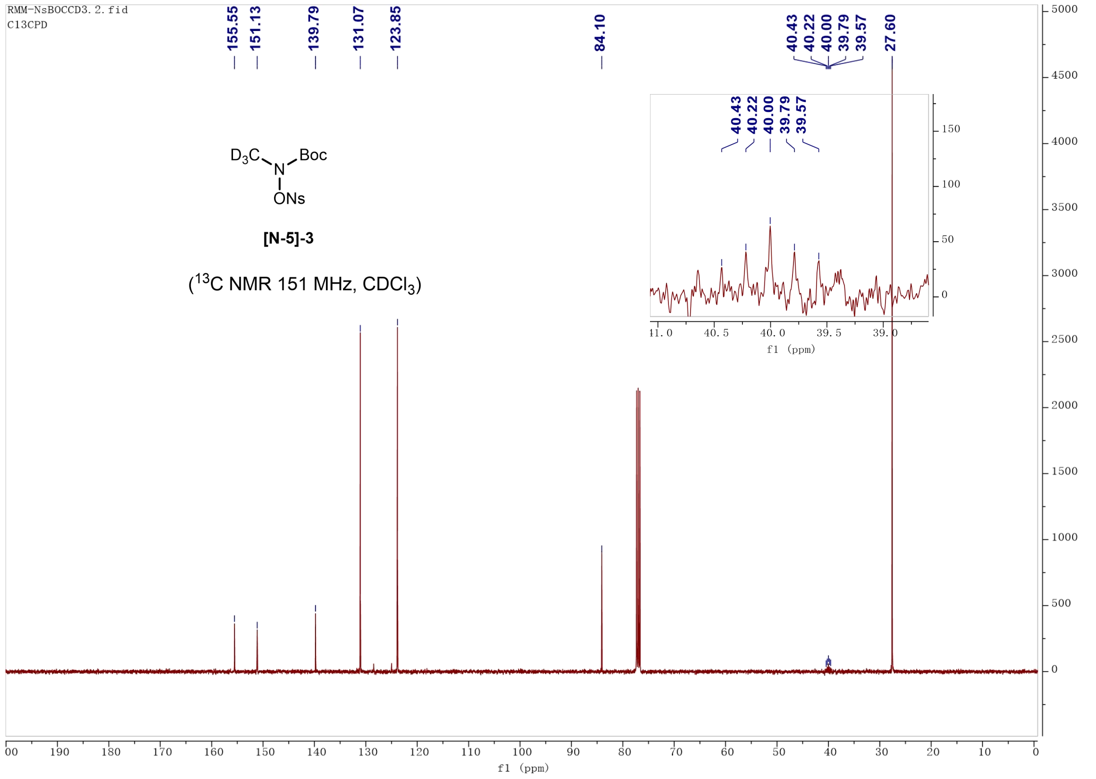
RMM-NsBOCCD3.2.fid
C13CPD
155.55
151.13
139.79
131.07
123.85
84.10
40.43
40.22
40.00
39.79
39.57
27.60
D3C
N
Boc
ONs
[N-5]-3
(13C NMR 151 MHz, CDCl3)
f1 (ppm)

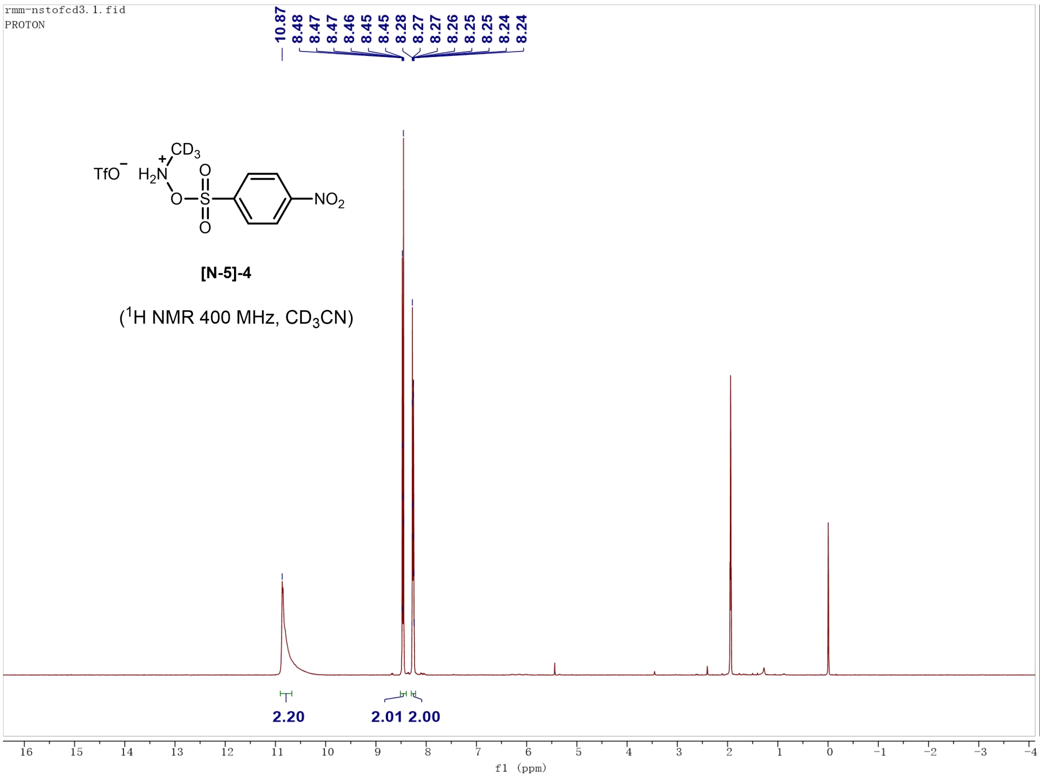
rmm-nstofcd3.1.fid
PROTON
10.87
8.48
8.47
8.47
8.46
8.45
8.45
8.28
8.27
8.27
8.26
8.25
8.24
8.24
TfO⁻
CD3
H2N
O
S
NO2
[N-5]-4
(1H NMR 400 MHz, CD3CN)
2.20
2.01
2.00
f1 (ppm)

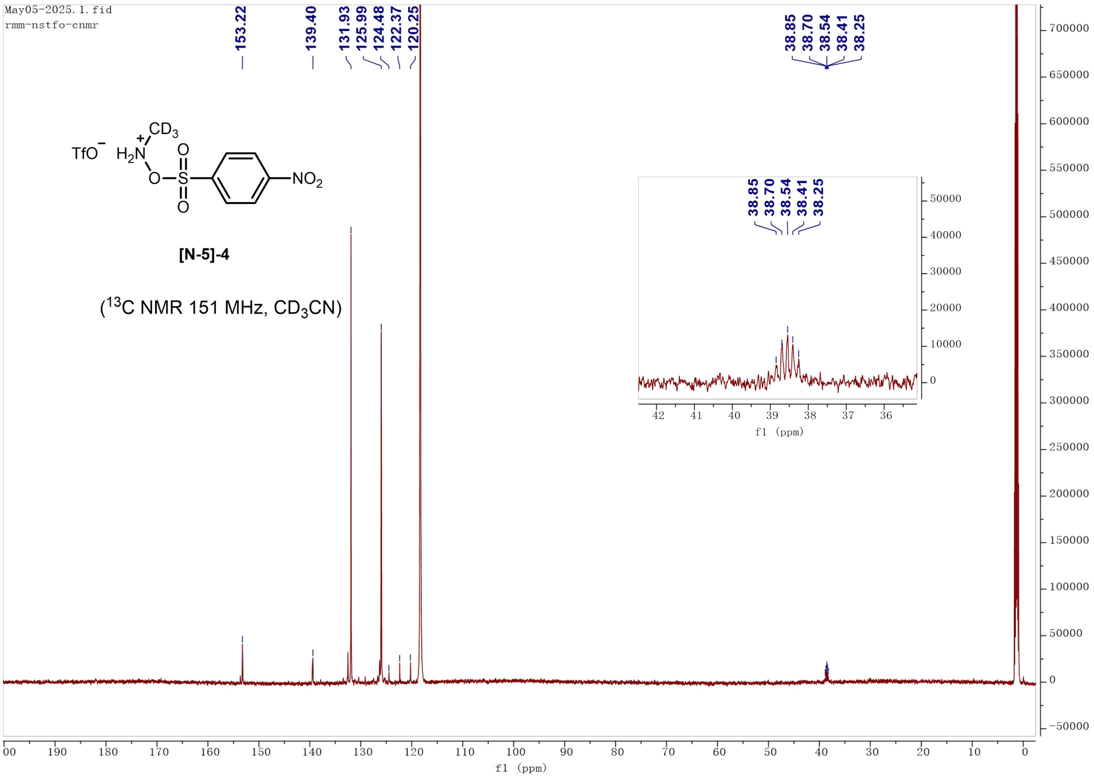
May05-2025.1.fid
rmm-nstfo-cnmr
153.22
139.40
131.93
125.99
124.48
122.37
120.25
38.85
38.70
38.54
38.41
38.25
TfO⁻
CD3
H2N
O
S
NO2
[N-5]-4
(13C NMR 151 MHz, CD3CN)
f1 (ppm)

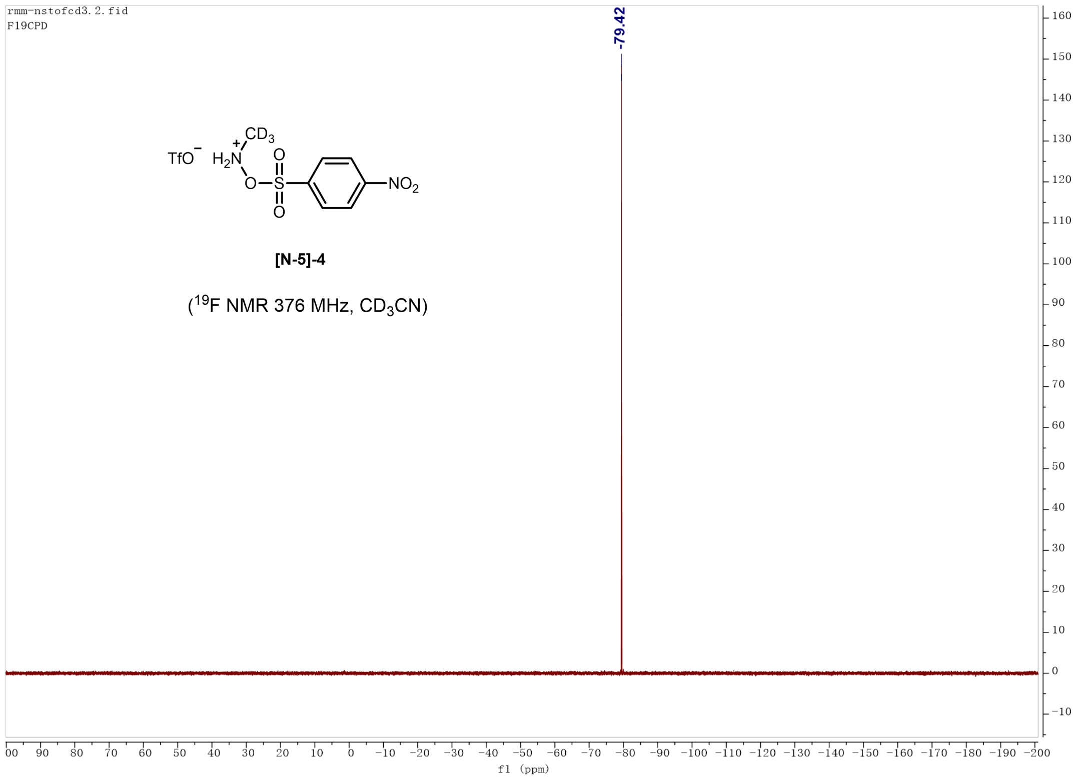
rmm-nstofcd3.2.fid
F19CPD
-79.42
TfO⁻
[N-5]-4
(19F NMR 376 MHz, CD3CN)
f1 (ppm)

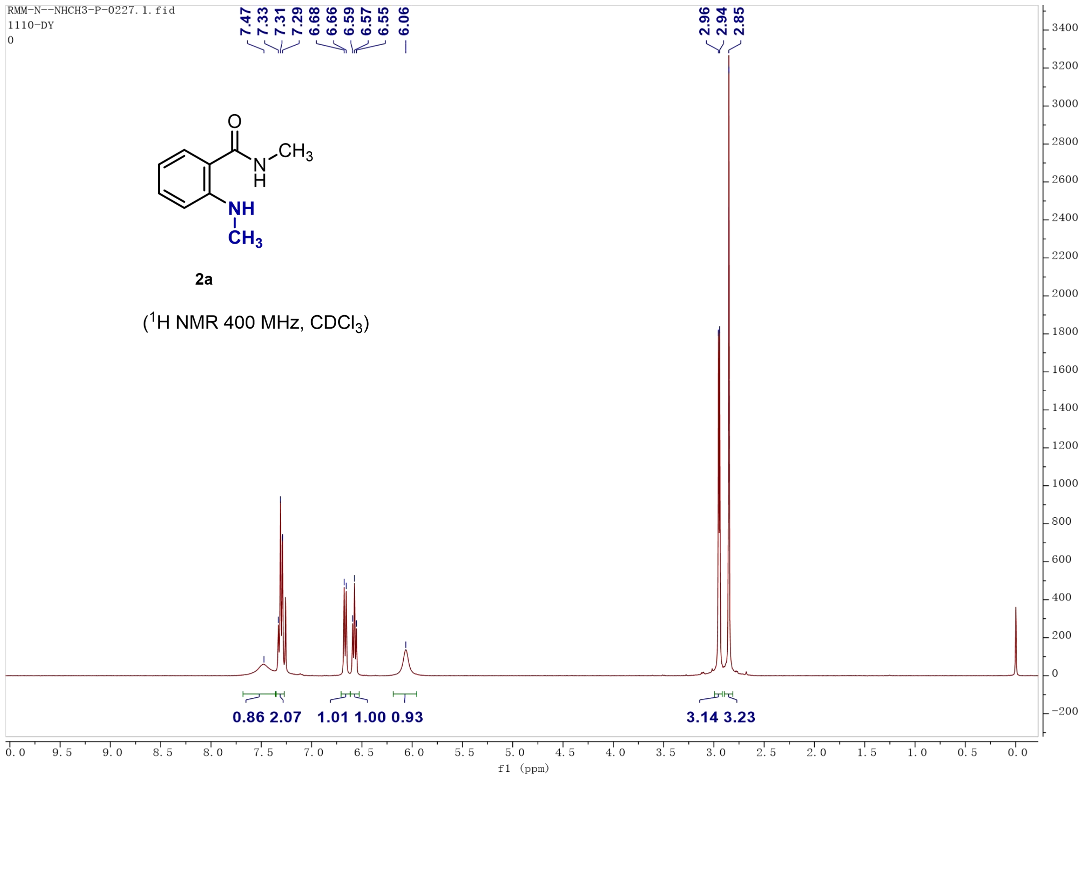
RMM-N--NHCH3-P-0227.1.fid
1110-DY
0
7.47
7.33
7.31
7.29
6.68
6.66
6.59
6.57
6.55
6.06
2.96
2.94
2.85
2a
(1H NMR 400 MHz, CDCl3)
0.86 2.07 1.01 1.00 0.93
3.14 3.23
f1 (ppm)

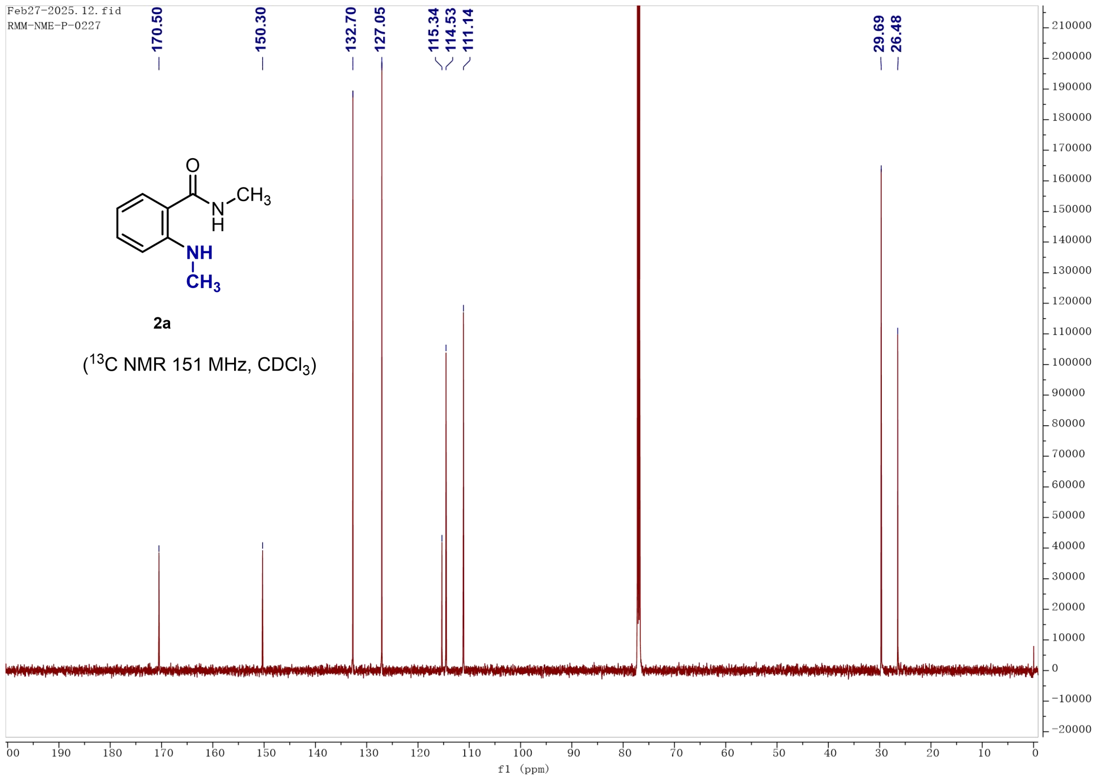
Feb27-2025.12.fid
RMM-NME-P-0227
170.50
150.30
132.70
127.05
115.34
114.53
111.14
29.69
26.48
2a
(^{13}C NMR 151 MHz, $CDCl_3$)
f1 (ppm)

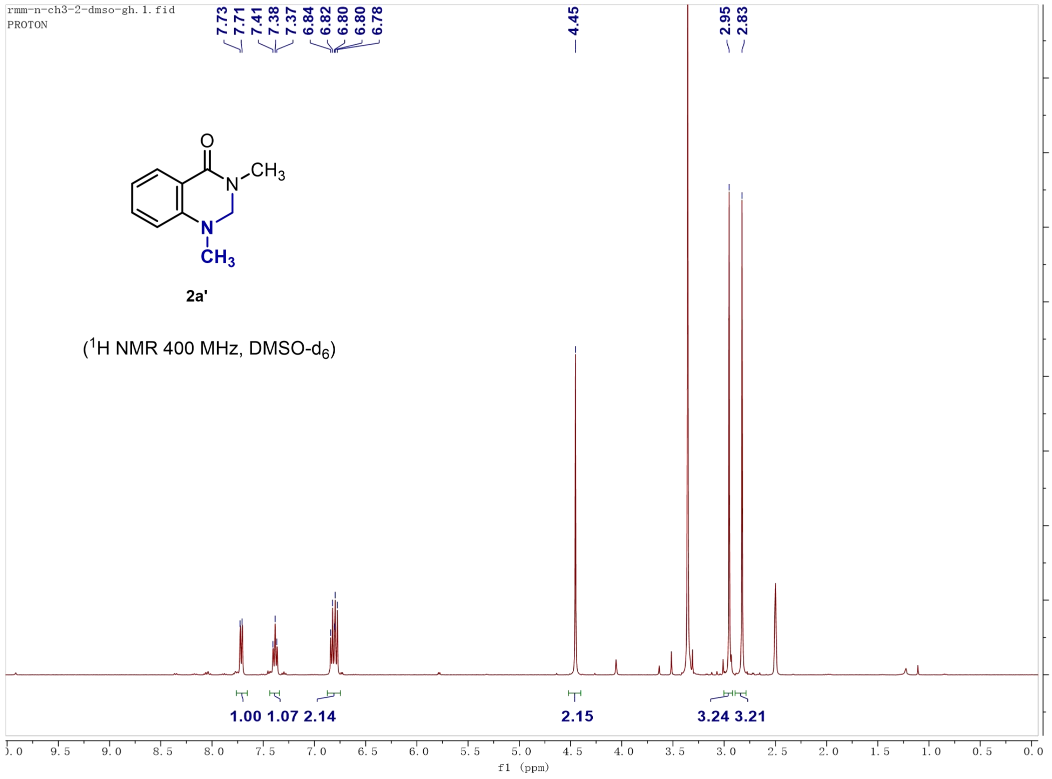
rmm-n-ch3-2-dmso-gh.1.fid
PROTON
7.73
7.71
7.41
7.38
7.37
6.84
6.82
6.80
6.80
6.78
4.45
2.95
2.83
2a'
(^{1}H NMR 400 MHz, DMSO-d_6)
1.00
1.07
2.14
2.15
3.24
3.21
f1 (ppm)

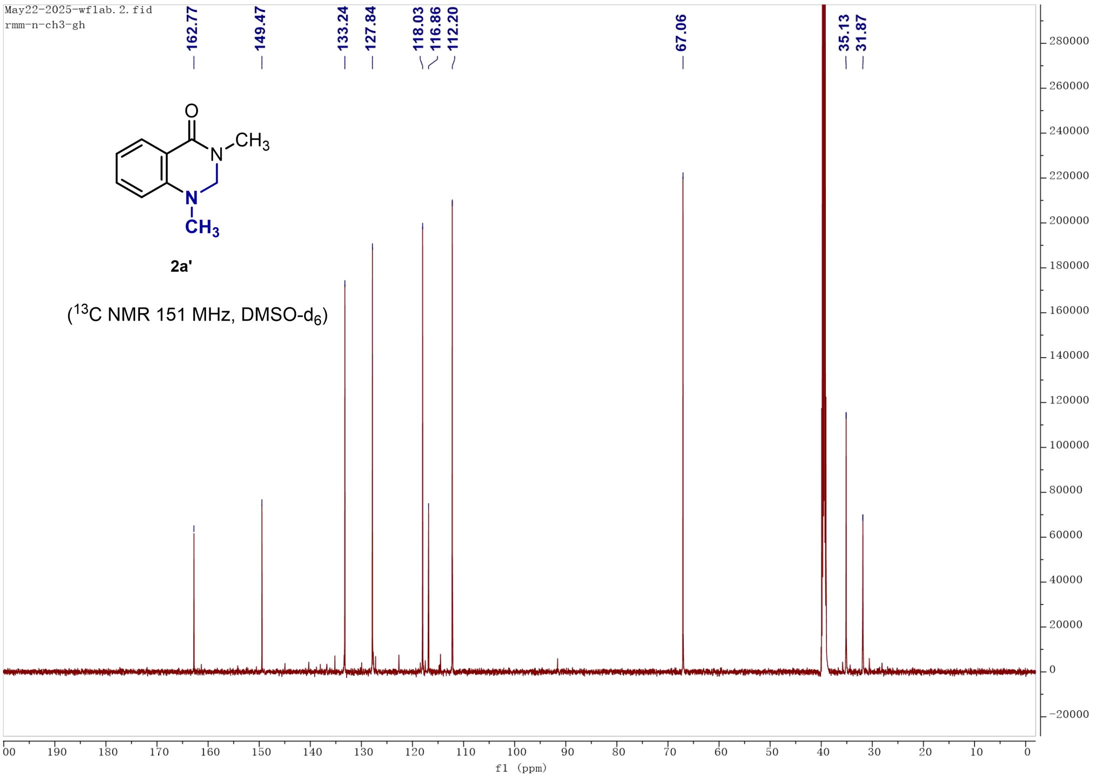

2a'

(^{13}C NMR 151 MHz, DMSO-d_6)

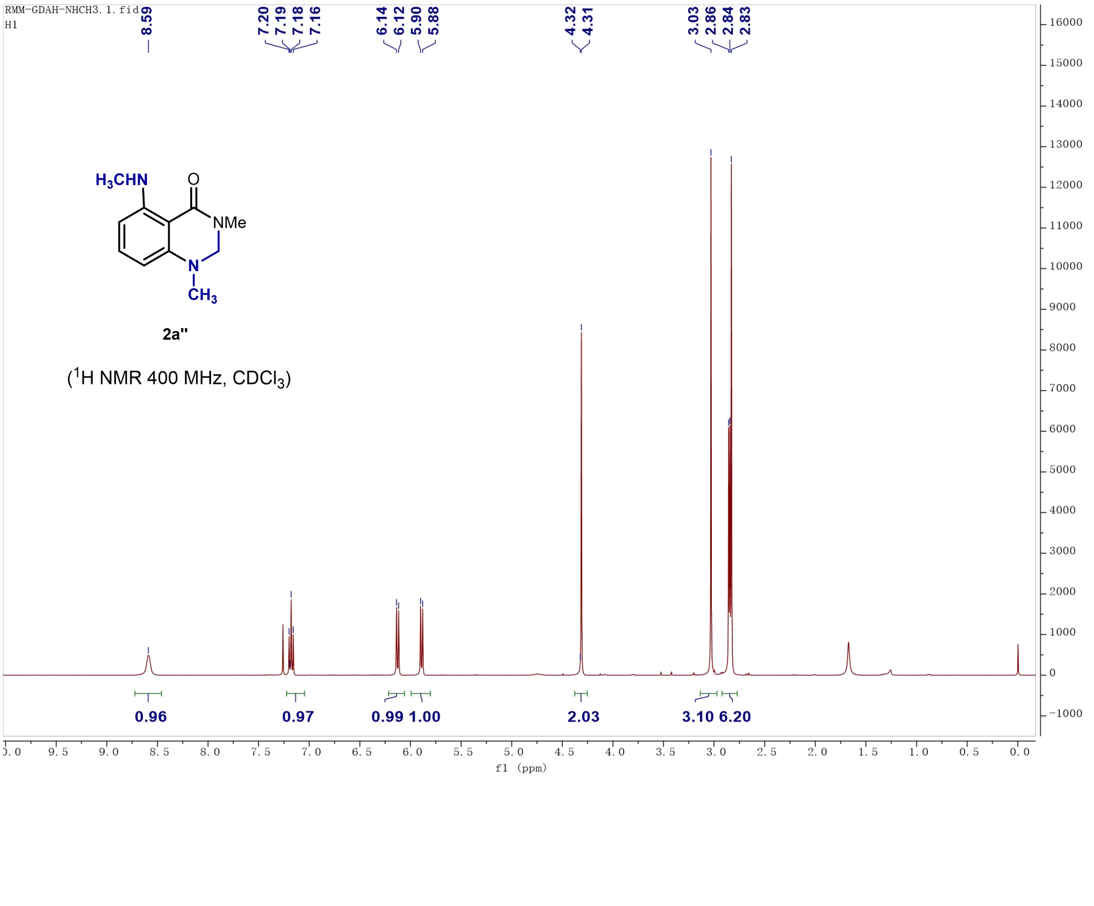

2a''

(^1H NMR 400 MHz, $CDCl_3$)

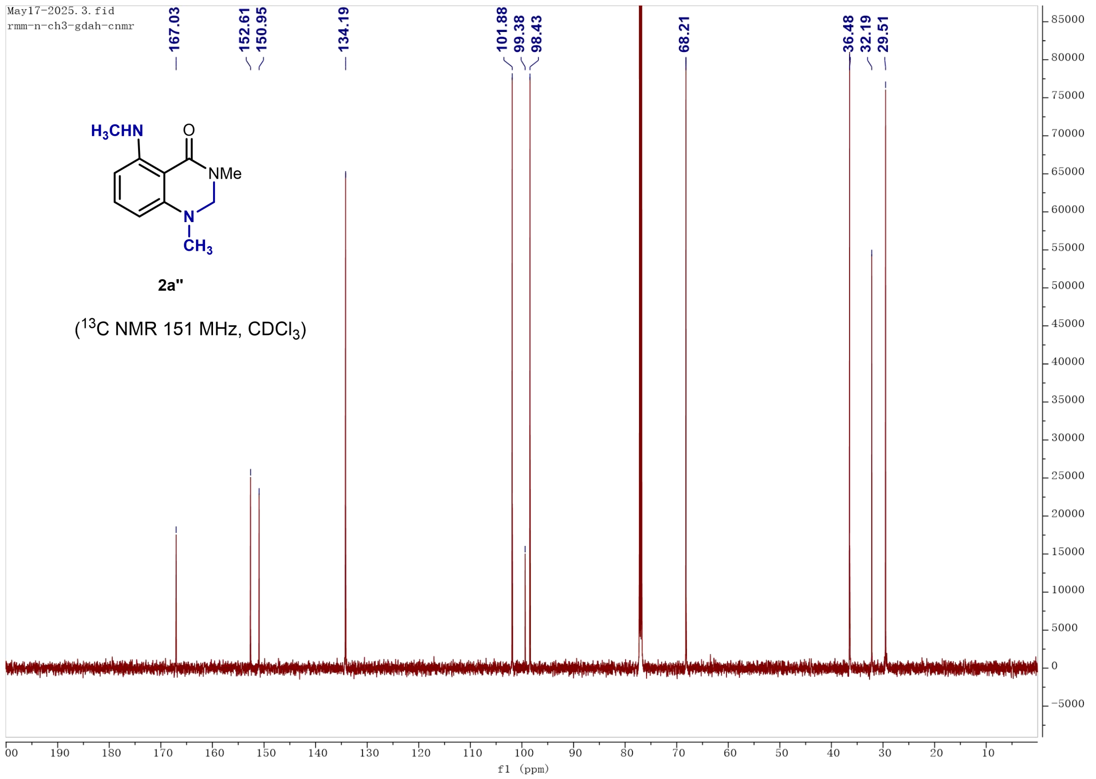
May17-2025.3.fid
rmm-n-ch3-gdah-cnmr
167.03
152.61
150.95
134.19
101.88
99.38
98.43
68.21
36.48
32.19
29.51
H3CHN
O
NMe
N
CH3
2a''
(13C NMR 151 MHz, CDCl3)
f1 (ppm)

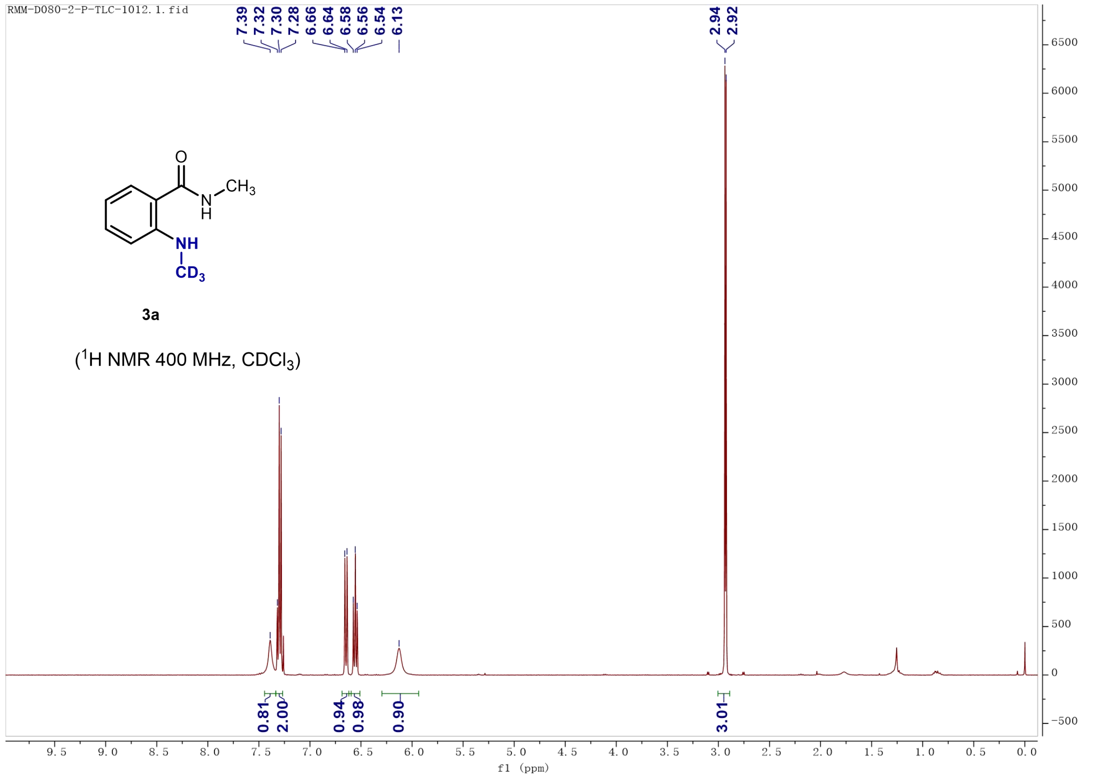
RMM-D080-2-P-TLC-1012.1.fid
7.39
7.32
7.30
7.28
6.66
6.64
6.58
6.56
6.54
6.13
2.94
2.92
O
N
H
CH3
NH
CD3
3a
(1H NMR 400 MHz, CDCl3)
0.81
2.00
0.94
0.98
0.90
3.01
f1 (ppm)

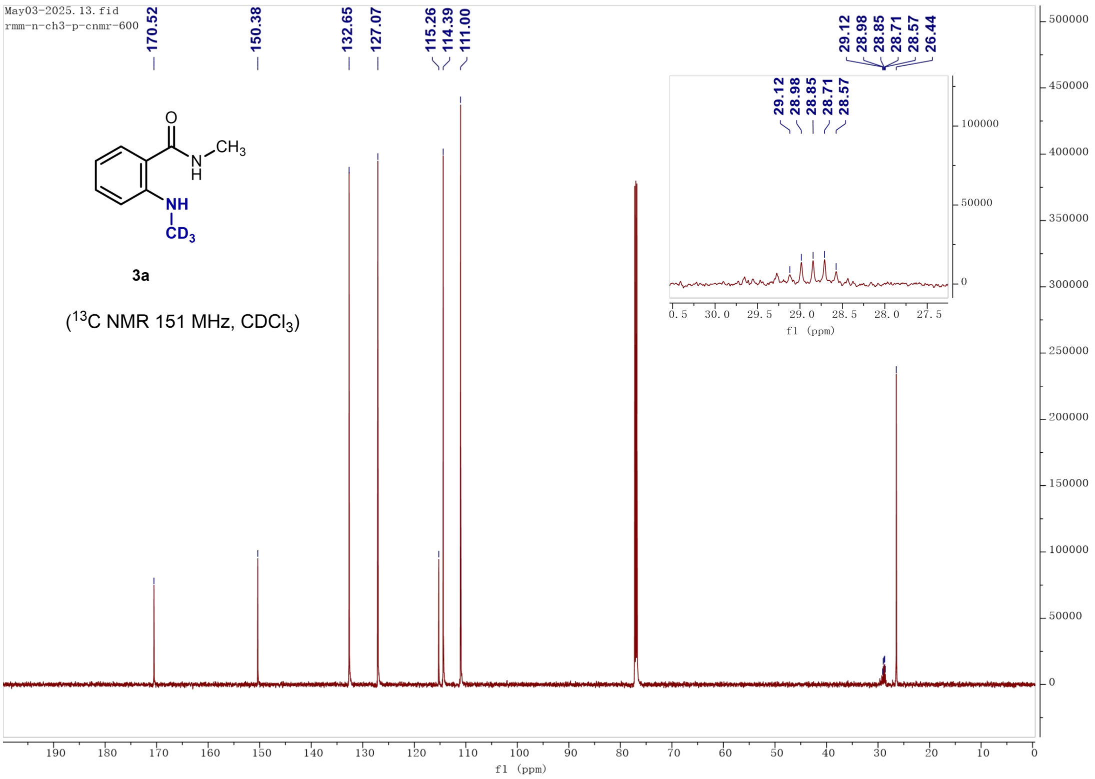
May03-2025.13.fid
rmm-n-ch3-p-cnmr-600
170.52
150.38
132.65
127.07
115.26
114.39
111.00
29.12
28.98
28.85
28.71
28.57
26.44
3a
(13C NMR 151 MHz, CDCl3)
f1 (ppm)

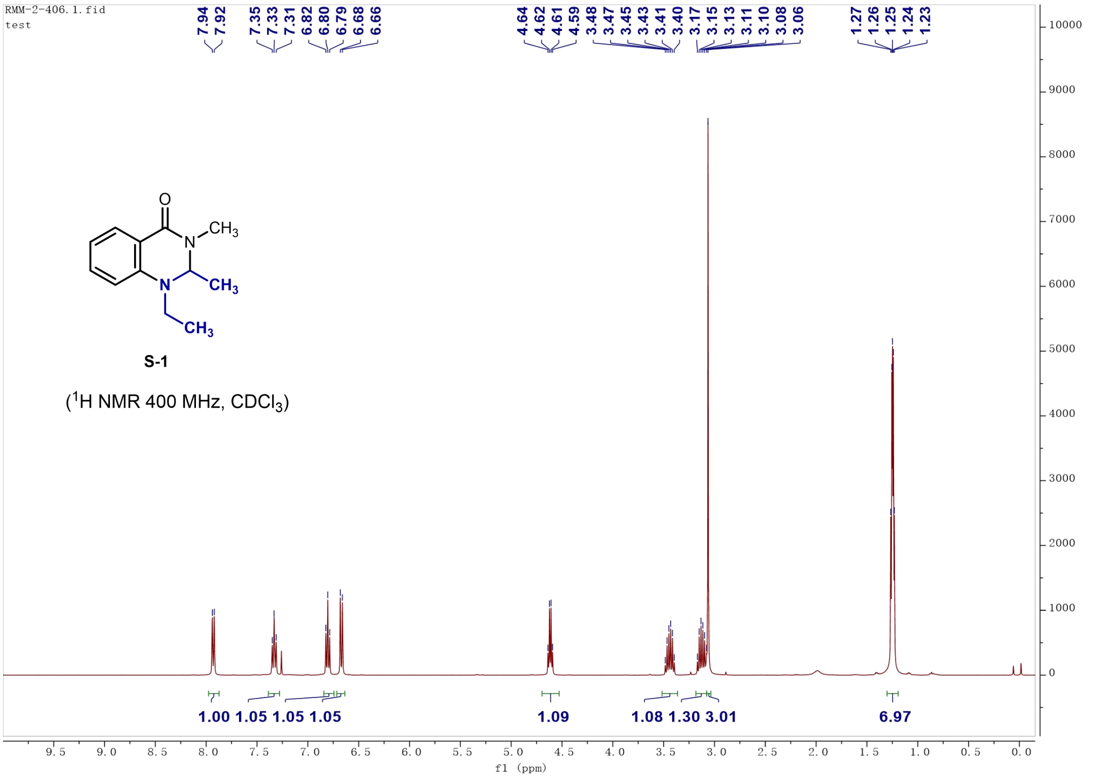
RMM-2-406.1.fid
test
7.94
7.92
7.35
7.33
7.31
6.82
6.80
6.79
6.68
6.66
4.64
4.62
4.61
4.59
3.48
3.47
3.45
3.43
3.41
3.40
3.17
3.15
3.13
3.11
3.10
3.08
3.06
1.27
1.26
1.25
1.24
1.23
S-1
(1H NMR 400 MHz, CDCl3)
1.00 1.05 1.05 1.05
1.09
1.08 1.30 3.01
6.97
f1 (ppm)

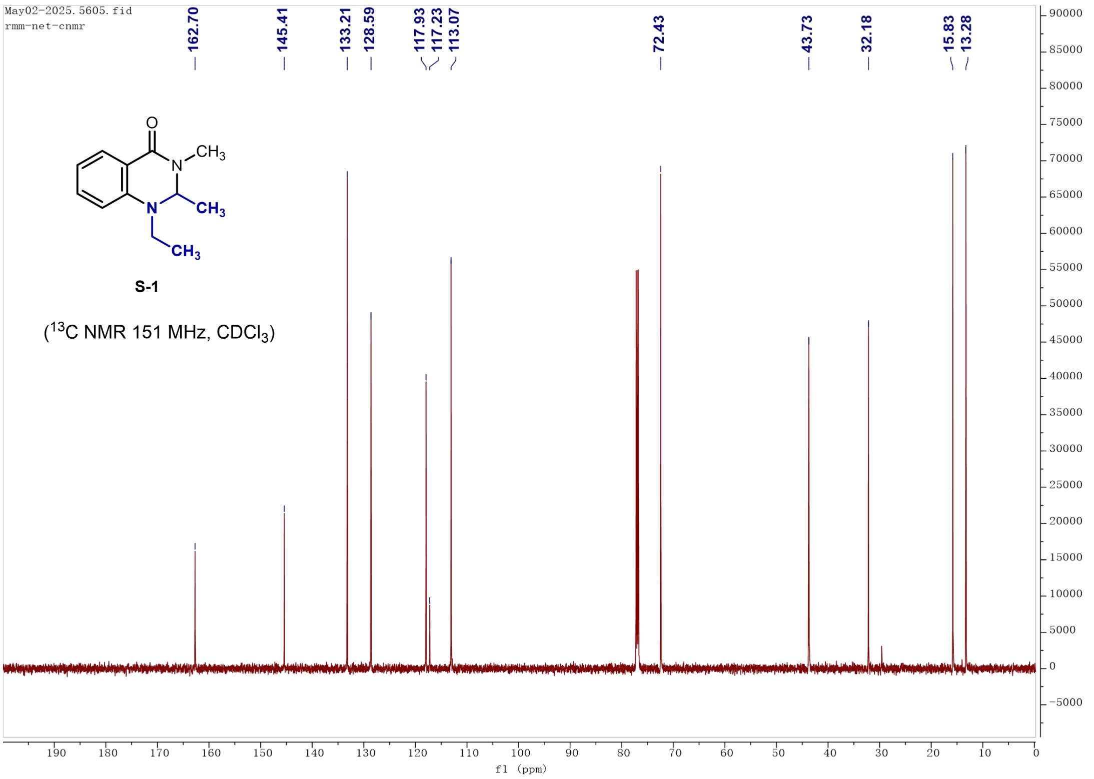
May02-2025.5605.fid
rmm-net-cnmr
162.70
145.41
133.21
128.59
117.93
117.23
113.07
72.43
43.73
32.18
15.83
13.28
S-1
(^{13}C NMR 151 MHz, $CDCl_3$)
f1 (ppm)

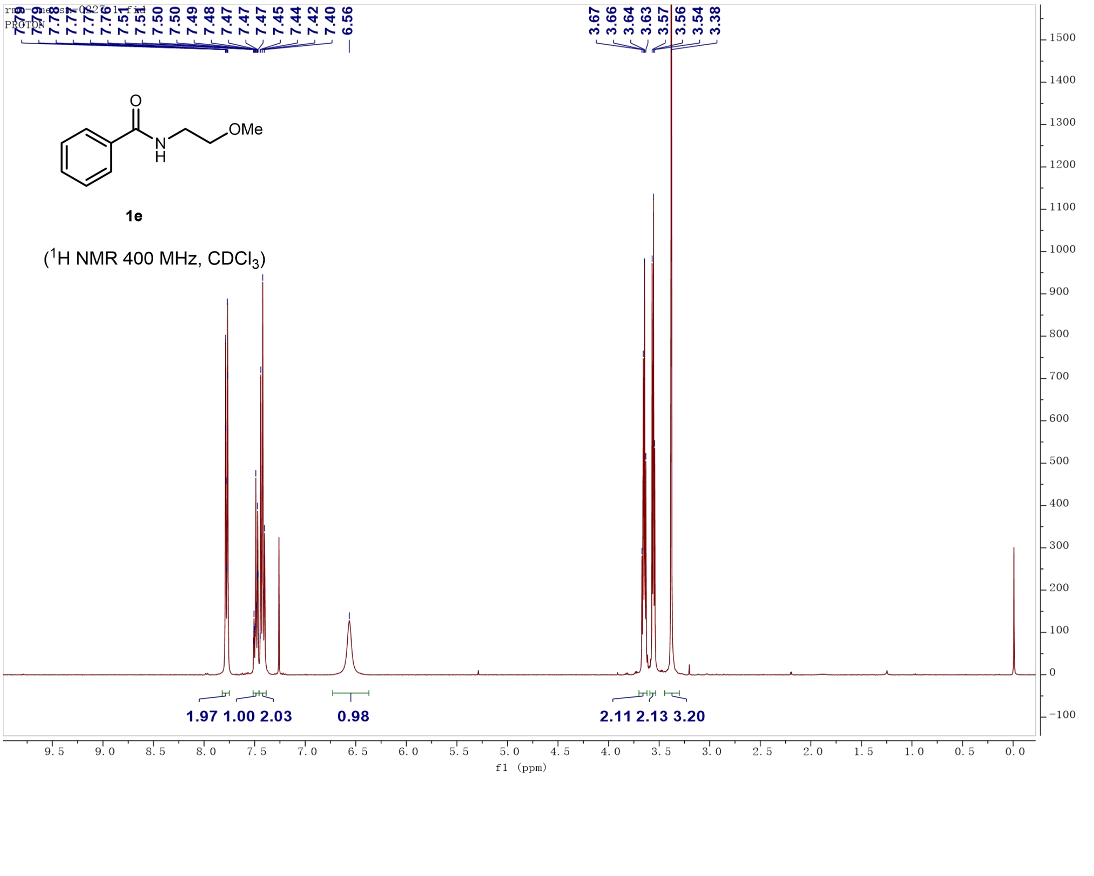
PROTON
7.78
7.77
7.77
7.76
7.51
7.50
7.49
7.48
7.47
7.47
7.47
7.45
7.44
7.42
7.40
6.56
3.67
3.66
3.64
3.63
3.57
3.56
3.54
3.38
OMe
1e
(^{1}H NMR 400 MHz, $CDCl_3$)
1.97 1.00 2.03
0.98
2.11 2.13 3.20
f1 (ppm)

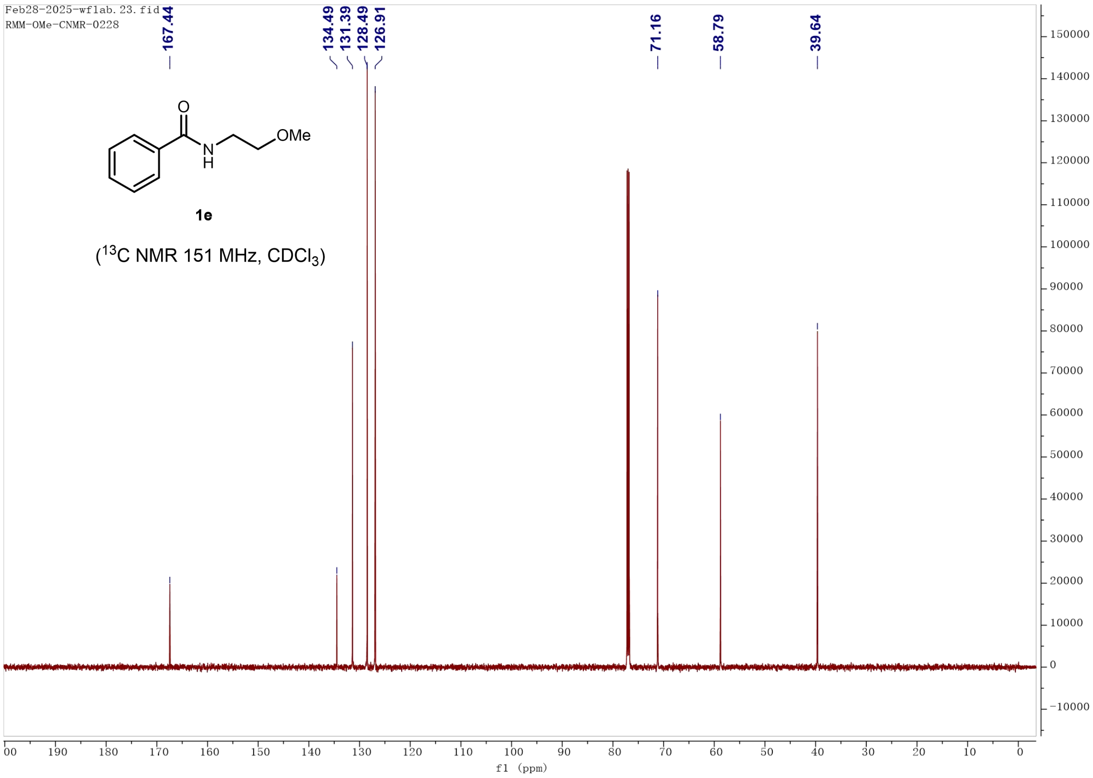
Feb28-2025-wflab.23.fid
RMM-OMe-CNMR-0228
167.44
134.49
131.39
128.49
126.91
71.16
58.79
39.64
OMe
N
H
O
1e
(^{13}C NMR 151 MHz, $CDCl_3$)
f1 (ppm)

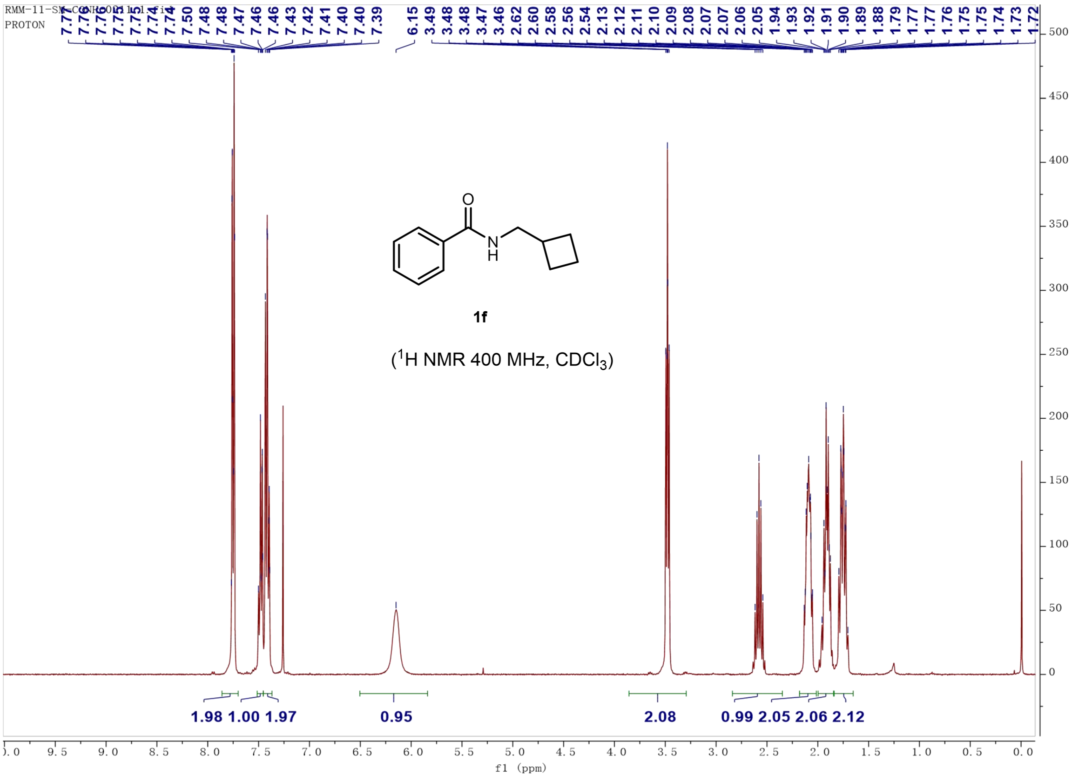
PROTON
7.77
7.76
7.76
7.75
7.74
7.74
7.50
7.48
7.48
7.47
7.46
7.46
7.43
7.42
7.41
7.40
7.39
6.15
3.49
3.48
3.47
3.46
2.62
2.60
2.58
2.56
2.54
2.13
2.12
2.11
2.10
2.09
2.08
2.07
2.07
2.06
2.05
1.94
1.93
1.92
1.91
1.90
1.89
1.88
1.79
1.77
1.77
1.76
1.75
1.75
1.74
1.73
1.72
O
N
H
1f
(^{1}H NMR 400 MHz, $CDCl_3$)
1.98 1.00 1.97
0.95
2.08
0.99 2.05 2.06 2.12
f1 (ppm)

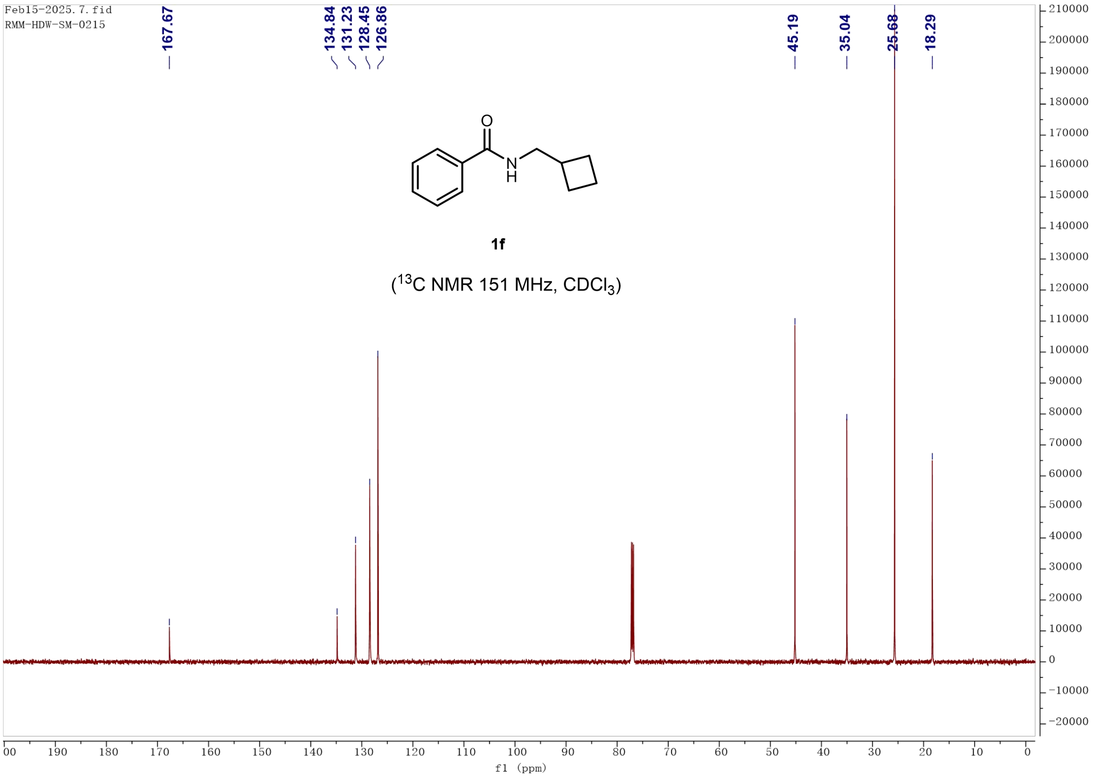
Feb15-2025.7.fid
RMM-HDW-SM-0215
167.67
134.84
131.23
128.45
126.86
45.19
35.04
25.68
18.29
O
N
H
1f
(13C NMR 151 MHz, CDCl3)
f1 (ppm)

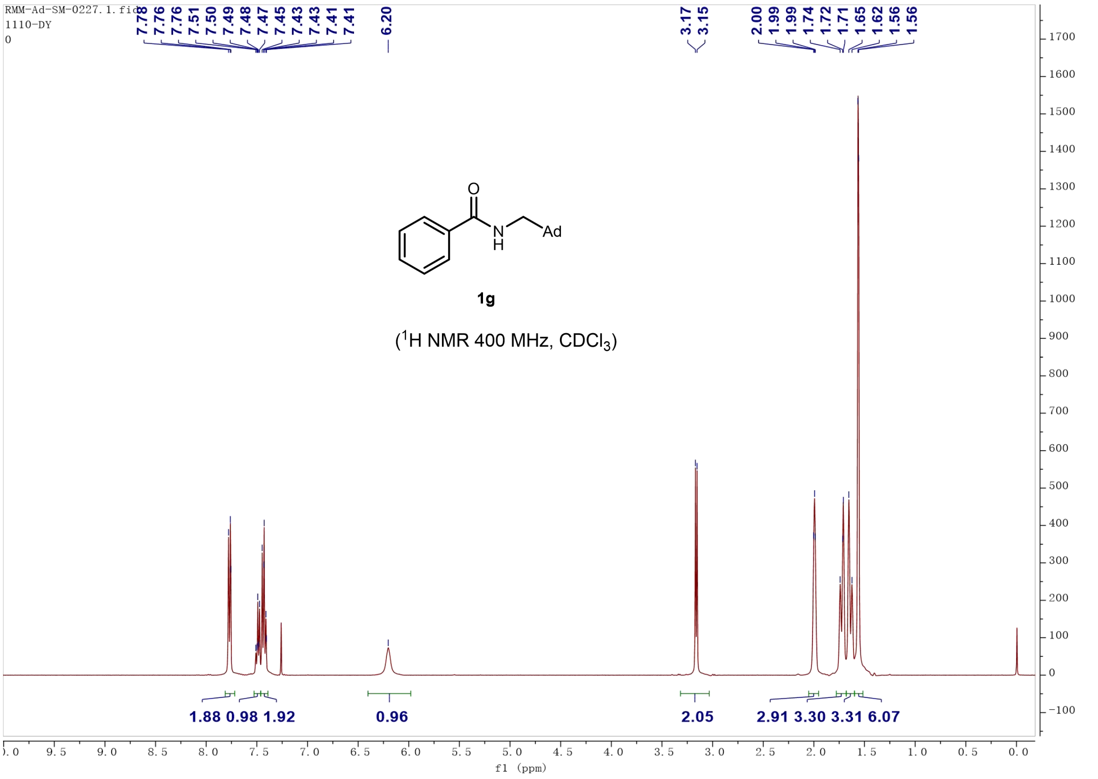
RMM-Ad-SM-0227.1.fid
1110-DY
7.78
7.76
7.76
7.51
7.50
7.49
7.48
7.47
7.45
7.43
7.43
7.41
7.41
6.20
3.17
3.15
2.00
1.99
1.99
1.74
1.72
1.71
1.65
1.62
1.56
1.56
O
N
H
Ad
1g
(1H NMR 400 MHz, CDCl3)
1.88
0.98
1.92
0.96
2.05
2.91
3.30
3.31
6.07
f1 (ppm)

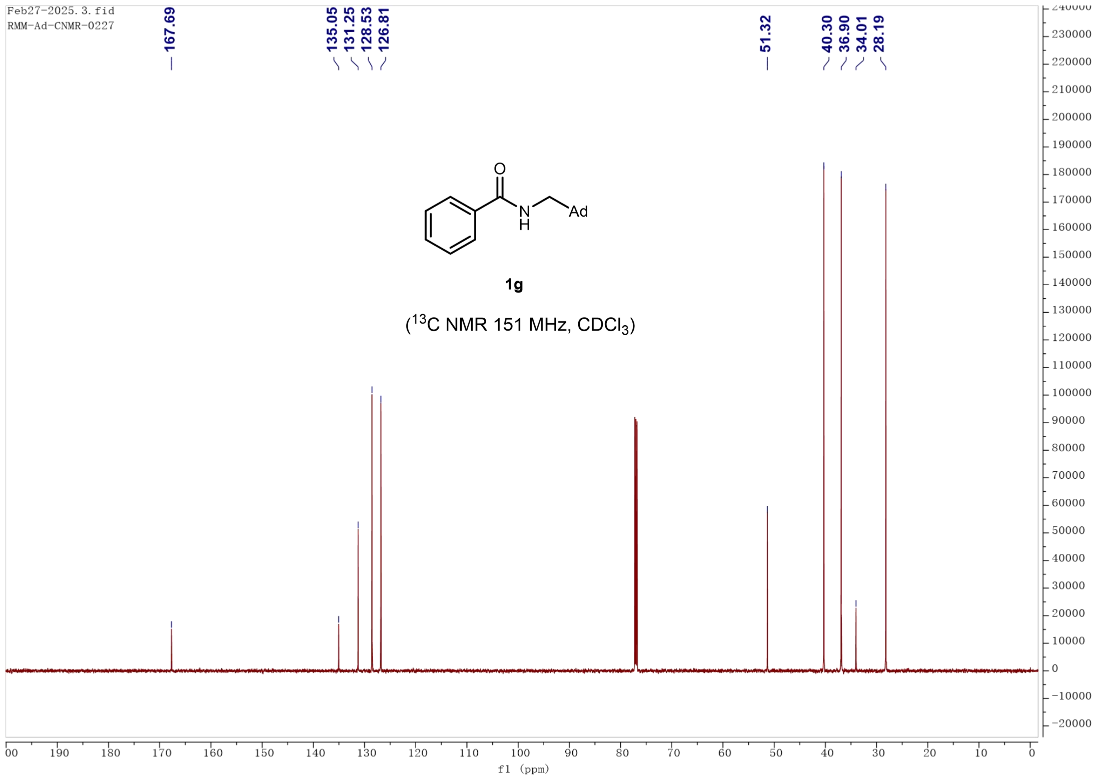

Feb27-2025.3.fid
RMM-Ad-CNMR-0227
167.69
135.05
131.25
128.53
126.81
51.32
40.30
36.90
34.01
28.19
O
N
H
Ad
1g
(^{13}C NMR 151 MHz, $CDCl_3$)
f1 (ppm)

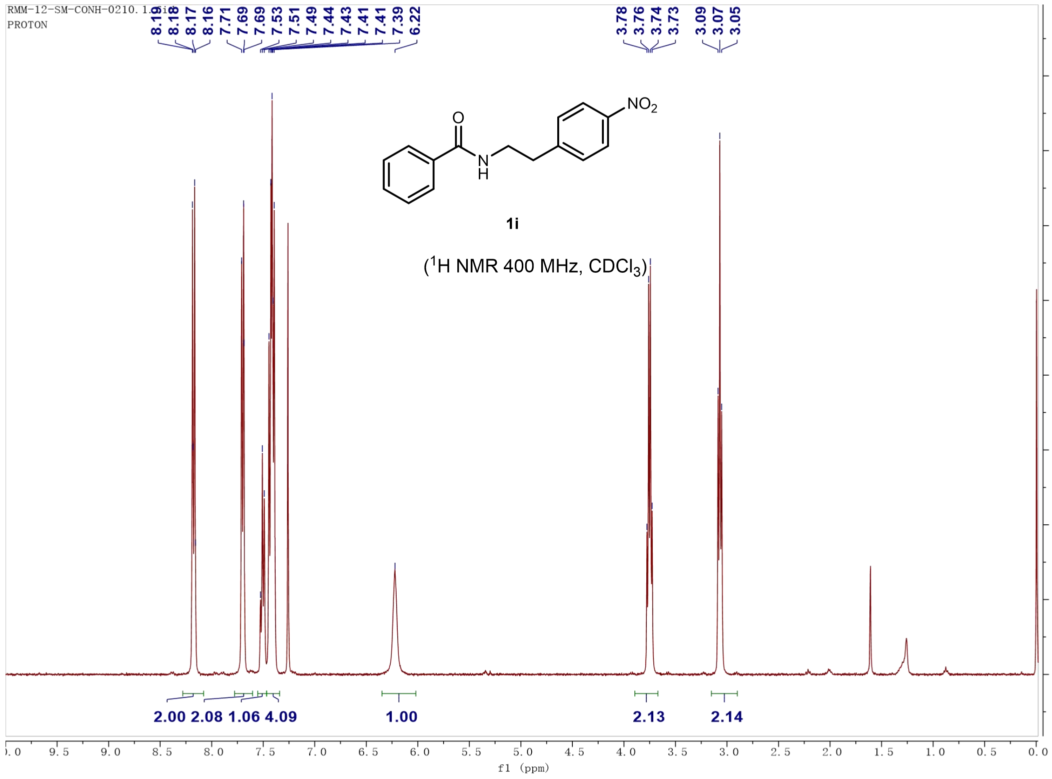

RMM-12-SM-CONH-0210.1.fid
PROTON
8.19
8.18
8.17
8.16
7.71
7.69
7.69
7.53
7.51
7.49
7.44
7.43
7.41
7.41
7.39
6.22
3.78
3.76
3.74
3.73
3.09
3.07
3.05
O
N
H
NO_2
1i
(^{1}H NMR 400 MHz, $CDCl_3$)
2.00
2.08
1.06
4.09
1.00
2.13
2.14
f1 (ppm)

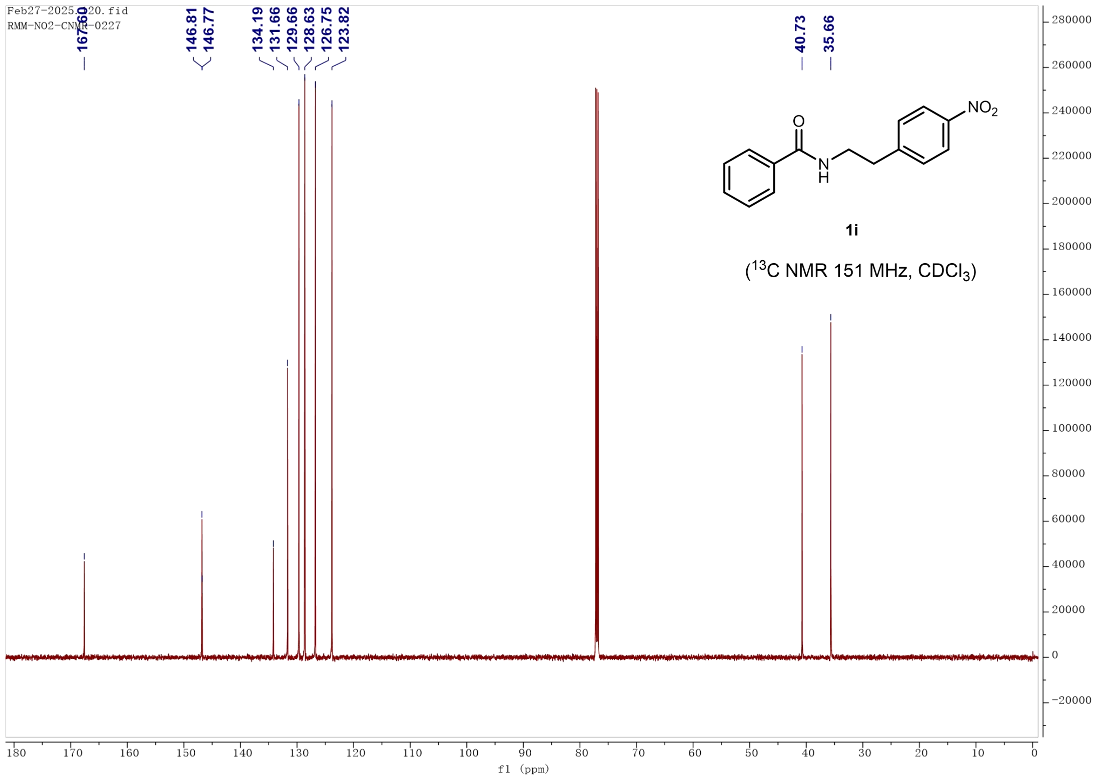
RMM-NO2-CNMR-0227
146.81
146.77
134.19
131.66
129.66
128.63
126.75
123.82
40.73
35.66
NO2
O
N
H
1i
(13C NMR 151 MHz, CDCl3)
f1 (ppm)

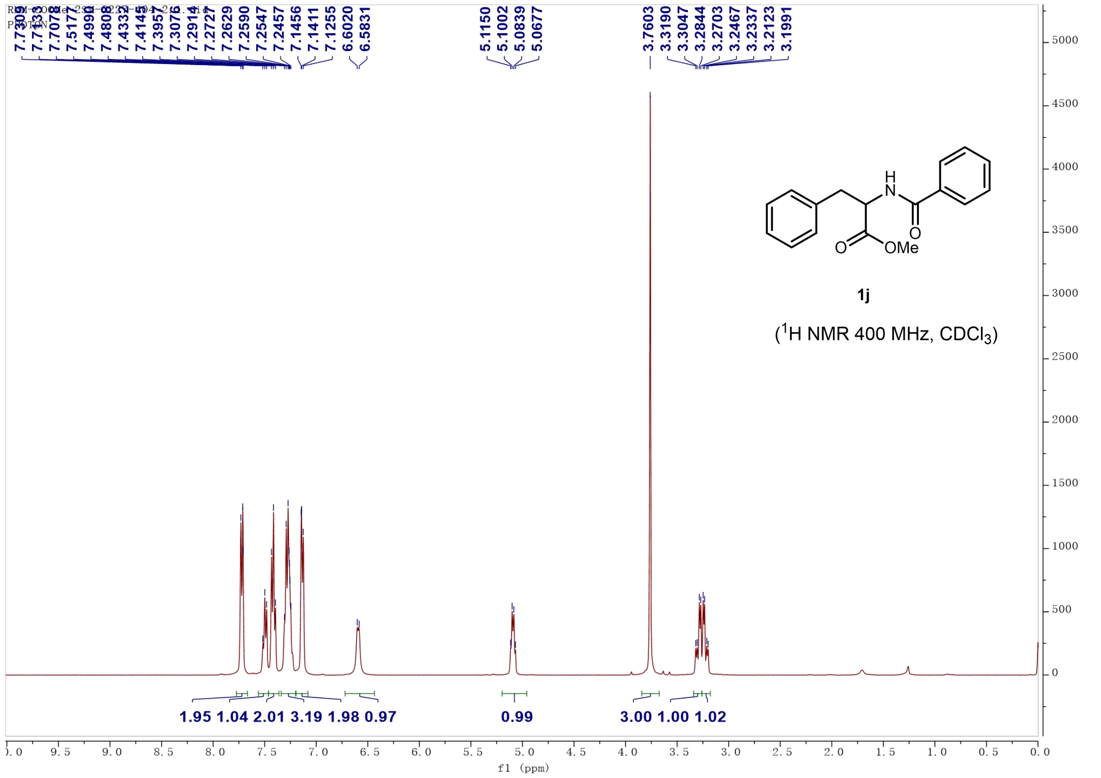
7.5177
7.4990
7.4808
7.4333
7.4145
7.3957
7.3076
7.2914
7.2727
7.2629
7.2590
7.2547
7.2457
7.1456
7.1411
7.1255
6.6020
6.5831
5.1150
5.1002
5.0839
5.0677
3.7603
3.3190
3.3047
3.2844
3.2703
3.2467
3.2337
3.2123
3.1991
H
N
O
O
OMe
1j
(1H NMR 400 MHz, CDCl3)
1.95 1.04 2.01 3.19 1.98 0.97
0.99
3.00 1.00 1.02
f1 (ppm)

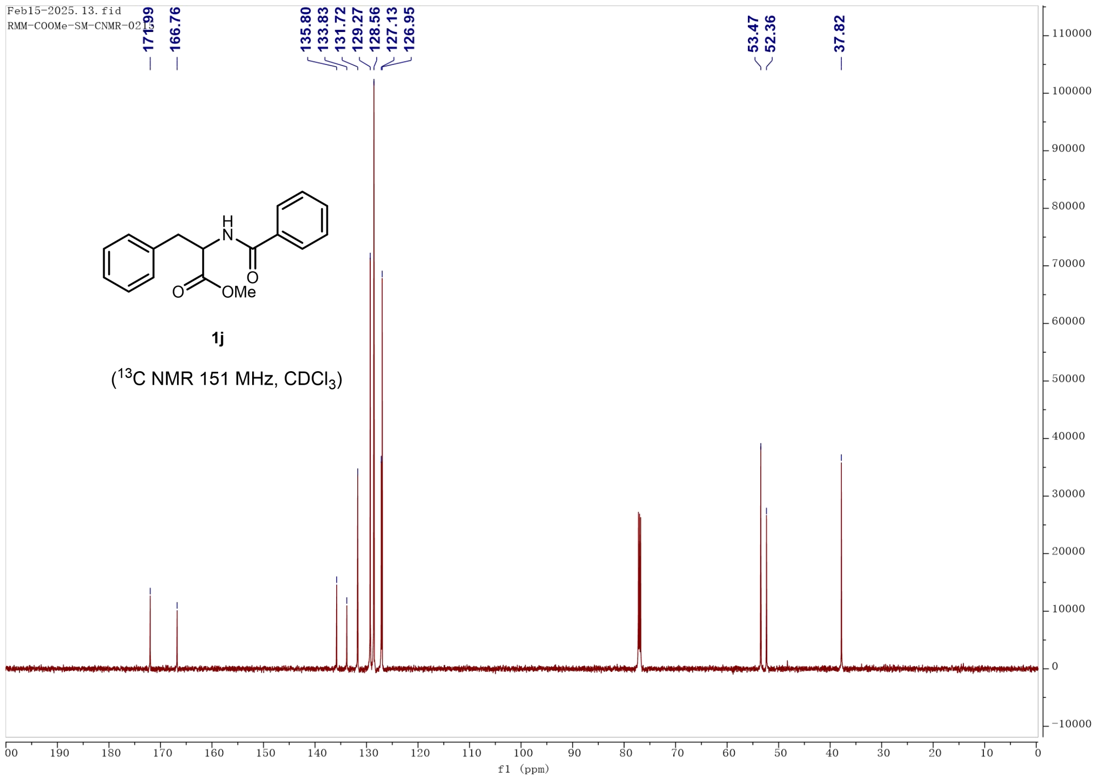
Feb15-2025.13.fid
RMM-COOMe-SM-CNMR-02
171.99
166.76
135.80
133.83
131.72
129.27
128.56
127.13
126.95
53.47
52.36
37.82
1j
(13C NMR 151 MHz, CDCl3)
f1 (ppm)

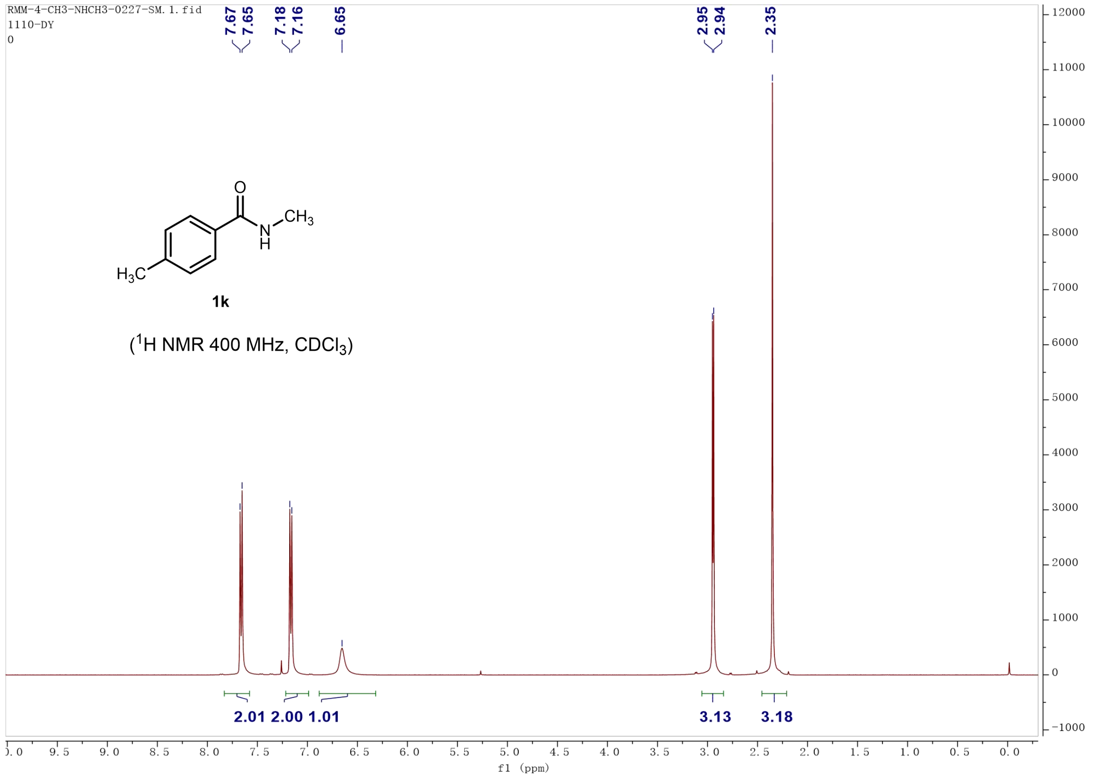
RMM-4-CH3-NHCH3-0227-SM.1.fid
1110-DY
0
7.67
7.65
7.18
7.16
6.65
2.95
2.94
2.35
1k
(1H NMR 400 MHz, CDCl3)
2.01 2.00 1.01
3.13
3.18
f1 (ppm)

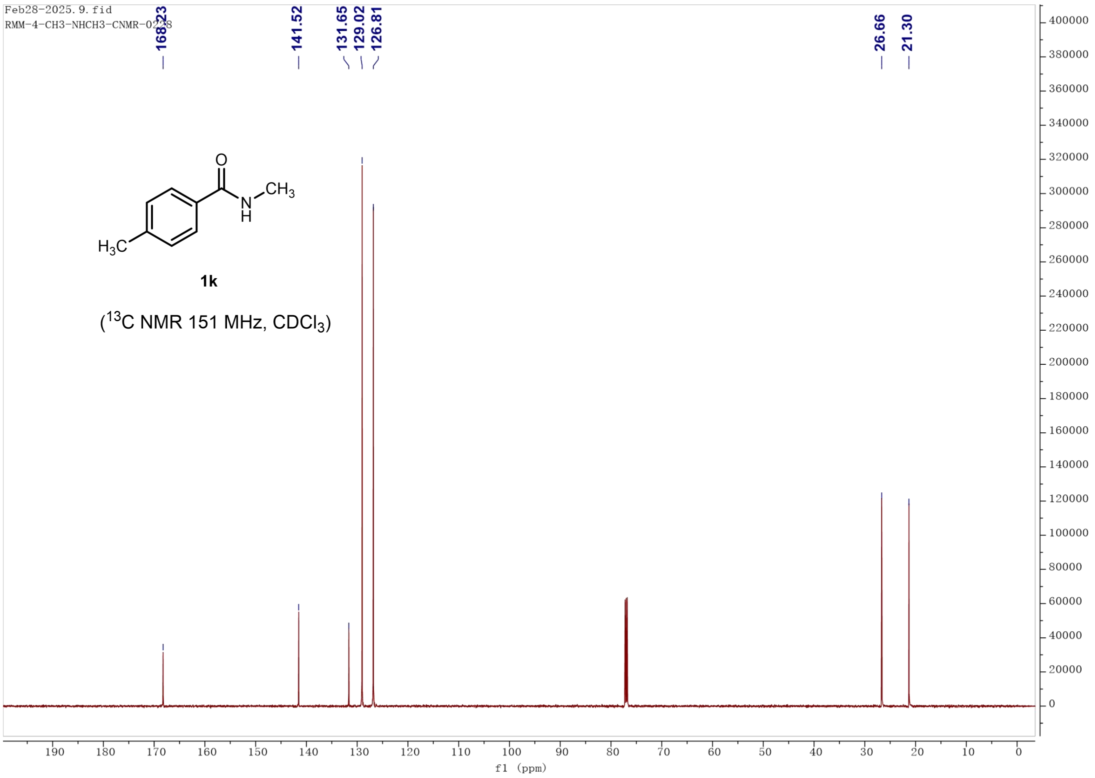
Feb28-2025.9.fid
168.23
141.52
131.65
129.02
126.81
26.66
21.30
1k
(13C NMR 151 MHz, CDCl3)
f1 (ppm)

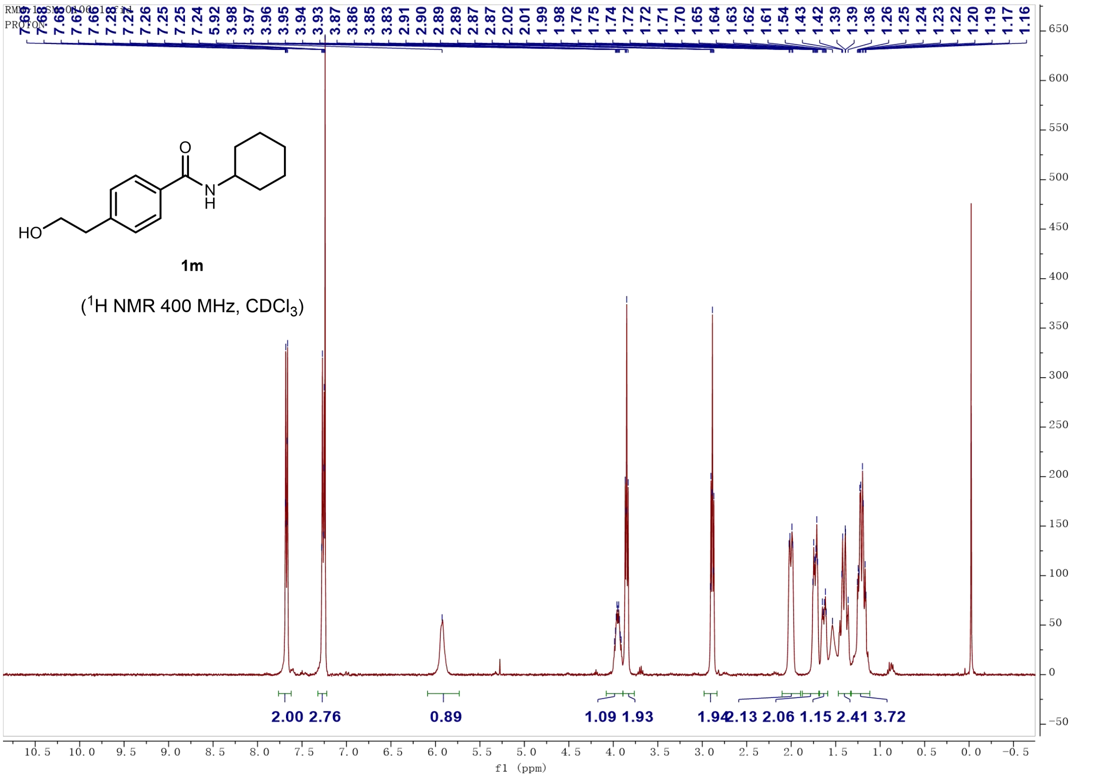
PROTON
7.68 7.67 7.66 7.28 7.26 7.26 7.25 7.25 7.24 5.92 3.98 3.97 3.96 3.95 3.94 3.93 3.87 3.86 3.85 3.83 2.91 2.90 2.89 2.89 2.87 2.02 2.01 1.99 1.98 1.76 1.75 1.74 1.72 1.72 1.71 1.70 1.65 1.64 1.63 1.62 1.61 1.54 1.43 1.42 1.39 1.39 1.36 1.26 1.25 1.24 1.23 1.22 1.20 1.19 1.17 1.16
1m
(1H NMR 400 MHz, CDCl3)
2.00 2.76 0.89 1.09 1.93 1.94 2.13 2.06 1.15 2.41 3.72
f1 (ppm)

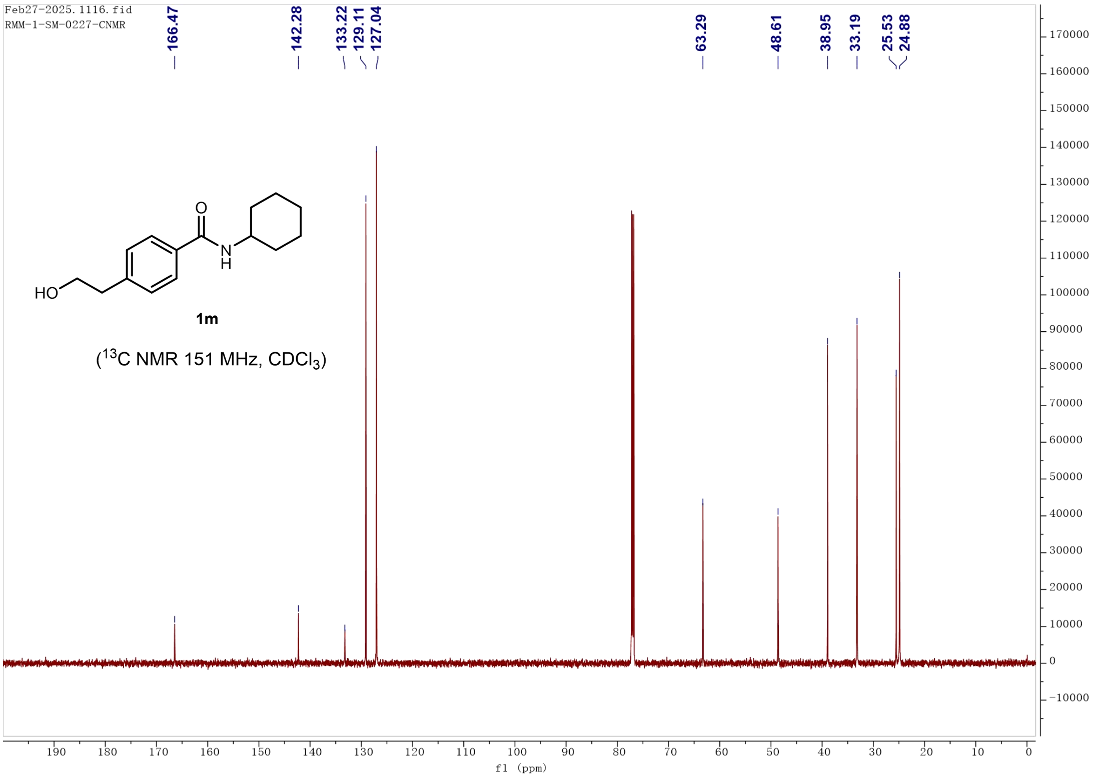

1m

(^{13}C NMR 151 MHz, $CDCl_3$)

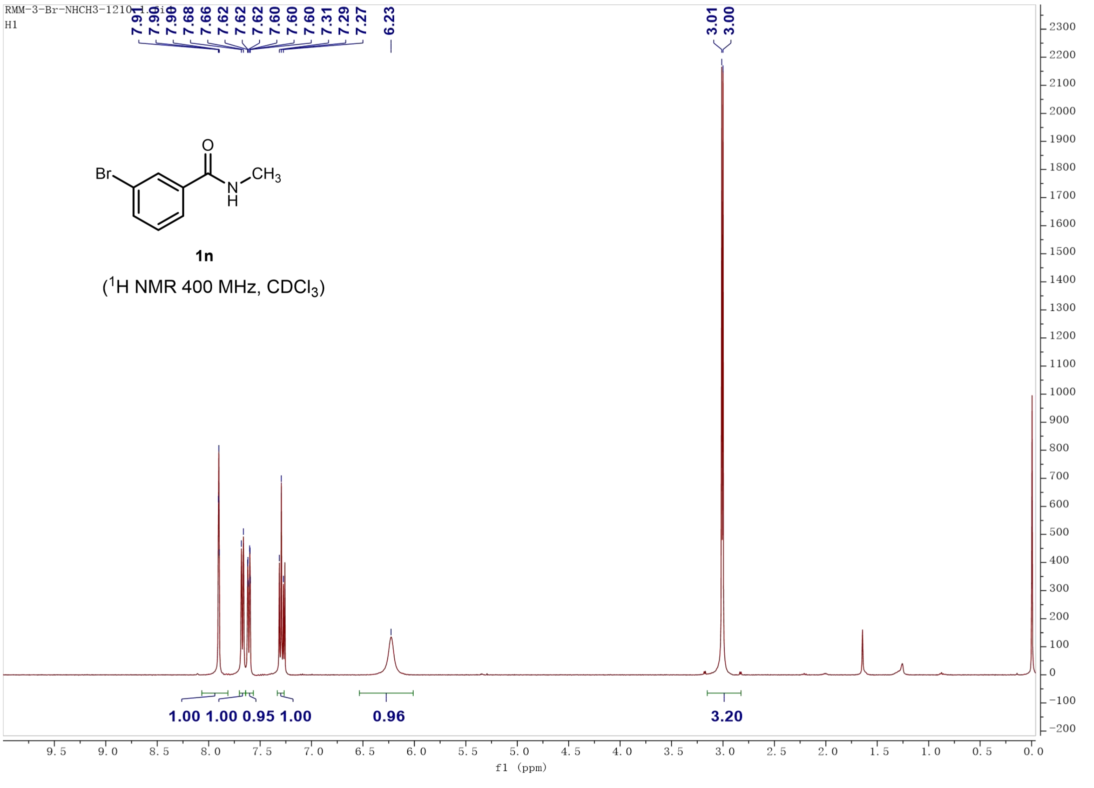

1n

(^{1}H NMR 400 MHz, $CDCl_3$)

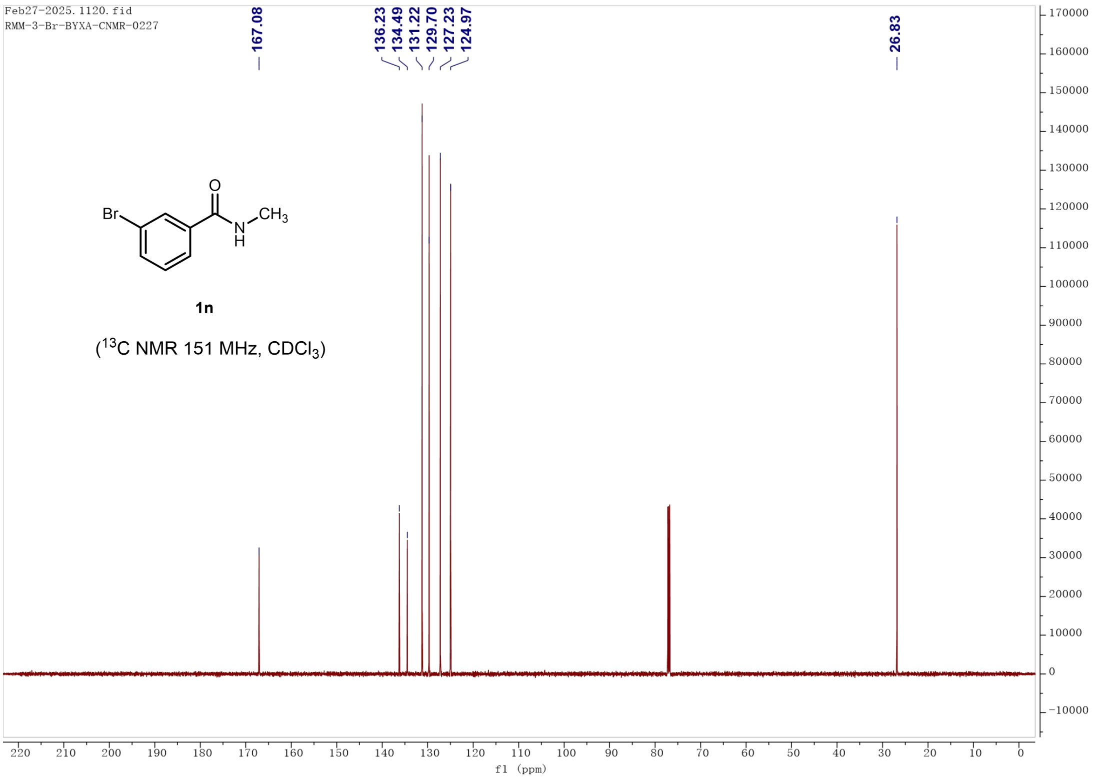
Feb27-2025.1120.fid
RMM-3-Br-BYXA-CNMR-0227
167.08
136.23
134.49
131.22
129.70
127.23
124.97
26.83
Br
O
N
H
CH3
1n
(13C NMR 151 MHz, CDCl3)
f1 (ppm)

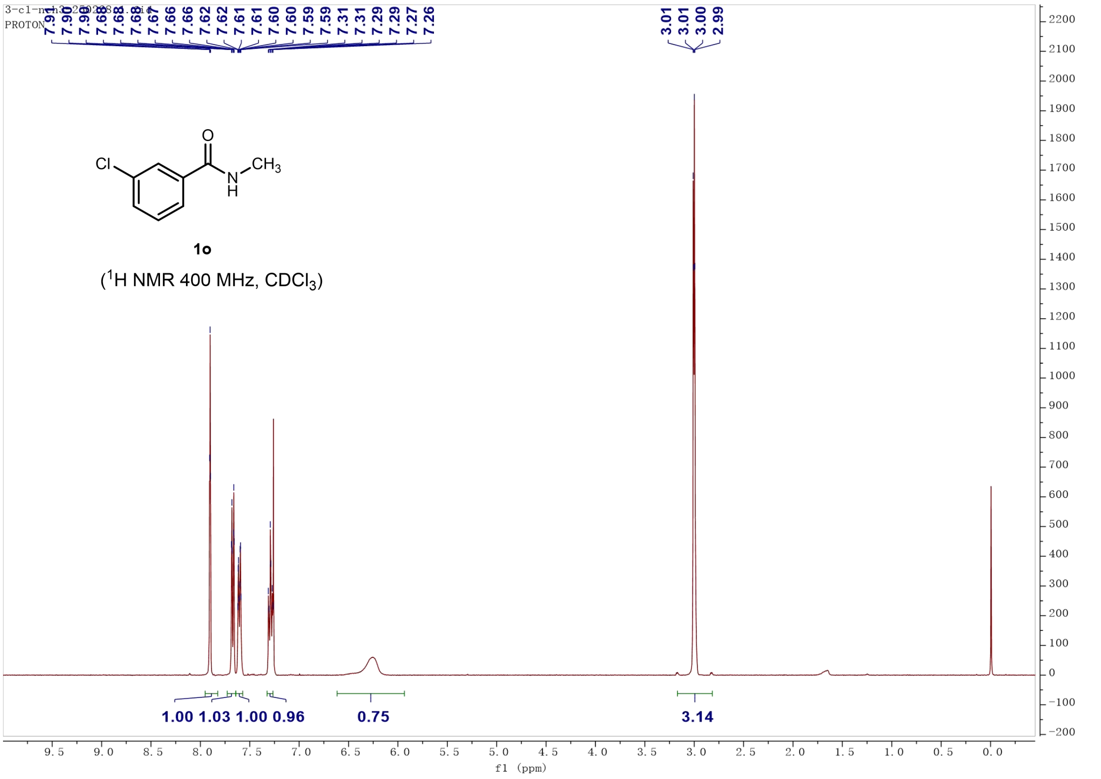
PROTON
7.91
7.90
7.68
7.68
7.68
7.67
7.66
7.62
7.62
7.61
7.61
7.60
7.59
7.59
7.31
7.29
7.29
7.27
7.26
3.01
3.01
3.00
2.99
Cl
O
N
H
CH3
1o
(1H NMR 400 MHz, CDCl3)
1.00
1.03
1.00
0.96
0.75
3.14
f1 (ppm)

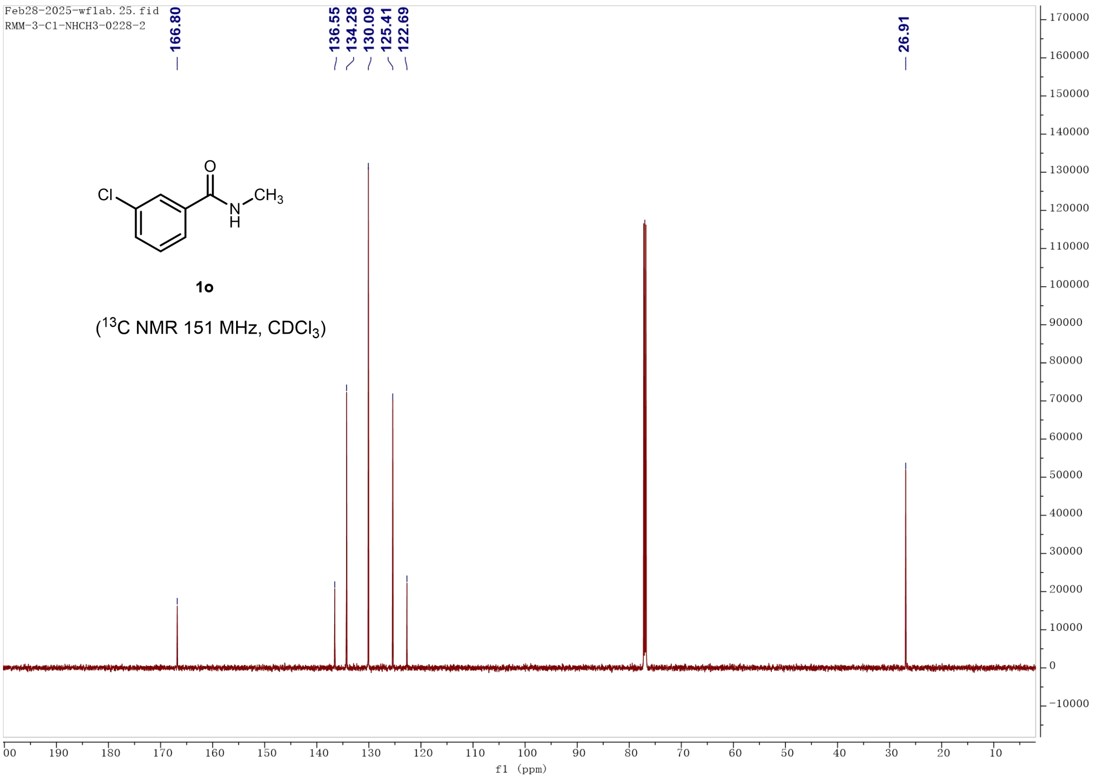
Feb28-2025-wflab.25.fid
RMM-3-Cl-NHCH3-0228-2
166.80
136.55
134.28
130.09
125.41
122.69
26.91
1o
(13C NMR 151 MHz, CDCl3)
f1 (ppm)

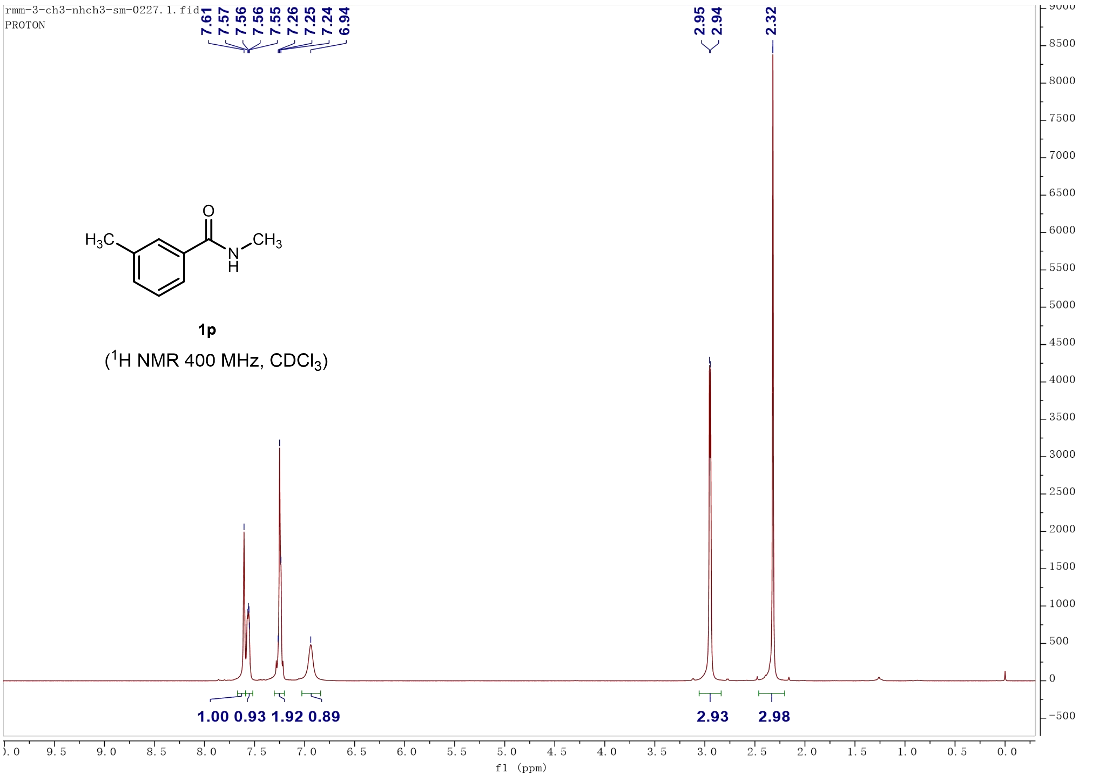
rmm-3-ch3-nhch3-sm-0227.1.fid
PROTON
7.61
7.57
7.56
7.56
7.55
7.26
7.25
7.24
6.94
2.95
2.94
2.32
1p
(1H NMR 400 MHz, CDCl3)
1.00 0.93 1.92 0.89
2.93
2.98
f1 (ppm)

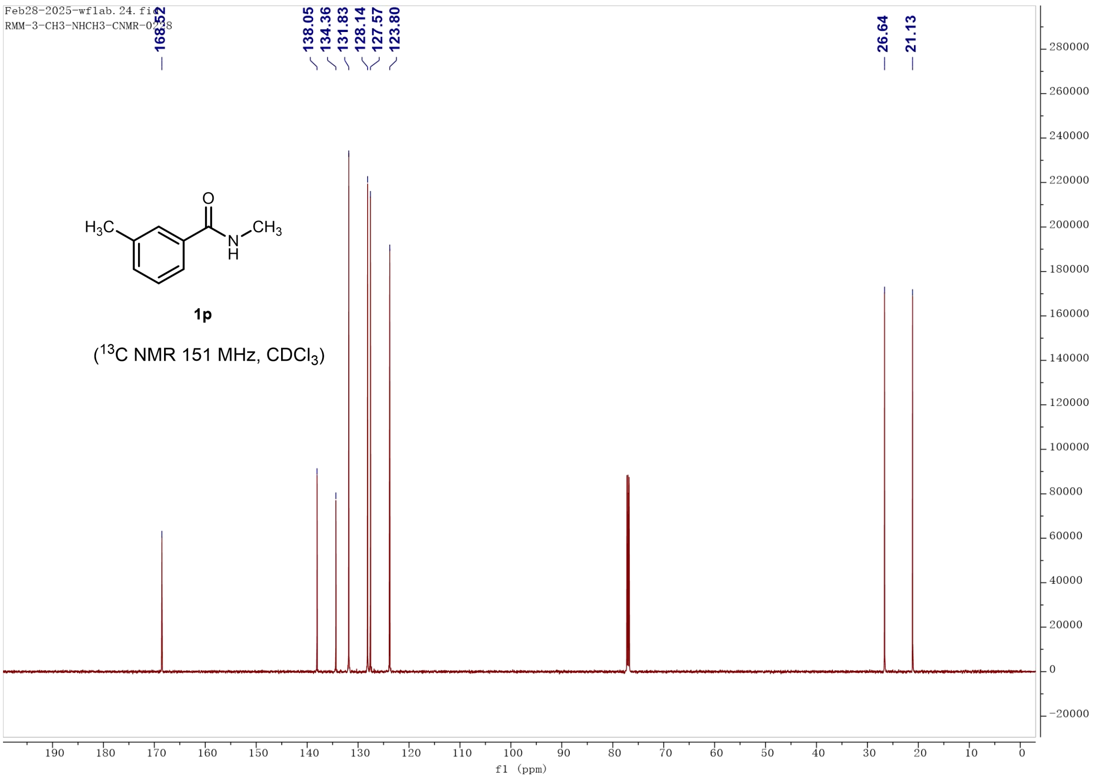

(^{13}C NMR 151 MHz, $CDCl_3$)

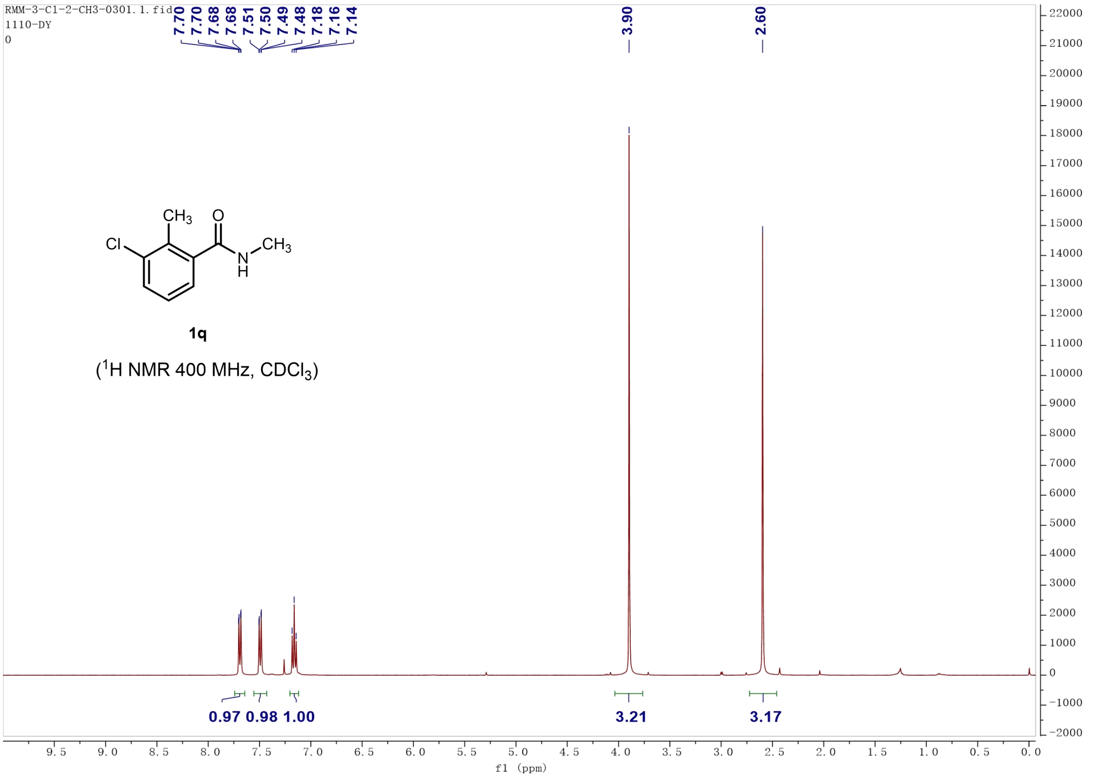

(^{1}H NMR 400 MHz, $CDCl_3$)

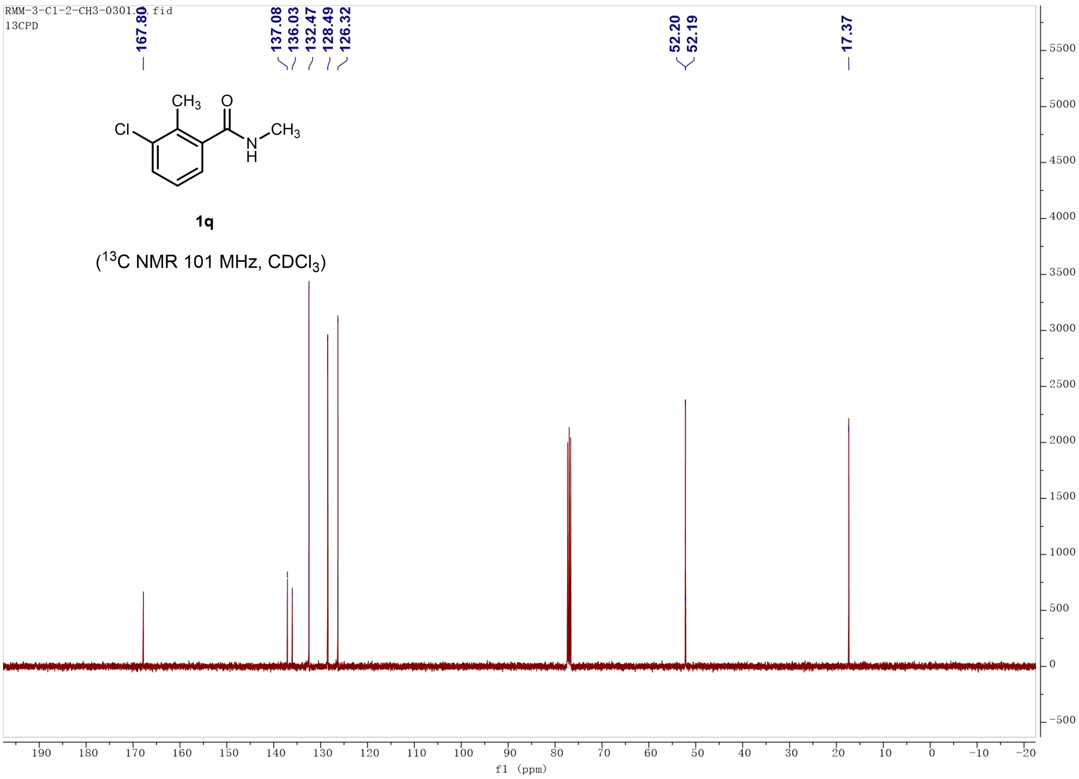
RMM-3-Cl-2-CH3-0301.1.fid
13CPD
167.80
137.08
136.03
132.47
128.49
126.32
52.20
52.19
17.37
1q
(13C NMR 101 MHz, CDCl3)
f1 (ppm)

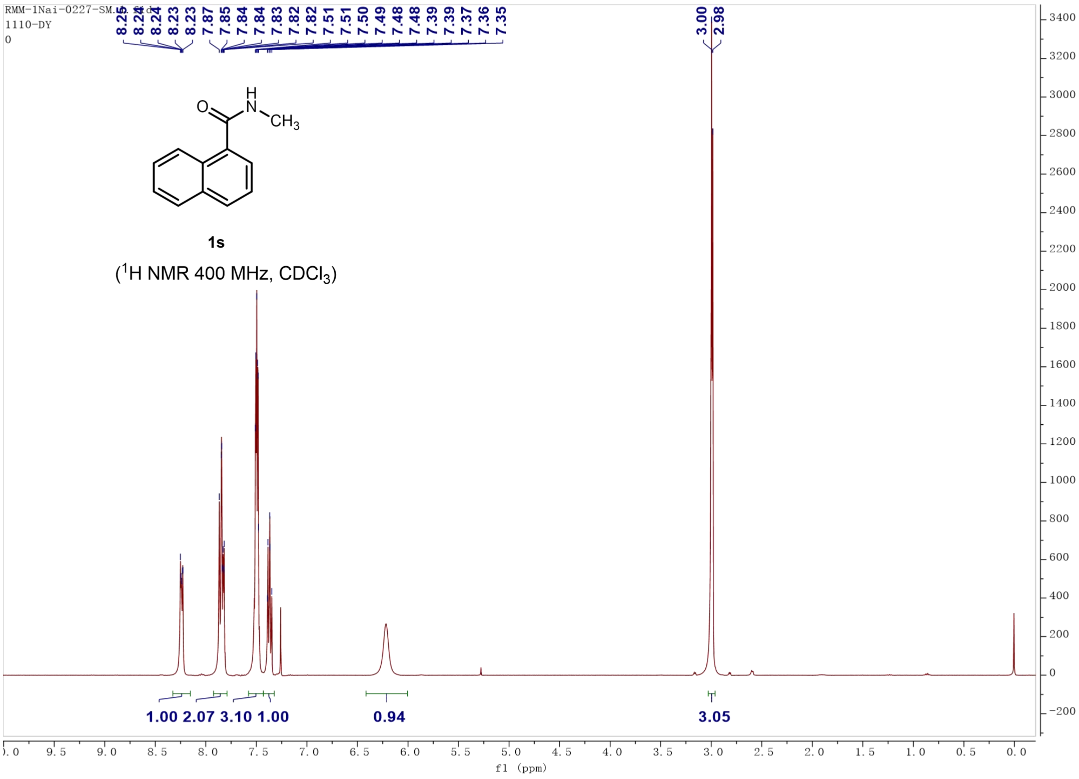
1110-DY
8.25
8.24
8.24
8.23
8.23
7.87
7.85
7.84
7.84
7.83
7.82
7.82
7.51
7.50
7.49
7.48
7.48
7.39
7.39
7.37
7.36
7.35
3.00
2.98
1s
(1H NMR 400 MHz, CDCl3)
1.00
2.07
3.10
1.00
0.94
3.05
f1 (ppm)

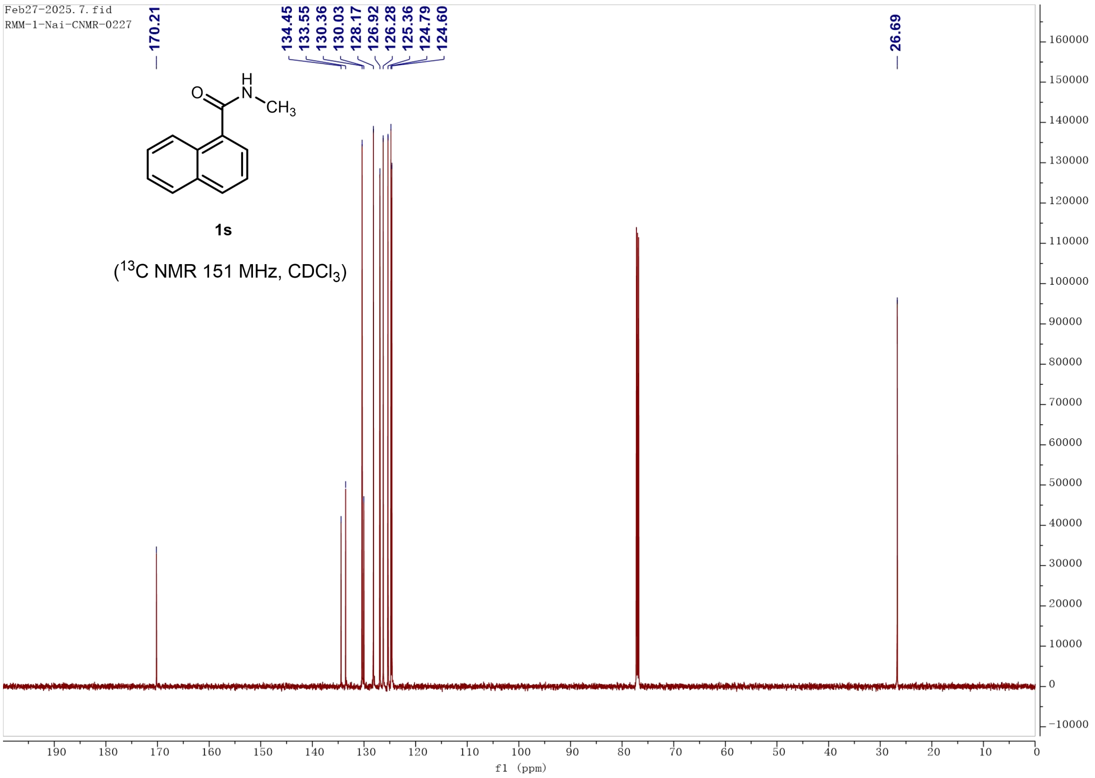
Feb27-2025.7.fid
RMM-1-Nai-CNMR-0227
170.21
134.45
133.55
130.36
130.03
128.17
126.92
126.28
125.36
124.79
124.60
26.69
1s
(^{13}C NMR 151 MHz, CDCl$_3$)
f1 (ppm)

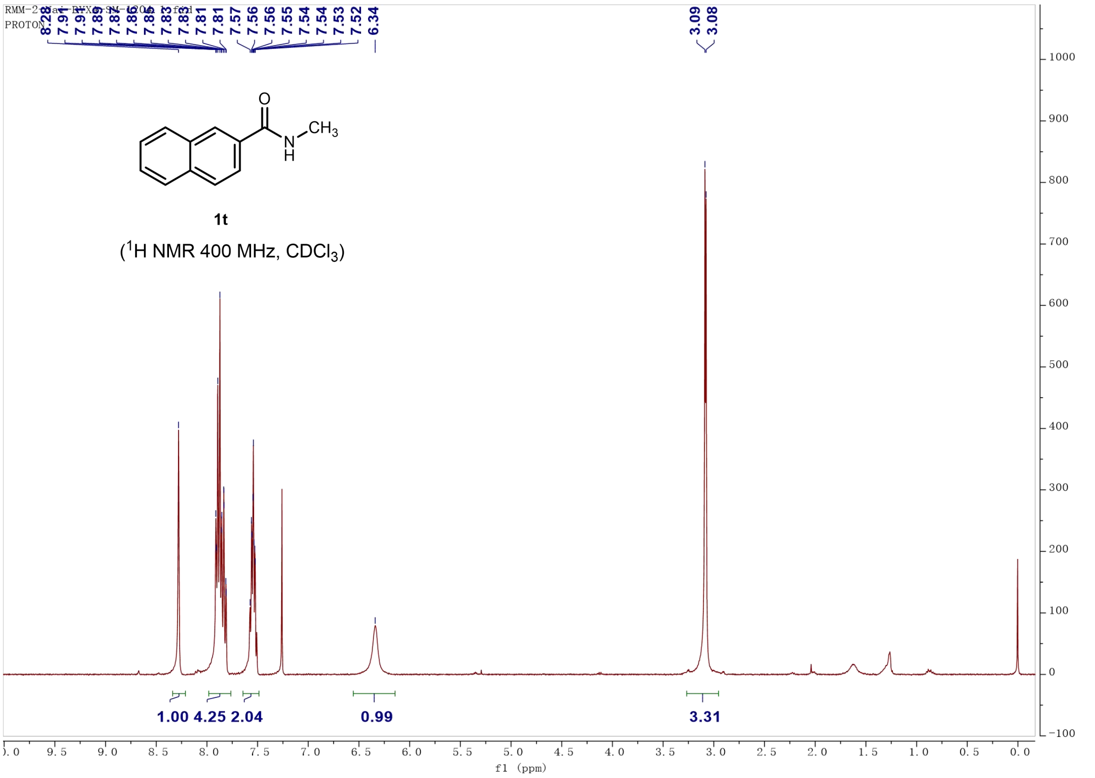
PROTON
8.28
7.91
7.89
7.87
7.86
7.85
7.83
7.82
7.81
7.57
7.56
7.55
7.54
7.54
7.53
7.52
6.34
3.09
3.08
1t
(^{1}H NMR 400 MHz, CDCl$_3$)
1.00
4.25
2.04
0.99
3.31
f1 (ppm)

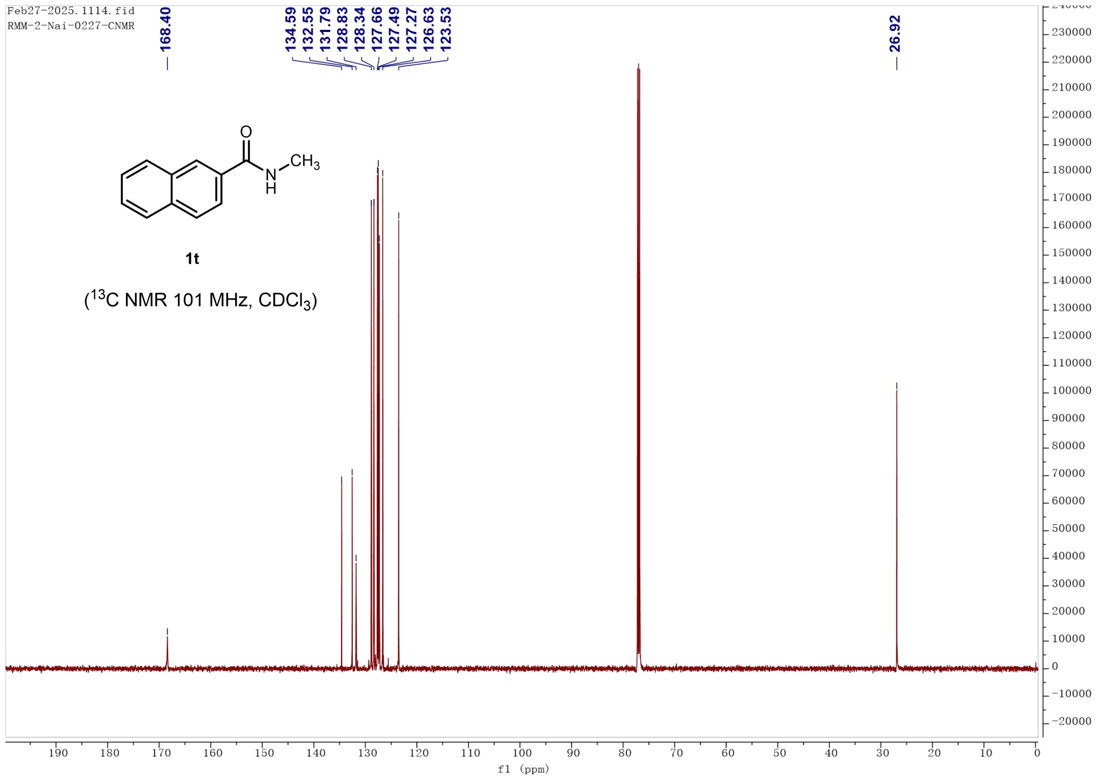
Feb27-2025.1114.fid
RMM-2-Nai-0227-CNMR
168.40
134.59
132.55
131.79
128.83
128.34
127.66
127.49
127.27
126.63
123.53
26.92
1t
(13C NMR 101 MHz, CDCl3)
f1 (ppm)

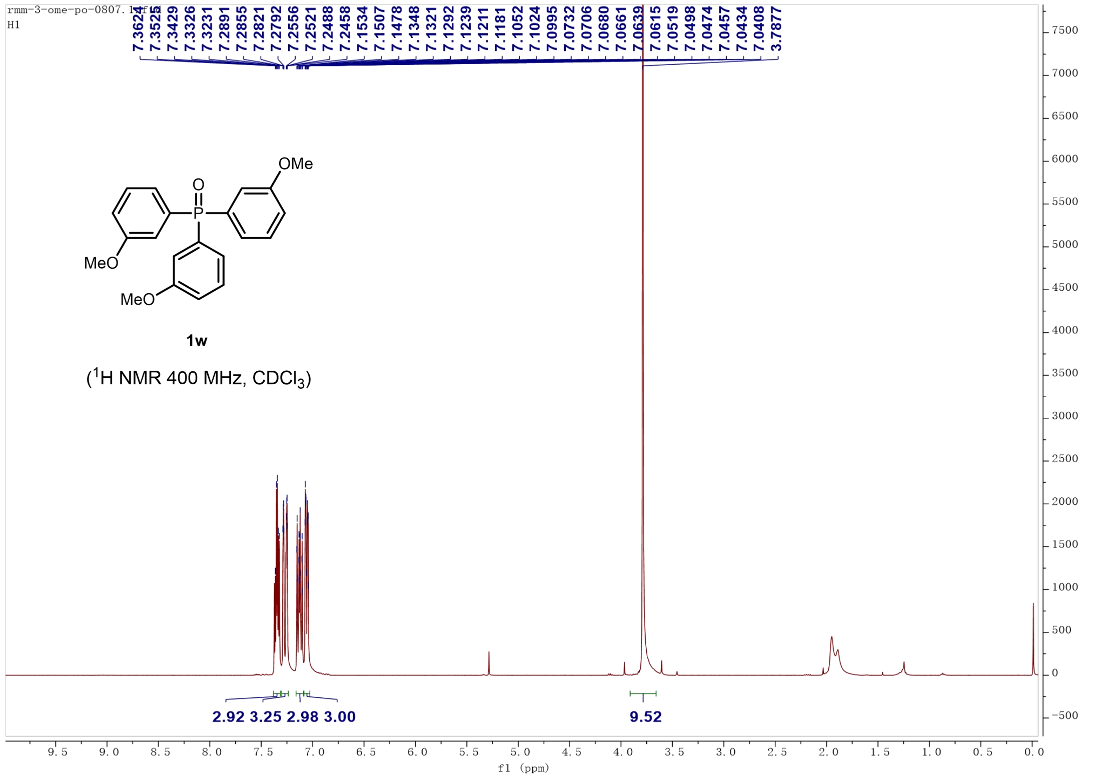
H1
7.3624
7.3525
7.3429
7.3326
7.3231
7.2891
7.2855
7.2821
7.2792
7.2556
7.2521
7.2488
7.2458
7.1534
7.1507
7.1478
7.1348
7.1321
7.1292
7.1239
7.1211
7.1181
7.1052
7.1024
7.0995
7.0732
7.0706
7.0680
7.0661
7.0639
7.0615
7.0519
7.0498
7.0474
7.0457
7.0434
7.0408
3.7877
OMe
MeO
MeO
1w
(1H NMR 400 MHz, CDCl3)
2.92 3.25 2.98 3.00
9.52
f1 (ppm)

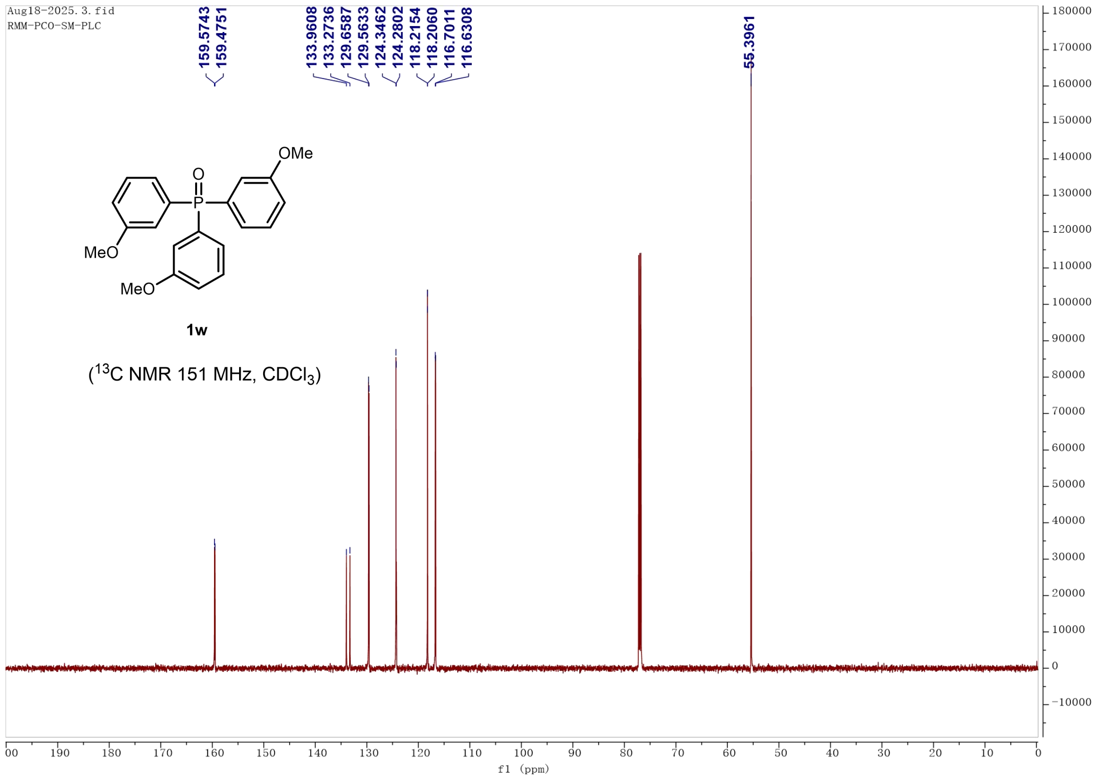
Aug18-2025.3.fid
RMM-PCO-SM-PLC
159.5743
159.4751
133.9608
133.2736
129.6587
129.5633
124.3462
124.2802
118.2154
118.2060
116.7011
116.6308
55.3961
OMe
O
P
MeO
MeO
1w
(^{13}C NMR 151 MHz, $CDCl_3$)
f1 (ppm)

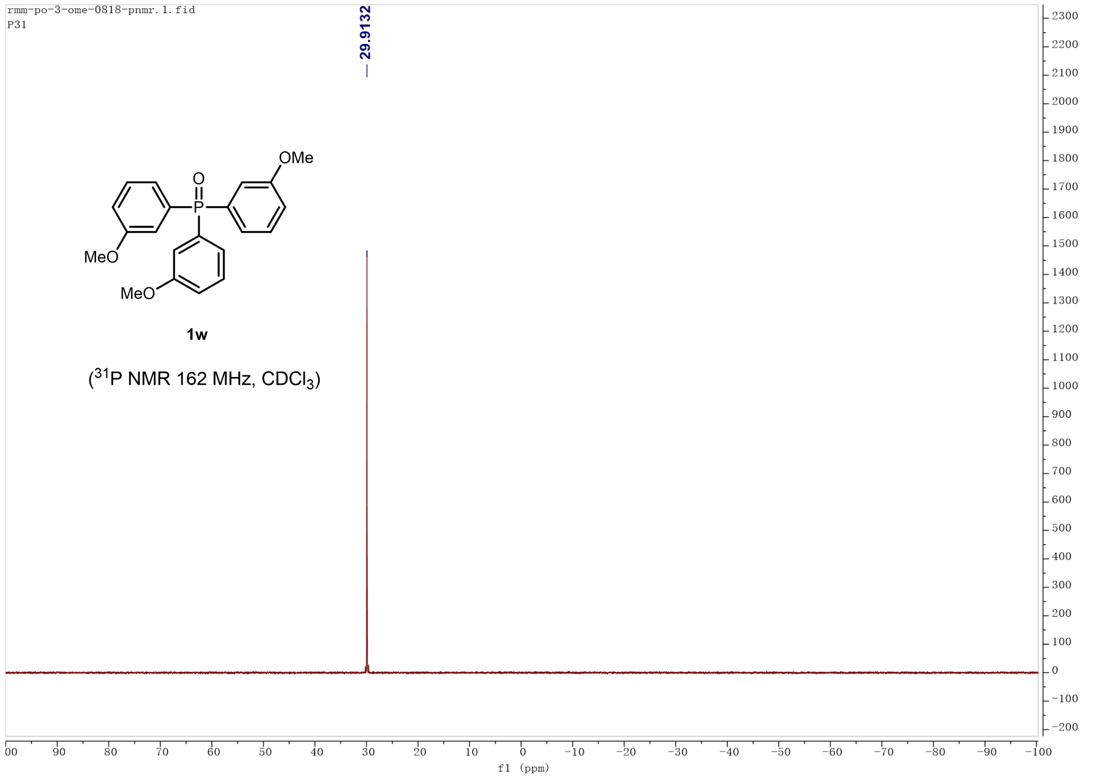
rmm-po-3-ome-0818-pnmr.1.fid
P31
29.9132
OMe
O
P
MeO
MeO
1w
(^{31}P NMR 162 MHz, $CDCl_3$)
f1 (ppm)

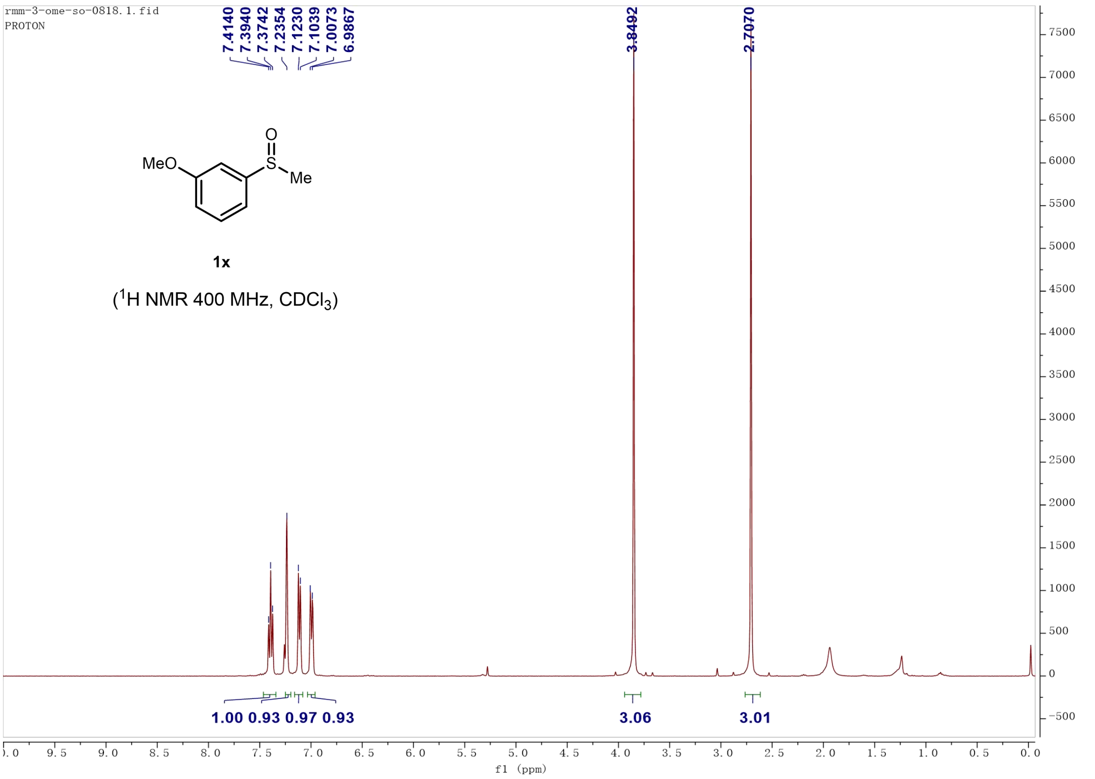
rmm-3-ome-so-0818.1.fid
PROTON
7.4140 7.3940 7.3742 7.2354 7.1230 7.1039 7.0073 6.9867
3.8492
2.7070
MeO
O
S
Me
1x
(^{1}H NMR 400 MHz, CDCl$_3$)
1.00 0.93 0.97 0.93
3.06
3.01
f1 (ppm)

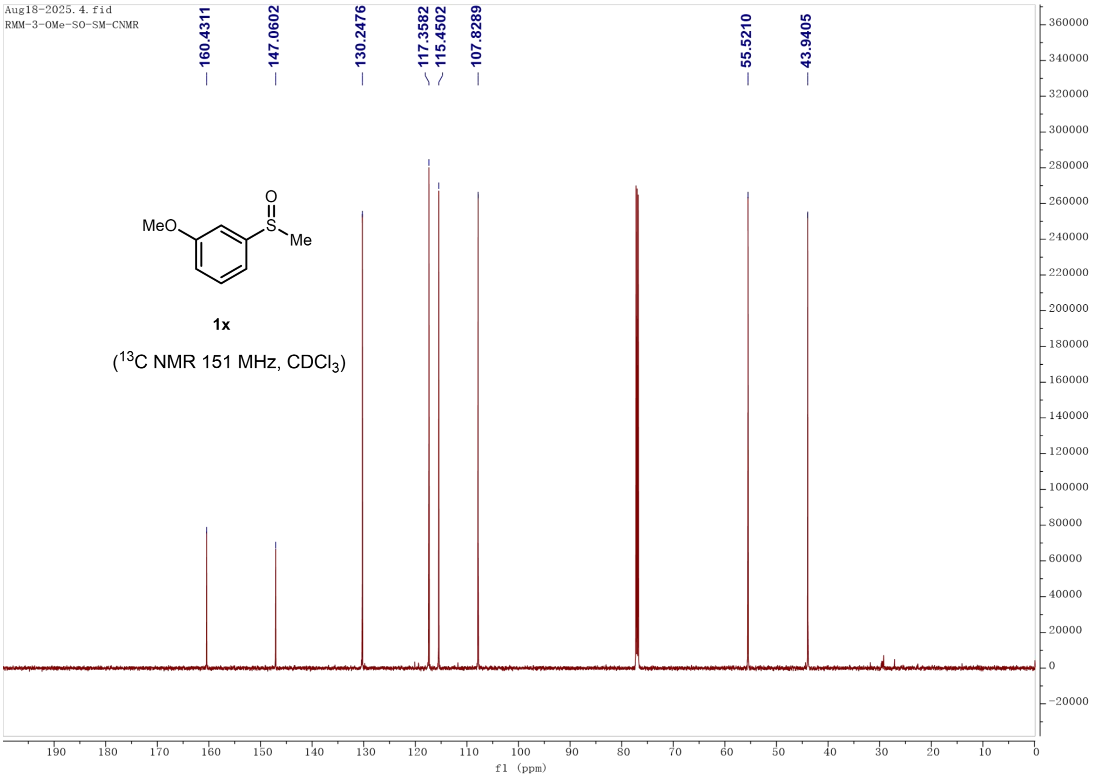
Aug18-2025.4.fid
RMM-3-OMe-SO-SM-CNMR
160.4311
147.0602
130.2476
117.3582
115.4502
107.8289
55.5210
43.9405
MeO
O
S
Me
1x
(^{13}C NMR 151 MHz, CDCl$_3$)
f1 (ppm)

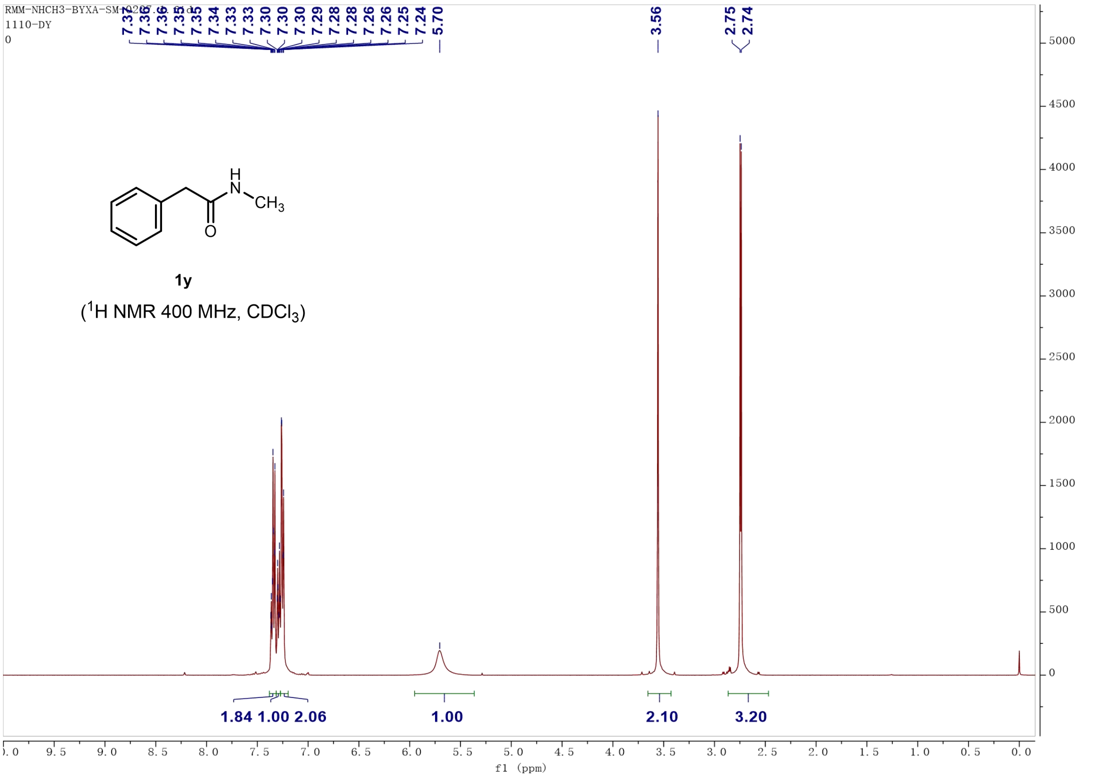
7.37 7.36 7.36 7.35 7.35 7.34 7.33 7.33 7.30 7.30 7.30 7.29 7.28 7.28 7.26 7.26 7.25 7.24
5.70
3.56
2.75 2.74
1y
(^{1}H NMR 400 MHz, $CDCl_3$)
1.84 1.00 2.06
1.00
2.10
3.20
f1 (ppm)

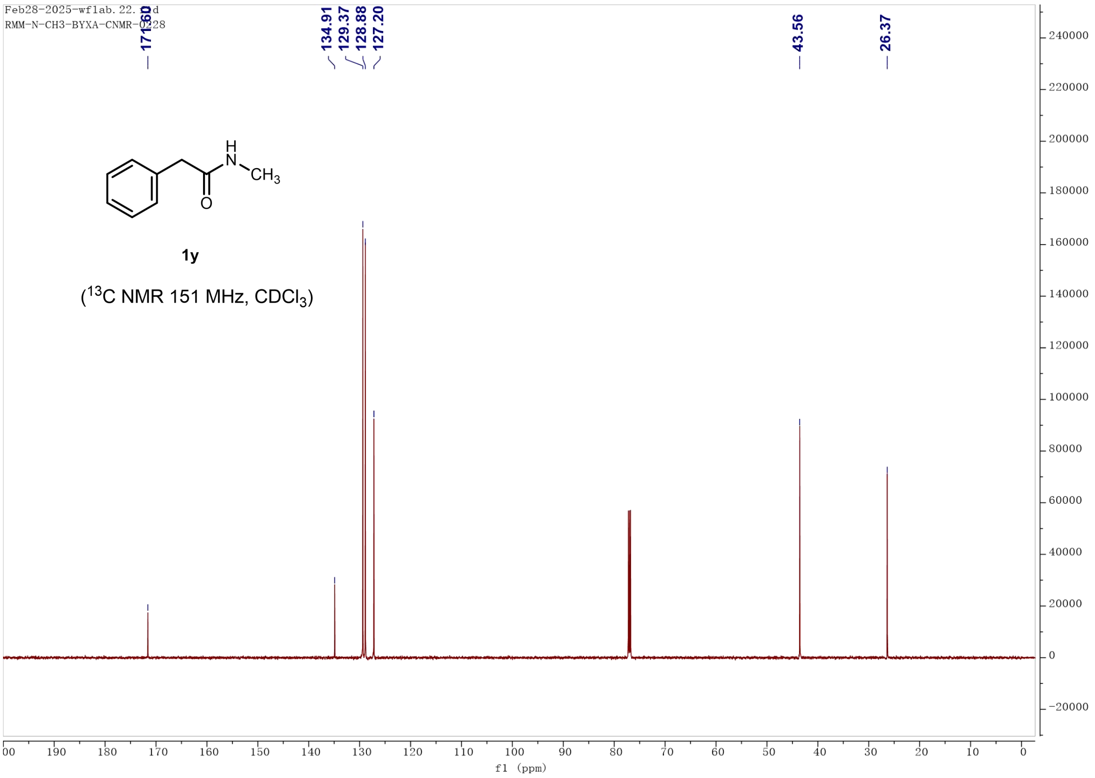
171.60
134.91 129.37 128.88 127.20
43.56
26.37
1y
(^{13}C NMR 151 MHz, $CDCl_3$)
f1 (ppm)

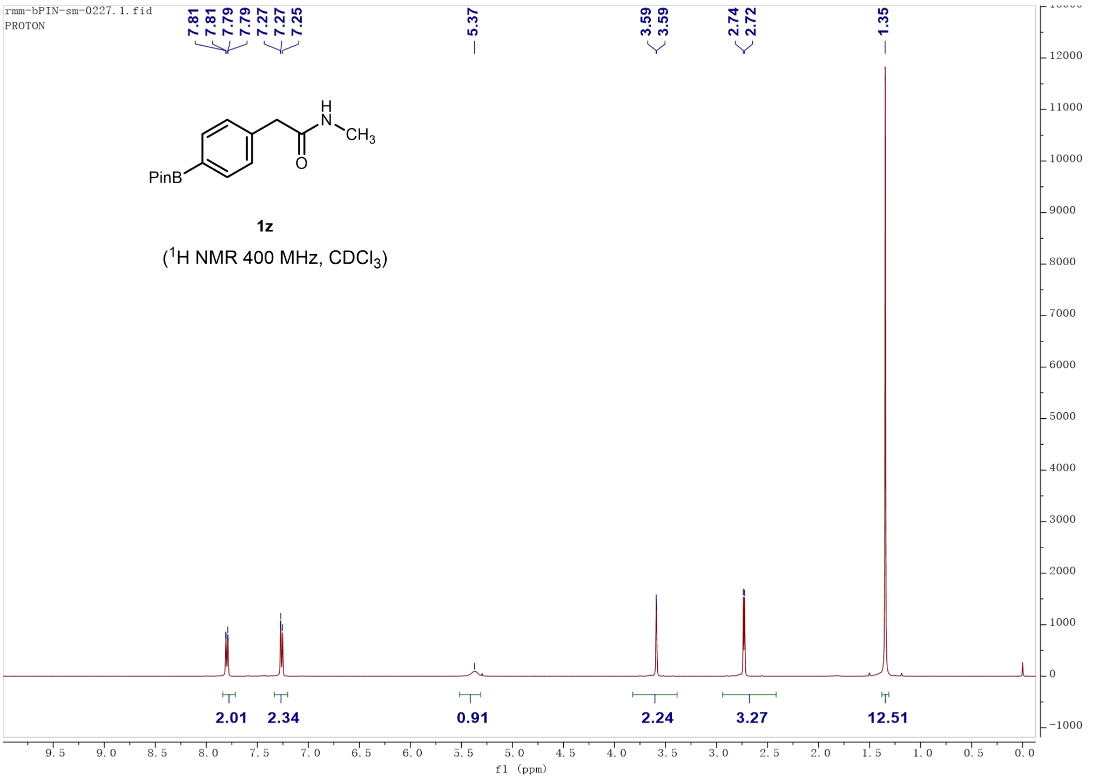
rmm-bPIN-sm-0227.1.fid
PROTON
7.81
7.81
7.79
7.79
7.27
7.27
7.25
5.37
3.59
3.59
2.74
2.72
1.35
PinB
H
N
CH3
O
1z
(1H NMR 400 MHz, CDCl3)
2.01
2.34
0.91
2.24
3.27
12.51
f1 (ppm)

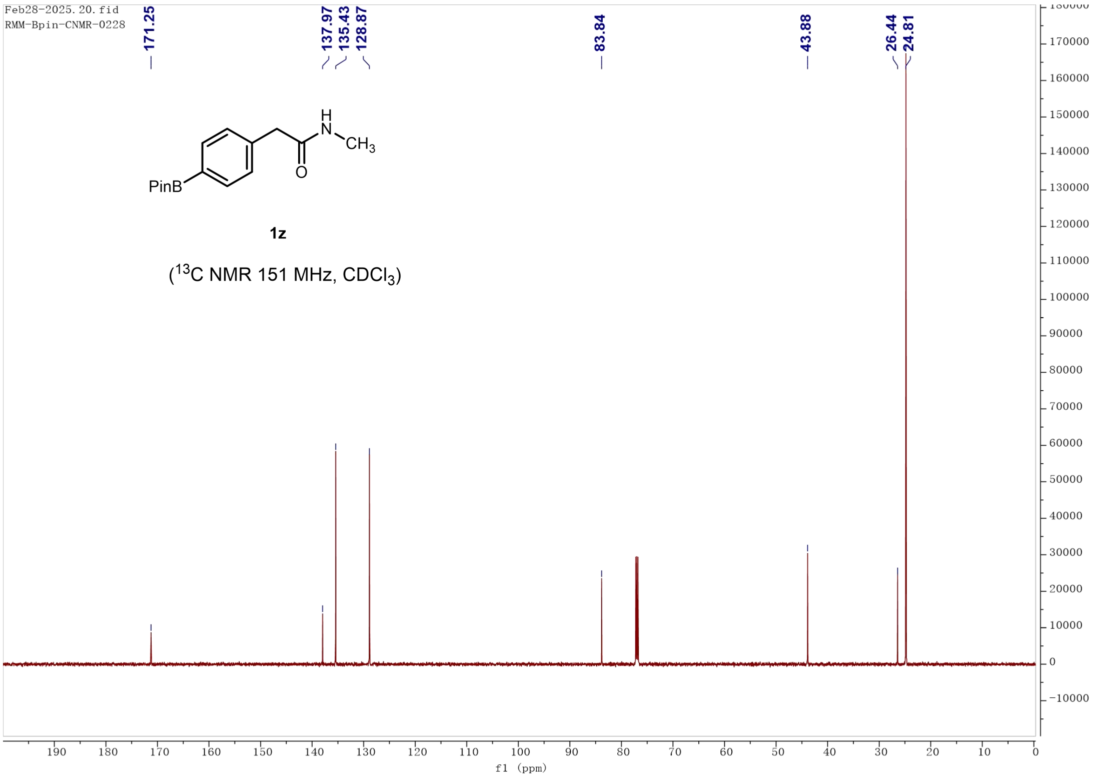
Feb28-2025.20.fid
RMM-Bpin-CNMR-0228
171.25
137.97
135.43
128.87
83.84
43.88
26.44
24.81
PinB
H
N
CH3
O
1z
(13C NMR 151 MHz, CDCl3)
f1 (ppm)

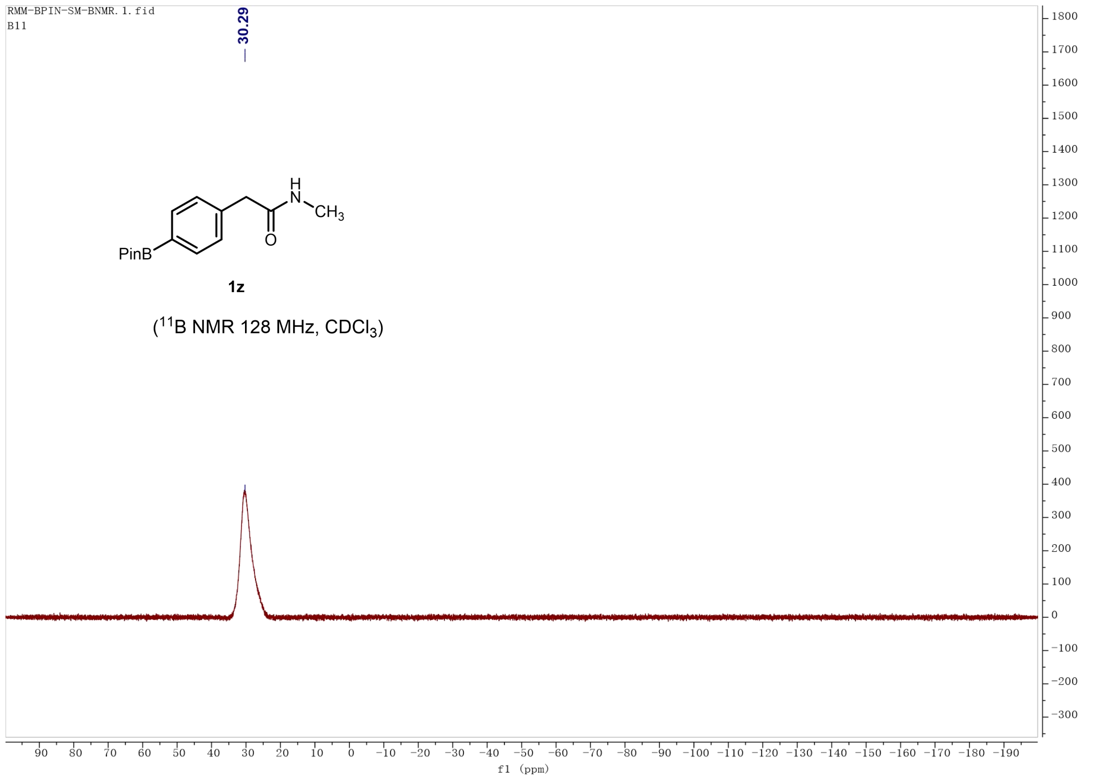
RMM-BPIN-SM-BNMR.1.fid
B11
30.29
PinB
H
N
CH3
O
1z
(11B NMR 128 MHz, CDCl3)
f1 (ppm)

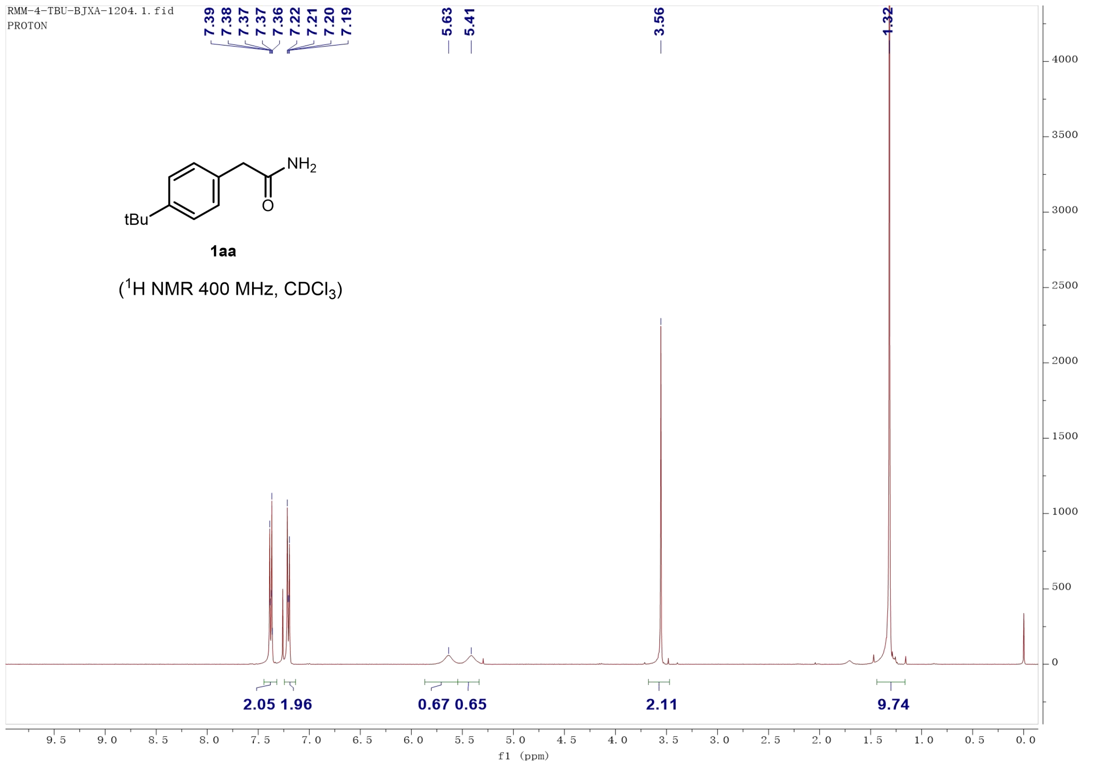
RMM-4-TBU-BJXA-1204.1.fid
PROTON
7.39 7.38 7.37 7.37 7.36 7.22 7.21 7.20 7.19
5.63 5.41
3.56
1.32
NH2
O
tBu
1aa
(1H NMR 400 MHz, CDCl3)
2.05 1.96 0.67 0.65 2.11 9.74
f1 (ppm)

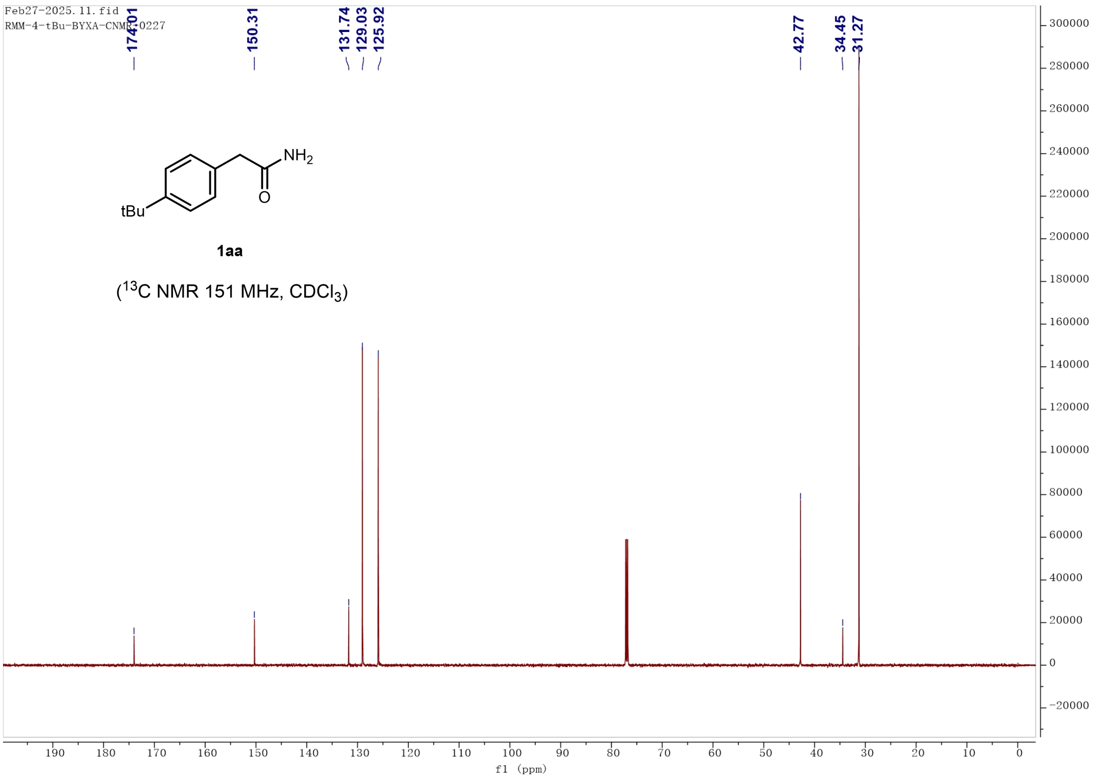

Feb27-2025.11.fid
RMM-4-tBu-BYXA-CNMR-0227
174.01
150.31
131.74
129.03
125.92
42.77
34.45
31.27
NH2
O
tBu
1aa
(13C NMR 151 MHz, CDCl3)
f1 (ppm)

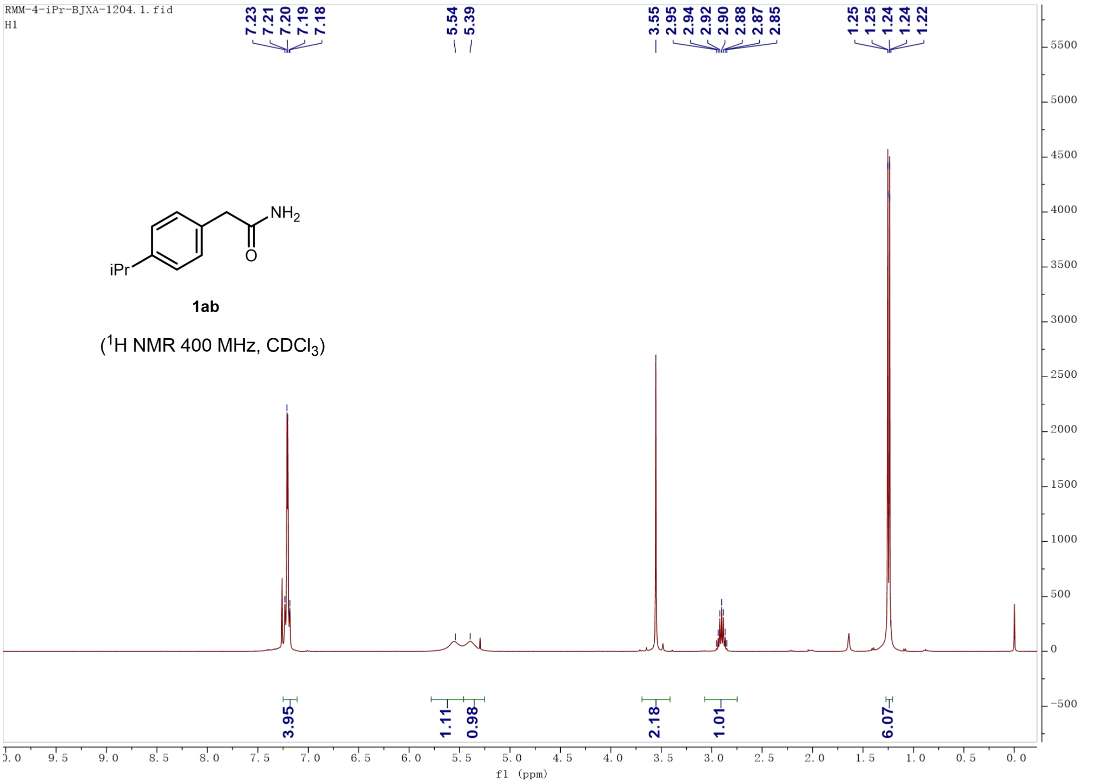

RMM-4-iPr-BJXA-1204.1.fid
H1
7.23
7.21
7.20
7.19
7.18
5.54
5.39
3.55
2.95
2.94
2.92
2.90
2.88
2.87
2.85
1.25
1.25
1.24
1.24
1.22
NH2
O
iPr
1ab
(1H NMR 400 MHz, CDCl3)
3.95
1.11
0.98
2.18
1.01
6.07
f1 (ppm)

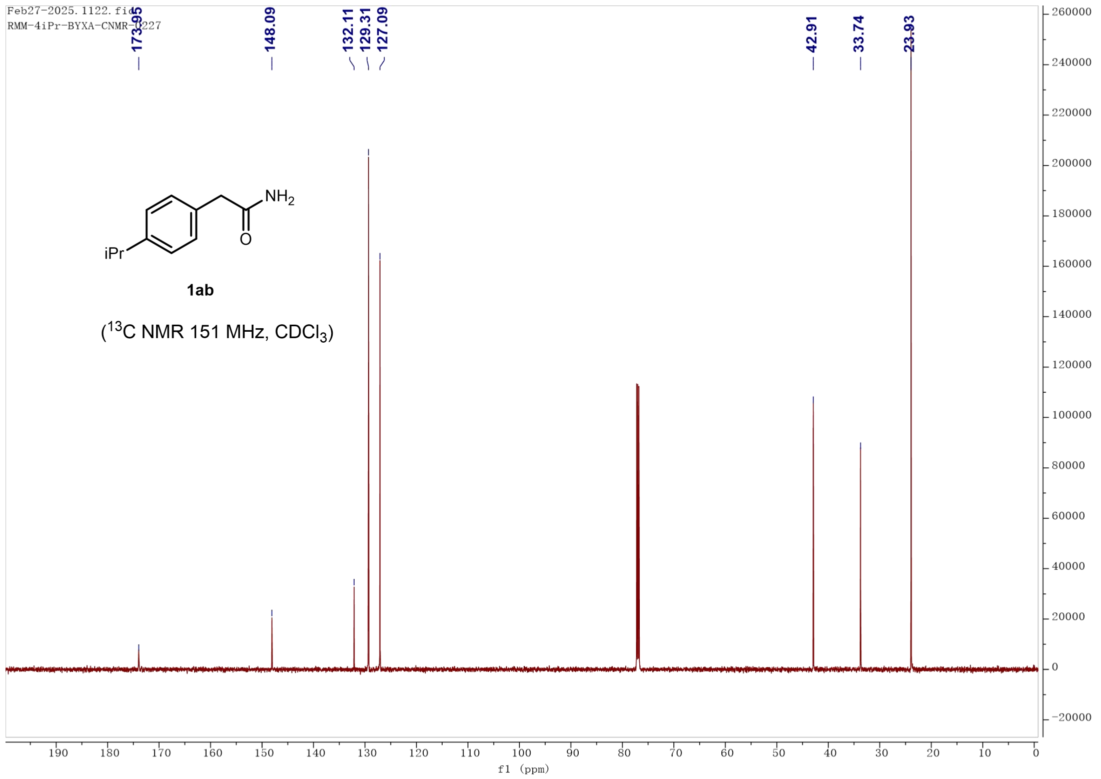
Feb27-2025.1122.fid
RMM-4iPr-BYXA-CNMR-0227
173.95
148.09
132.11
129.31
127.09
42.91
33.74
23.93
NH2
O
iPr
1ab
(13C NMR 151 MHz, CDCl3)
f1 (ppm)

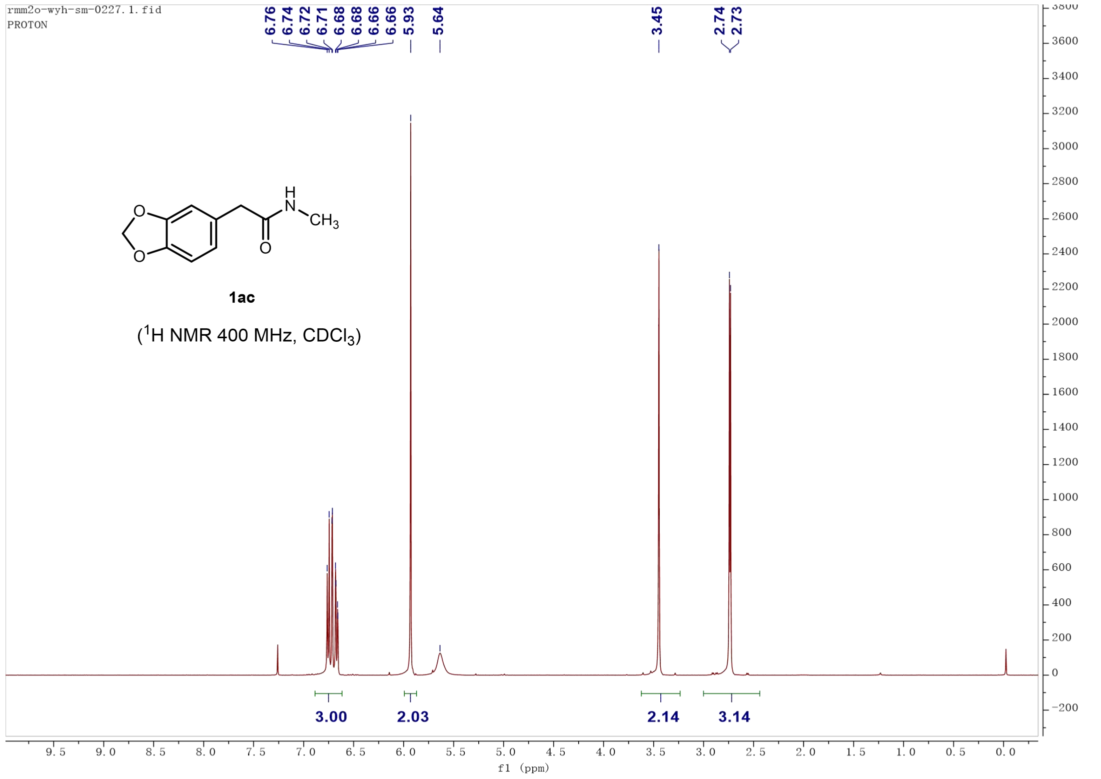
rmm2o-wyh-sm-0227.1.fid
PROTON
6.76
6.74
6.72
6.71
6.68
6.68
6.66
6.66
5.93
5.64
3.45
2.74
2.73
H
N
CH3
O
O
O
1ac
(1H NMR 400 MHz, CDCl3)
3.00
2.03
2.14
3.14
f1 (ppm)

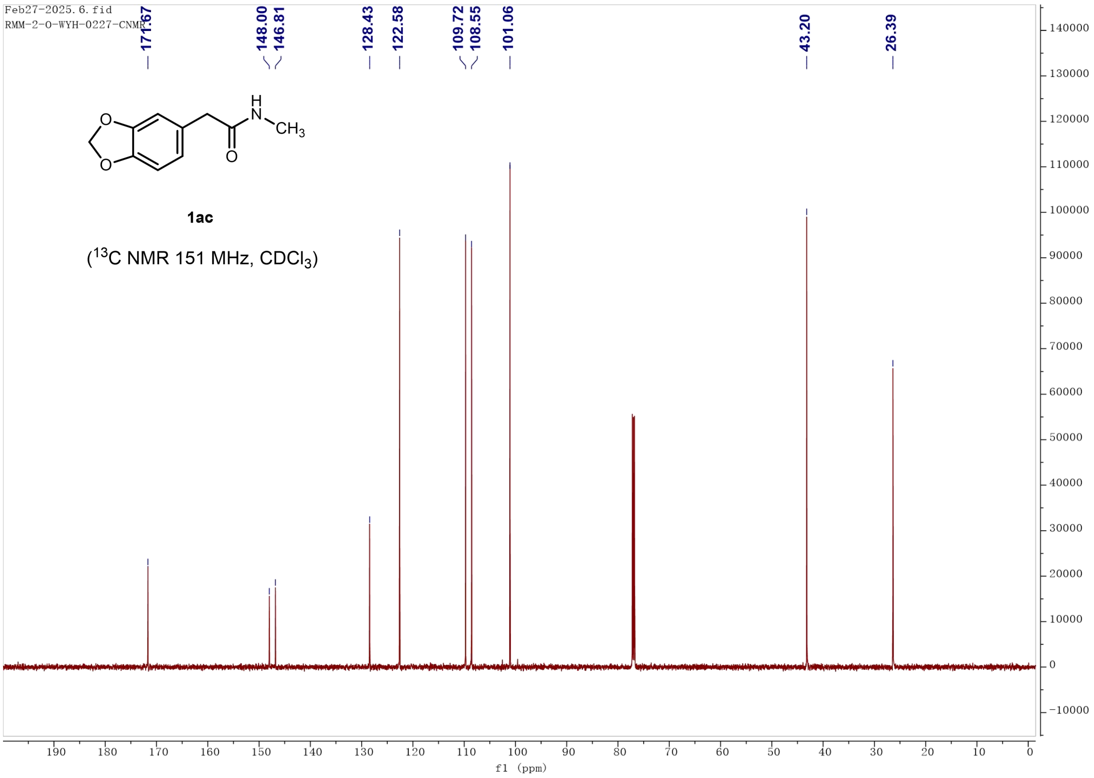
Feb27-2025.6.fid
RMM-2-O-WYH-0227-CNMR
171.67
148.00
146.81
128.43
122.58
109.72
108.55
101.06
43.20
26.39
1ac
(^{13}C NMR 151 MHz, CDCl$_3$)
f1 (ppm)

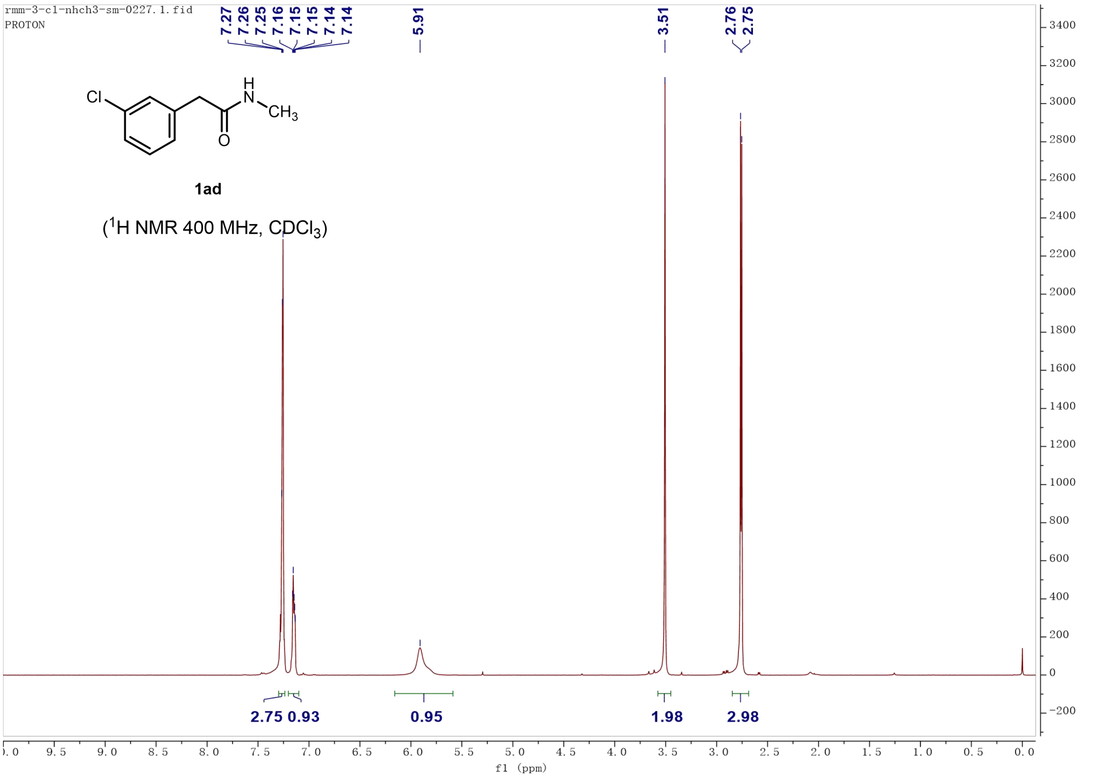
rmm-3-cl-nhch3-sm-0227.1.fid
PROTON
7.27
7.26
7.25
7.16
7.15
7.15
7.14
7.14
5.91
3.51
2.76
2.75
1ad
(^{1}H NMR 400 MHz, CDCl$_3$)
2.75
0.93
0.95
1.98
2.98
f1 (ppm)

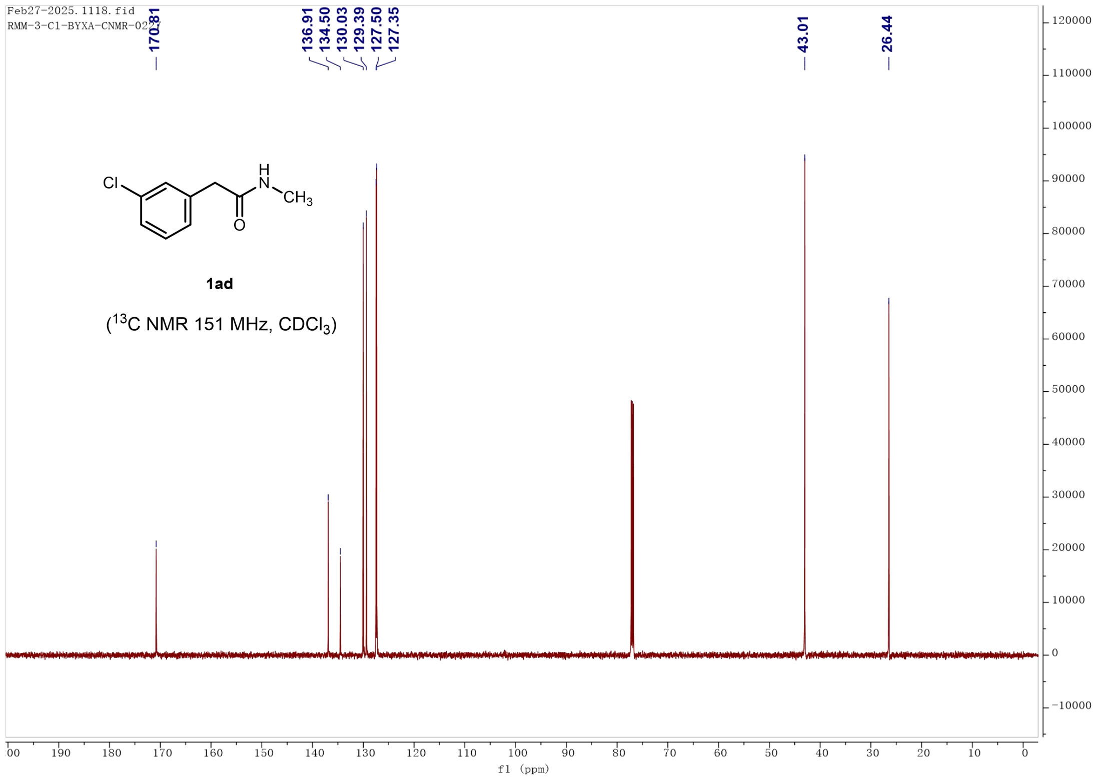
Feb27-2025.1118.fid
RMM-3-C1-BYXA-CNMR-02
170.81
136.91
134.50
130.03
129.39
127.50
127.35
43.01
26.44
Cl
H
N
CH3
O
1ad
(13C NMR 151 MHz, CDCl3)
f1 (ppm)

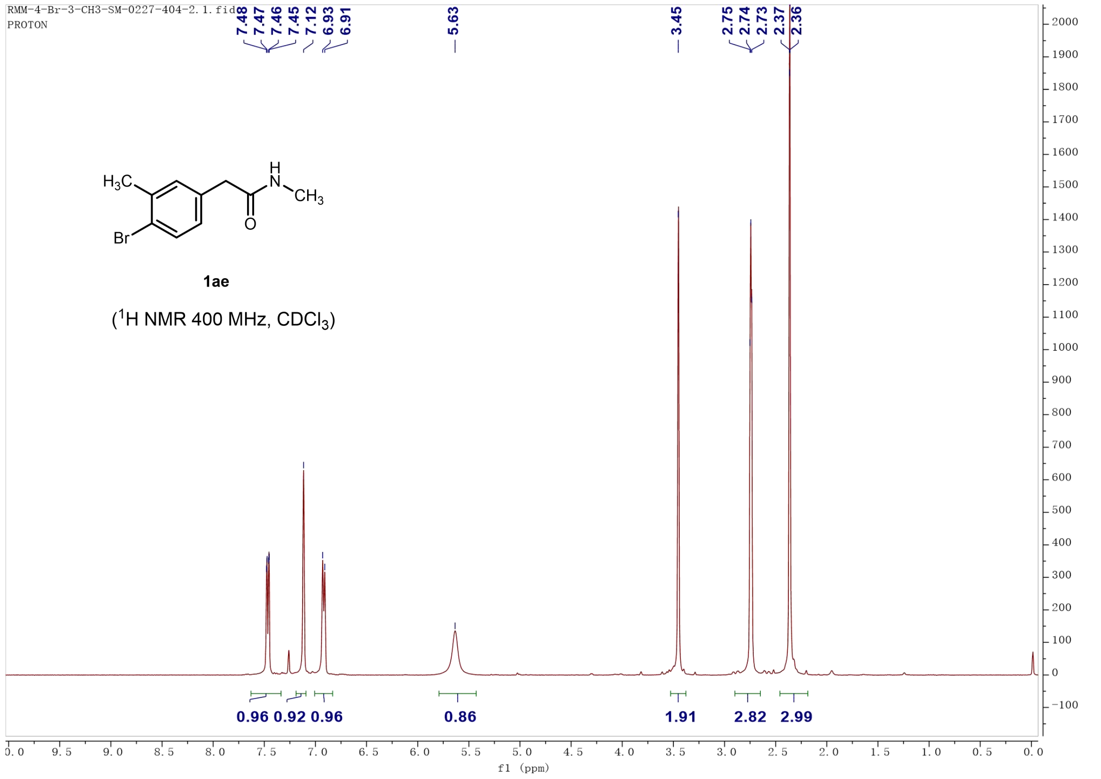
RMM-4-Br-3-CH3-SM-0227-404-2.1.fid
PROTON
7.48
7.47
7.46
7.45
7.12
6.93
6.91
5.63
3.45
2.75
2.74
2.73
2.37
2.36
H3C
Br
H
N
CH3
O
1ae
(1H NMR 400 MHz, CDCl3)
0.96
0.92
0.96
0.86
1.91
2.82
2.99
f1 (ppm)

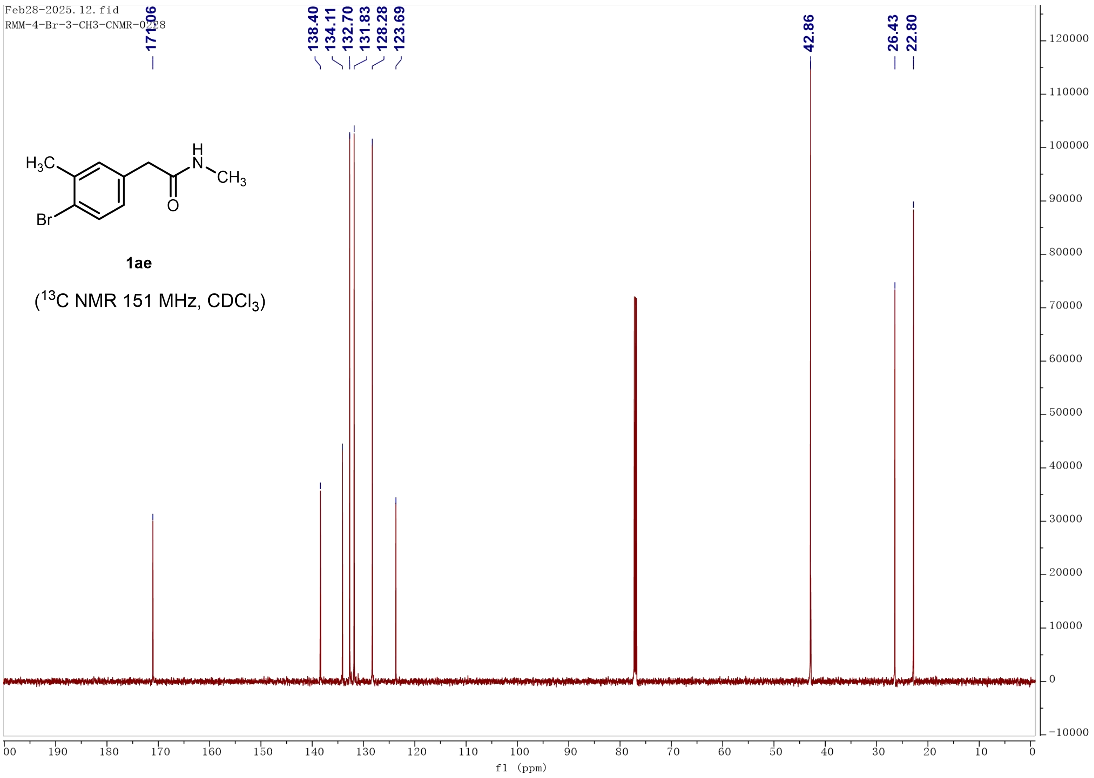
Feb28-2025.12.fid
RMM-4-Br-3-CH3-CNMR-0228
171.06
138.40
134.11
132.70
131.83
128.28
123.69
42.86
26.43
22.80
1ae
(13C NMR 151 MHz, CDCl3)
f1 (ppm)

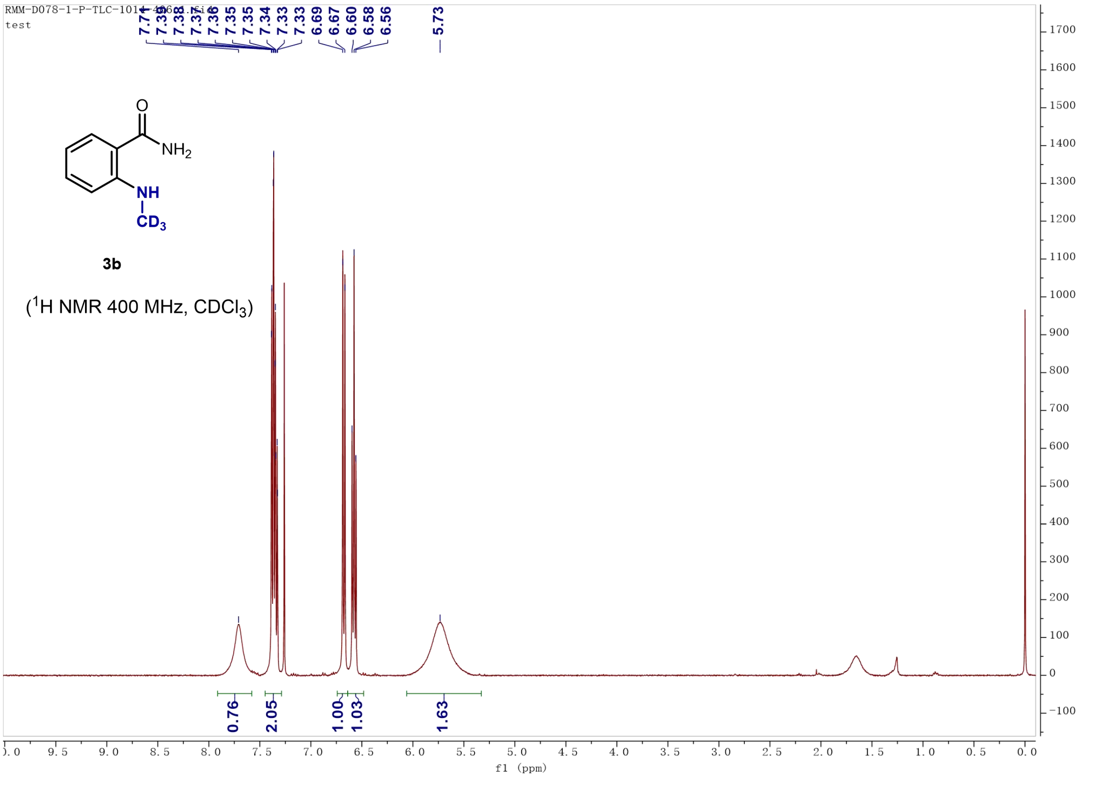
RMM-D078-1-P-TLC-1014
test
7.71
7.39
7.38
7.37
7.36
7.35
7.35
7.34
7.33
7.33
6.69
6.67
6.60
6.58
6.56
5.73
3b
(1H NMR 400 MHz, CDCl3)
0.76
2.05
1.00
1.03
1.63
f1 (ppm)

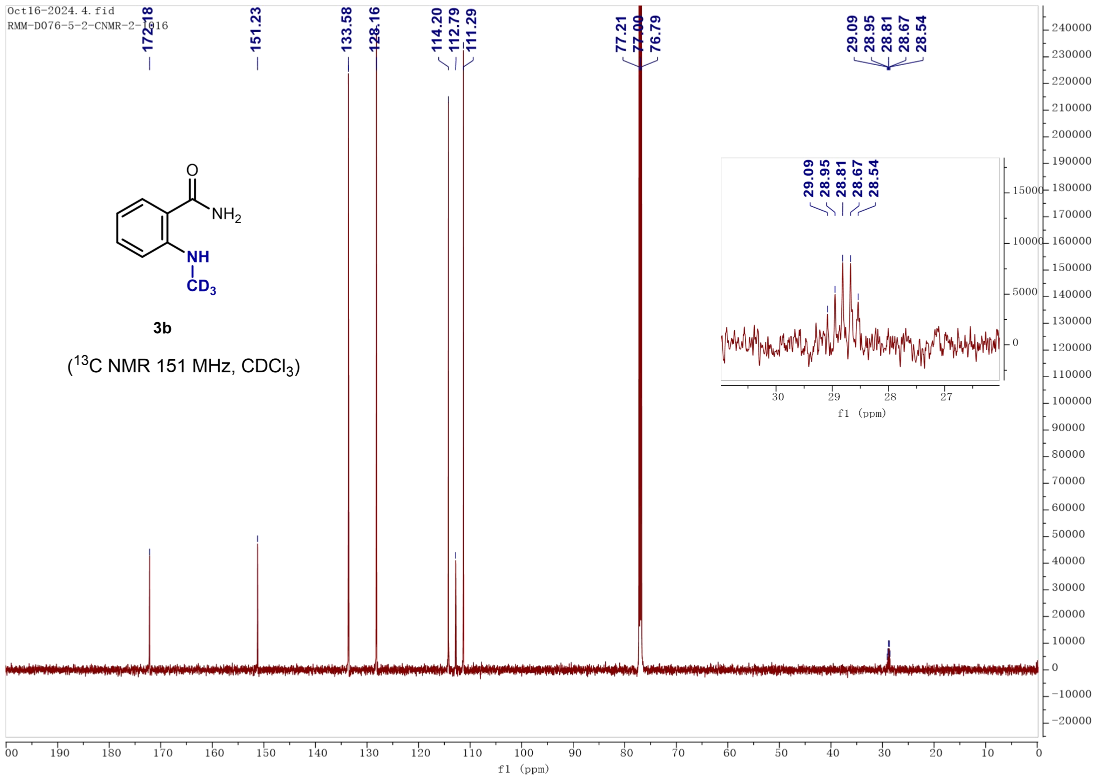

Oct16-2024.4.fid
RMM-D076-5-2-CNMR-2-1016
172.18
151.23
133.58
128.16
114.20
112.79
111.29
77.21
77.00
76.79
29.09
28.95
28.81
28.67
28.54
NH2
NH
CD3
3b
(13C NMR 151 MHz, CDCl3)
f1 (ppm)

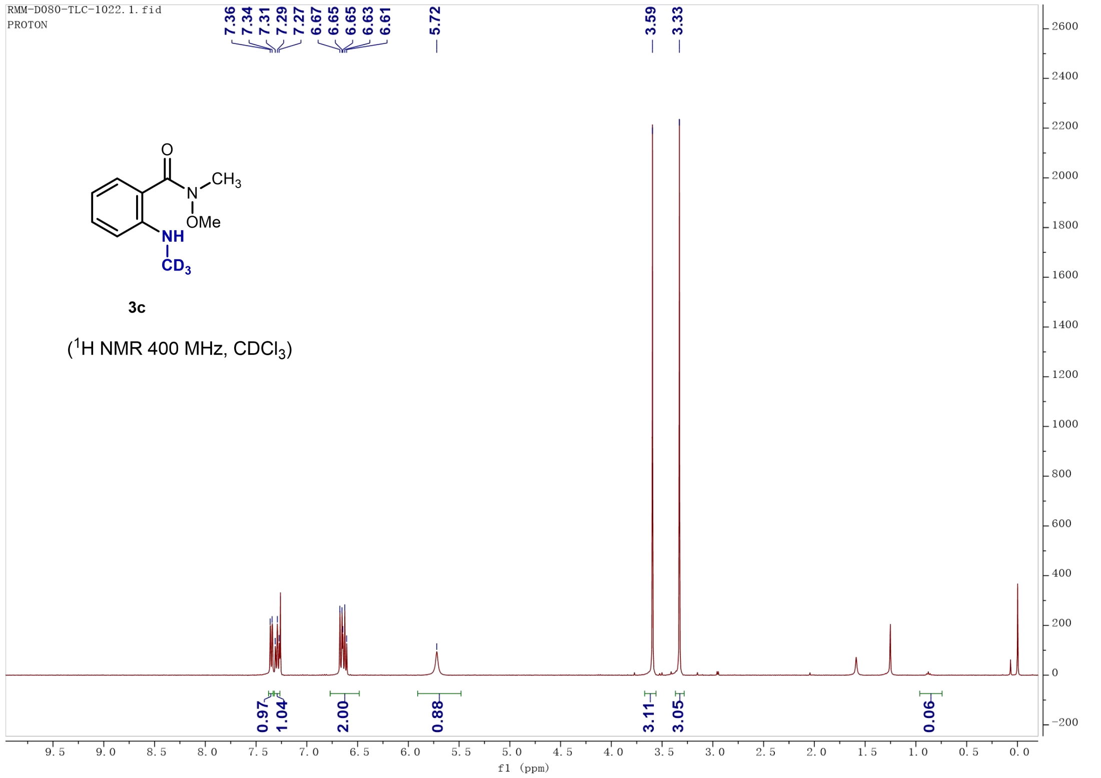

RMM-D080-TLC-1022.1.fid
PROTON
7.36
7.34
7.31
7.29
7.27
6.67
6.65
6.65
6.63
6.61
5.72
3.59
3.33
CH3
OMe
NH
CD3
3c
(1H NMR 400 MHz, CDCl3)
0.97
1.04
2.00
0.88
3.11
3.05
0.06
f1 (ppm)

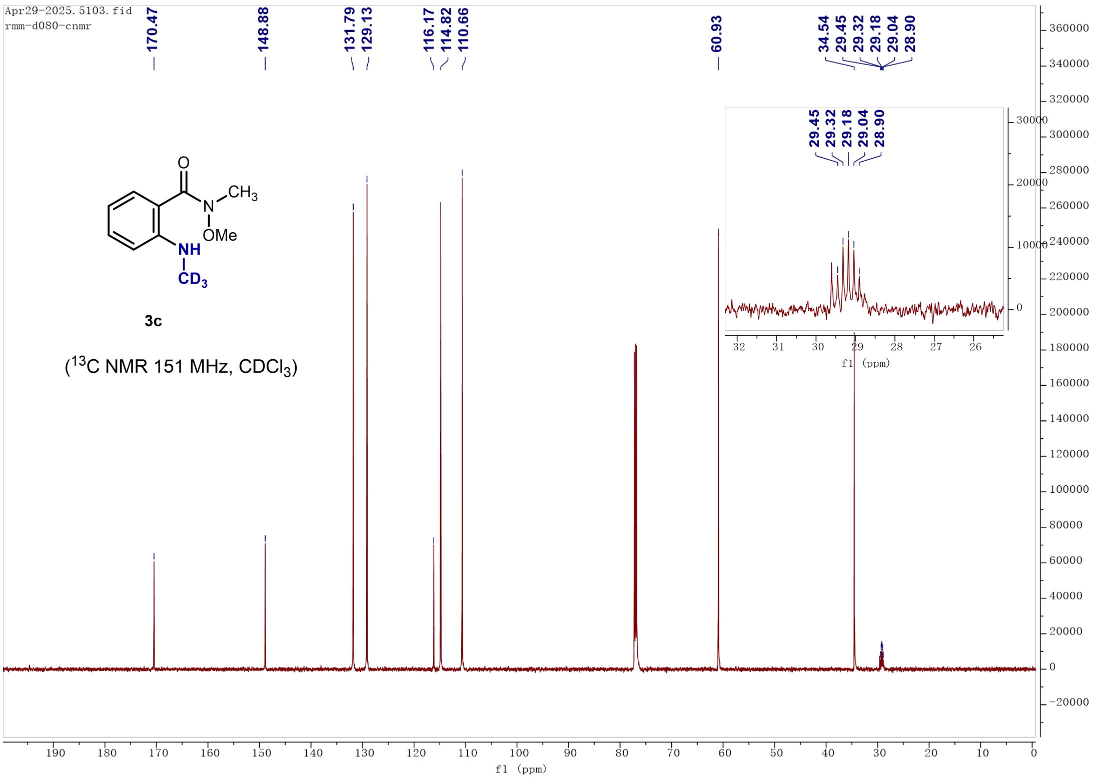
Apr29-2025.5103.fid
rmm-d080-cnmr
170.47
148.88
131.79
129.13
116.17
114.82
110.66
60.93
34.54
29.45
29.32
29.18
29.04
28.90
3c
(13C NMR 151 MHz, CDCl3)
f1 (ppm)

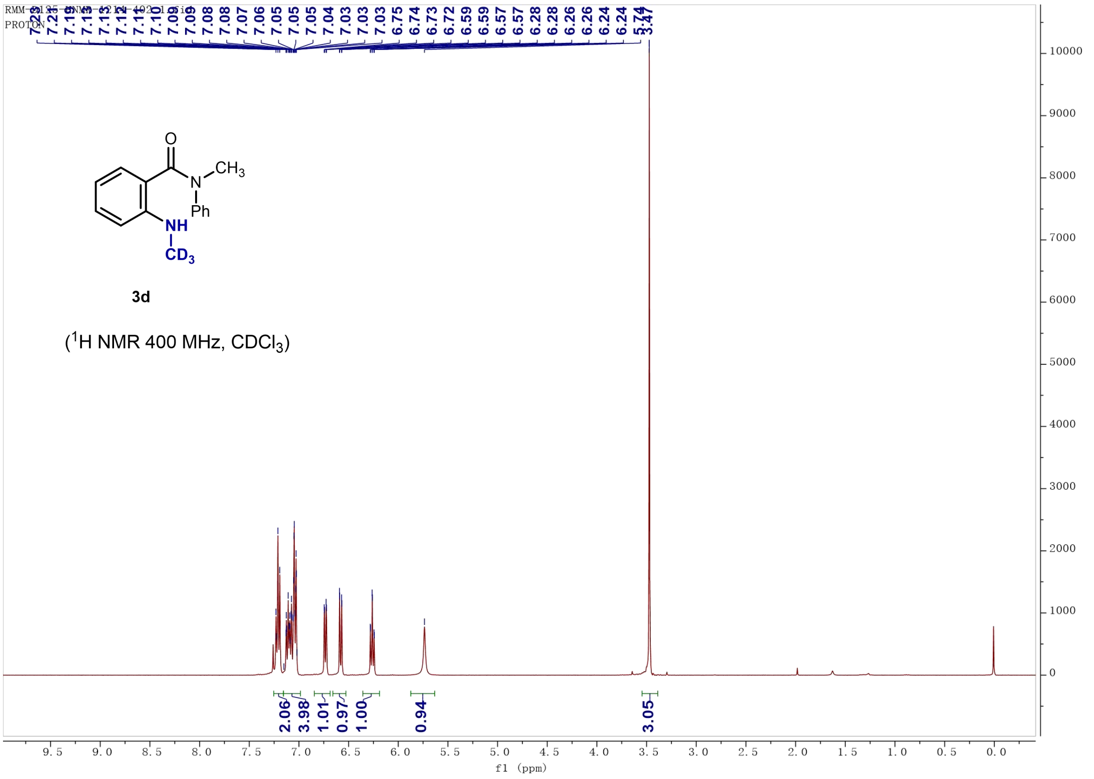
PROTON
7.23
7.21
7.15
7.13
7.11
7.10
7.09
7.09
7.08
7.07
7.06
7.05
7.05
7.04
7.03
7.03
6.75
6.74
6.73
6.72
6.59
6.57
6.57
6.28
6.26
6.26
6.24
6.24
5.74
3.47
3d
(1H NMR 400 MHz, CDCl3)
2.06
3.98
1.01
0.97
1.00
0.94
3.05
f1 (ppm)

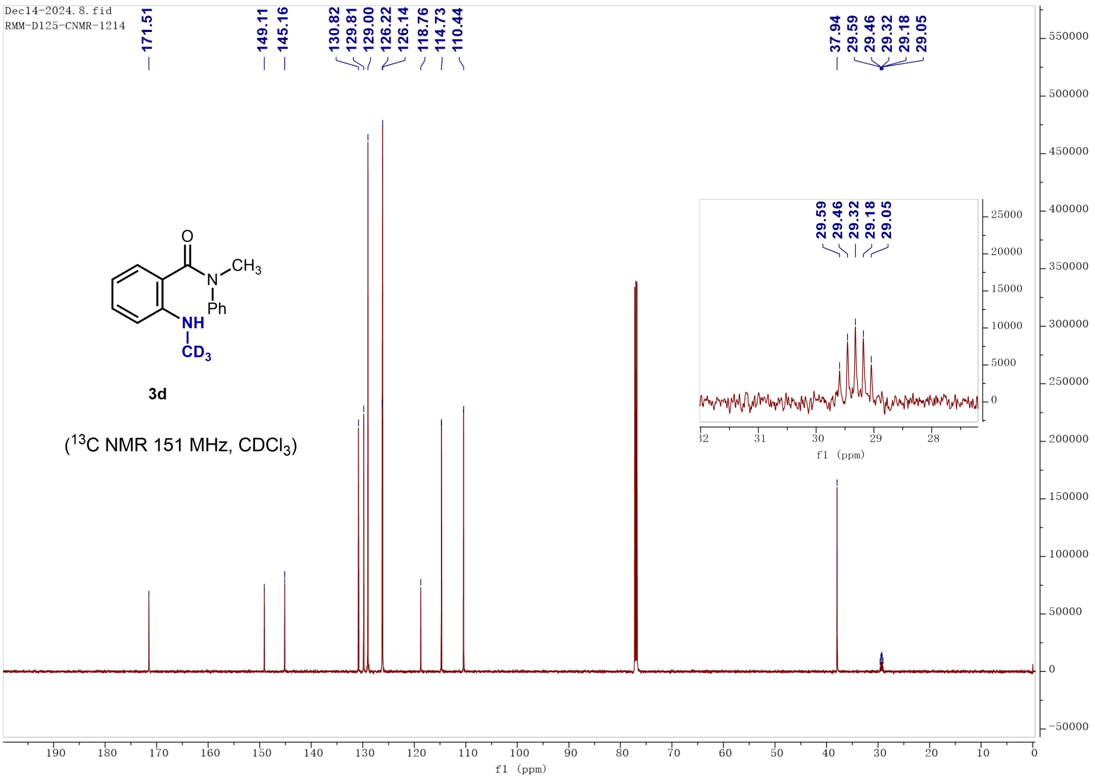
Dec14-2024.8.fid
RMM-D125-CNMR-1214
171.51
149.11
145.16
130.82
129.81
129.00
126.22
126.14
118.76
114.73
110.44
37.94
29.59
29.46
29.32
29.18
29.05
3d
(^{13}C NMR 151 MHz, $CDCl_3$)
f1 (ppm)

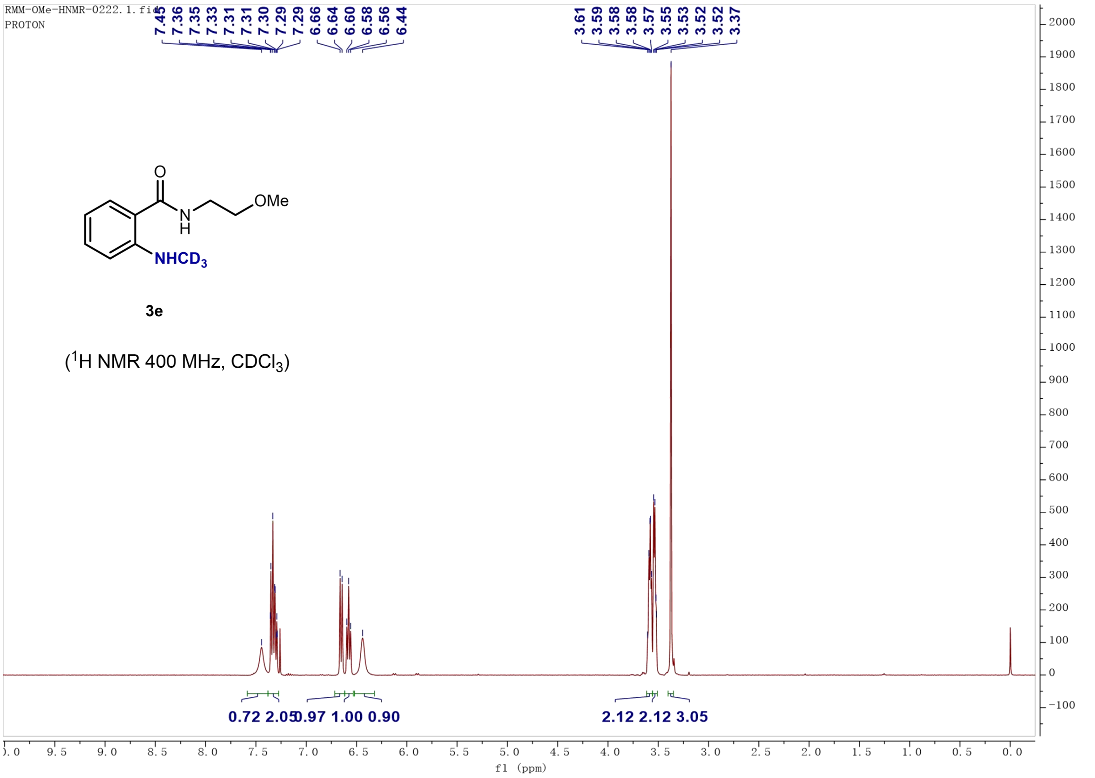
RMM-OMe-HNMR-0222.1.fid
PROTON
7.45
7.36
7.35
7.33
7.31
7.30
7.29
7.29
6.66
6.64
6.60
6.58
6.56
6.44
3.61
3.59
3.58
3.58
3.57
3.55
3.53
3.52
3.52
3.37
3e
(^{1}H NMR 400 MHz, $CDCl_3$)
0.72 2.05 0.97 1.00 0.90
2.12 2.12 3.05
f1 (ppm)

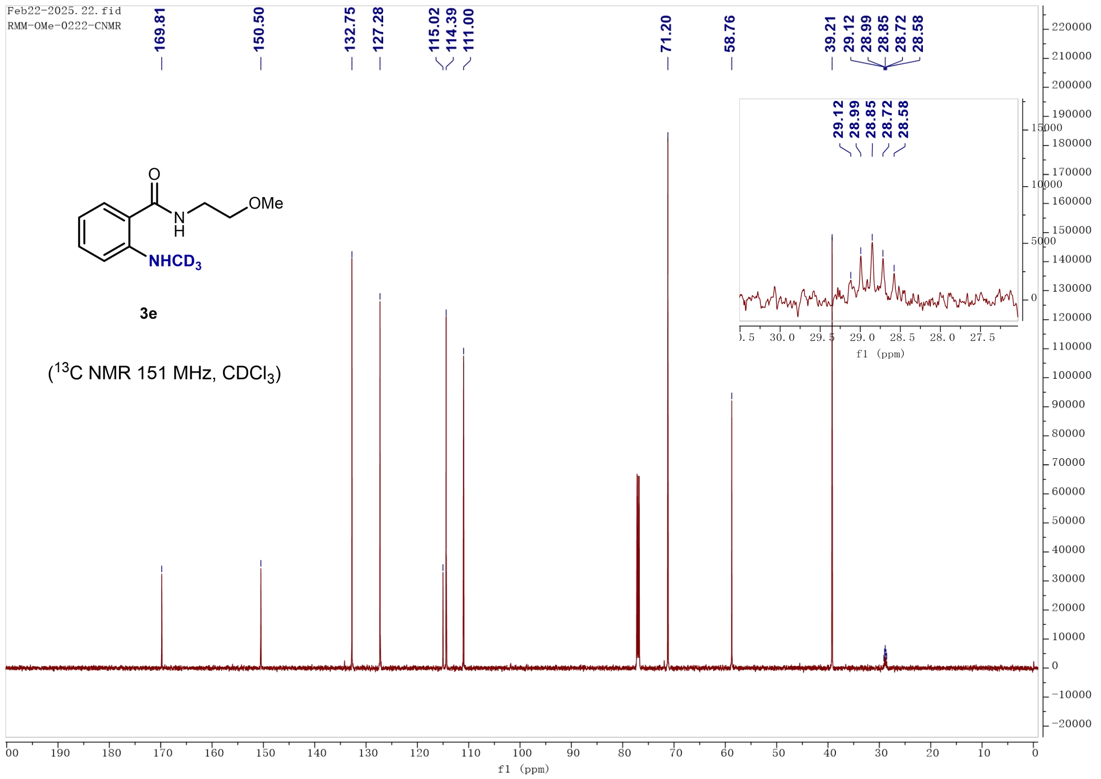
Feb22-2025.22.fid
RMM-OMe-0222-CNMR
169.81
150.50
132.75
127.28
115.02
114.39
111.00
71.20
58.76
39.21
29.12
28.99
28.85
28.72
28.58
OMe
NHCD3
3e
(13C NMR 151 MHz, CDCl3)
f1 (ppm)

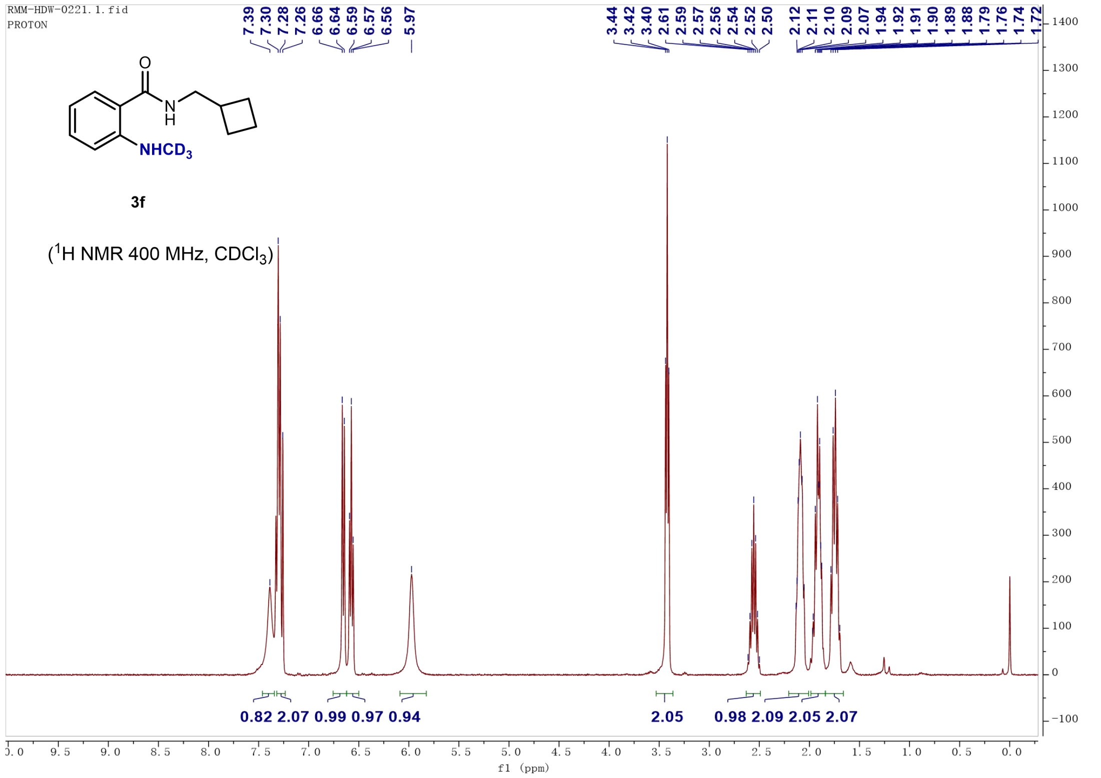
RMM-HDW-0221.1.fid
PROTON
7.39
7.30
7.28
7.26
6.66
6.64
6.59
6.57
6.56
5.97
3.44
3.42
3.40
2.61
2.59
2.57
2.56
2.54
2.52
2.50
2.12
2.11
2.10
2.09
2.07
1.94
1.92
1.91
1.90
1.89
1.88
1.79
1.76
1.74
1.72
NHCD3
3f
(1H NMR 400 MHz, CDCl3)
0.82 2.07 0.99 0.97 0.94
2.05
0.98 2.09 2.05 2.07
f1 (ppm)

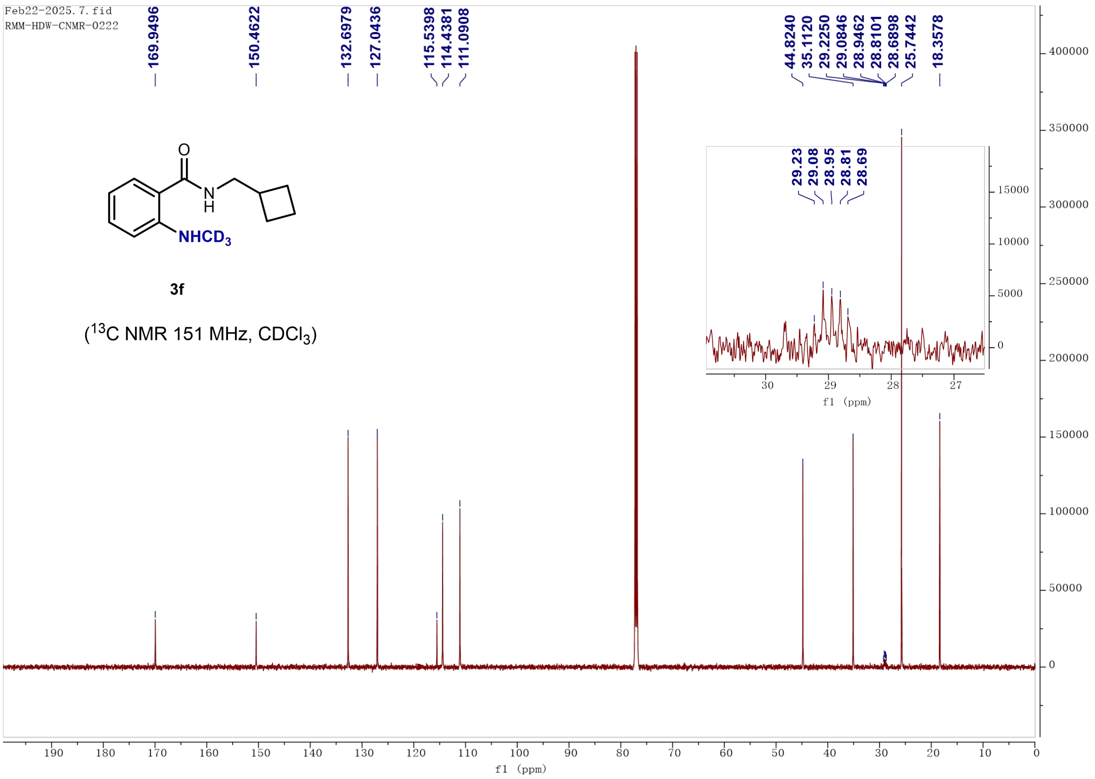
Feb22-2025.7.fid
RMM-HDW-CNMR-0222
169.9496
150.4622
132.6979
127.0436
115.5398
114.4381
111.0908
44.8240
35.1120
29.2250
29.0846
28.9462
28.8101
28.6898
25.7442
18.3578
NHCD3
3f
(13C NMR 151 MHz, CDCl3)
29.23
29.08
28.95
28.81
28.69
f1 (ppm)

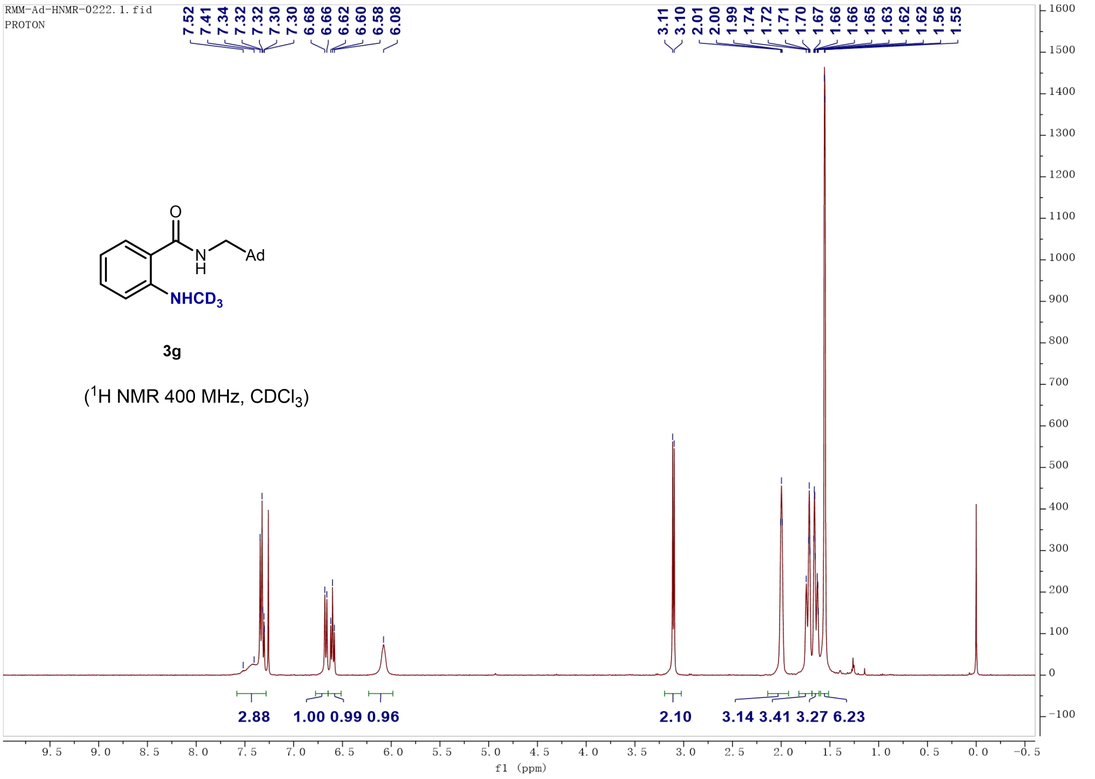
RMM-Ad-HNMR-0222.1.fid
PROTON
7.52
7.41
7.34
7.32
7.32
7.30
7.30
6.68
6.66
6.62
6.60
6.58
6.08
3.11
3.10
2.01
2.00
1.99
1.74
1.72
1.71
1.70
1.67
1.66
1.65
1.63
1.62
1.56
1.55
Ad
NHCD3
3g
(1H NMR 400 MHz, CDCl3)
2.88
1.00 0.99 0.96
2.10
3.14 3.41 3.27 6.23
f1 (ppm)

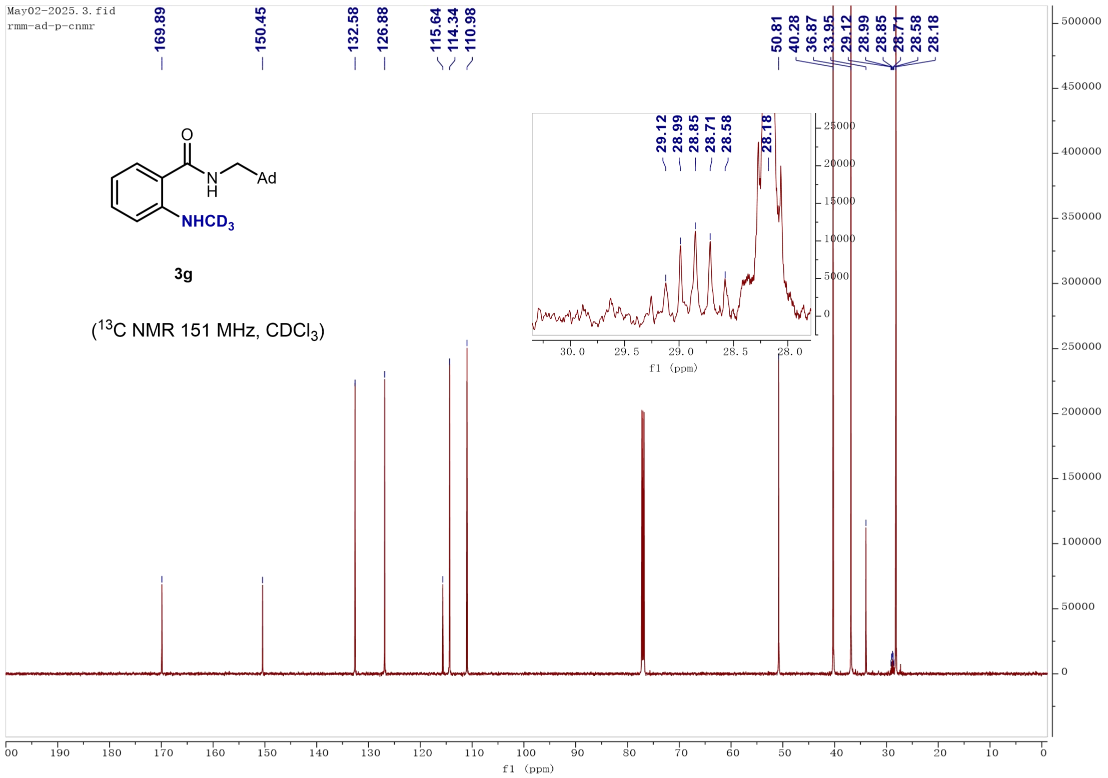
May02-2025.3.fid
rmm-ad-p-cnmr
169.89
150.45
132.58
126.88
115.64
114.34
110.98
50.81
40.28
36.87
33.95
29.12
28.99
28.85
28.71
28.58
28.18
NHCD3
3g
(13C NMR 151 MHz, CDCl3)
f1 (ppm)

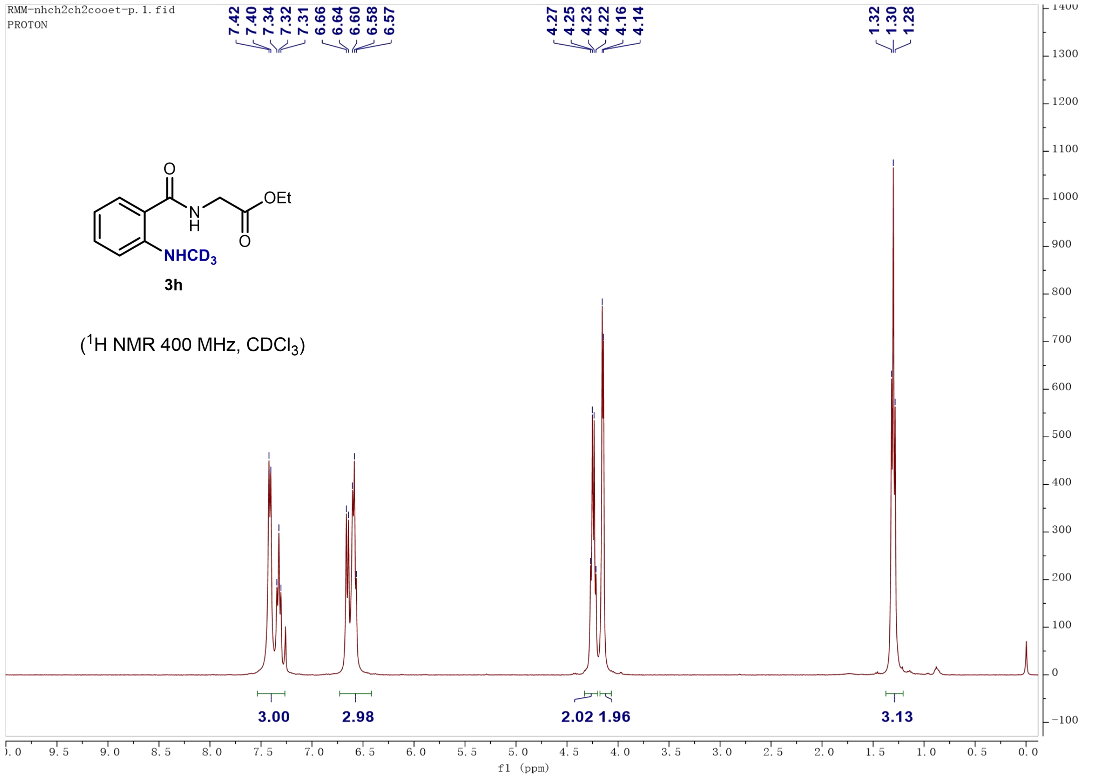
RMM-nhch2ch2cooet-p.1.fid
PROTON
7.42
7.40
7.34
7.32
7.31
6.66
6.64
6.60
6.58
6.57
4.27
4.25
4.23
4.22
4.16
4.14
1.32
1.30
1.28
OEt
NHCD3
3h
(1H NMR 400 MHz, CDCl3)
3.00
2.98
2.02
1.96
3.13
f1 (ppm)

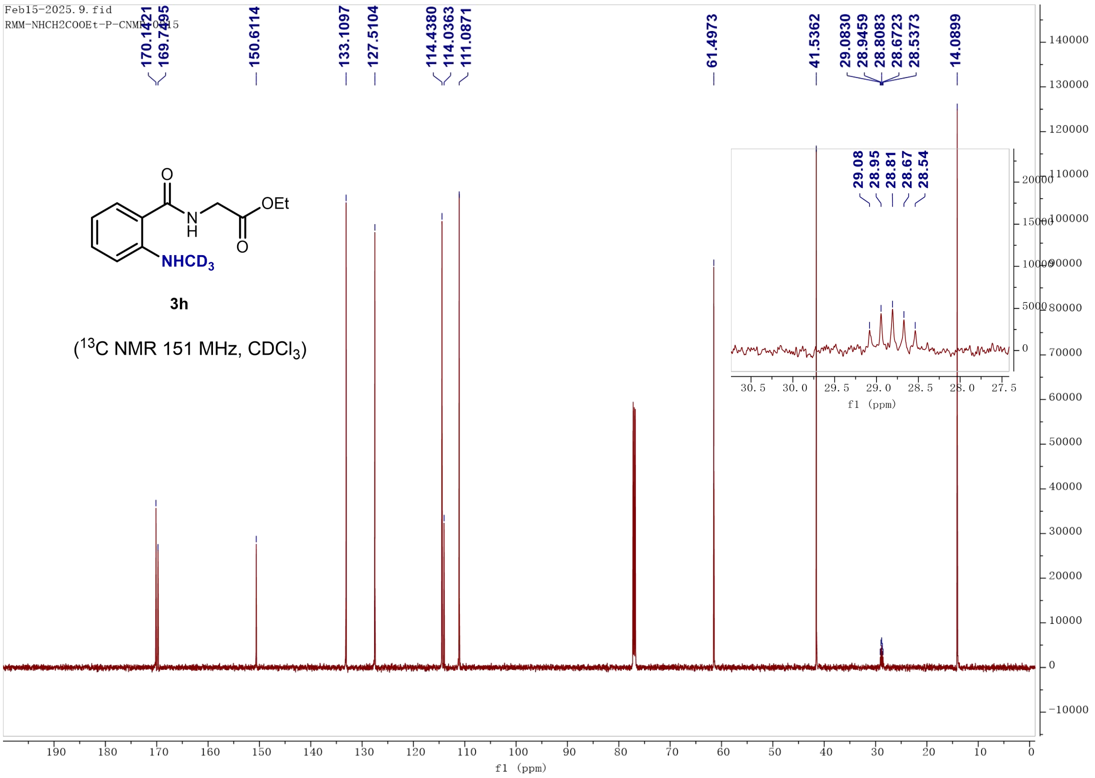
Feb15-2025.9.fid
170.1421
169.7495
150.6114
133.1097
127.5104
114.4380
114.0363
111.0871
61.4973
41.5362
29.0830
28.9459
28.8083
28.6723
28.5373
14.0899
29.08
28.95
28.81
28.67
28.54
f1 (ppm)
O
N
H
OEt
O
NHCD3
3h
(13C NMR 151 MHz, CDCl3)
f1 (ppm)

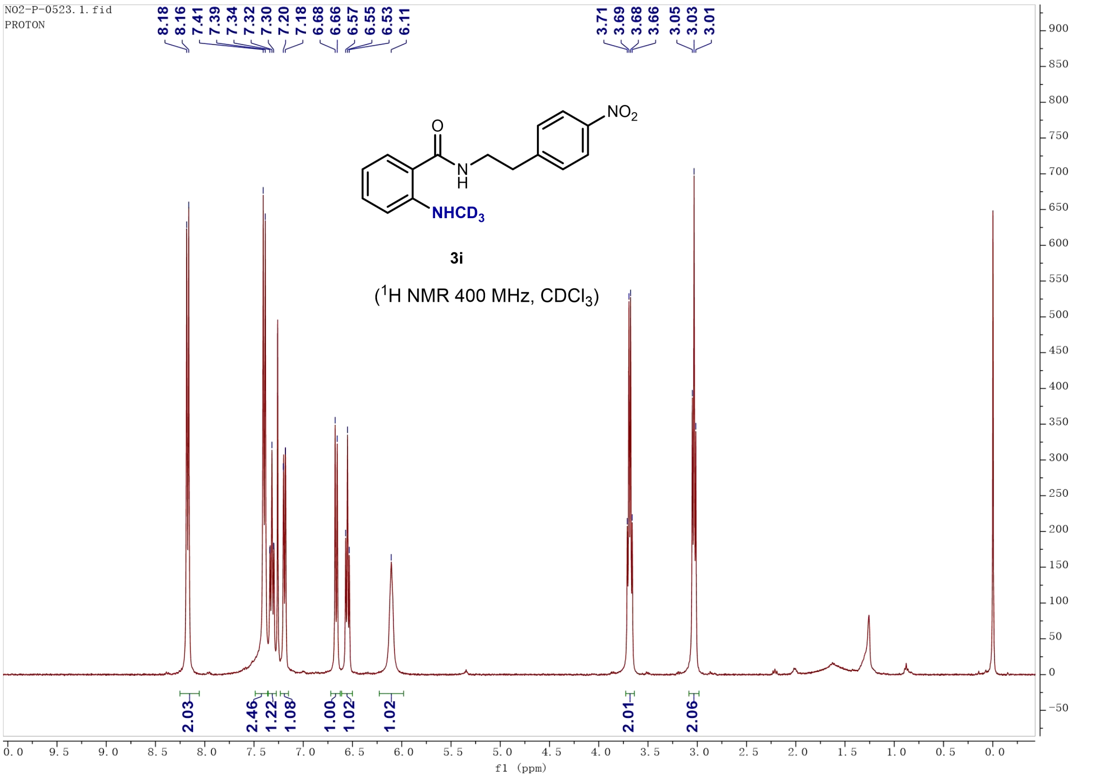
NO2-P-0523.1.fid
PROTON
8.18
8.16
7.41
7.39
7.34
7.32
7.30
7.20
7.18
6.68
6.66
6.57
6.55
6.53
6.11
3.71
3.69
3.68
3.66
3.05
3.03
3.01
O
NO2
N
H
NHCD3
3i
(1H NMR 400 MHz, CDCl3)
2.03
2.46
1.22
1.08
1.00
1.02
1.02
2.01
2.06
f1 (ppm)

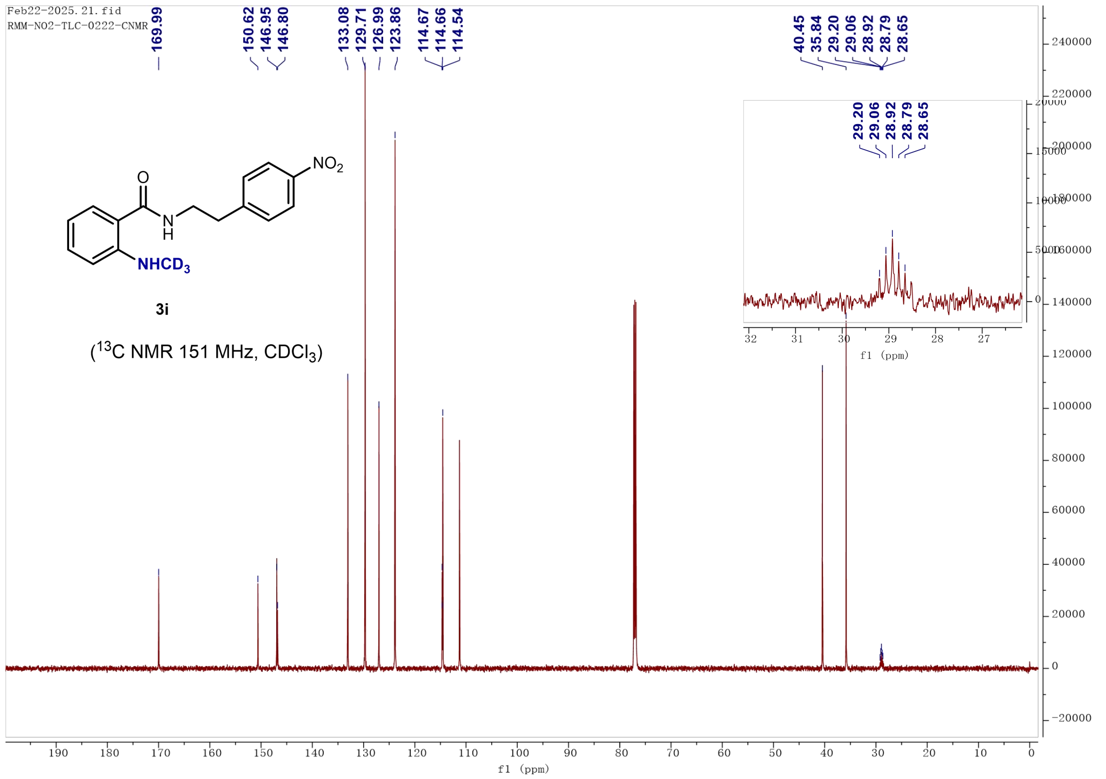

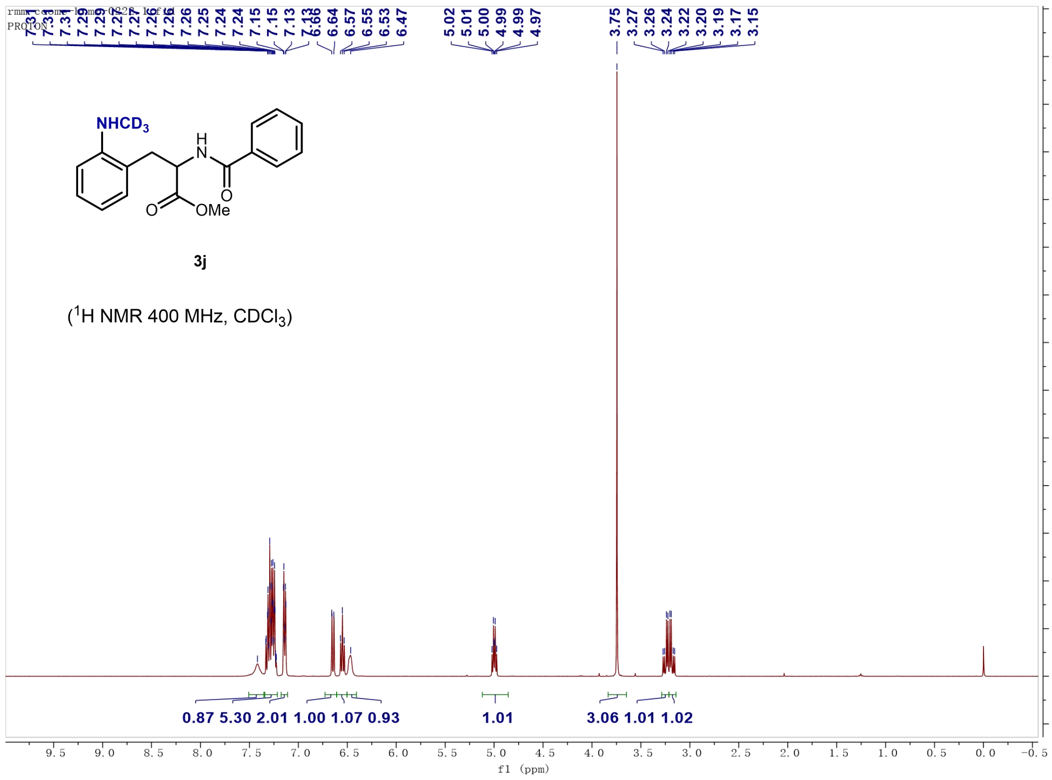

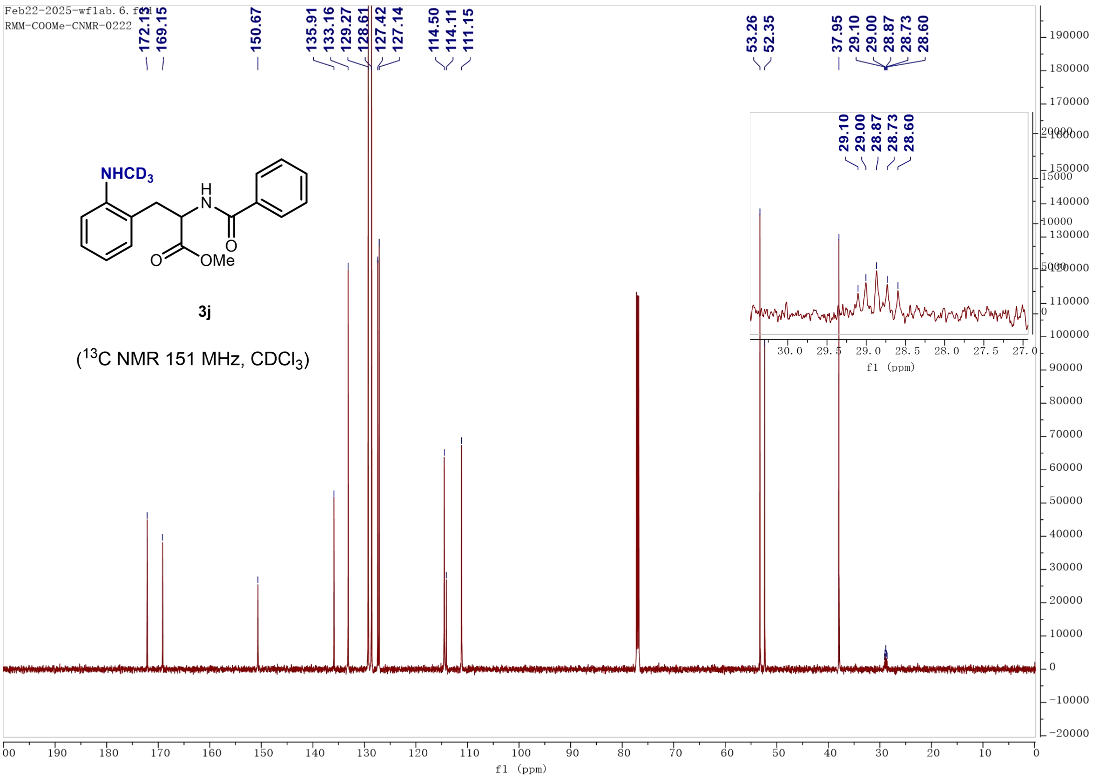
Feb22-2025-wflab.6.fid
RMM-COOMe-CNMR-0222
172.13
169.15
150.67
135.91
133.16
129.27
128.61
127.42
127.14
114.50
114.11
111.15
53.26
52.35
37.95
29.10
29.00
28.87
28.73
28.60
NHCD3
H
N
O
O
OMe
3j
(13C NMR 151 MHz, CDCl3)
f1 (ppm)

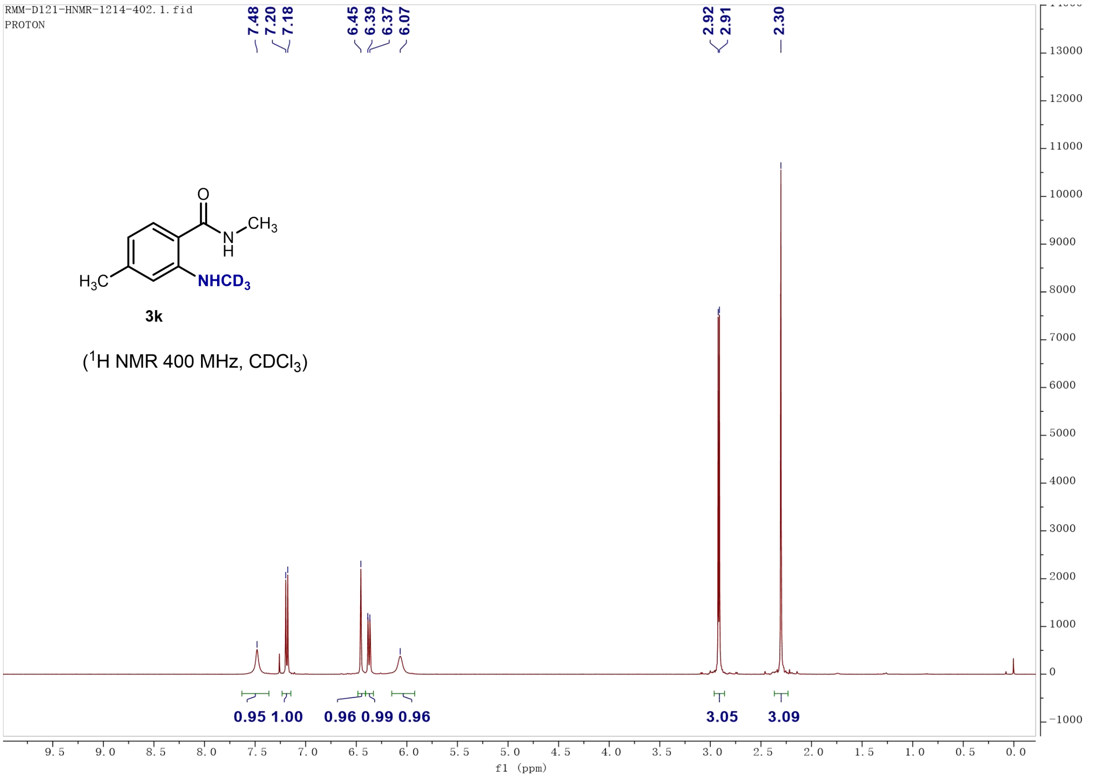
RMM-D121-HNMR-1214-402.1.fid
PROTON
7.48
7.20
7.18
6.45
6.39
6.37
6.07
2.92
2.91
2.30
O
N
H
CH3
H3C
NHCD3
3k
(1H NMR 400 MHz, CDCl3)
0.95
1.00
0.96
0.99
0.96
3.05
3.09
f1 (ppm)

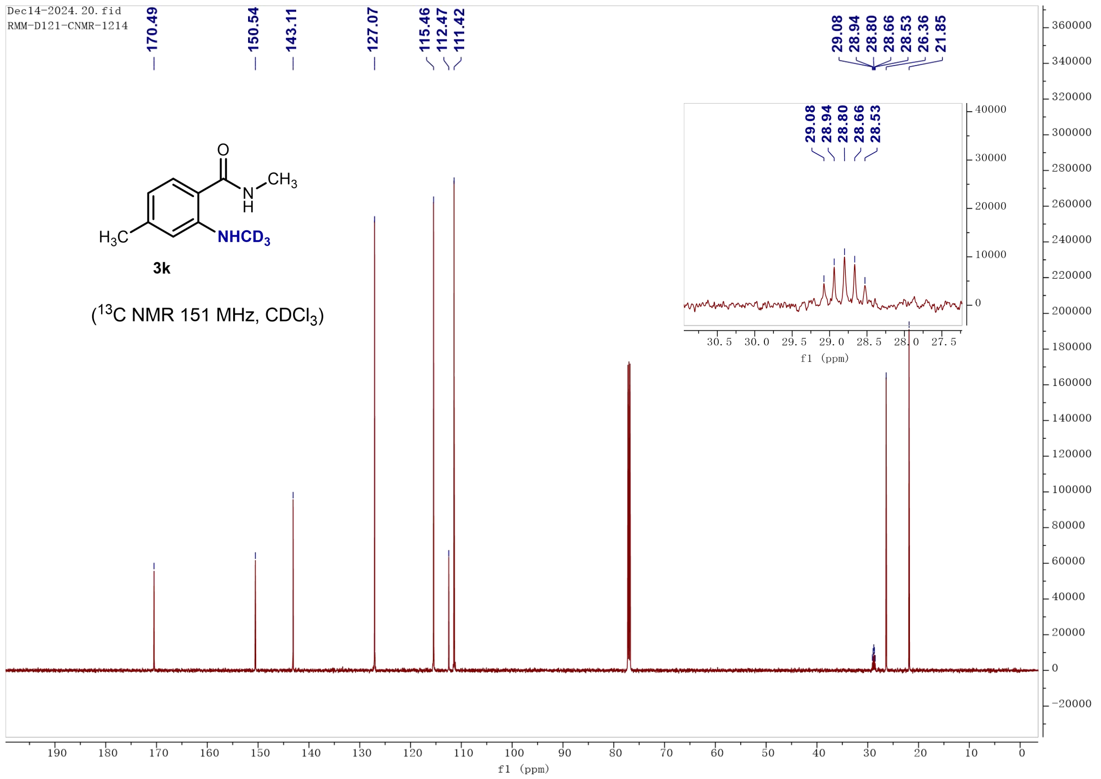
Dec14-2024.20.fid
RMM-D121-CNMR-1214
170.49
150.54
143.11
127.07
115.46
112.47
111.42
29.08
28.94
28.80
28.66
28.53
26.36
21.85
NHCD3
3k
(13C NMR 151 MHz, CDCl3)
f1 (ppm)

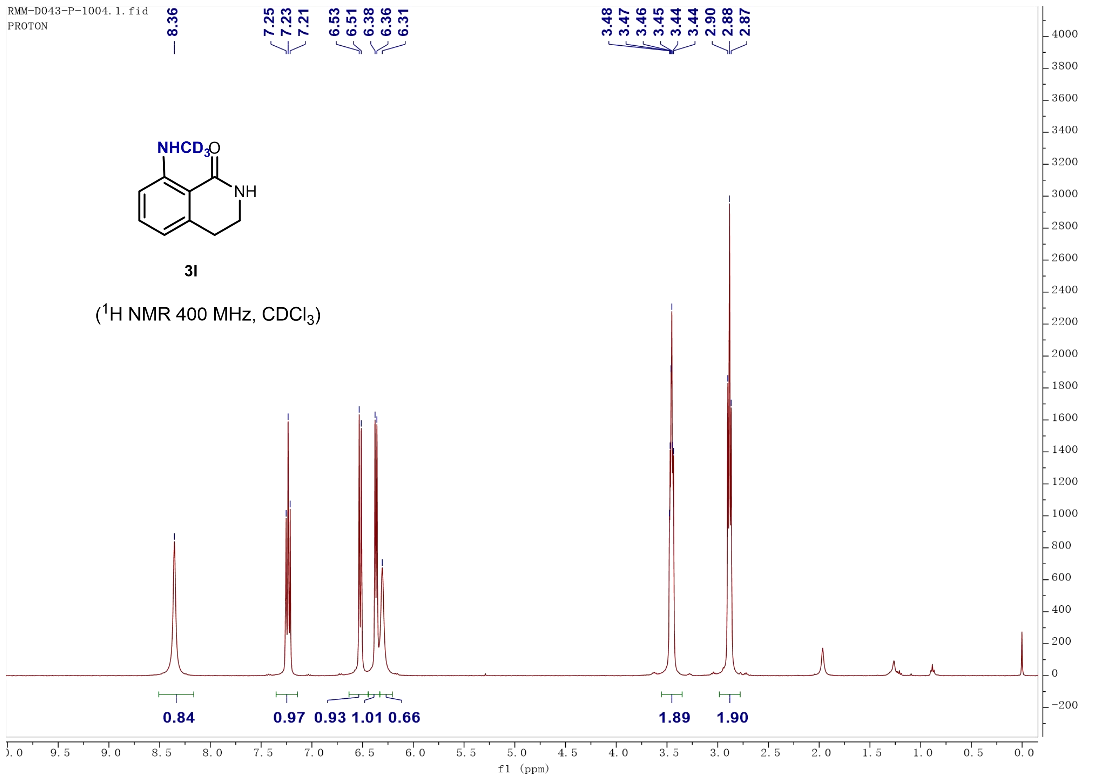
RMM-D043-P-1004.1.fid
PROTON
8.36
7.25
7.23
7.21
6.53
6.51
6.38
6.36
6.31
3.48
3.47
3.46
3.45
3.44
3.44
2.90
2.88
2.87
NHCD3
NH
3l
(1H NMR 400 MHz, CDCl3)
0.84
0.97
0.93
1.01
0.66
1.89
1.90
f1 (ppm)

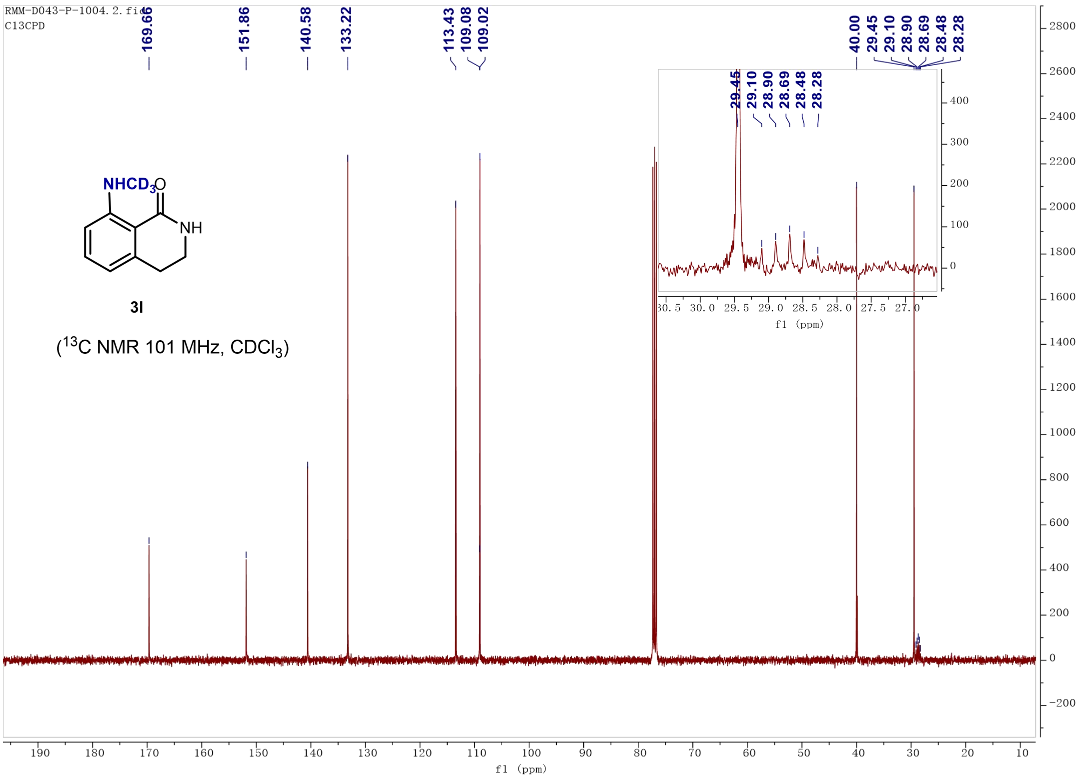
RMM-D043-P-1004.2.fid
C13CPD
169.66
151.86
140.58
133.22
113.43
109.08
109.02
40.00
29.45
29.10
28.90
28.69
28.48
28.28
NHCD3
O
NH
3l
(13C NMR 101 MHz, CDCl3)
f1 (ppm)

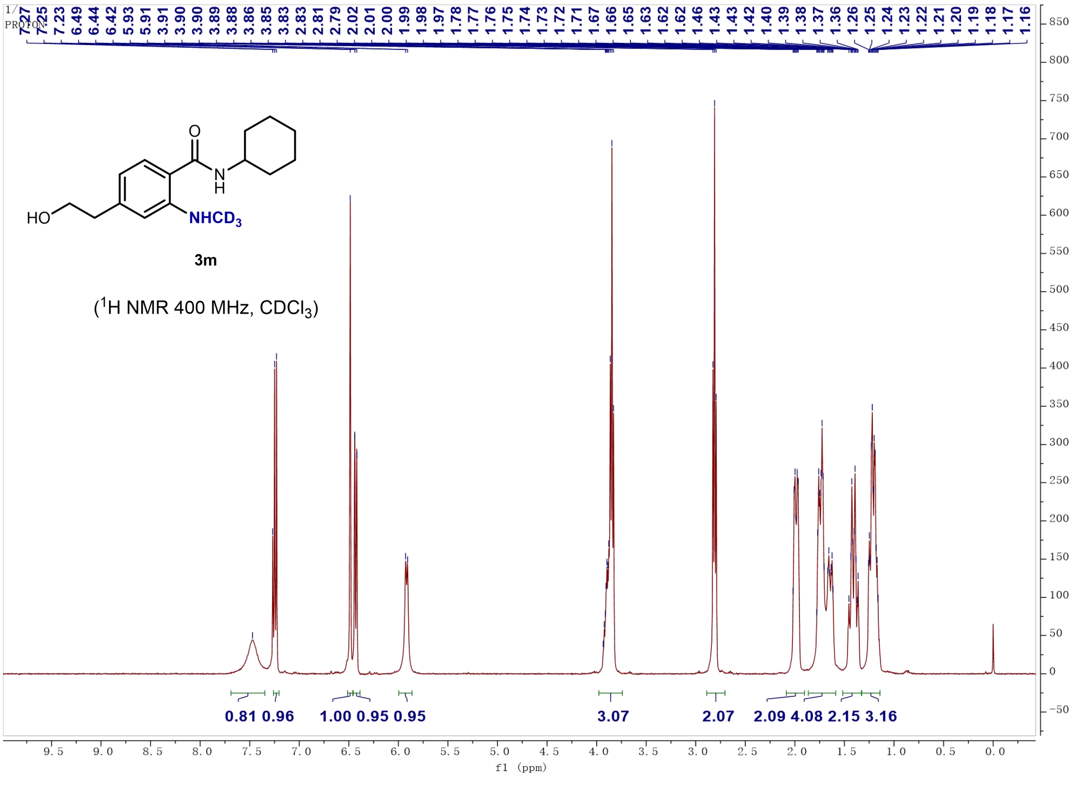
PROTON
7.27
7.25
7.23
6.49
6.44
6.42
5.93
5.91
3.91
3.90
3.89
3.88
3.86
3.85
3.83
2.83
2.81
2.79
2.02
2.01
2.00
1.99
1.98
1.97
1.78
1.77
1.76
1.75
1.74
1.73
1.72
1.71
1.67
1.66
1.65
1.63
1.62
1.46
1.43
1.43
1.42
1.40
1.39
1.38
1.37
1.36
1.26
1.25
1.24
1.23
1.22
1.21
1.20
1.19
1.18
1.17
1.16
O
N
H
HO
NHCD3
3m
(1H NMR 400 MHz, CDCl3)
0.81 0.96 1.00 0.95 0.95 3.07 2.07 2.09 4.08 2.15 3.16
f1 (ppm)

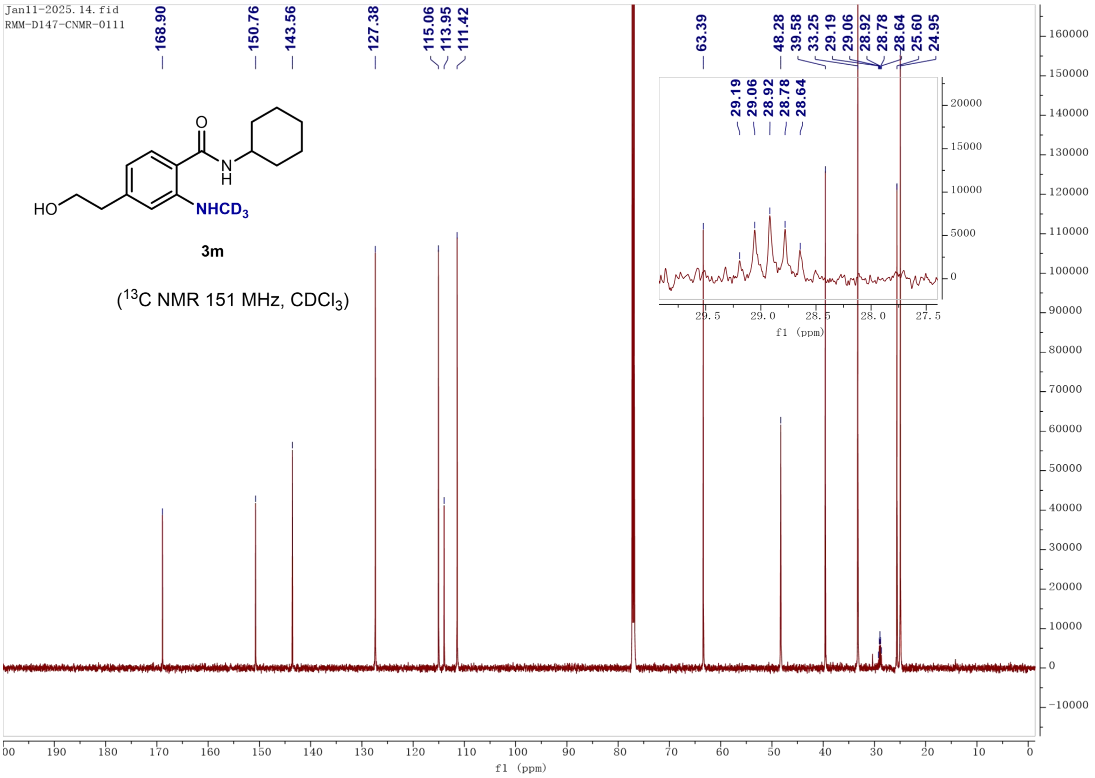
Jan11-2025.14.fid
RMM-D147-CNMR-0111
168.90
150.76
143.56
127.38
115.06
113.95
111.42
63.39
48.28
39.58
33.25
29.19
29.06
28.92
28.78
28.64
25.60
24.95
HO
NHCD3
3m
(13C NMR 151 MHz, CDCl3)
f1 (ppm)

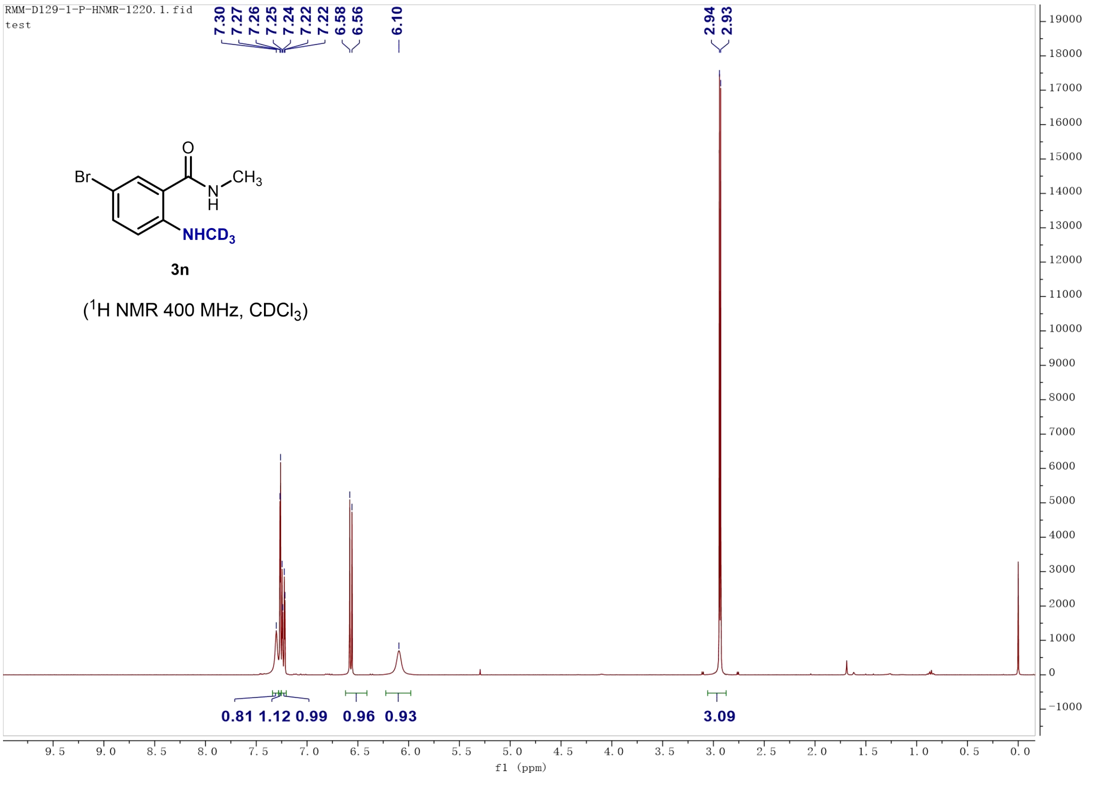
RMM-D129-1-P-HNMR-1220.1.fid
test
7.30
7.27
7.26
7.25
7.24
7.22
7.22
6.58
6.56
6.10
2.94
2.93
Br
CH3
NHCD3
3n
(1H NMR 400 MHz, CDCl3)
0.81 1.12 0.99 0.96 0.93
3.09
f1 (ppm)

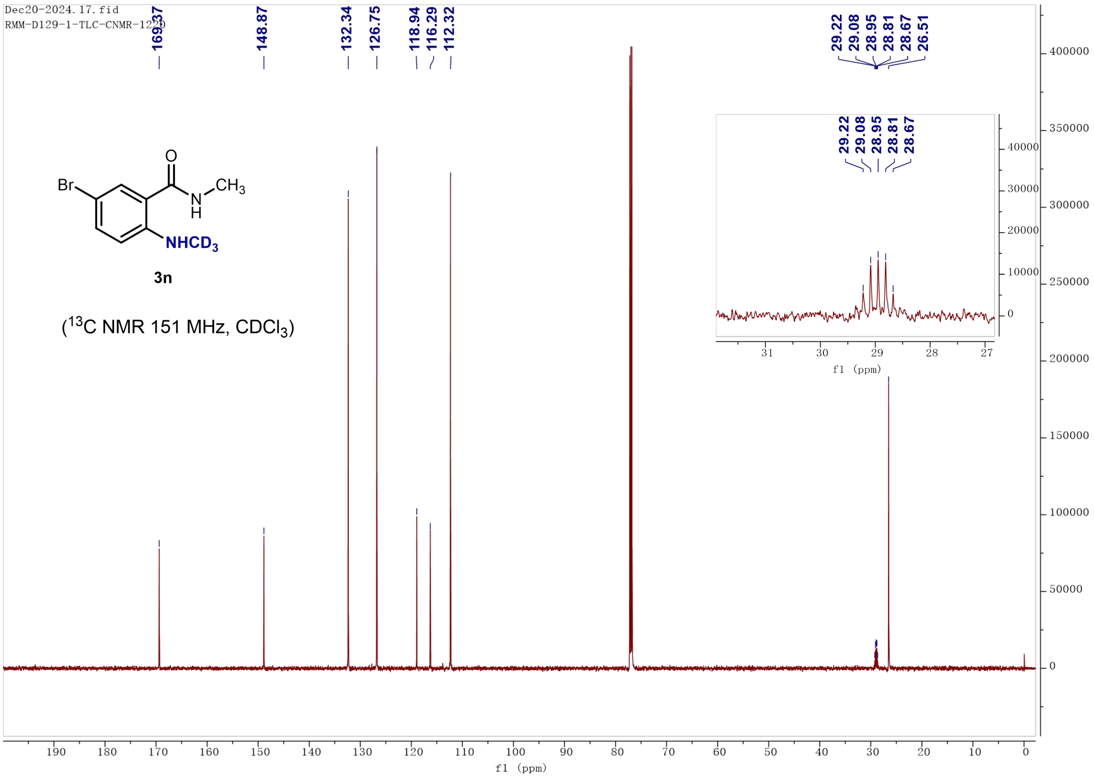
Dec20-2024.17.fid
RMM-D129-1-TLC-CNMR-12[illegible]
169.37
148.87
132.34
126.75
118.94
116.29
112.32
29.22
29.08
28.95
28.81
28.67
26.51
Br
O
N
CH3
H
NHCD3
3n
(13C NMR 151 MHz, CDCl3)
f1 (ppm)

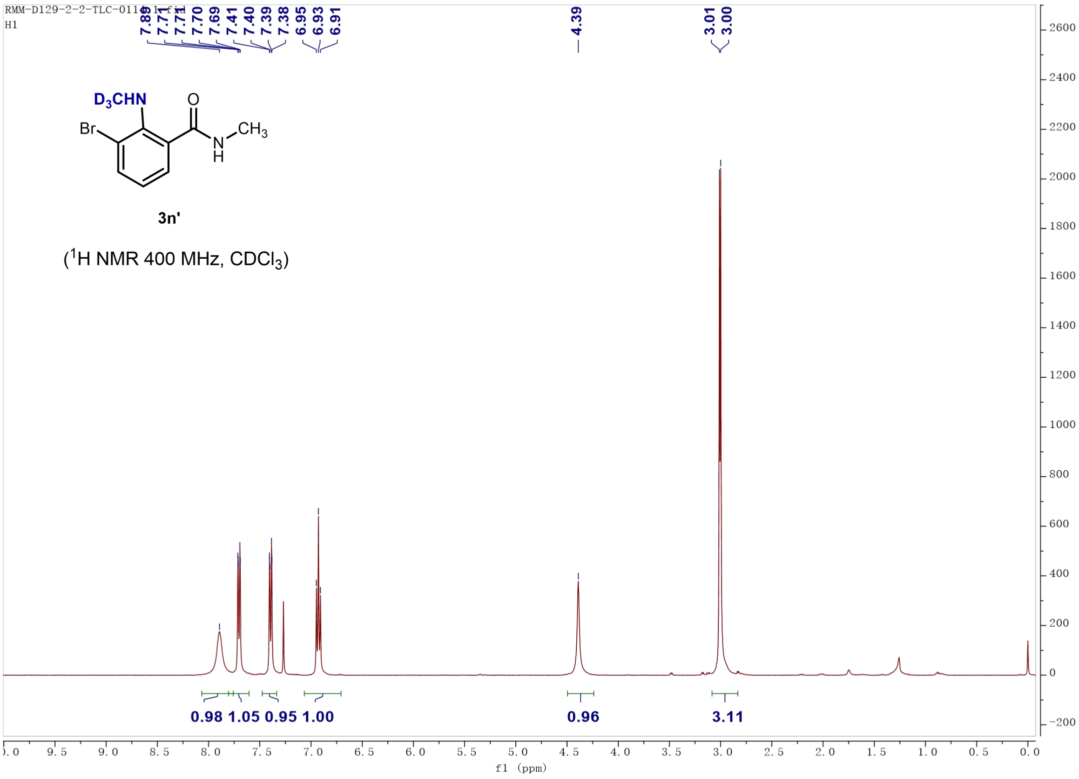
RMM-D129-2-2-TLC-011[illegible]
H1
7.89
7.71
7.71
7.70
7.69
7.41
7.40
7.39
7.38
6.95
6.93
6.91
4.39
3.01
3.00
D3CHN
O
Br
N
CH3
H
3n'
(1H NMR 400 MHz, CDCl3)
0.98
1.05
0.95
1.00
0.96
3.11
f1 (ppm)

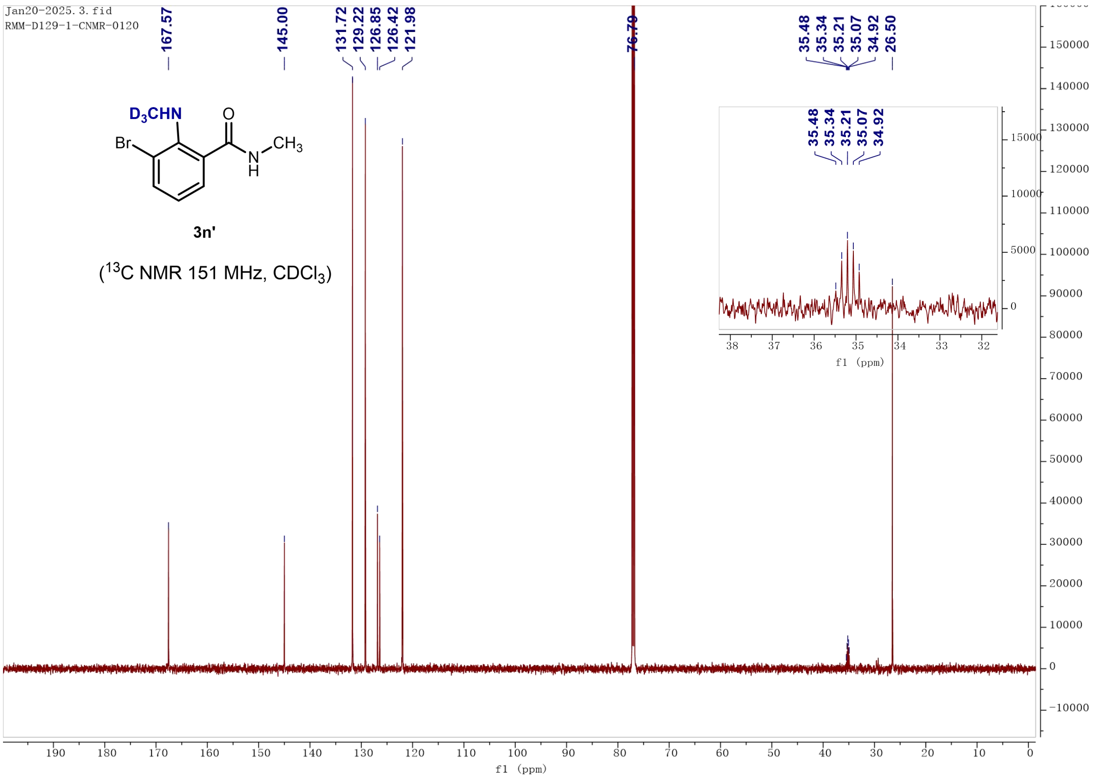
Jan20-2025.3.fid
RMM-D129-1-CNMR-0120
D3CHN
Br
O
N
H
CH3
3n'
(13C NMR 151 MHz, CDCl3)
f1 (ppm)

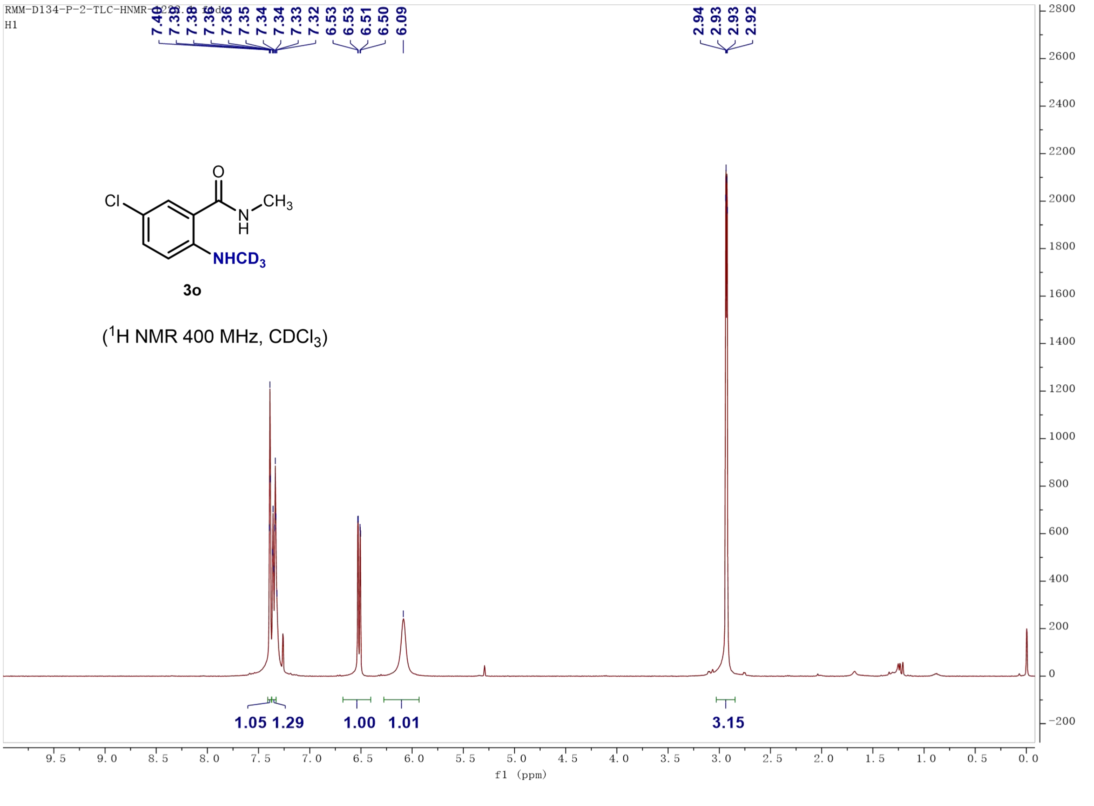
H1
Cl
O
N
H
CH3
NHCD3
3o
(1H NMR 400 MHz, CDCl3)
1.05 1.29
1.00 1.01
3.15
f1 (ppm)

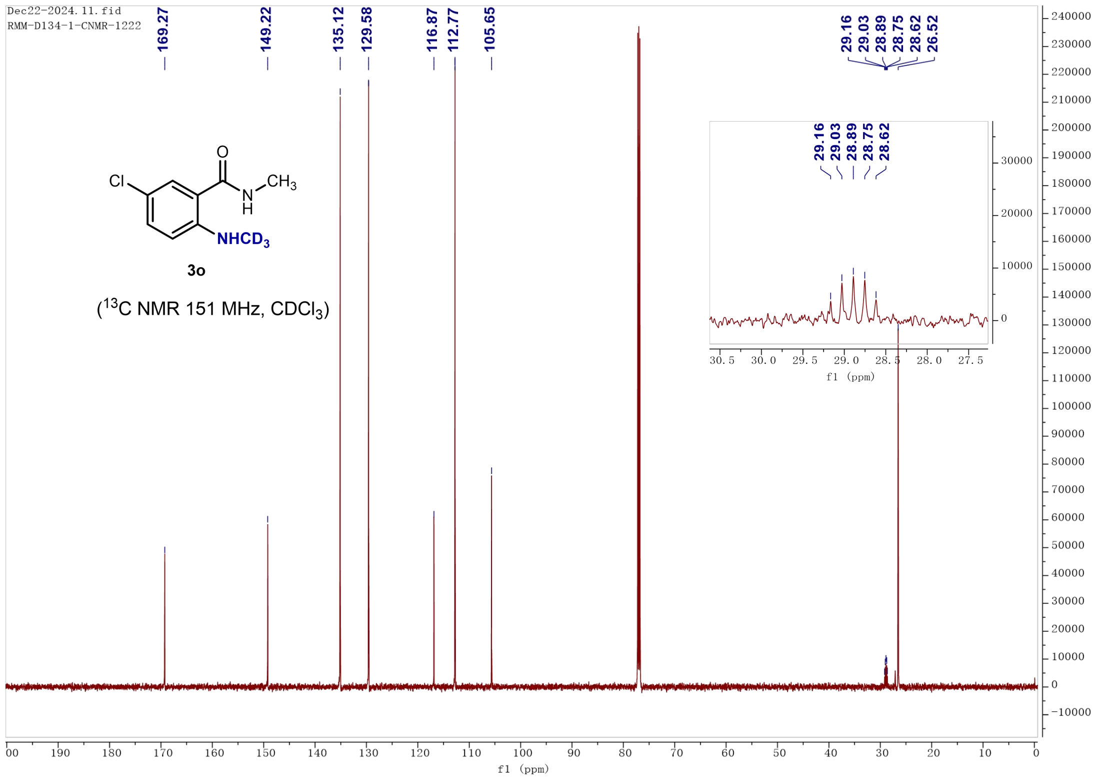
Dec22-2024.11.fid
RMM-D134-1-CNMR-1222
169.27
149.22
135.12
129.58
116.87
112.77
105.65
29.16
29.03
28.89
28.75
28.62
26.52
Cl
O
N
H
CH3
NHCD3
3o
(13C NMR 151 MHz, CDCl3)
f1 (ppm)

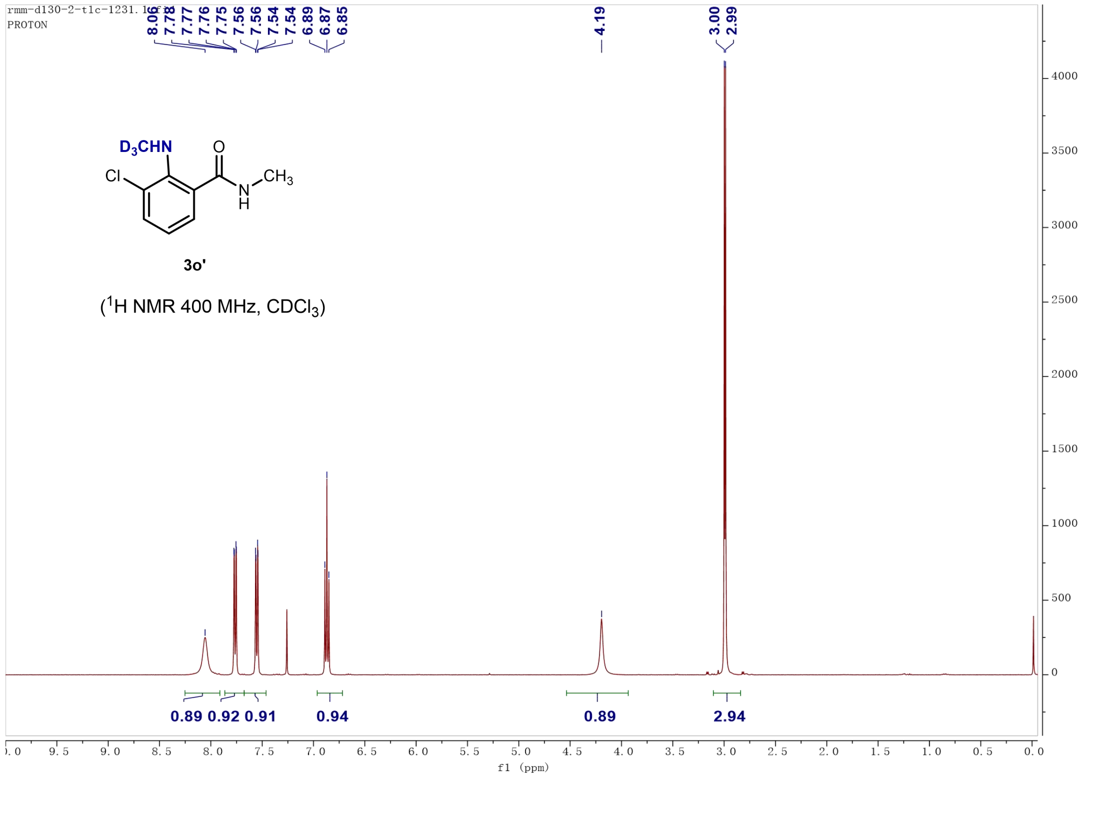
PROTON
8.06
7.78
7.77
7.76
7.75
7.56
7.56
7.54
7.54
6.89
6.87
6.85
4.19
3.00
2.99
D3CHN
O
Cl
N
H
CH3
3o'
(1H NMR 400 MHz, CDCl3)
0.89
0.92
0.91
0.94
0.89
2.94
f1 (ppm)

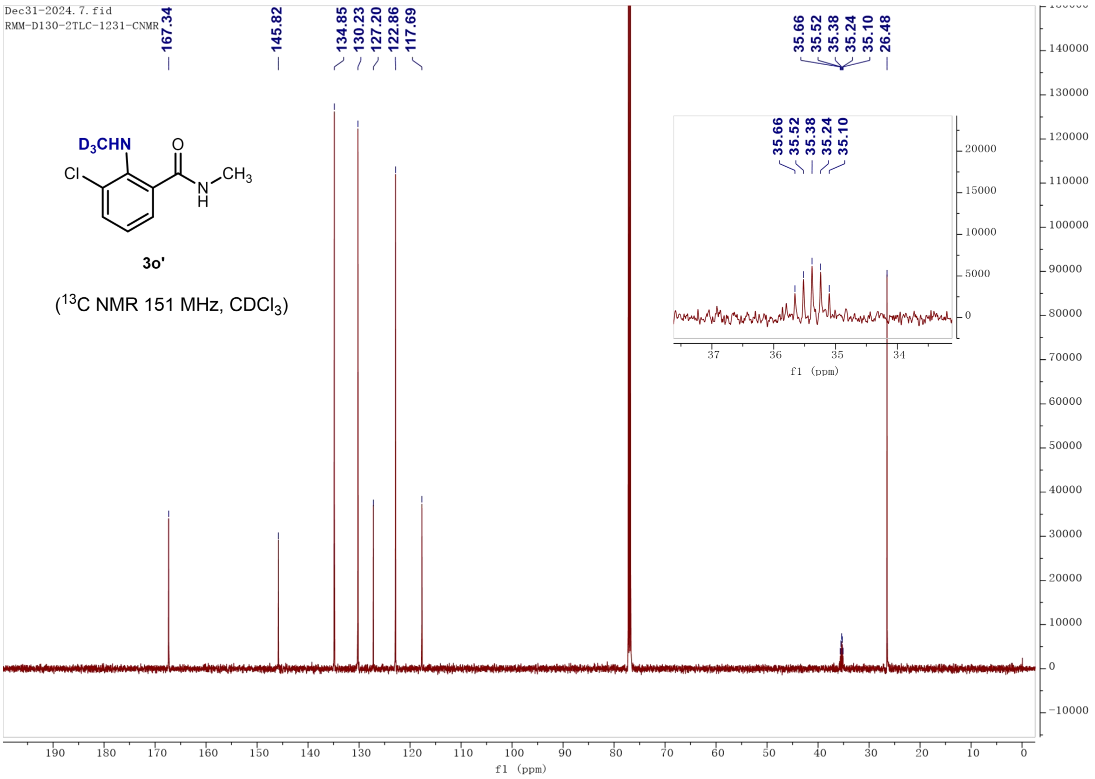
Dec31-2024.7.fid
RMM-D130-2TLC-1231-CNMR
167.34
145.82
134.85
130.23
127.20
122.86
117.69
35.66
35.52
35.38
35.24
35.10
26.48
D3CHN
Cl
O
N
H
CH3
3o'
(13C NMR 151 MHz, CDCl3)
f1 (ppm)

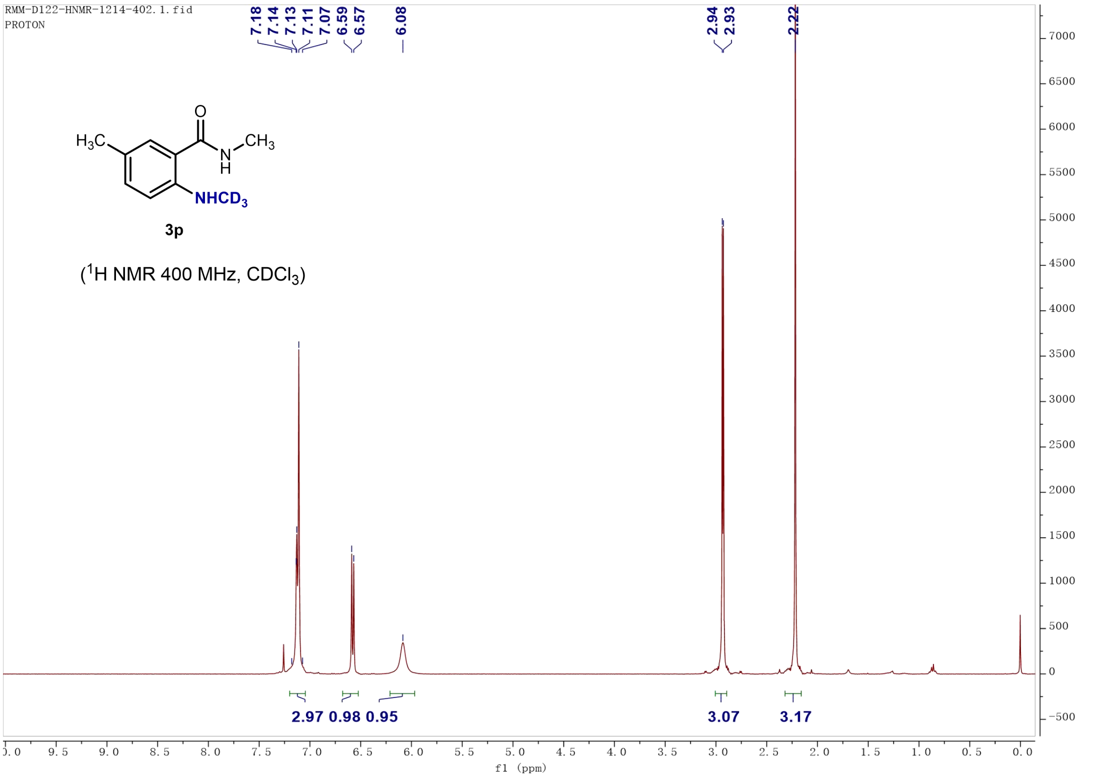
RMM-D122-HNMR-1214-402.1.fid
PROTON
7.18
7.14
7.13
7.11
7.07
6.59
6.57
6.08
2.94
2.93
2.22
H3C
O
N
H
CH3
NHCD3
3p
(1H NMR 400 MHz, CDCl3)
2.97
0.98
0.95
3.07
3.17
f1 (ppm)

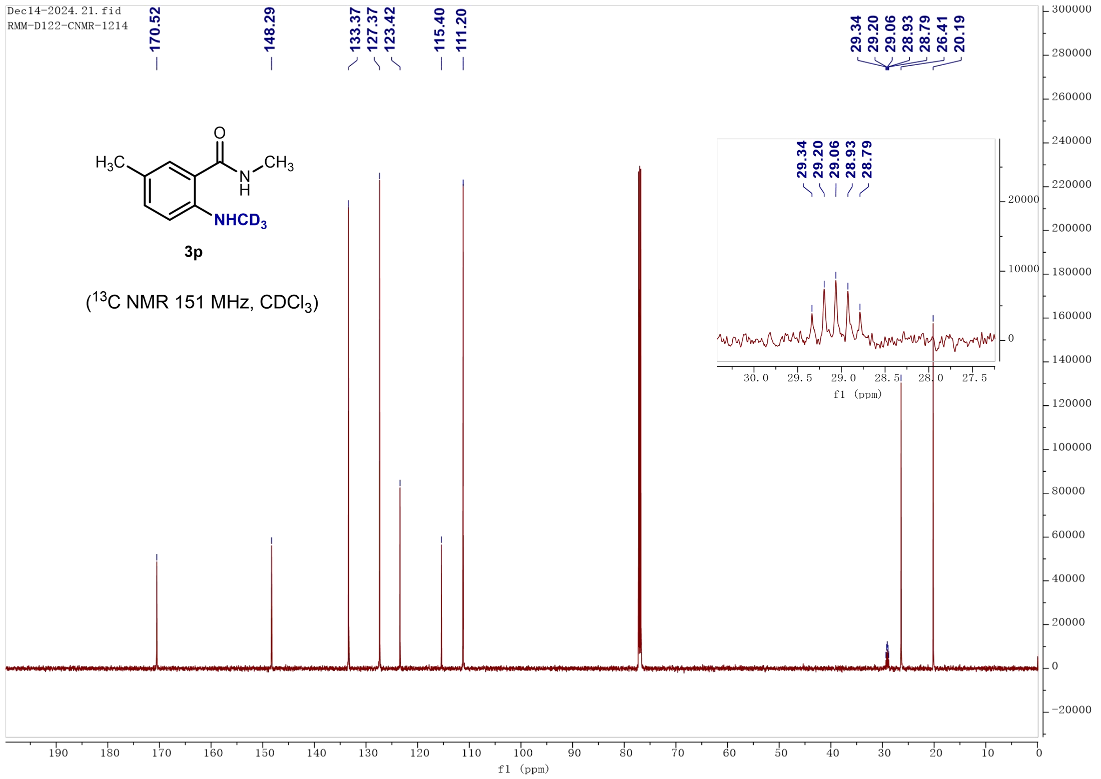
Dec14-2024.21.fid
RMM-D122-CNMR-1214
170.52
148.29
133.37
127.37
123.42
115.40
111.20
29.34
29.20
29.06
28.93
28.79
26.41
20.19
3p
(^{13}C NMR 151 MHz, CDCl$_3$)
f1 (ppm)

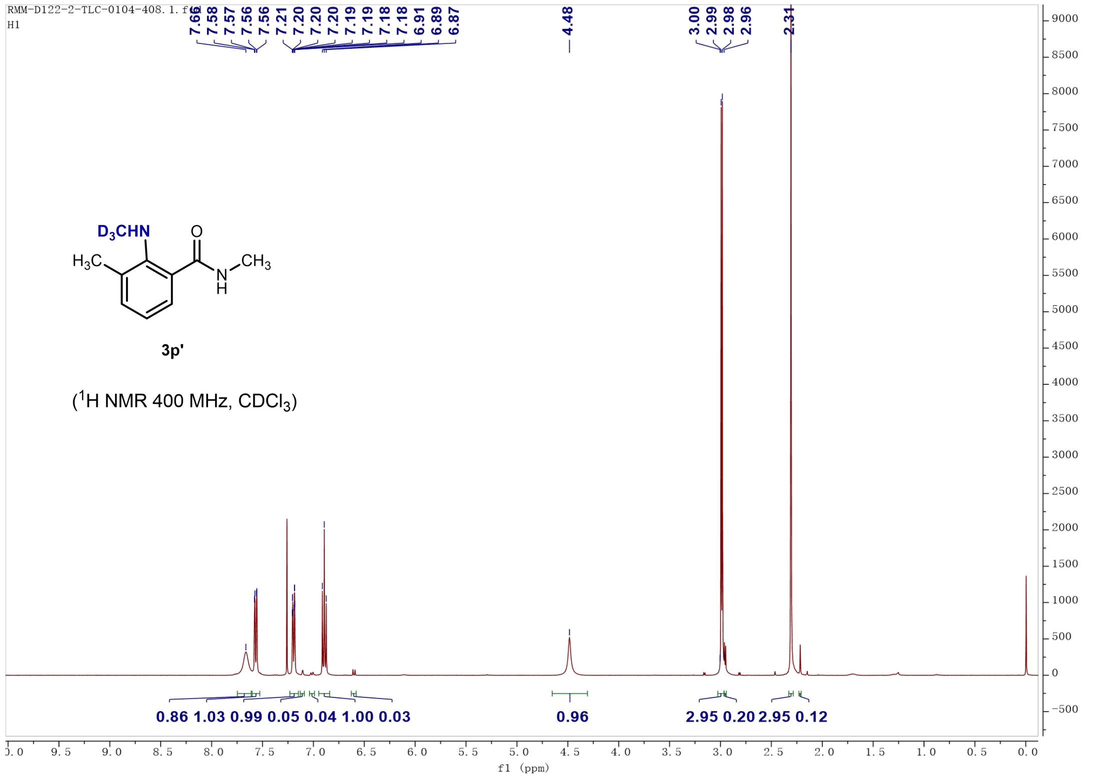
RMM-D122-2-TLC-0104-408.1.fid
H1
7.66
7.58
7.57
7.56
7.56
7.21
7.20
7.20
7.20
7.19
7.19
7.18
7.18
6.91
6.89
6.87
4.48
3.00
2.99
2.98
2.96
2.31
3p'
(^{1}H NMR 400 MHz, CDCl$_3$)
0.86 1.03 0.99 0.05 0.04 1.00 0.03
0.96
2.95 0.20 2.95 0.12
f1 (ppm)

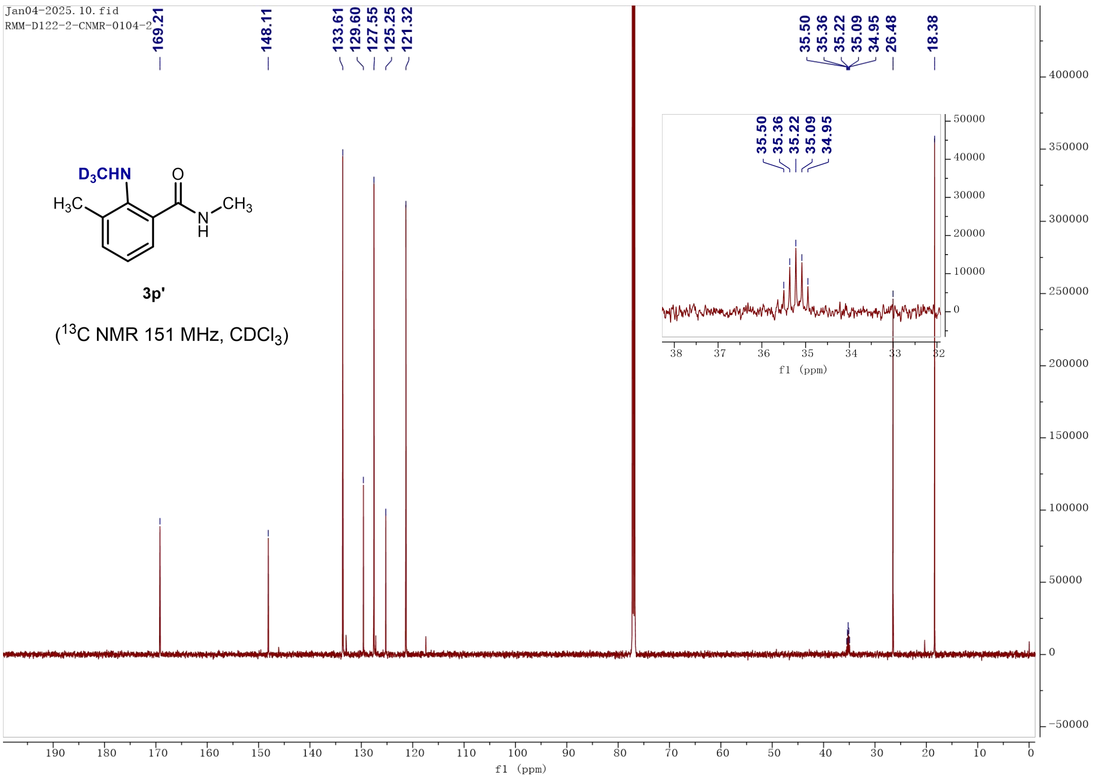
Jan04-2025.10.fid
RMM-D122-2-CNMR-0104-2
169.21
148.11
133.61
129.60
127.55
125.25
121.32
35.50
35.36
35.22
35.09
34.95
26.48
18.38
D3CHN
H3C
O
N
H
CH3
3p'
(13C NMR 151 MHz, CDCl3)
f1 (ppm)

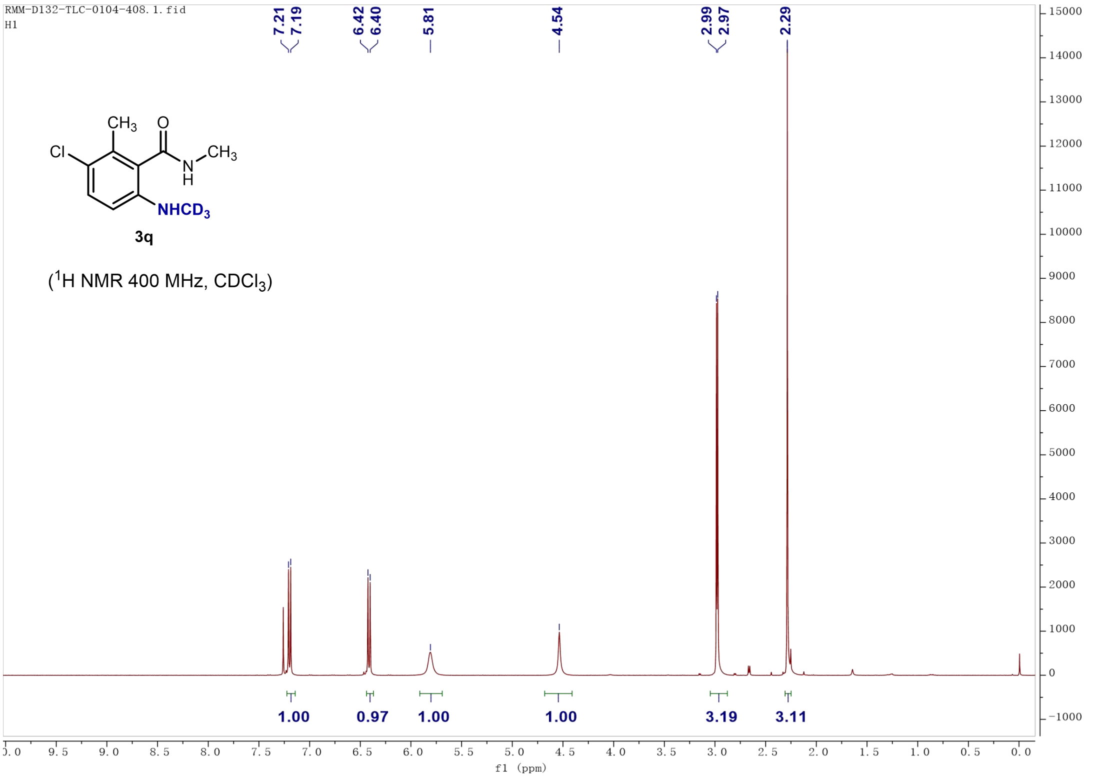
RMM-D132-TLC-0104-408.1.fid
H1
7.21
7.19
6.42
6.40
5.81
4.54
2.99
2.97
2.29
CH3
O
Cl
N
H
CH3
NHCD3
3q
(1H NMR 400 MHz, CDCl3)
1.00
0.97
1.00
1.00
3.19
3.11
f1 (ppm)

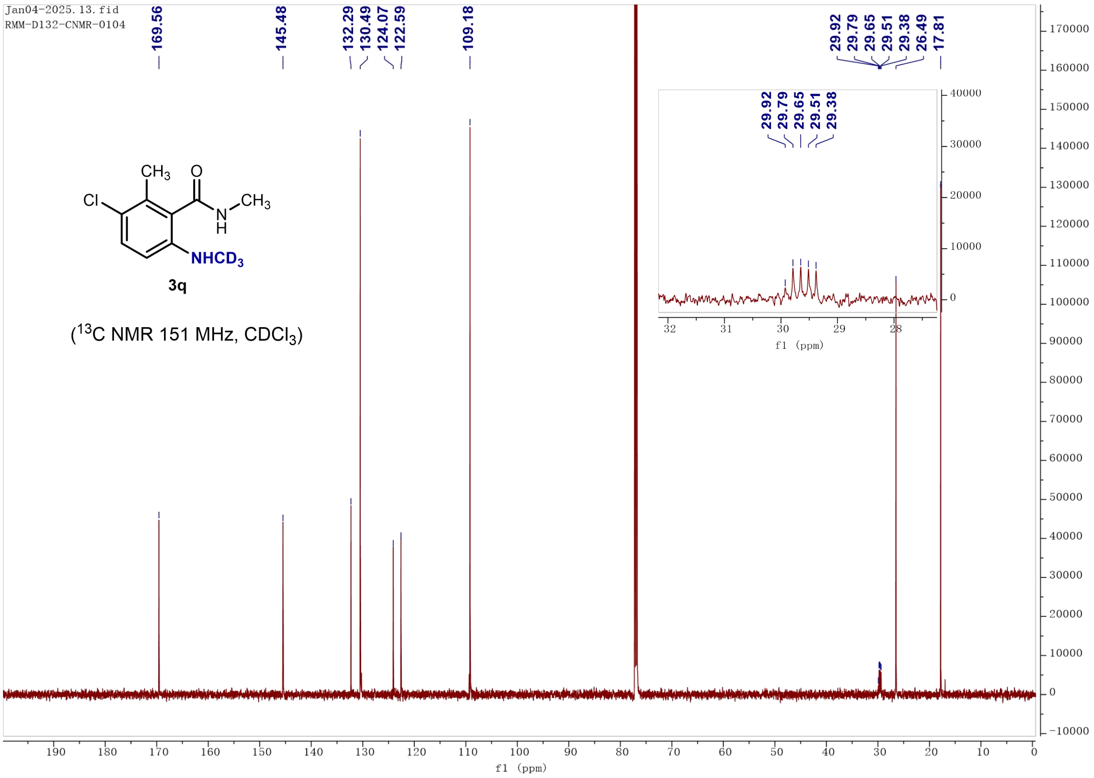
Jan04-2025.13.fid
RMM-D132-CNMR-0104
169.56
145.48
132.29
130.49
124.07
122.59
109.18
29.92
29.79
29.65
29.51
29.38
26.49
17.81
3q
(13C NMR 151 MHz, CDCl3)
f1 (ppm)

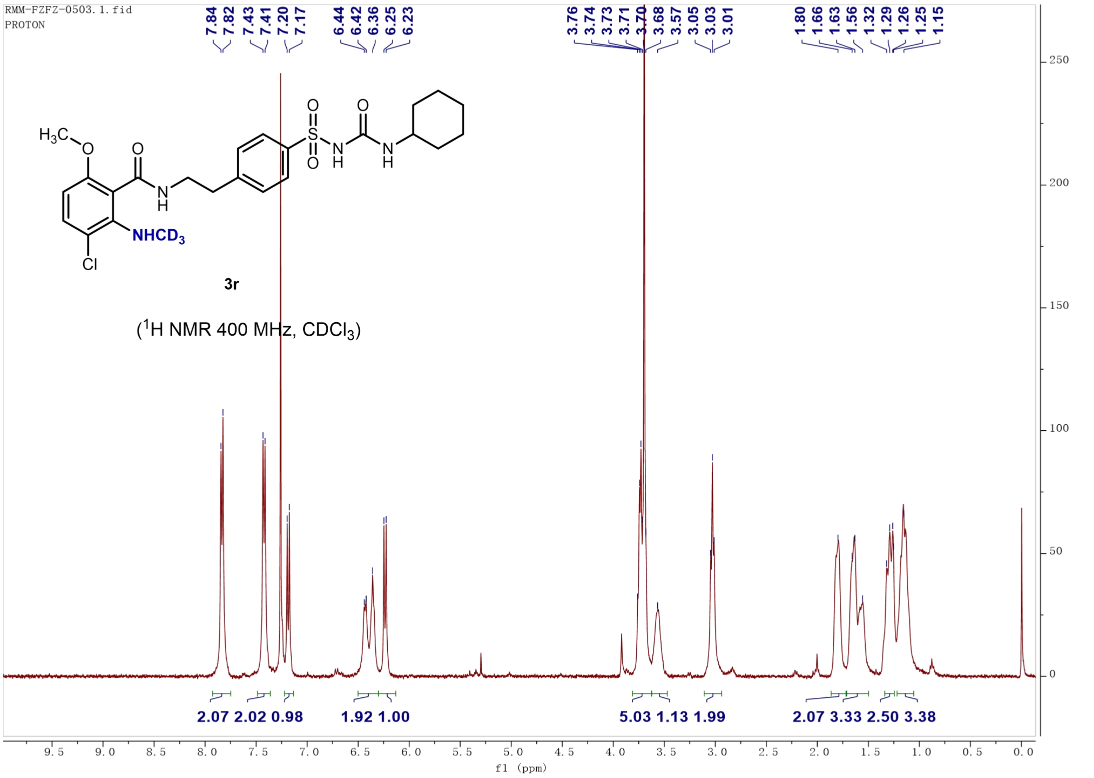
RMM-FZFZ-0503.1.fid
PROTON
7.84
7.82
7.43
7.41
7.20
7.17
6.44
6.42
6.36
6.25
6.23
3.76
3.74
3.73
3.71
3.70
3.68
3.57
3.05
3.03
3.01
1.80
1.66
1.63
1.56
1.32
1.29
1.26
1.25
1.15
3r
(1H NMR 400 MHz, CDCl3)
2.07 2.02 0.98
1.92 1.00
5.03 1.13 1.99
2.07 3.33 2.50 3.38
f1 (ppm)

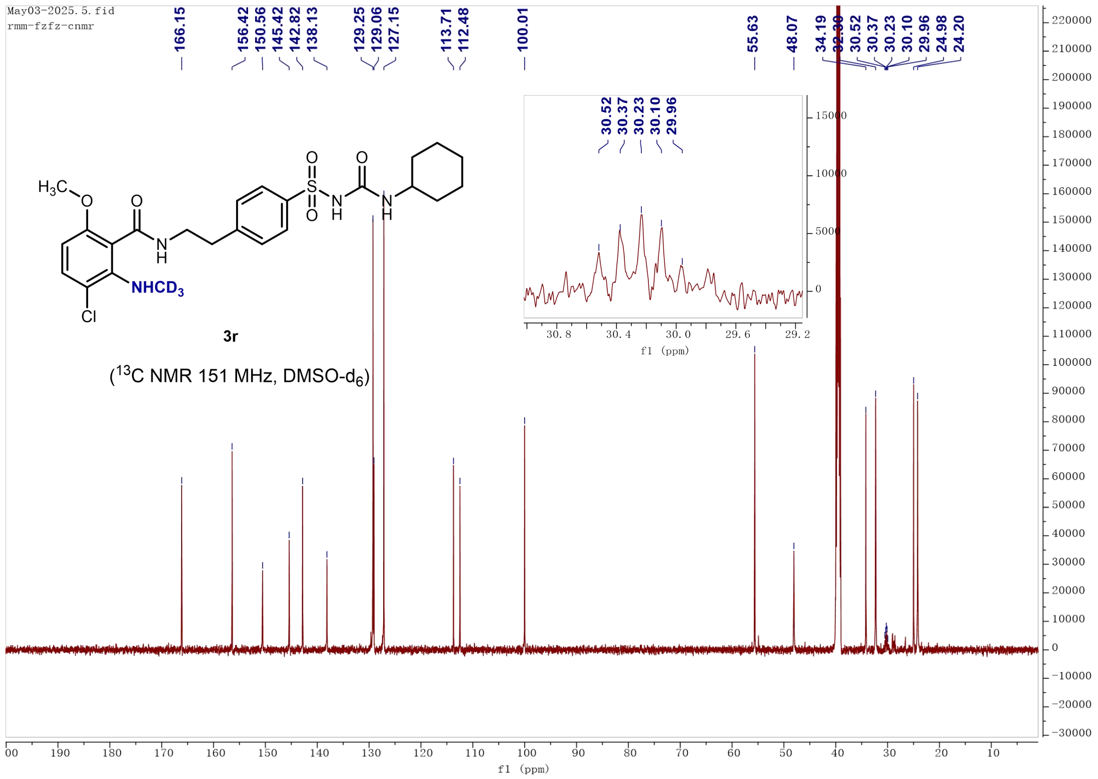
3r
(¹³C NMR 151 MHz, DMSO-d6)

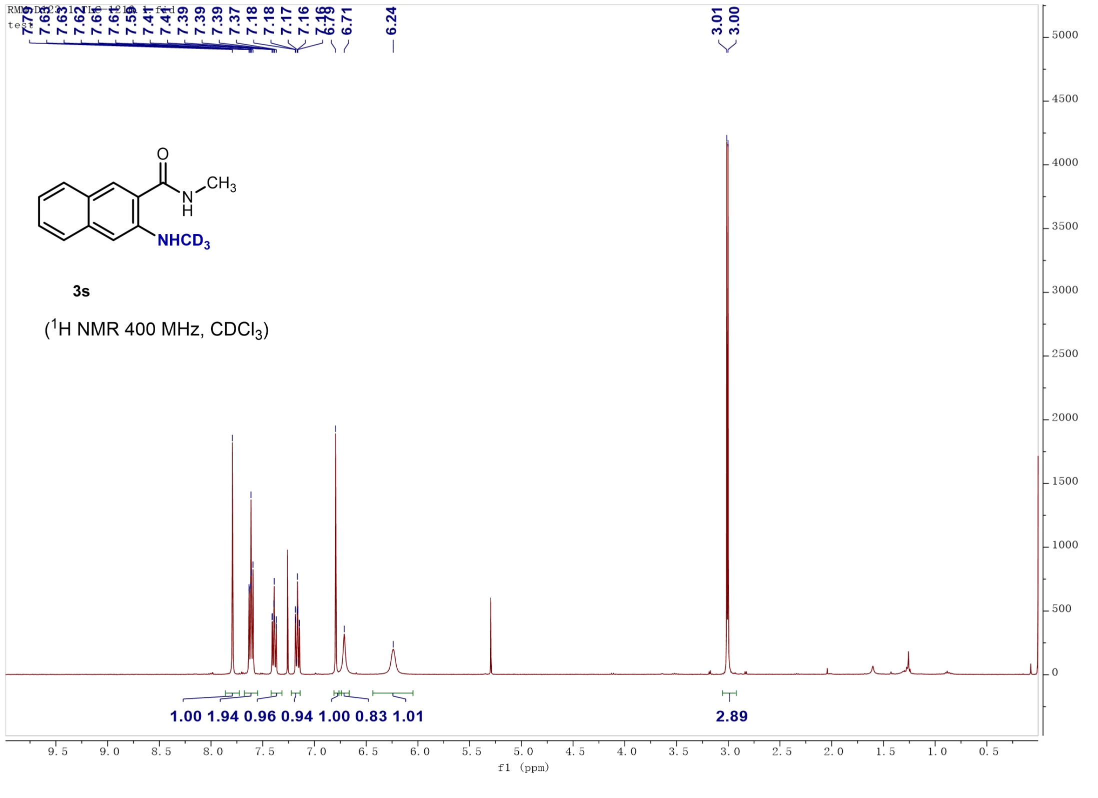
3s
(¹H NMR 400 MHz, CDCl3)

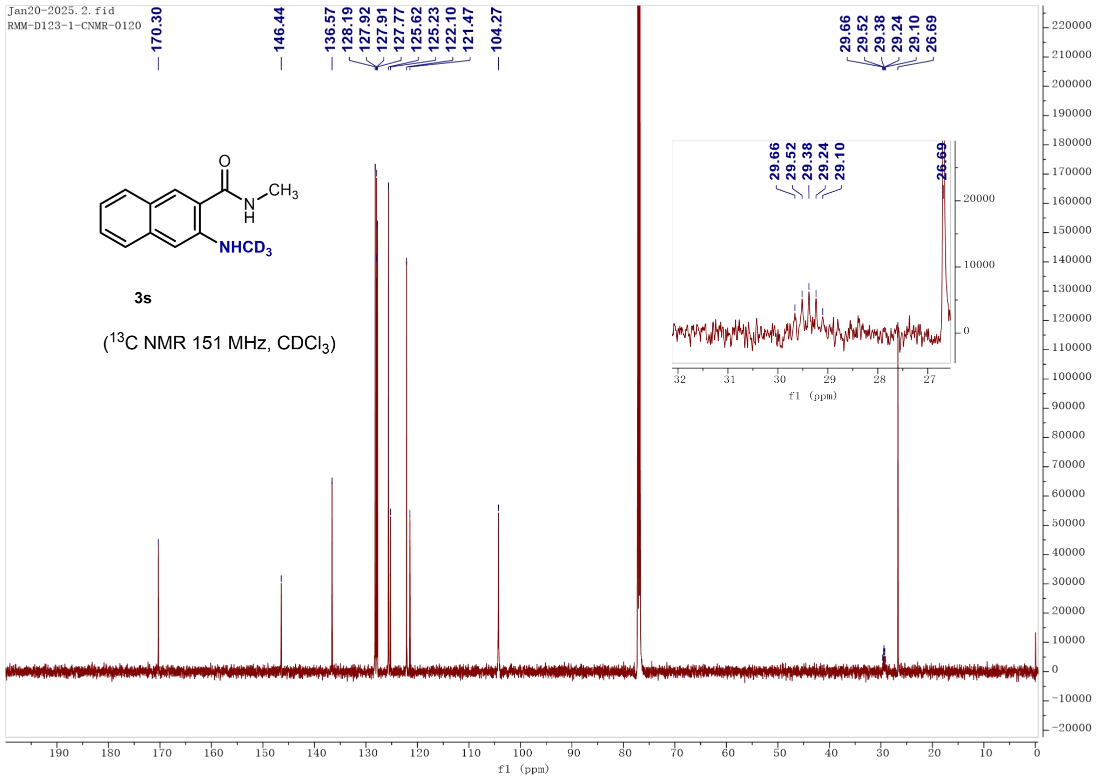
Jan20-2025.2.fid
RMM-D123-1-CNMR-0120
170.30
146.44
136.57
128.19
127.92
127.91
127.77
125.62
125.23
122.10
121.47
104.27
29.66
29.52
29.38
29.24
29.10
26.69
NHCD3
3s
(13C NMR 151 MHz, CDCl3)
f1 (ppm)

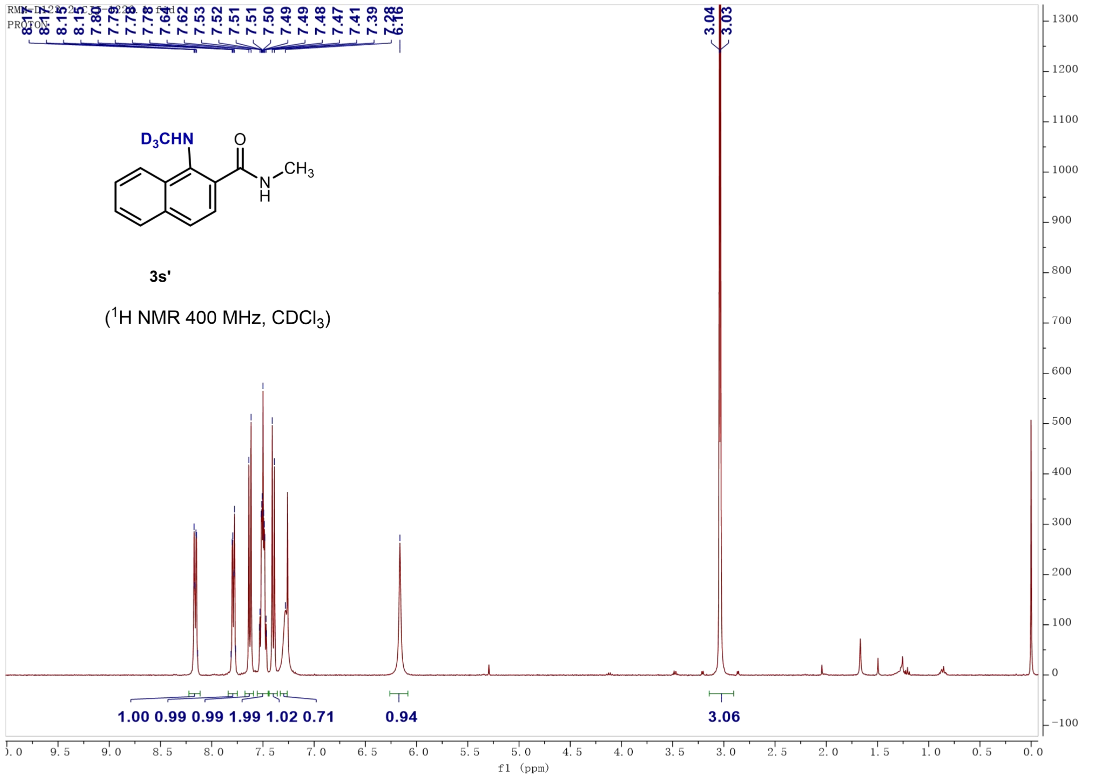
PROTON
8.15
7.53
7.52
7.51
7.50
7.49
7.48
7.47
7.41
7.39
6.16
3.04
3.03
D3CHN
3s'
(1H NMR 400 MHz, CDCl3)
1.00 0.99 0.99 1.99 1.02 0.71
0.94
3.06
f1 (ppm)

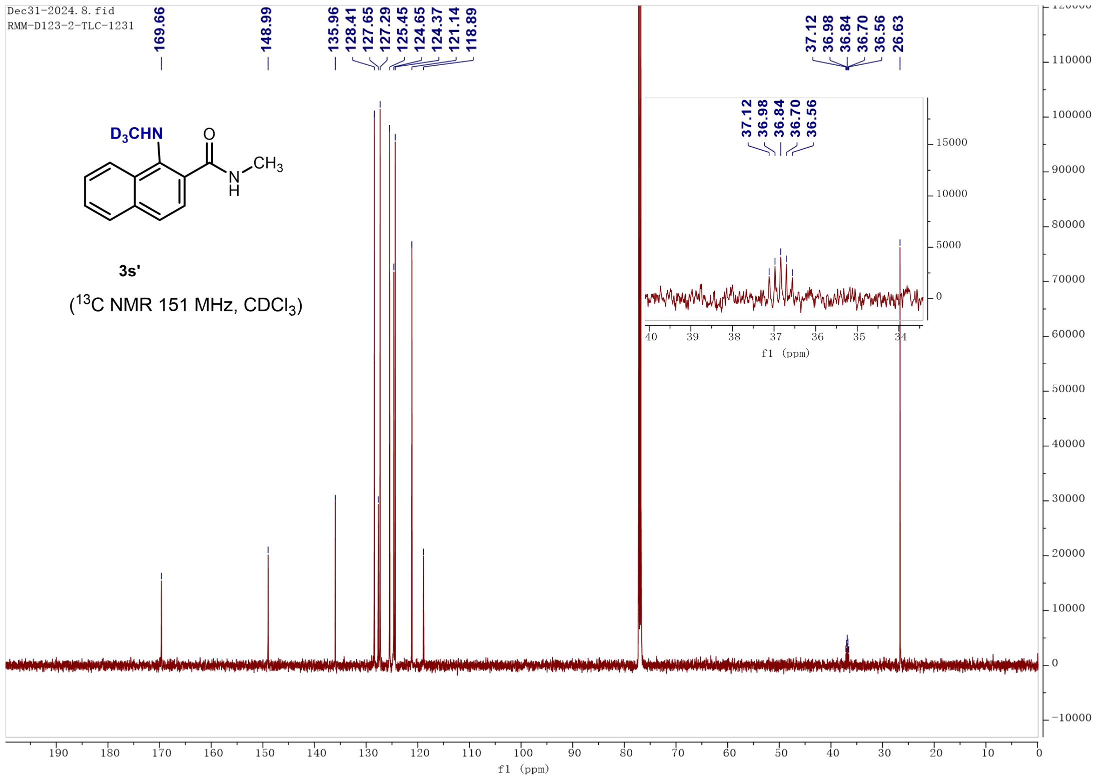

Dec31-2024.8.fid
RMM-D123-2-TLC-1231
169.66
148.99
135.96
128.41
127.65
127.29
125.45
124.65
124.37
121.14
118.89
37.12
36.98
36.84
36.70
36.56
26.63
D3CHN
O
N
H
CH3
3s'
(13C NMR 151 MHz, CDCl3)
f1 (ppm)

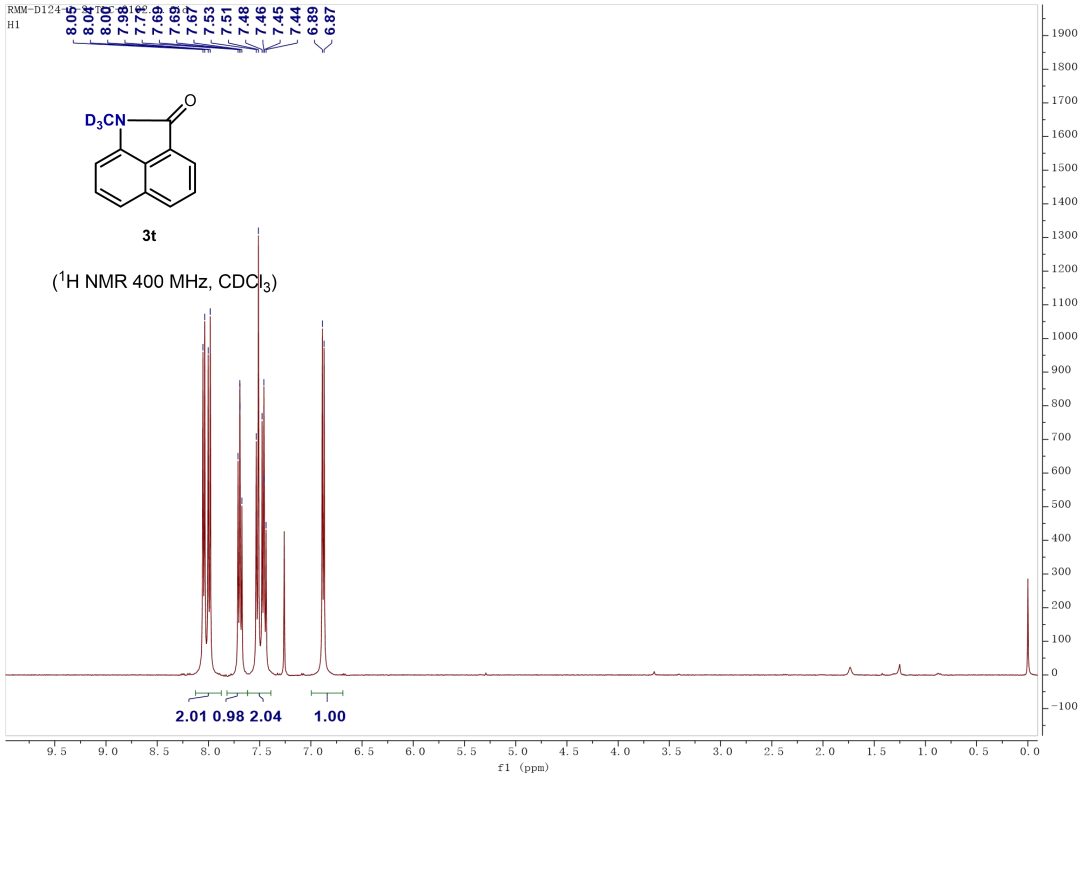

H1
8.05
8.04
8.00
7.98
7.71
7.69
7.69
7.67
7.53
7.51
7.48
7.46
7.45
7.44
6.89
6.87
D3CN
O
3t
(1H NMR 400 MHz, CDCl3)
2.01
0.98
2.04
1.00
f1 (ppm)

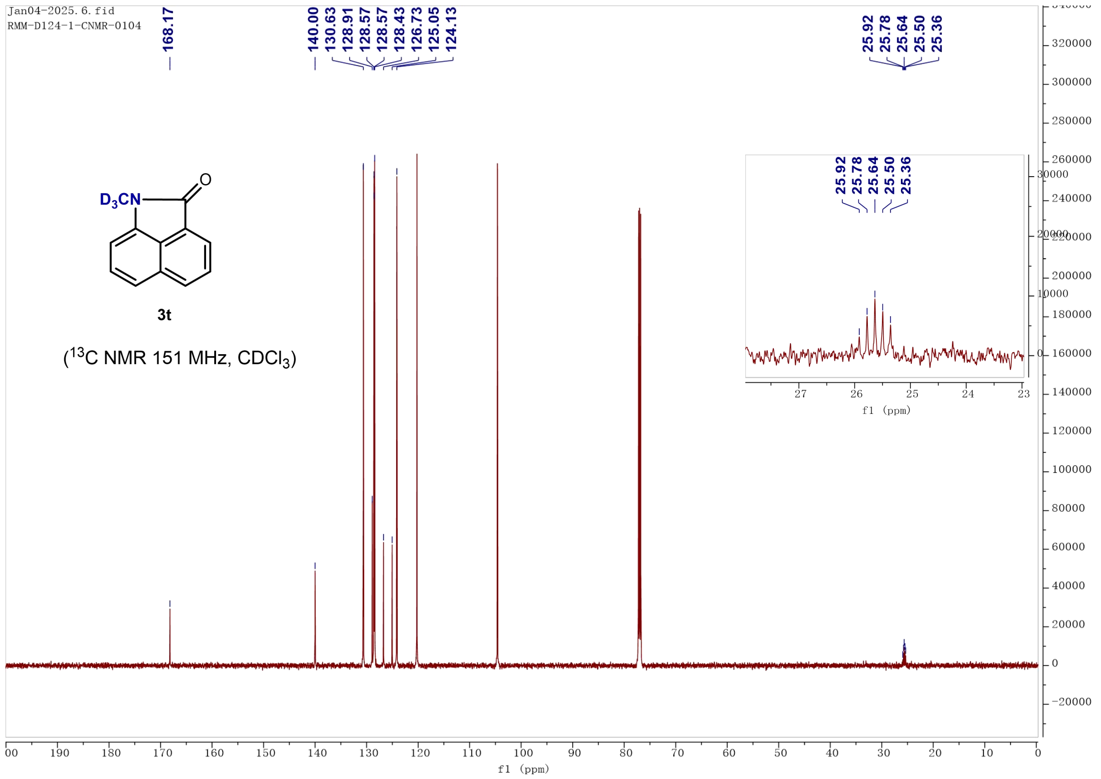
Jan04-2025.6.fid
RMM-D124-1-CNMR-0104
168.17
140.00
130.63
128.91
128.57
128.57
128.43
126.73
125.05
124.13
25.92
25.78
25.64
25.50
25.36
D3CN
O
3t
(13C NMR 151 MHz, CDCl3)
f1 (ppm)

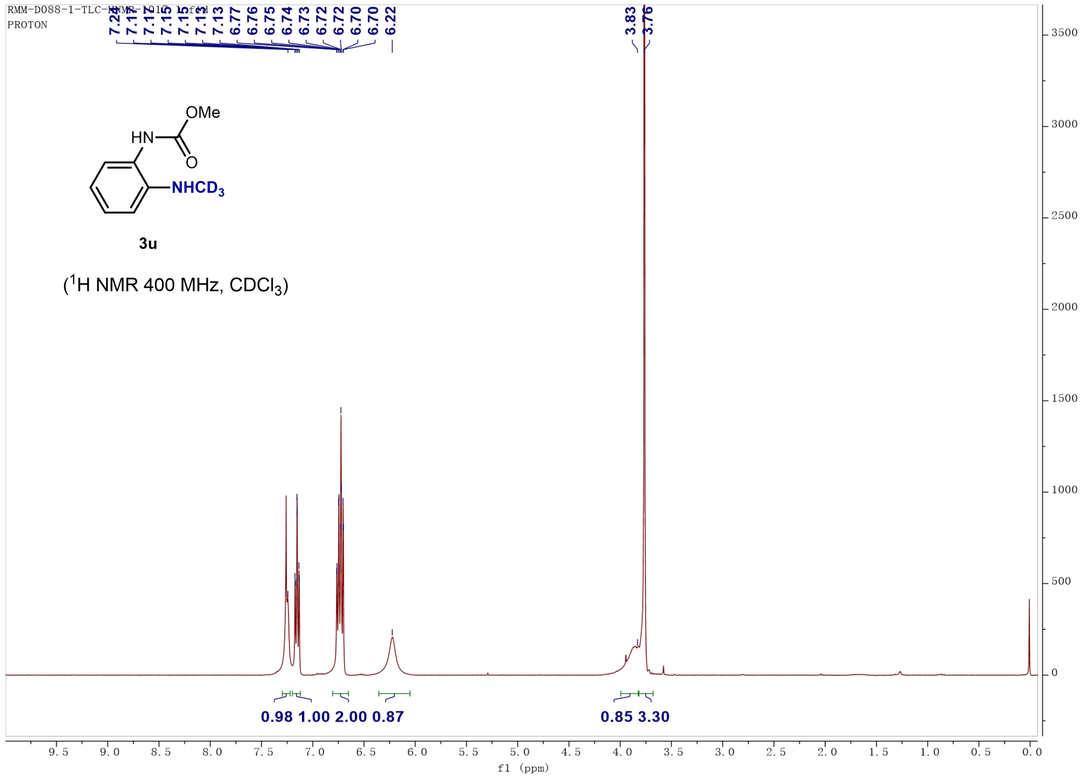
PROTON
7.24
7.17
7.16
7.15
7.15
7.13
7.13
6.77
6.76
6.75
6.74
6.73
6.72
6.72
6.70
6.70
6.22
3.83
3.76
OMe
HN
O
NHCD3
3u
(1H NMR 400 MHz, CDCl3)
0.98 1.00 2.00 0.87
0.85 3.30
f1 (ppm)

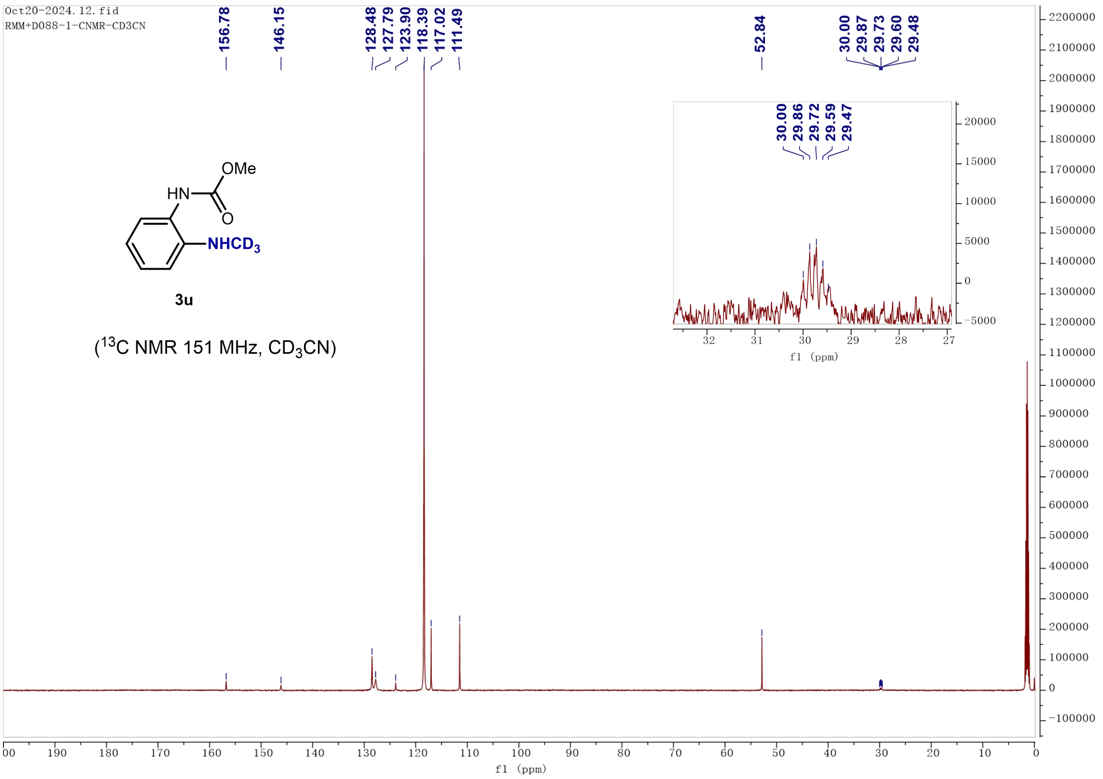
Oct20-2024.12.fid
RMM+D088-1-CNMR-CD3CN
156.78
146.15
128.48
127.79
123.90
118.39
117.02
111.49
52.84
30.00
29.87
29.73
29.60
29.48
OMe
HN
O
NHCD3
3u
(13C NMR 151 MHz, CD3CN)
f1 (ppm)

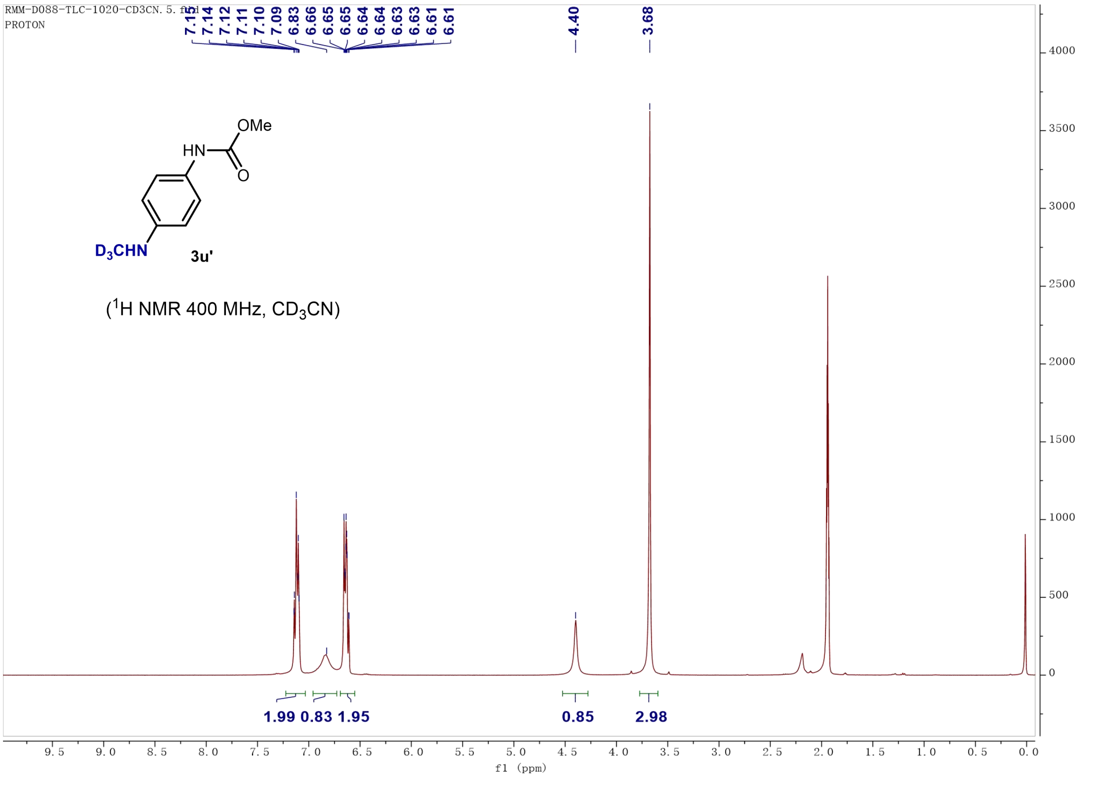
RMM-D088-TLC-1020-CD3CN.5.fid
PROTON
7.15
7.14
7.12
7.11
7.10
7.09
6.83
6.66
6.65
6.64
6.64
6.63
6.61
6.61
4.40
3.68
OMe
HN
O
D3CHN
3u'
(1H NMR 400 MHz, CD3CN)
1.99 0.83 1.95
0.85
2.98
f1 (ppm)

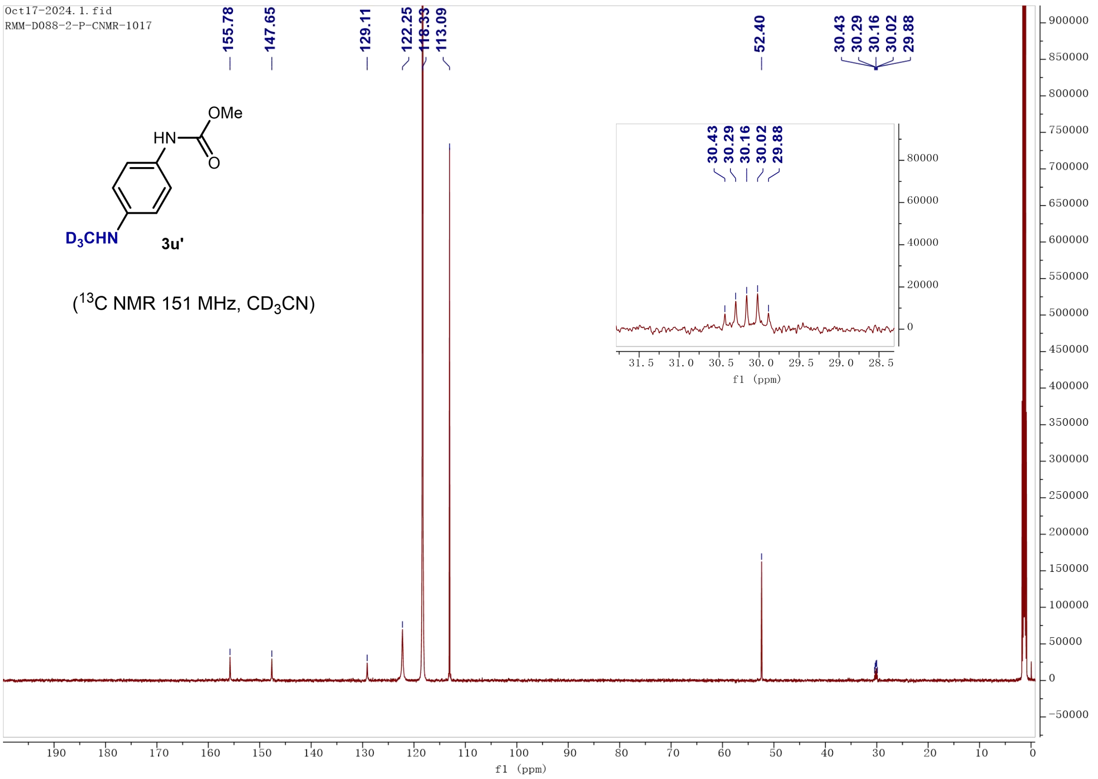
Oct17-2024.1.fid
RMM-D088-2-P-CNMR-1017
155.78
147.65
129.11
122.25
118.33
113.09
52.40
30.43
30.29
30.16
30.02
29.88
OMe
HN
O
D3CHN
3u'
(13C NMR 151 MHz, CD3CN)
f1 (ppm)

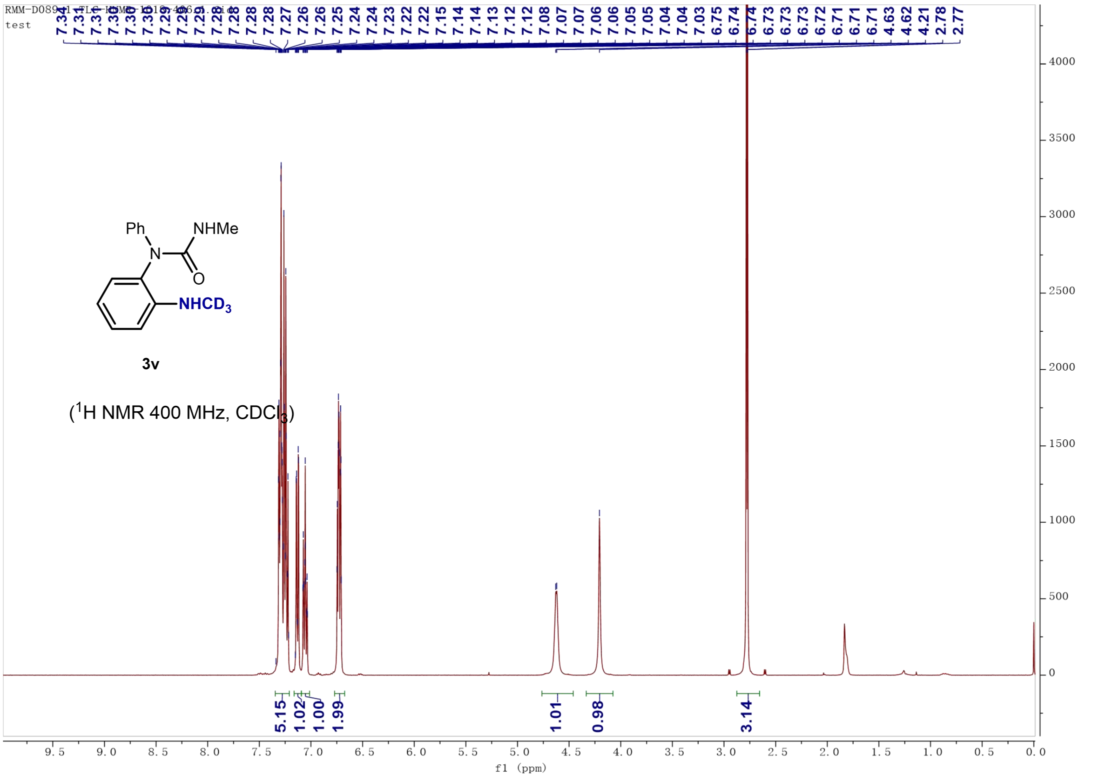
test
Ph
NHMe
N
O
NHCD3
3v
(1H NMR 400 MHz, CDCl3)
5.15
1.02
1.00
1.99
1.01
0.98
3.14
f1 (ppm)

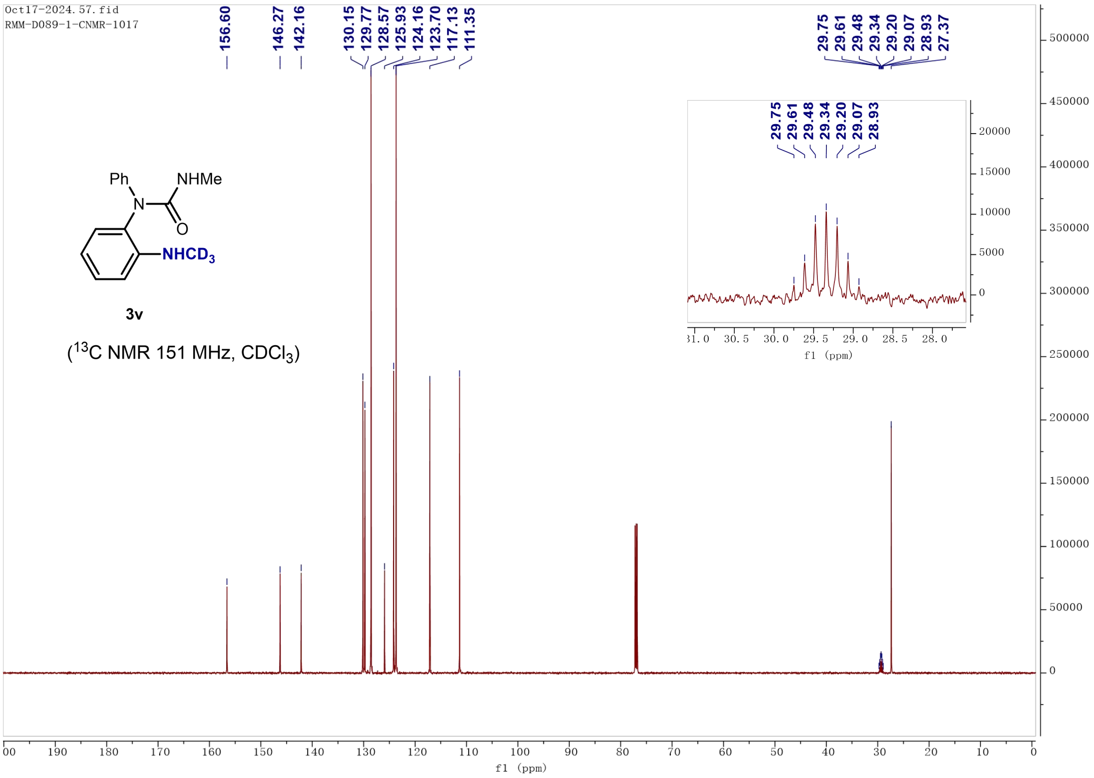
Oct17-2024.57.fid
RMM-D089-1-CNMR-1017
156.60
146.27
142.16
130.15
129.77
128.57
125.93
124.16
123.70
117.13
111.35
29.75
29.61
29.48
29.34
29.20
29.07
28.93
27.37
Ph
NHMe
N
O
NHCD3
3v
(13C NMR 151 MHz, CDCl3)
f1 (ppm)

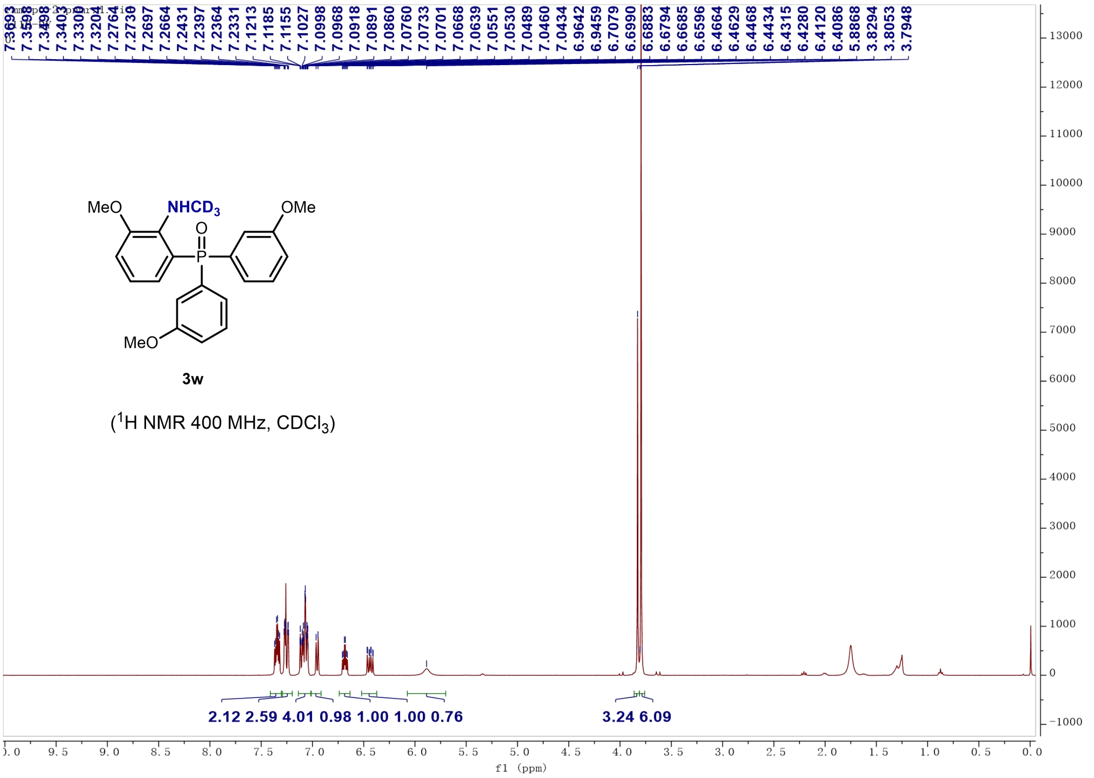
MeO
NHCD3
OMe
O
P
MeO
3w
(1H NMR 400 MHz, CDCl3)
2.12 2.59 4.01 0.98 1.00 1.00 0.76
3.24 6.09
f1 (ppm)

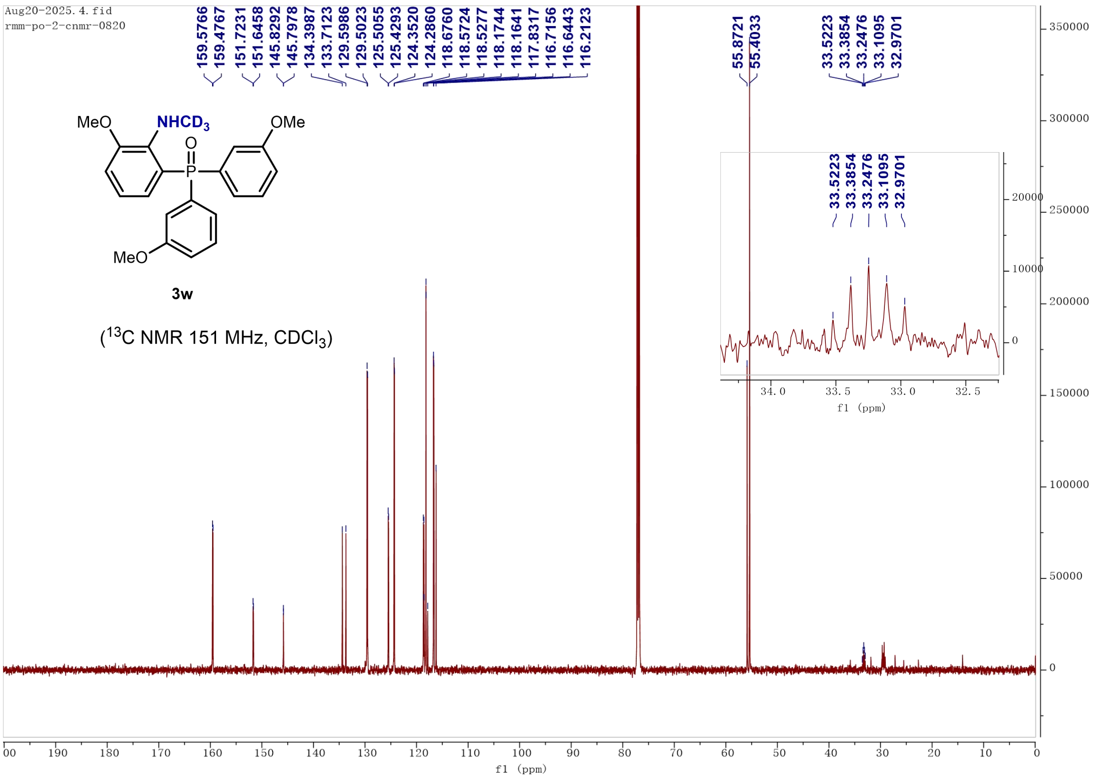
Aug20-2025.4.fid
rmm-po-2-cnmr-0820
159.5766
159.4767
151.7231
151.6458
145.8292
145.7978
134.3987
133.7123
129.5986
129.5023
125.5055
125.4293
124.3520
124.2860
118.6760
118.5724
118.5277
118.1744
118.1641
117.8317
116.7156
116.6443
116.2123
55.8721
55.4033
33.5223
33.3854
33.2476
33.1095
32.9701
MeO
NHCD3
OMe
MeO
3w
(13C NMR 151 MHz, CDCl3)
f1 (ppm)

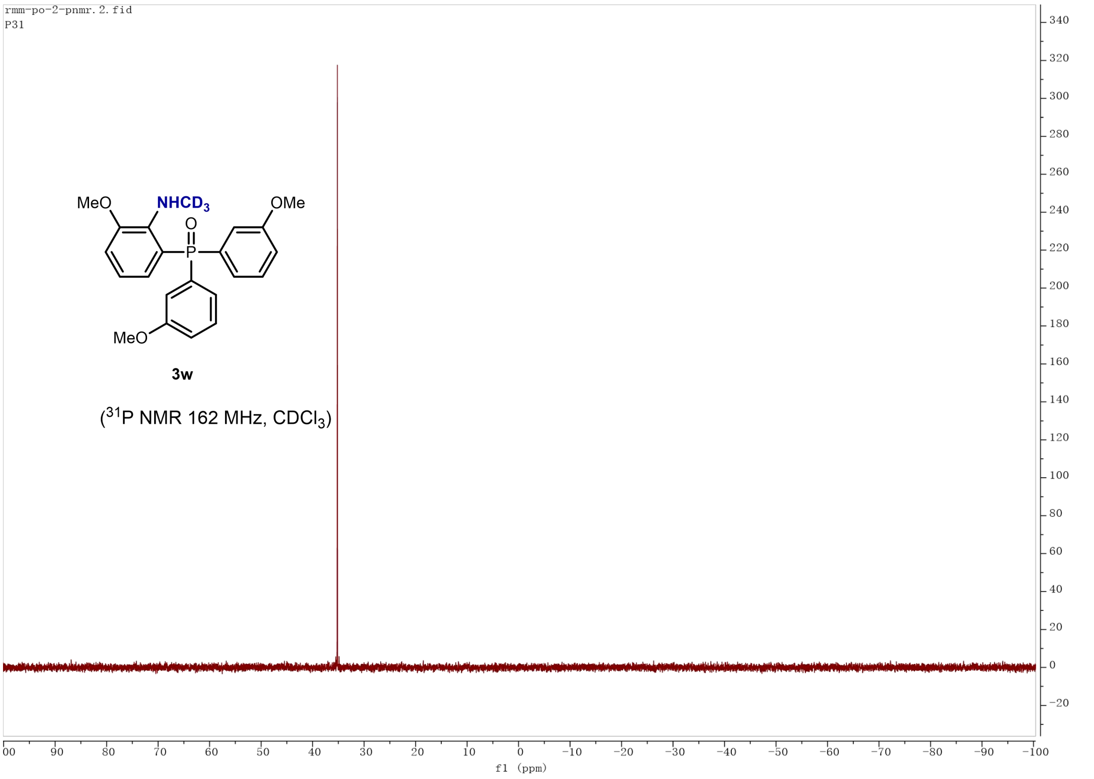
rmm-po-2-pnmr.2.fid
P31
MeO
NHCD3
OMe
MeO
3w
(31P NMR 162 MHz, CDCl3)
f1 (ppm)

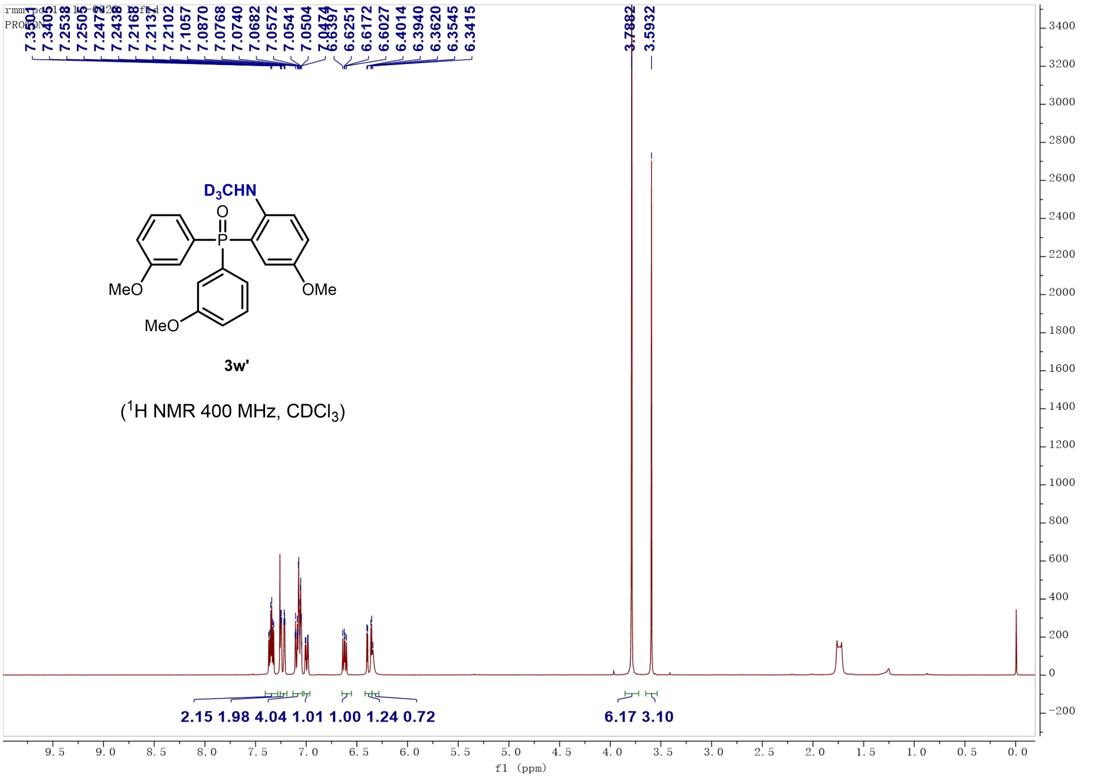
7.3501
7.3405
7.2538
7.2505
7.2472
7.2438
7.2168
7.2137
7.2102
7.1057
7.0870
7.0768
7.0740
7.0682
7.0572
7.0541
7.0504
7.0474
6.6397
6.6251
6.6172
6.6027
6.4014
6.3940
6.3620
6.3545
6.3415
3.7882
3.5932
D3CHN
O
P
MeO
OMe
MeO
3w'
(1H NMR 400 MHz, CDCl3)
2.15 1.98 4.04 1.01 1.00 1.24 0.72
6.17 3.10
f1 (ppm)

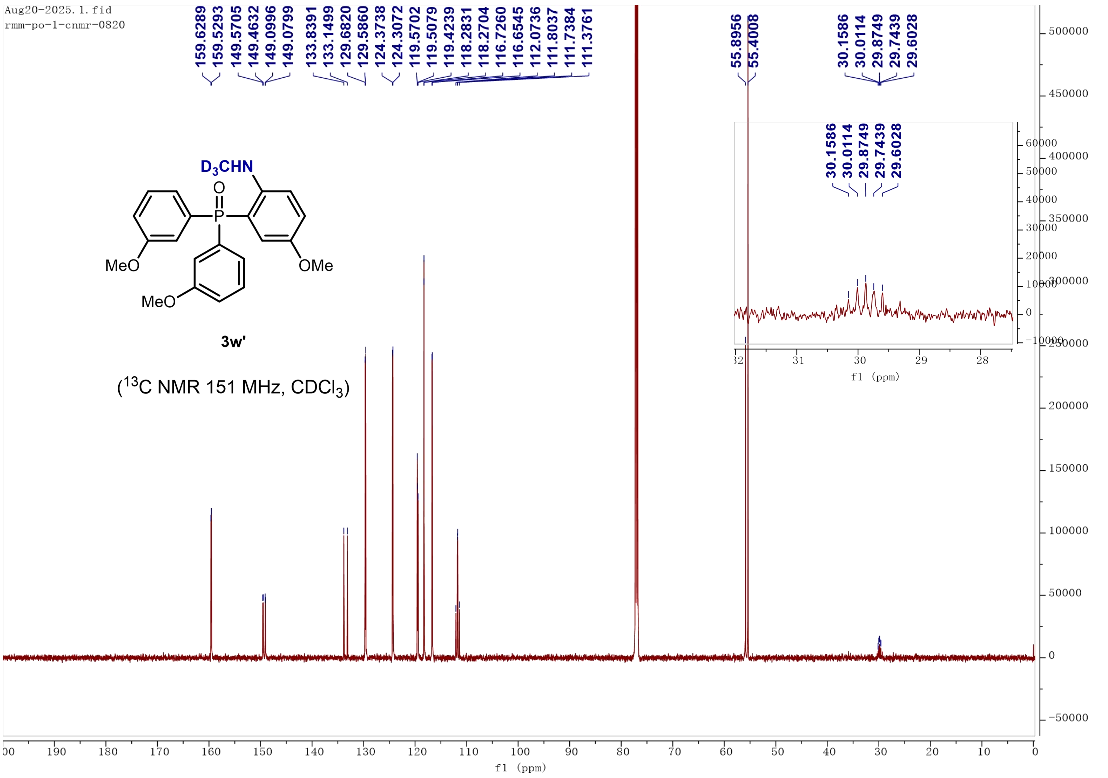
Aug20-2025.1.fid
rmm-po-1-cnmr-0820
159.6289
159.5293
149.5705
149.4632
149.0996
149.0799
133.8391
133.1499
129.6820
129.5860
124.3738
124.3072
119.5702
119.5079
119.4239
118.2831
118.2704
116.7260
116.6545
112.0736
111.8037
111.7384
111.3761
55.8956
55.4008
30.1586
30.0114
29.8749
29.7439
29.6028
D3CHN
O
P
MeO
OMe
MeO
3w'
(13C NMR 151 MHz, CDCl3)
f1 (ppm)

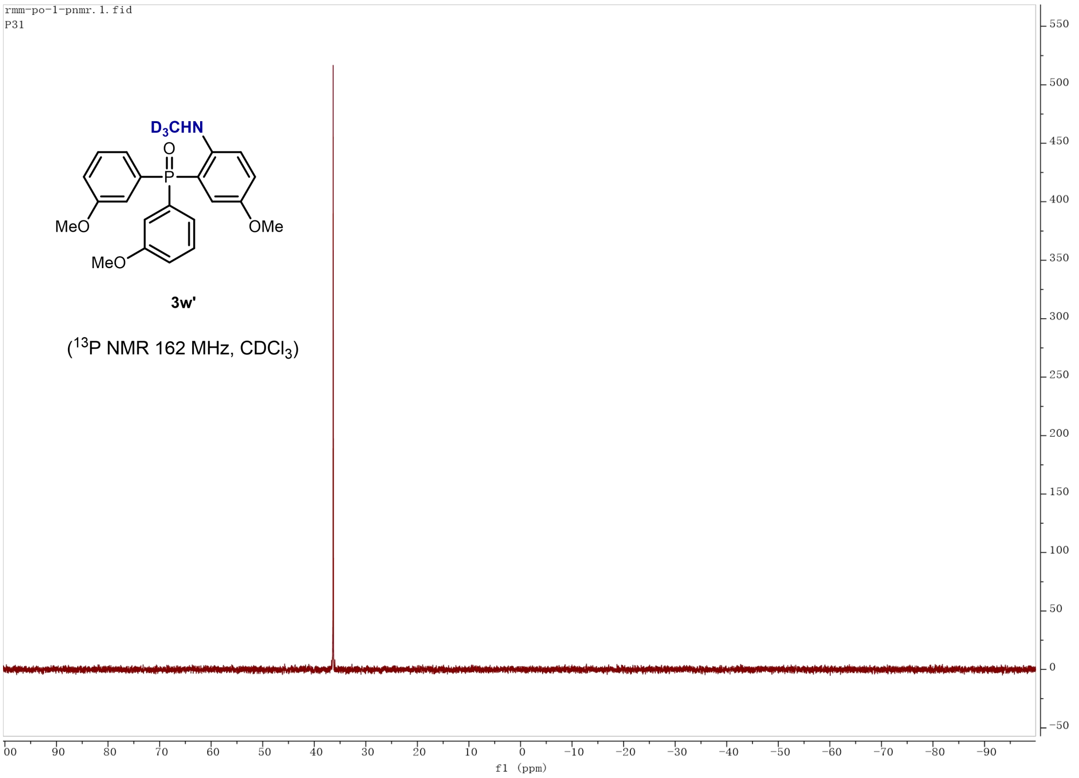
rmm-po-1-pnmr.1.fid
P31
D3CHN
O
P
MeO
OMe
MeO
3w'
(13P NMR 162 MHz, CDCl3)
f1 (ppm)

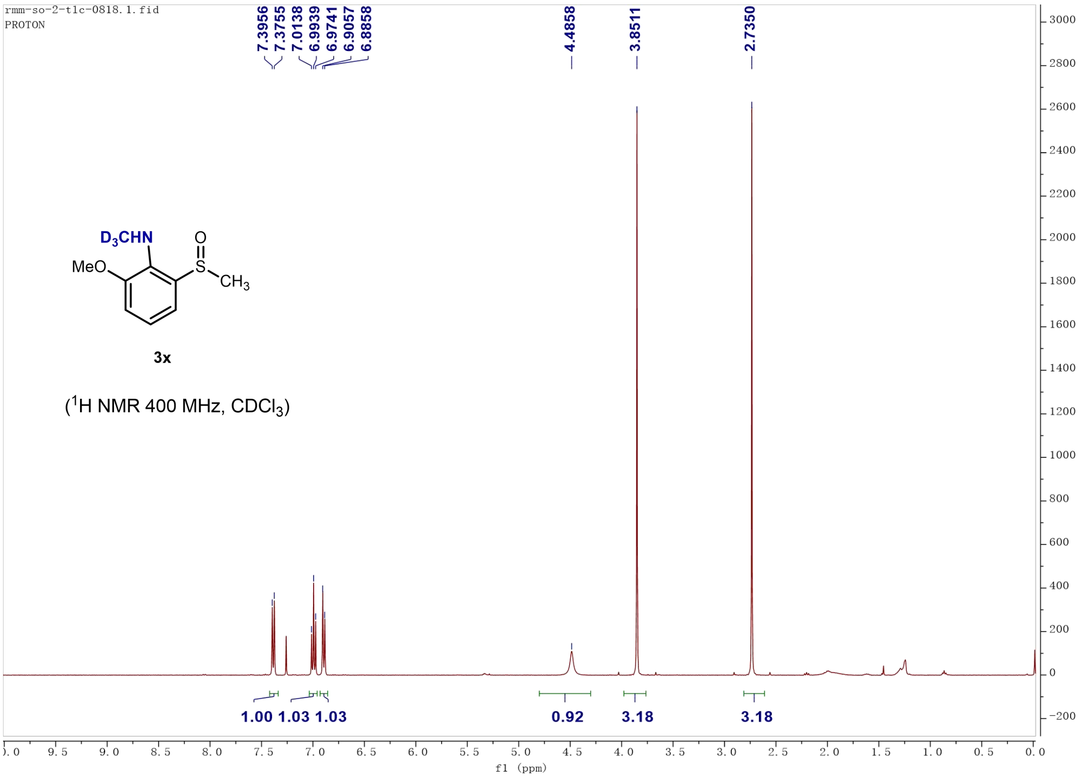
rmm-so-2-t1c-0818.1.fid
PROTON
7.3956
7.3755
7.0138
6.9939
6.9741
6.9057
6.8858
4.4858
3.8511
2.7350
D3CHN
O
MeO
S
CH3
3x
(1H NMR 400 MHz, CDCl3)
1.00 1.03 1.03
0.92
3.18
3.18
f1 (ppm)

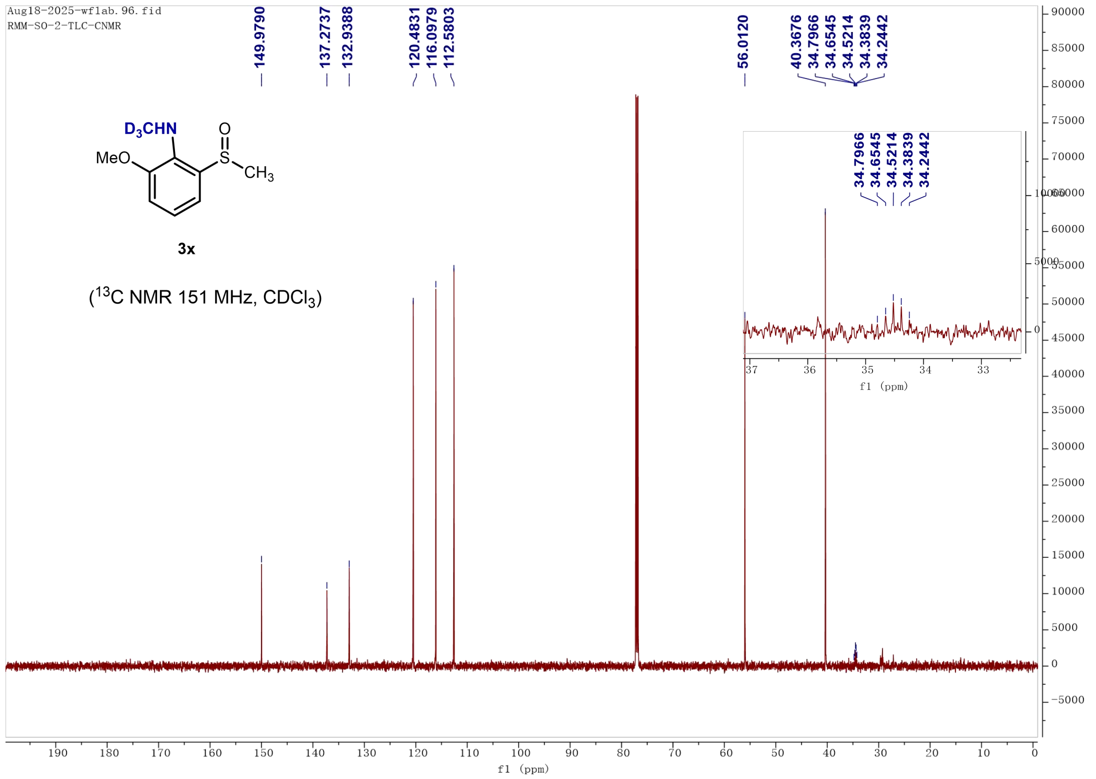
Aug18-2025-wflab.96.fid
RMM-SO-2-TLC-CNMR
149.9790
137.2737
132.9388
120.4831
116.0979
112.5803
56.0120
40.3676
34.7966
34.6545
34.5214
34.3839
34.2442
D3CHN
MeO
O
S
CH3
3x
(13C NMR 151 MHz, CDCl3)
f1 (ppm)

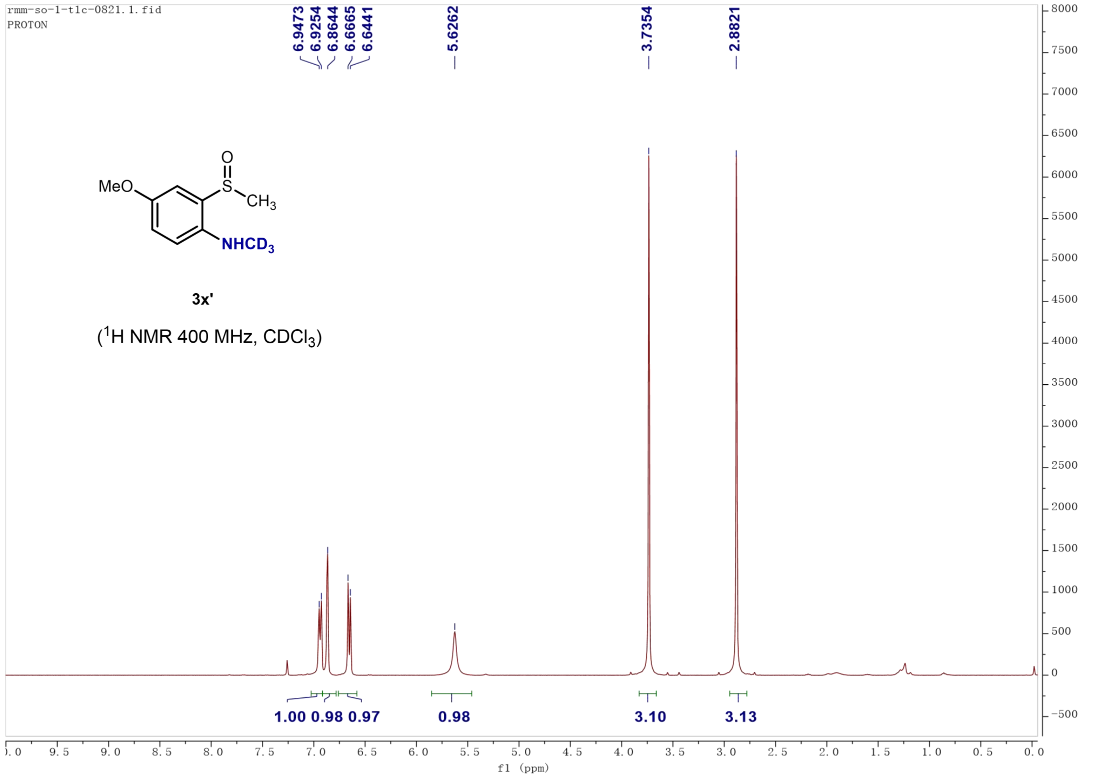
rmm-so-1-tlc-0821.1.fid
PROTON
6.9473
6.9254
6.8644
6.6665
6.6441
5.6262
3.7354
2.8821
MeO
O
S
CH3
NHCD3
3x'
(1H NMR 400 MHz, CDCl3)
1.00 0.98 0.97
0.98
3.10
3.13
f1 (ppm)

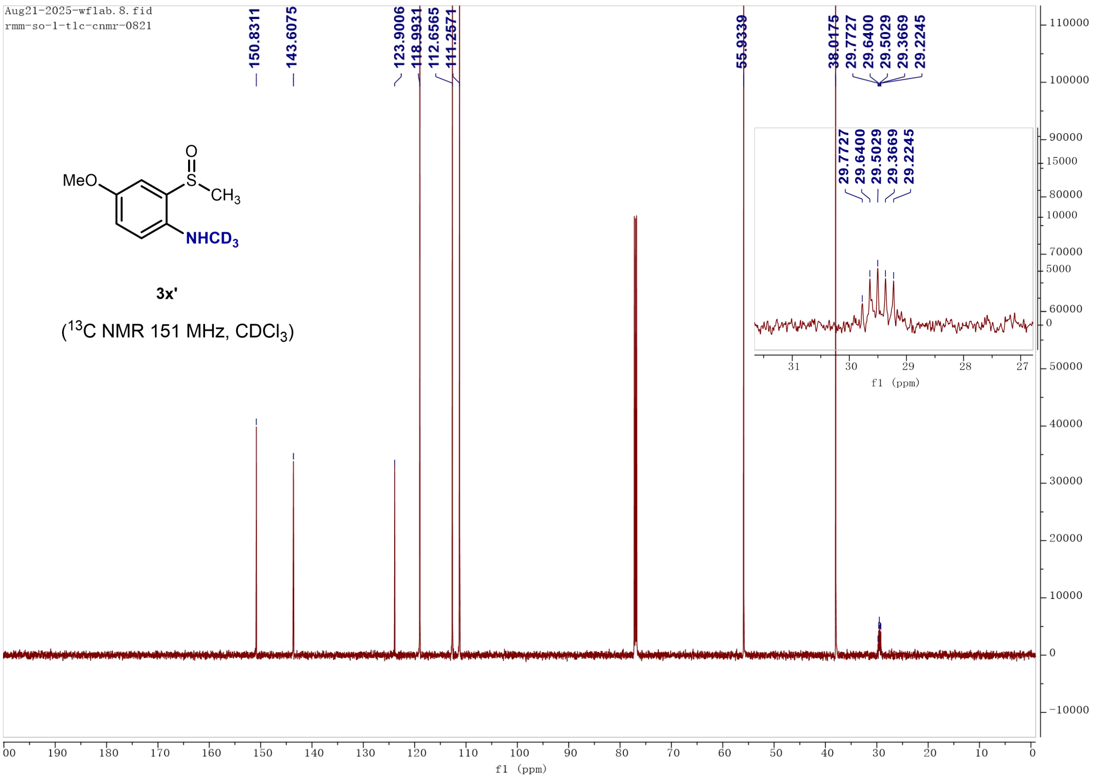

3x'

(^{13}C NMR 151 MHz, CDCl$_3$)

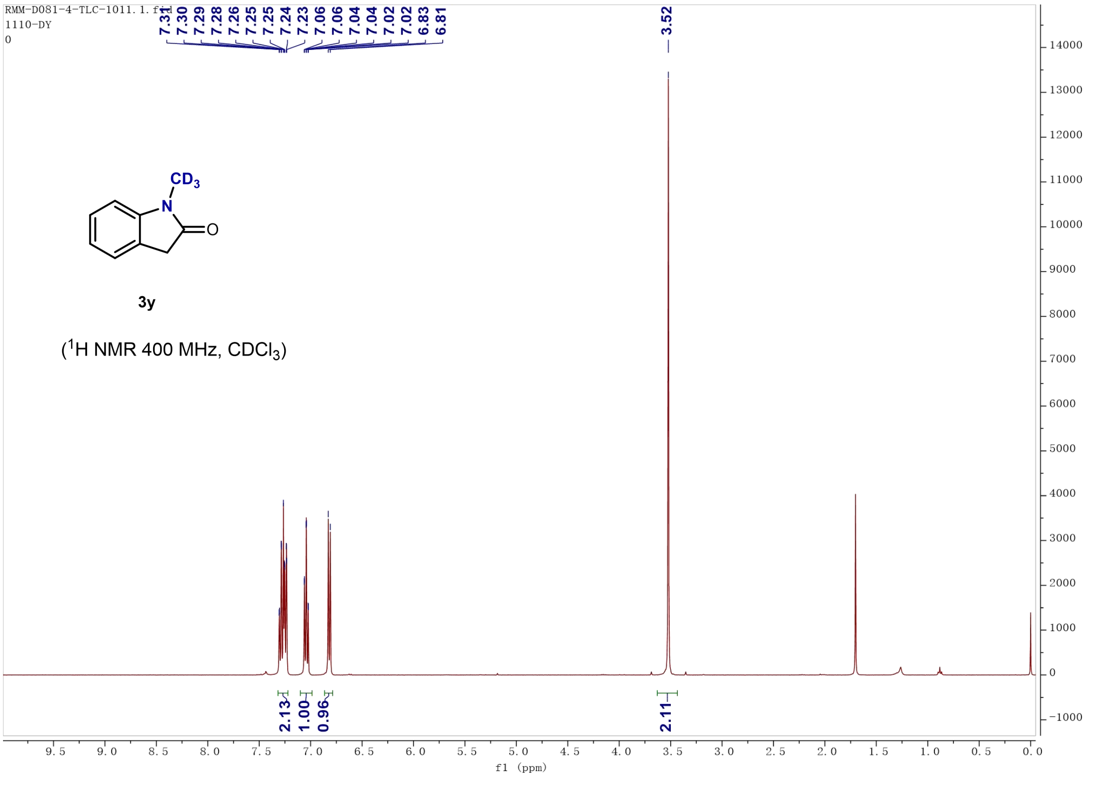

3y

(^{1}H NMR 400 MHz, CDCl$_3$)

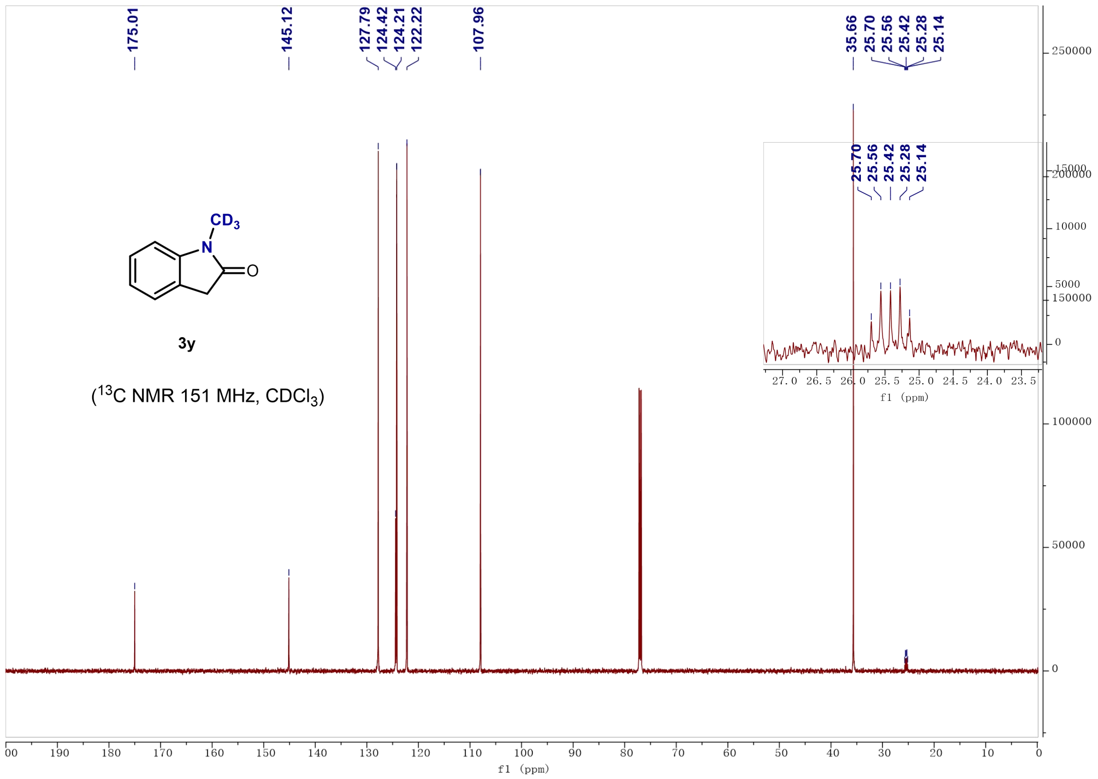
175.01
145.12
127.79
124.42
124.21
122.22
107.96
35.66
25.70
25.56
25.42
25.28
25.14
CD_3
N
O
3y
(^{13}C NMR 151 MHz, $CDCl_3$)
f1 (ppm)

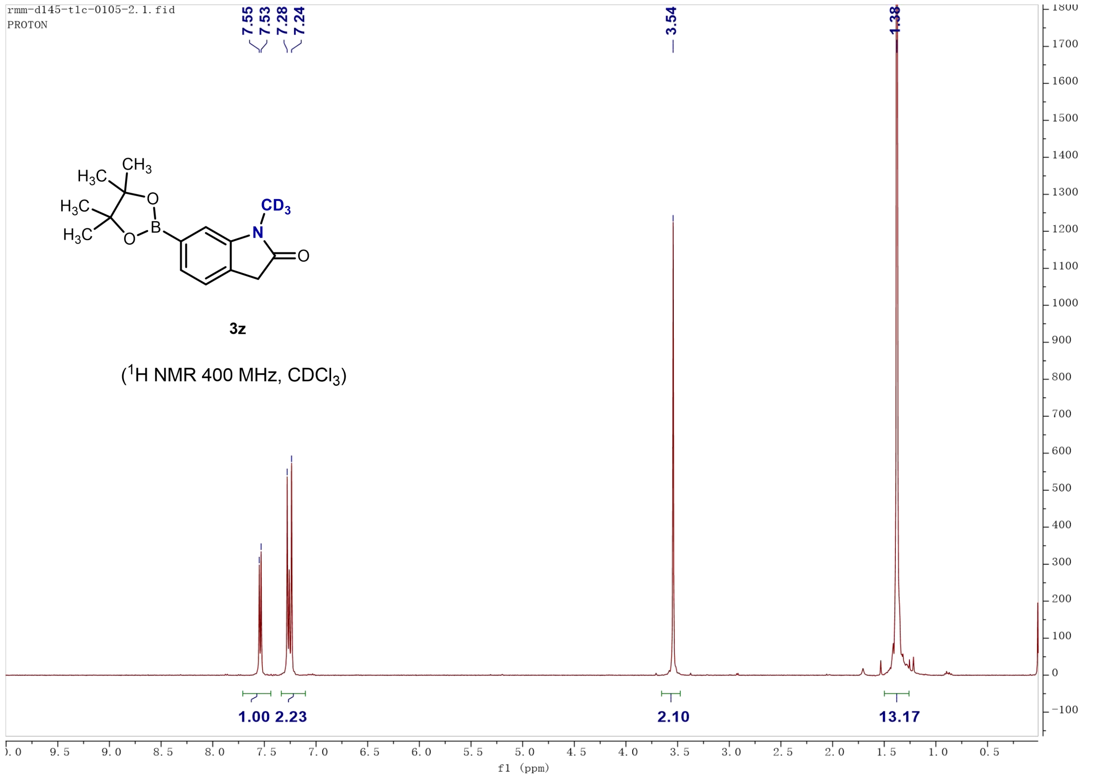
rmm-d145-t1c-0105-2.1.fid
PROTON
7.55
7.53
7.28
7.24
3.54
1.38
H_3C
CH_3
H_3C
H_3C
O
B
O
N
CD_3
O
3z
(^{1}H NMR 400 MHz, $CDCl_3$)
1.00
2.23
2.10
13.17
f1 (ppm)

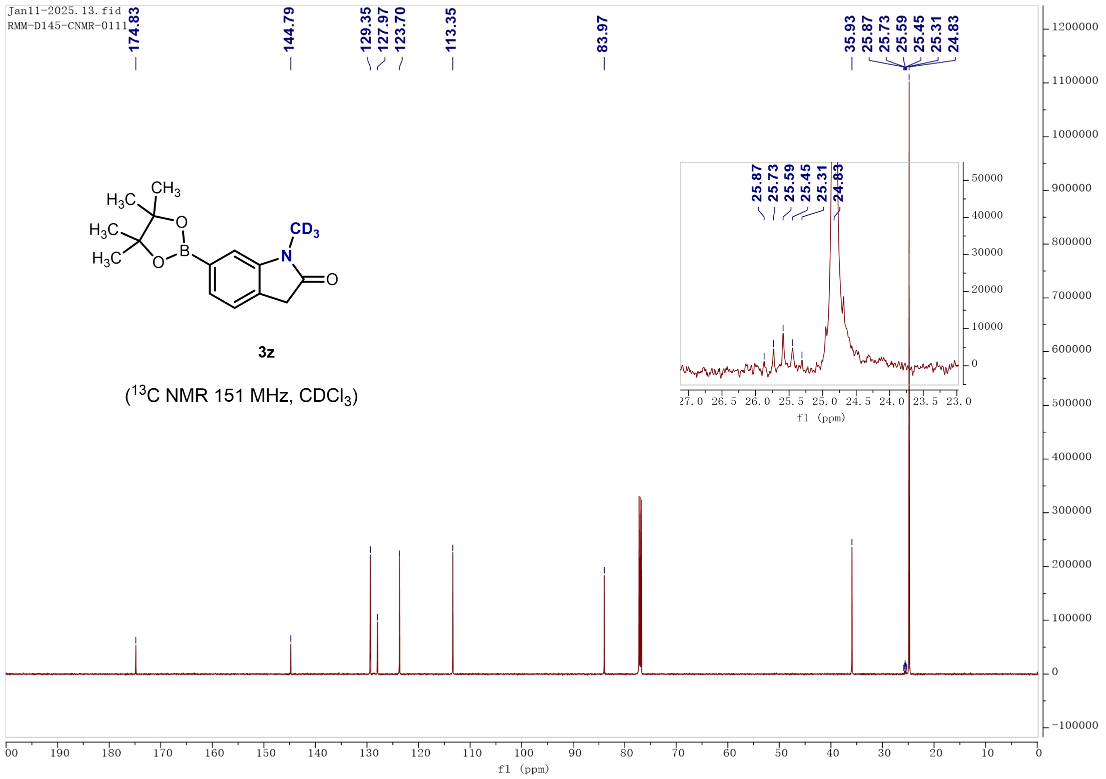
Jan11-2025.13.fid
RMM-D145-CNMR-0111
174.83
144.79
129.35
127.97
123.70
113.35
83.97
35.93
25.87
25.73
25.59
25.45
25.31
24.83
3z
(^{13}C NMR 151 MHz, CDCl$_3$)
f1 (ppm)

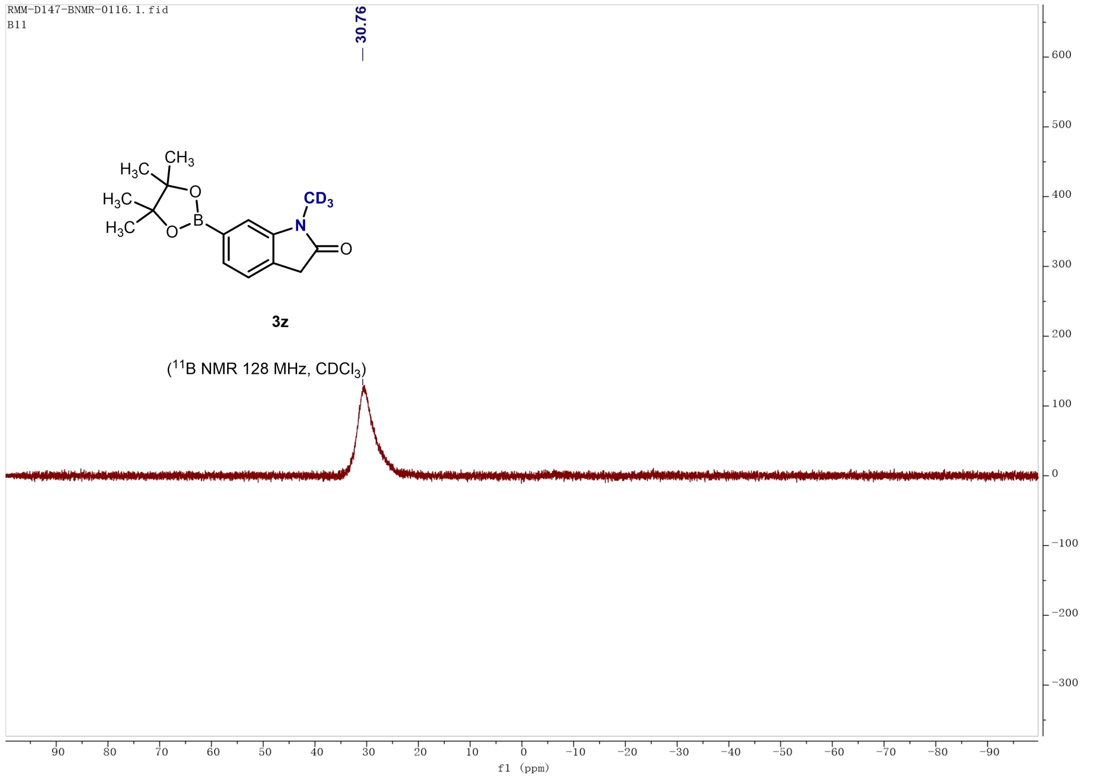
RMM-D147-BNMR-0116.1.fid
B11
30.76
3z
(^{11}B NMR 128 MHz, CDCl$_3$)
f1 (ppm)

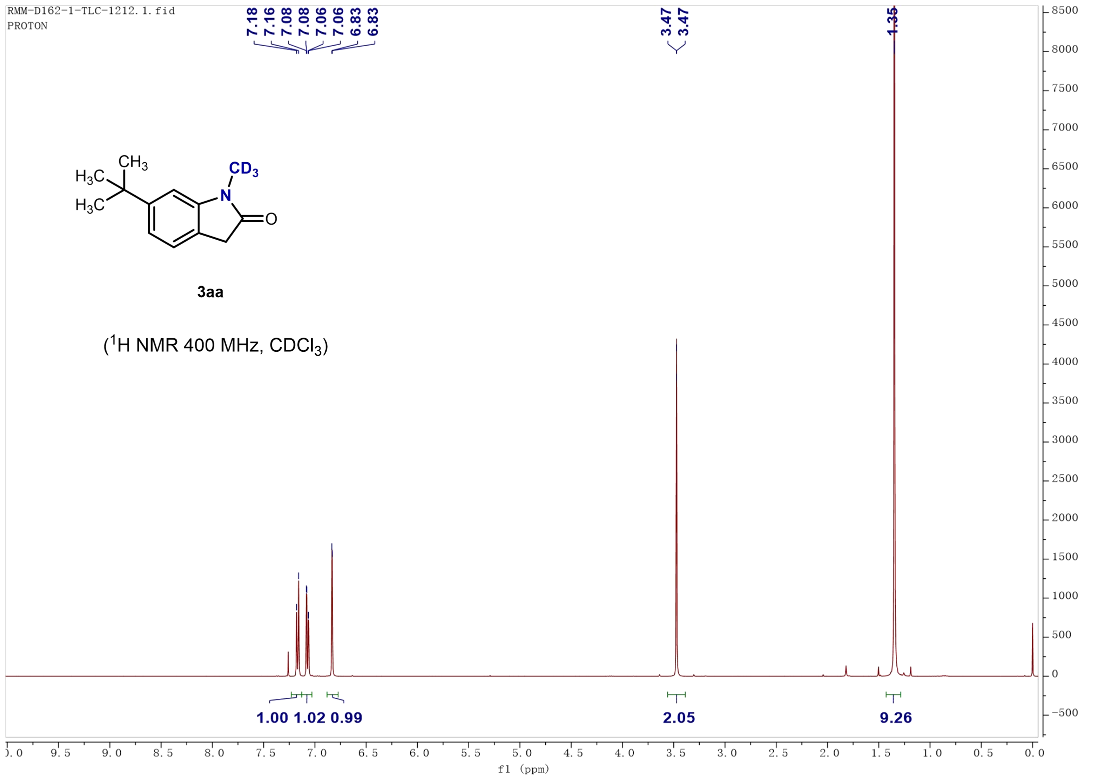
RMM-D162-1-TLC-1212.1.fid
PROTON
7.18 7.16 7.08 7.08 7.06 7.06 6.83 6.83
3.47 3.47
1.35
3aa
(1H NMR 400 MHz, CDCl3)
1.00 1.02 0.99
2.05
9.26
f1 (ppm)

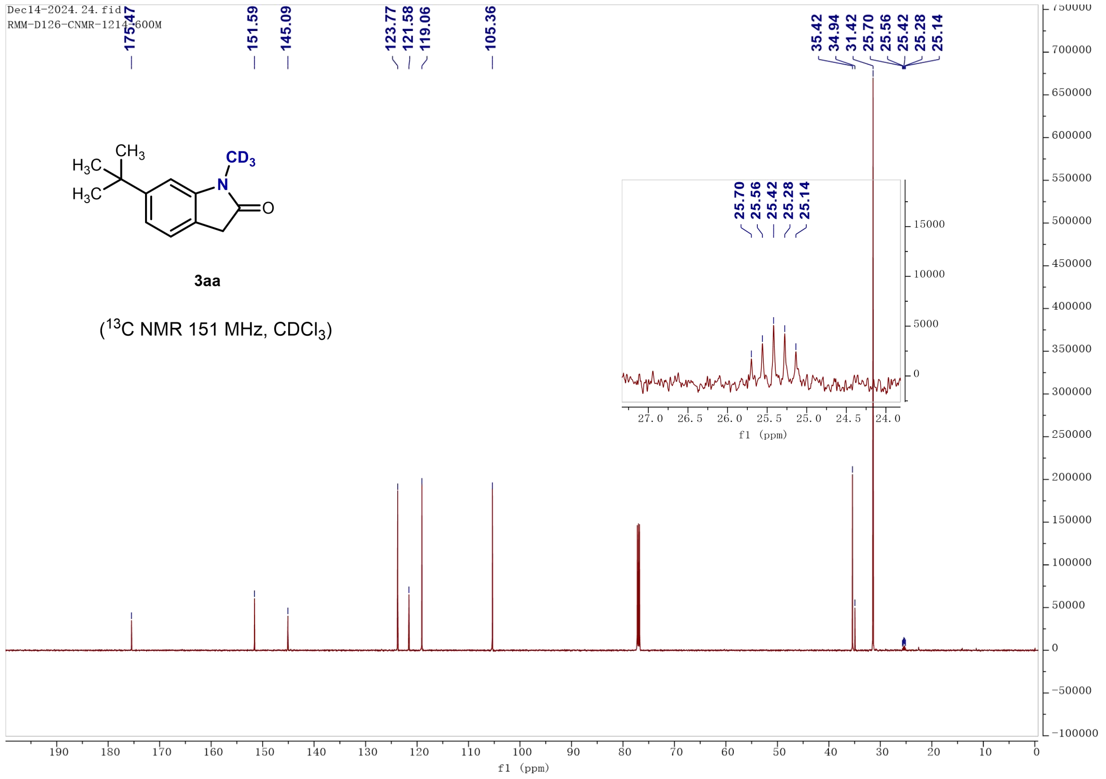
Dec14-2024.24.fid
RMM-D126-CNMR-1214-600M
175.47
151.59
145.09
123.77 121.58 119.06
105.36
35.42 34.94 31.42 25.70 25.56 25.42 25.28 25.14
3aa
(13C NMR 151 MHz, CDCl3)
25.70 25.56 25.42 25.28 25.14
f1 (ppm)

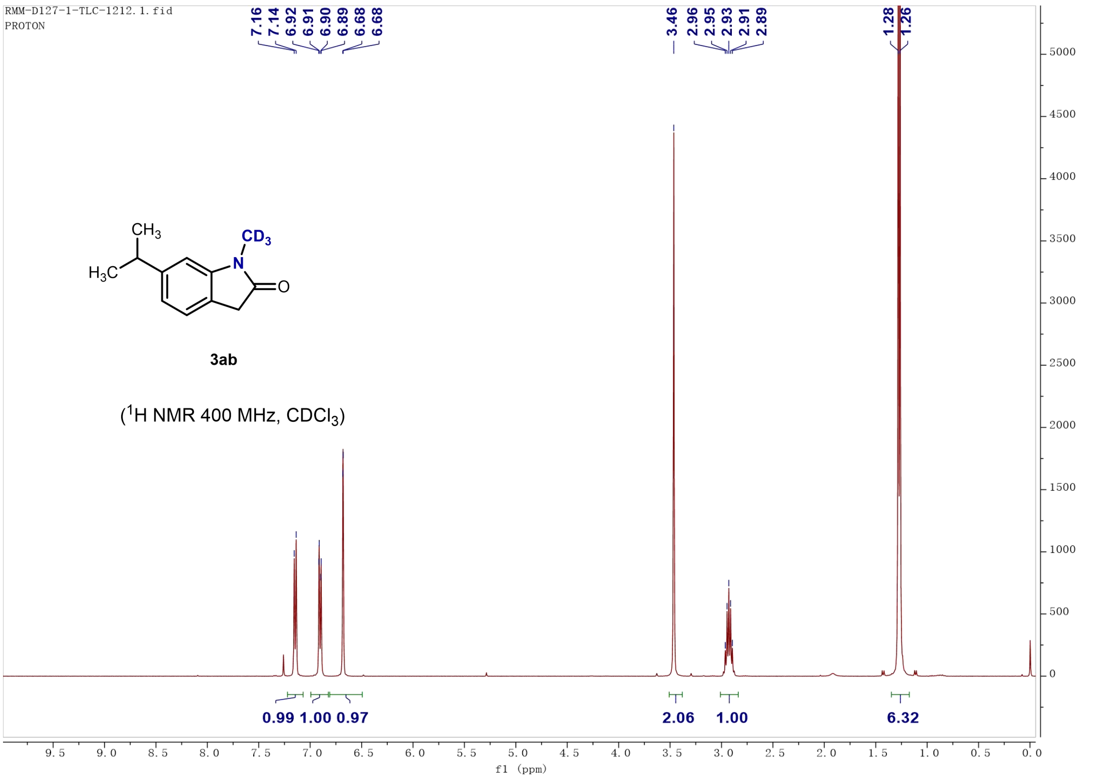
RMM-D127-1-TLC-1212.1.fid
PROTON
7.16
7.14
6.92
6.91
6.90
6.89
6.68
6.68
3.46
2.96
2.95
2.93
2.91
2.89
1.28
1.26
CH3
CD3
N
H3C
O
3ab
(1H NMR 400 MHz, CDCl3)
0.99 1.00 0.97
2.06
1.00
6.32
f1 (ppm)

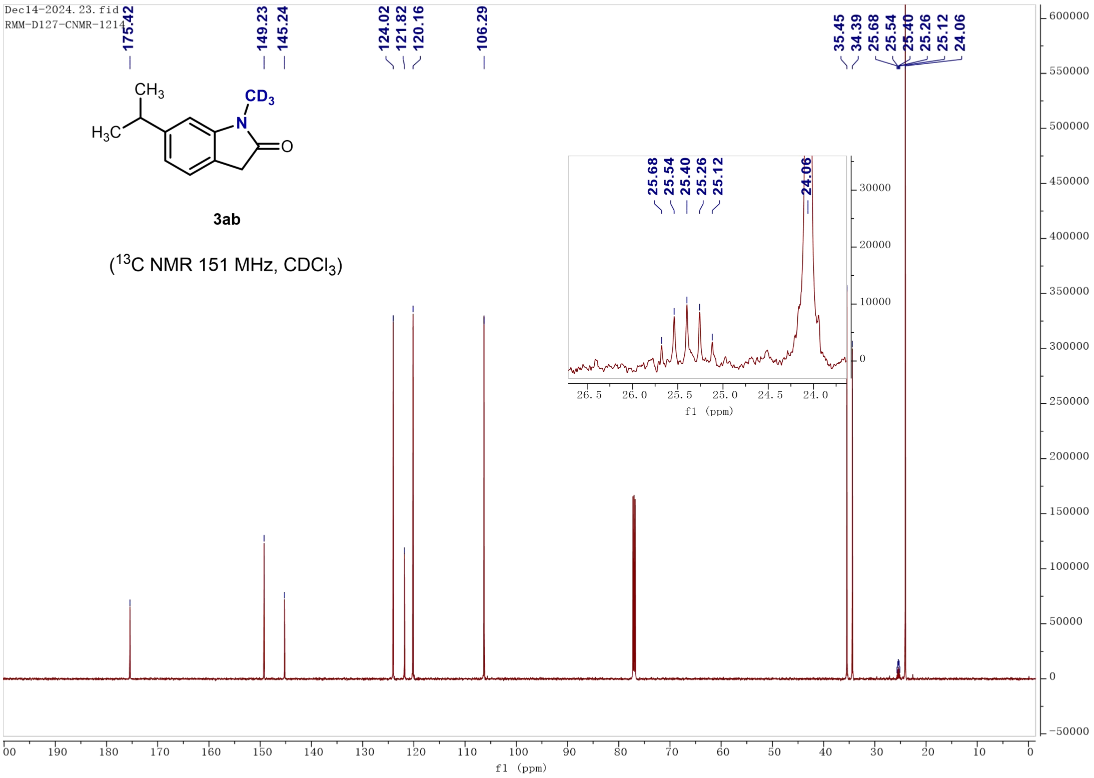
Dec14-2024.23.fid
RMM-D127-CNMR-1214
175.42
149.23
145.24
124.02
121.82
120.16
106.29
35.45
34.39
25.68
25.54
25.40
25.26
25.12
24.06
CH3
CD3
N
H3C
O
3ab
(13C NMR 151 MHz, CDCl3)
f1 (ppm)

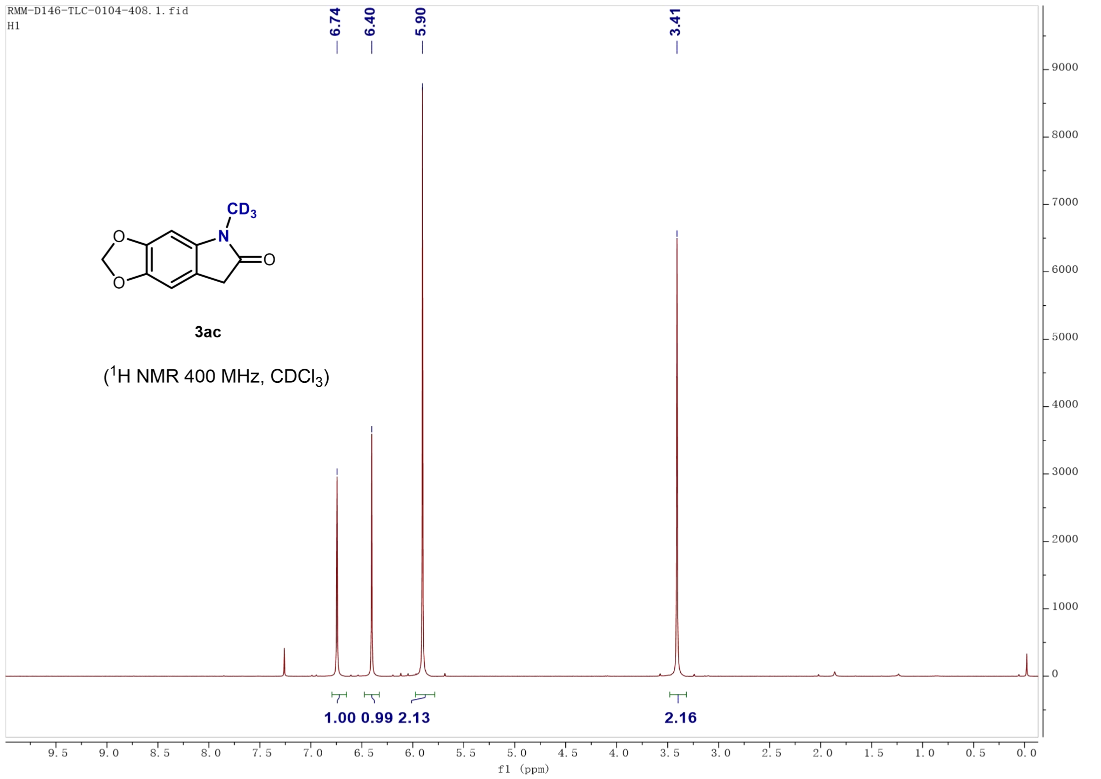
RMM-D146-TLC-0104-408.1.fid
H1
6.74
6.40
5.90
3.41
CD3
N
O
O
O
3ac
(1H NMR 400 MHz, CDCl3)
1.00 0.99 2.13
2.16
f1 (ppm)

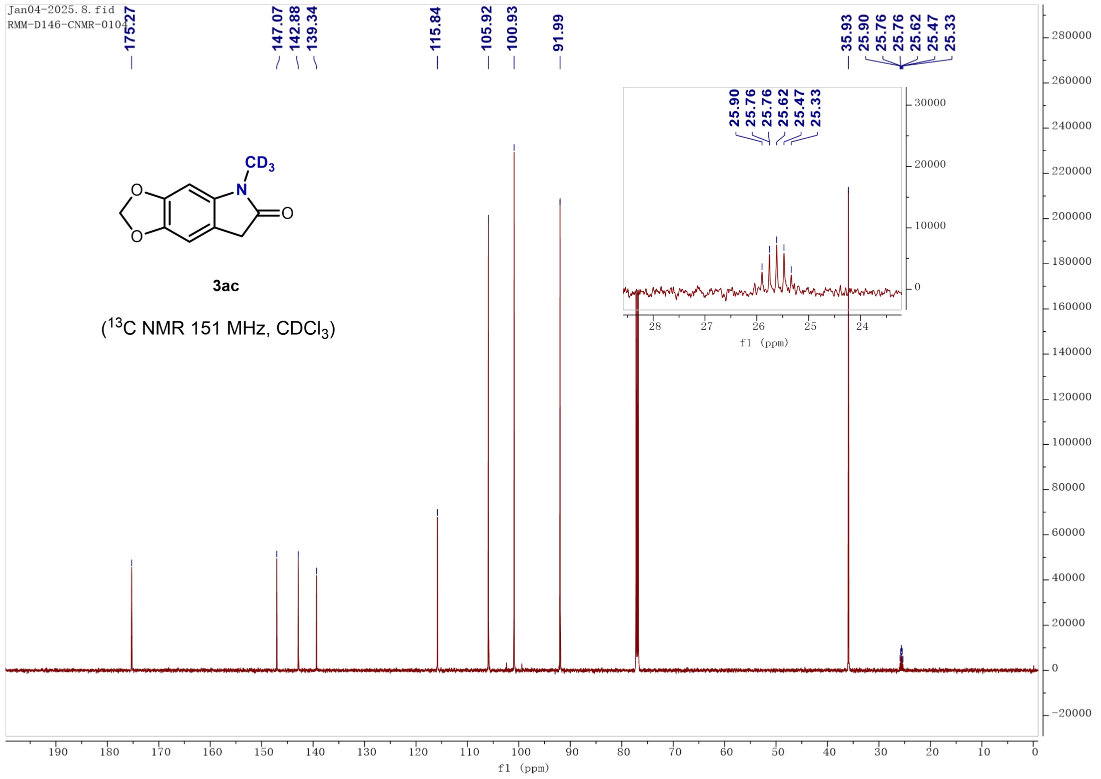
Jan04-2025.8.fid
RMM-D146-CNMR-0104
175.27
147.07
142.88
139.34
115.84
105.92
100.93
91.99
35.93
25.90
25.76
25.76
25.62
25.47
25.33
CD3
N
O
O
O
3ac
(13C NMR 151 MHz, CDCl3)
f1 (ppm)

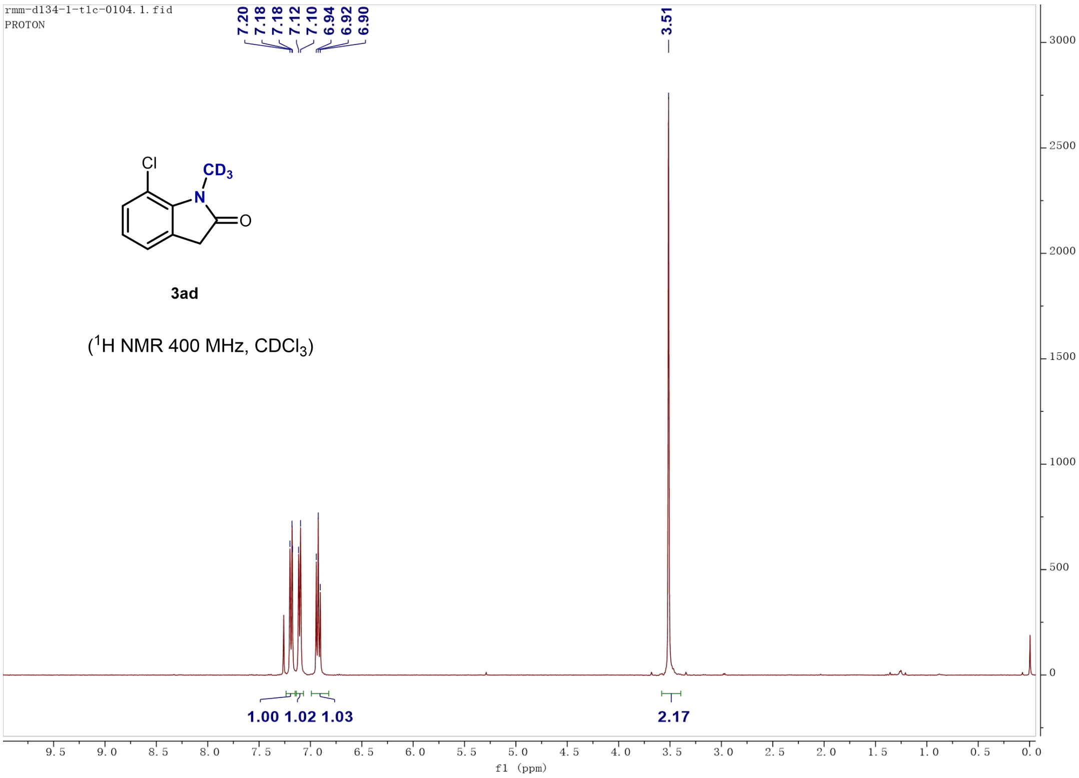

rmm-d134-1-tlc-0104.1.fid
PROTON
7.20
7.18
7.18
7.12
7.10
6.94
6.92
6.90
3.51
Cl
CD3
N
O
3ad
(1H NMR 400 MHz, CDCl3)
1.00
1.02
1.03
2.17
f1 (ppm)

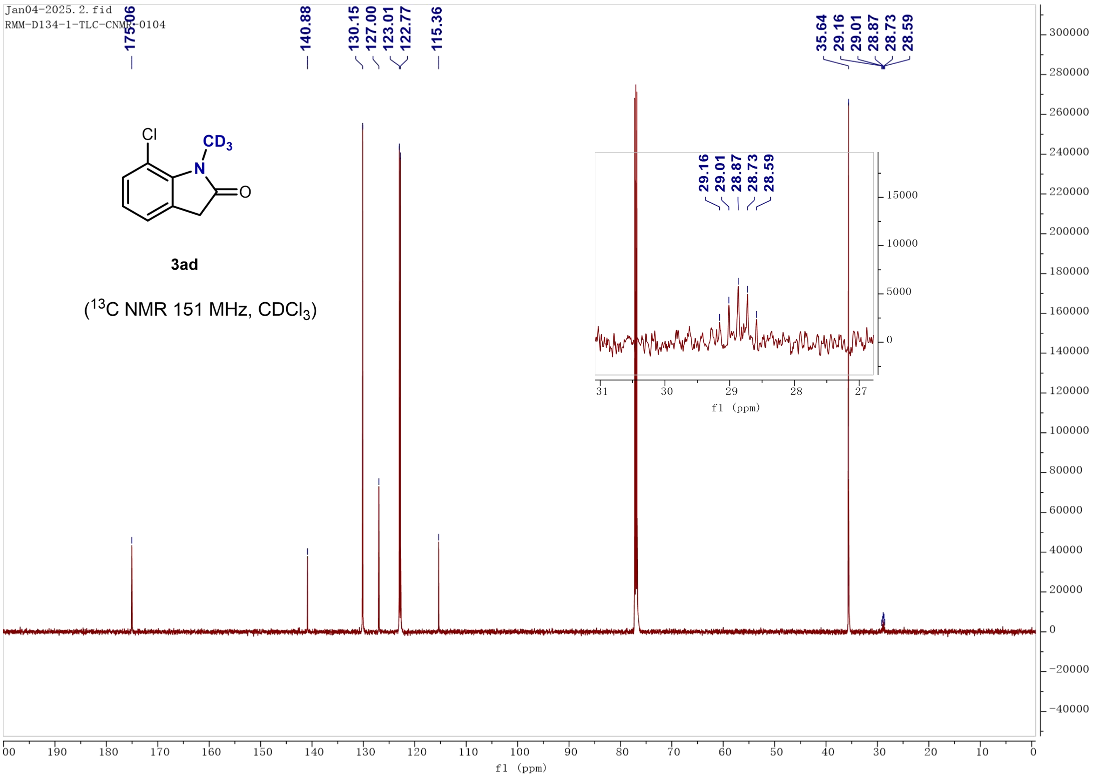

Jan04-2025.2.fid
RMM-D134-1-TLC-CNMR-0104
175.06
140.88
130.15
127.00
123.01
122.77
115.36
35.64
29.16
29.01
28.87
28.73
28.59
Cl
CD3
N
O
3ad
(13C NMR 151 MHz, CDCl3)
f1 (ppm)

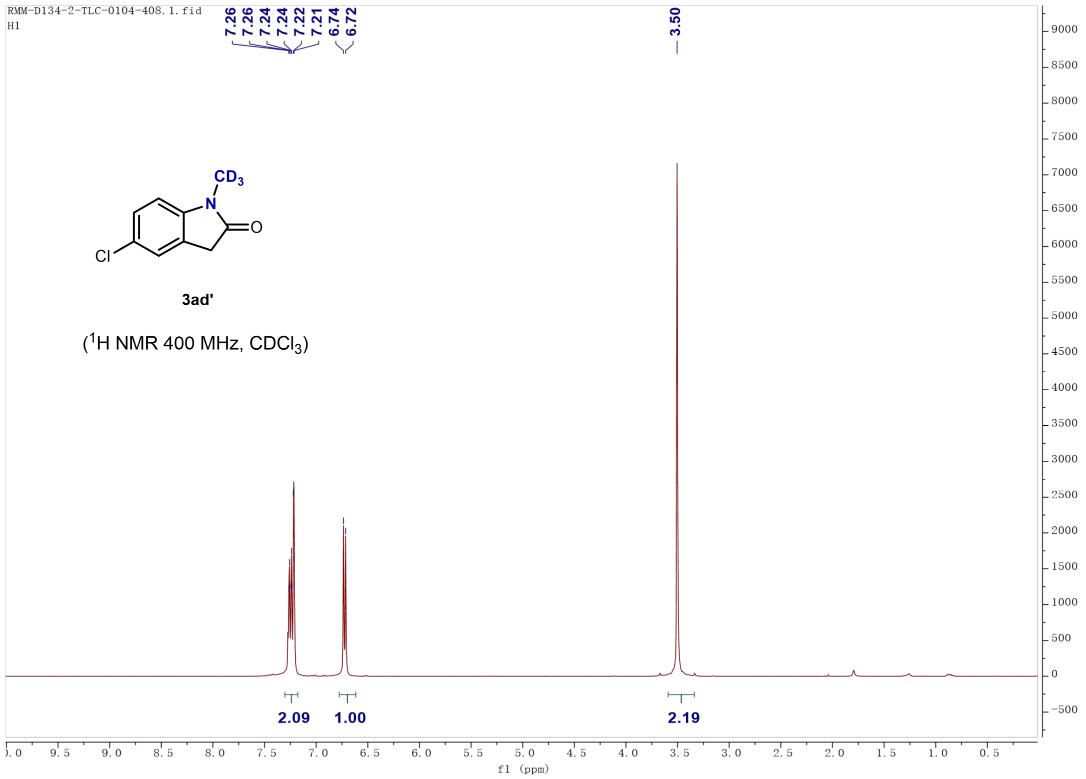
RMM-D134-2-TLC-0104-408.1.fid
H1
7.26
7.26
7.24
7.24
7.22
7.21
6.74
6.72
3.50
CD3
N
O
Cl
3ad'
(1H NMR 400 MHz, CDCl3)
2.09
1.00
2.19
f1 (ppm)

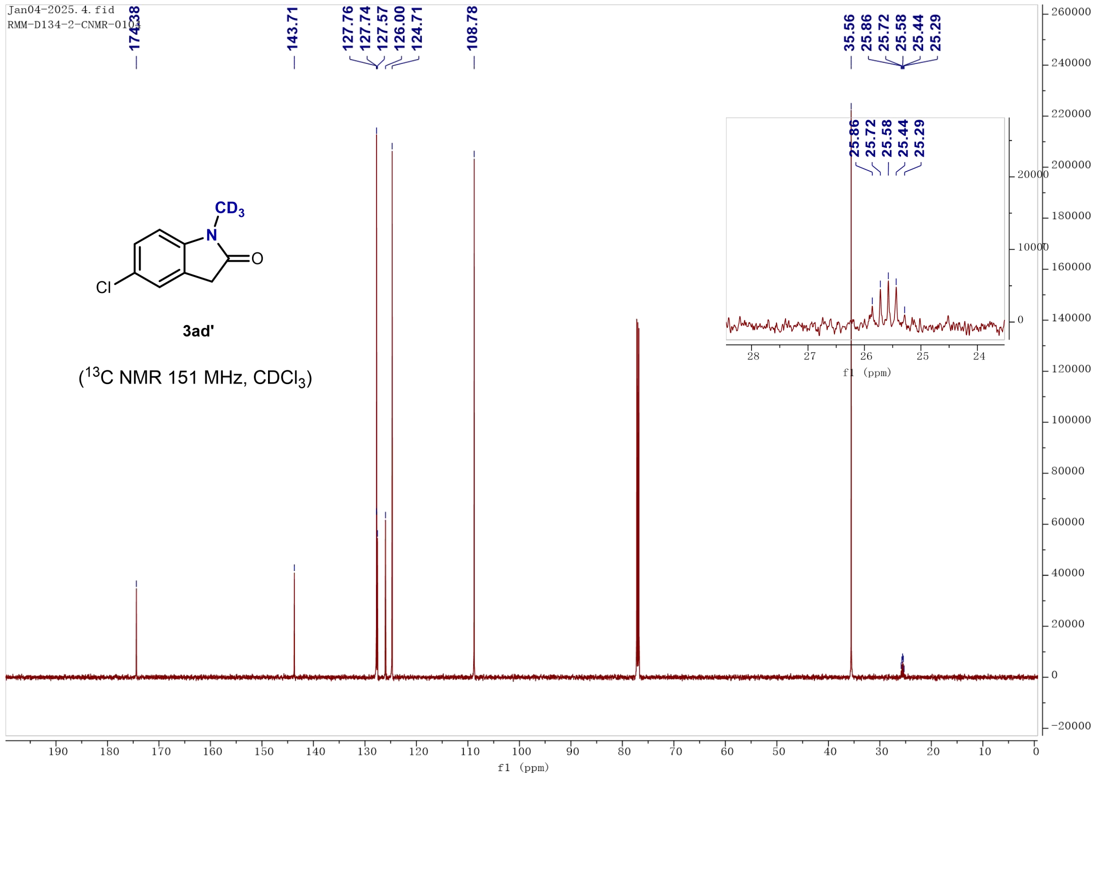
Jan04-2025.4.fid
RMM-D134-2-CNMR-0104
174.38
143.71
127.76
127.74
127.57
126.00
124.71
108.78
35.56
25.86
25.72
25.58
25.44
25.29
CD3
N
O
Cl
3ad'
(13C NMR 151 MHz, CDCl3)
f1 (ppm)

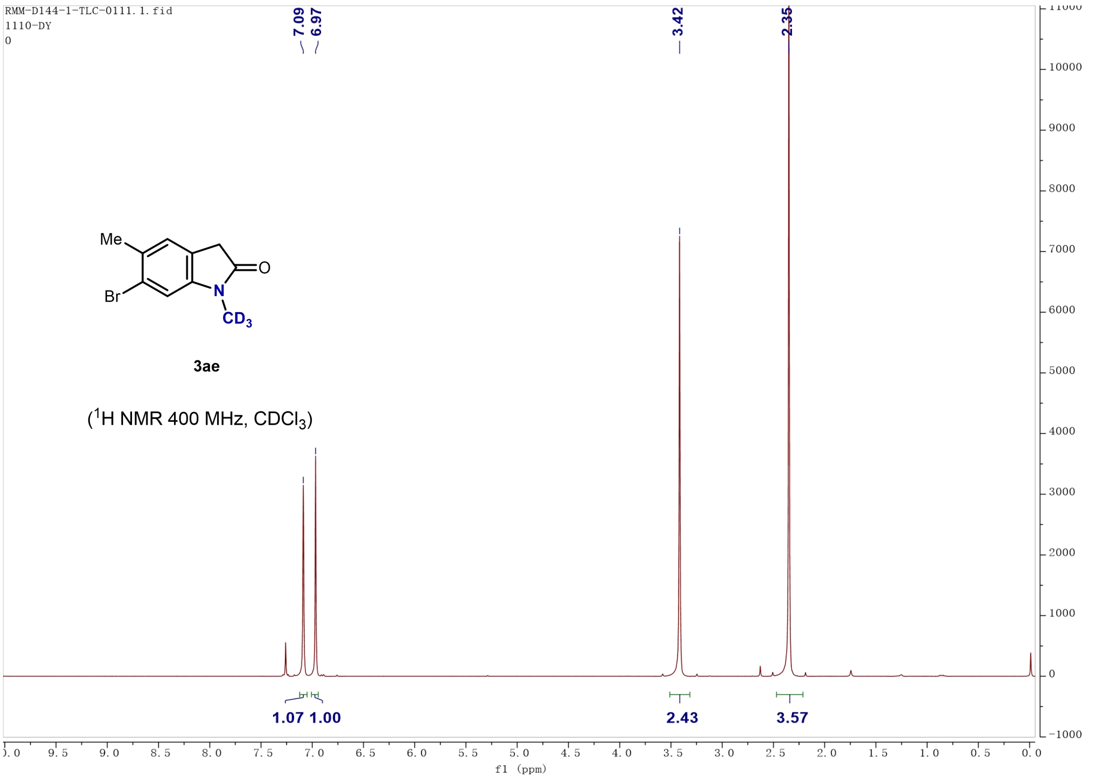
RMM-D144-1-TLC-0111.1.fid
1110-DY
0
7.09
6.97
3.42
2.35
Me
Br
N
O
CD3
3ae
(1H NMR 400 MHz, CDCl3)
1.07 1.00
2.43
3.57
f1 (ppm)

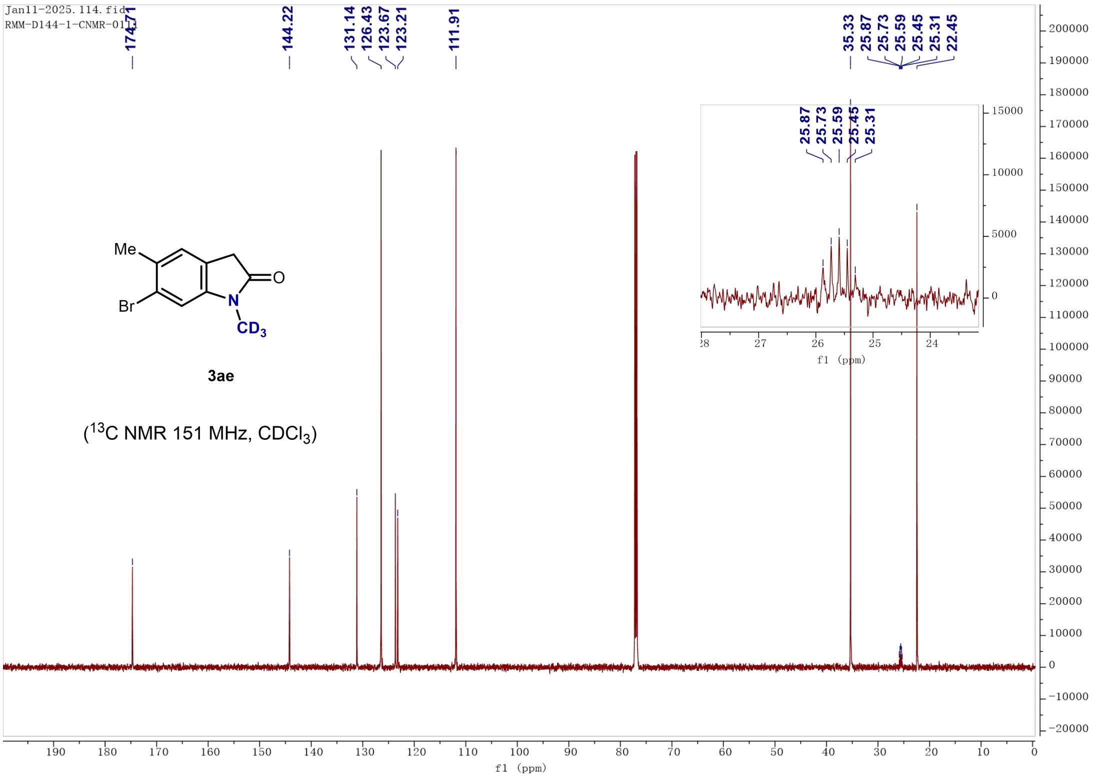
Jan11-2025.114.fid
174.71
144.22
131.14
126.43
123.67
123.21
111.91
35.33
25.87
25.73
25.59
25.45
25.31
22.45
Me
Br
N
O
CD3
3ae
(13C NMR 151 MHz, CDCl3)
f1 (ppm)

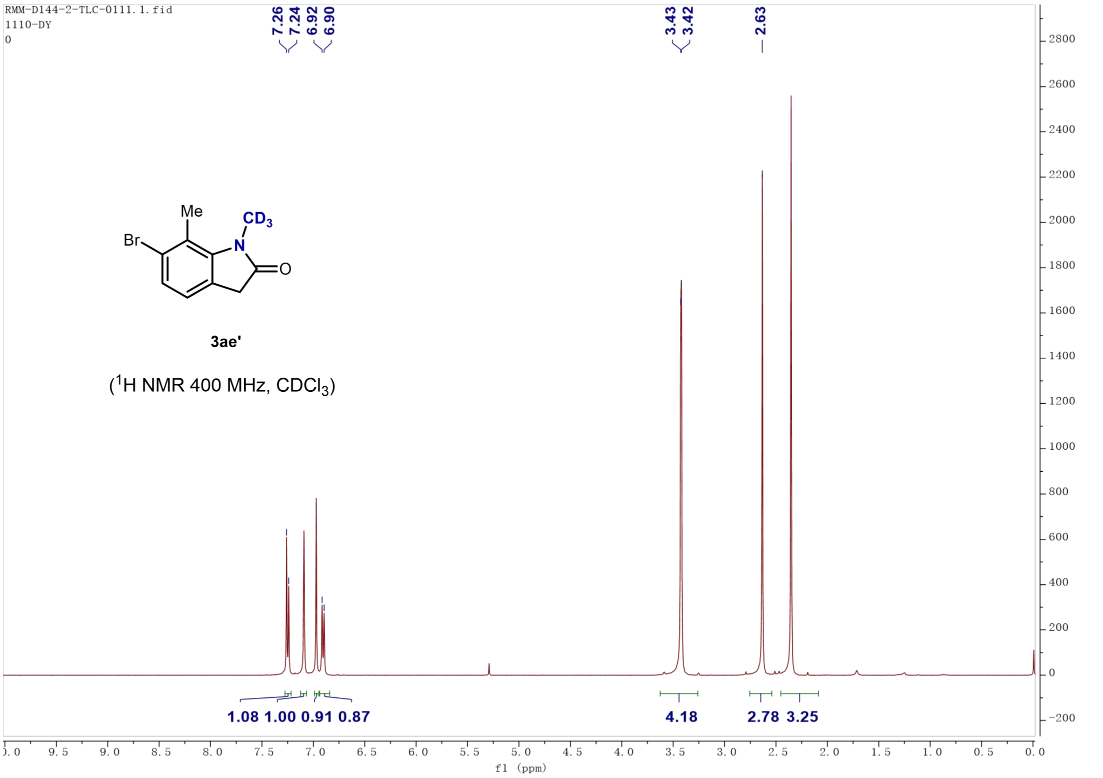
RMM-D144-2-TLC-0111.1.fid
1110-DY
7.26
7.24
6.92
6.90
3.43
3.42
2.63
Me
CD3
Br
N
O
3ae'
(1H NMR 400 MHz, CDCl3)
1.08 1.00 0.91 0.87
4.18
2.78 3.25
f1 (ppm)

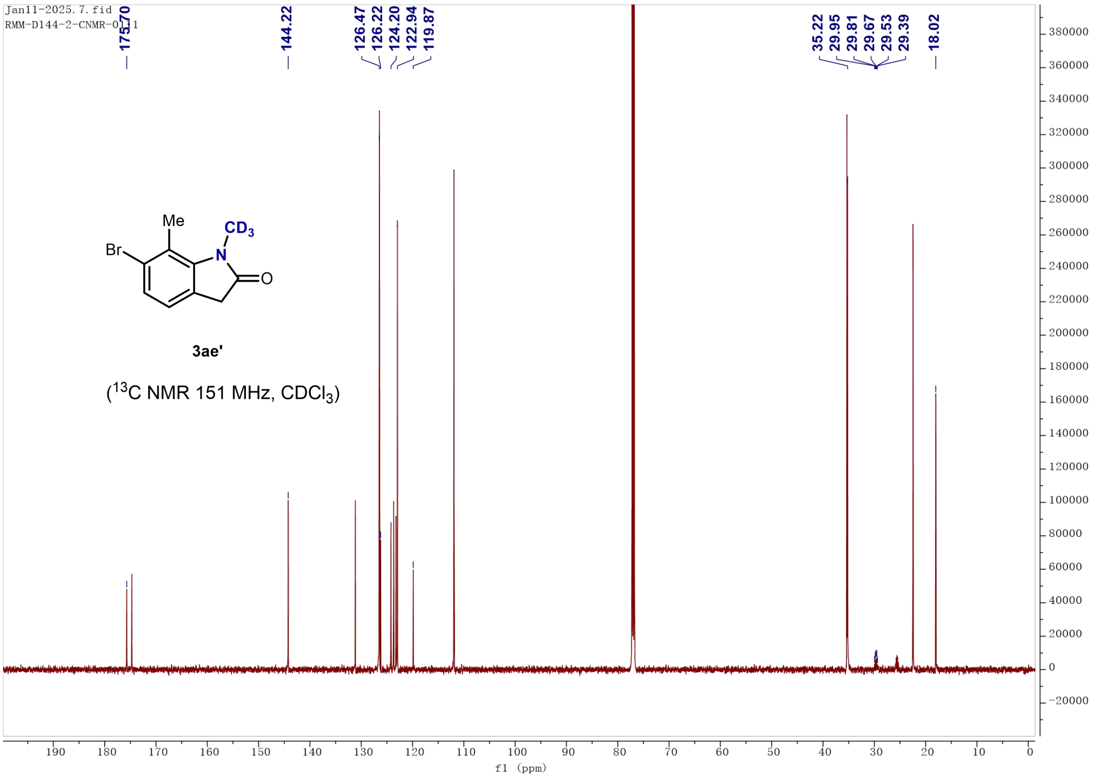
Jan11-2025.7.fid
RMM-D144-2-CNMR-0111
175.70
144.22
126.47
126.22
124.20
122.94
119.87
35.22
29.95
29.81
29.67
29.53
29.39
18.02
Me
CD3
Br
N
O
3ae'
(13C NMR 151 MHz, CDCl3)
f1 (ppm)

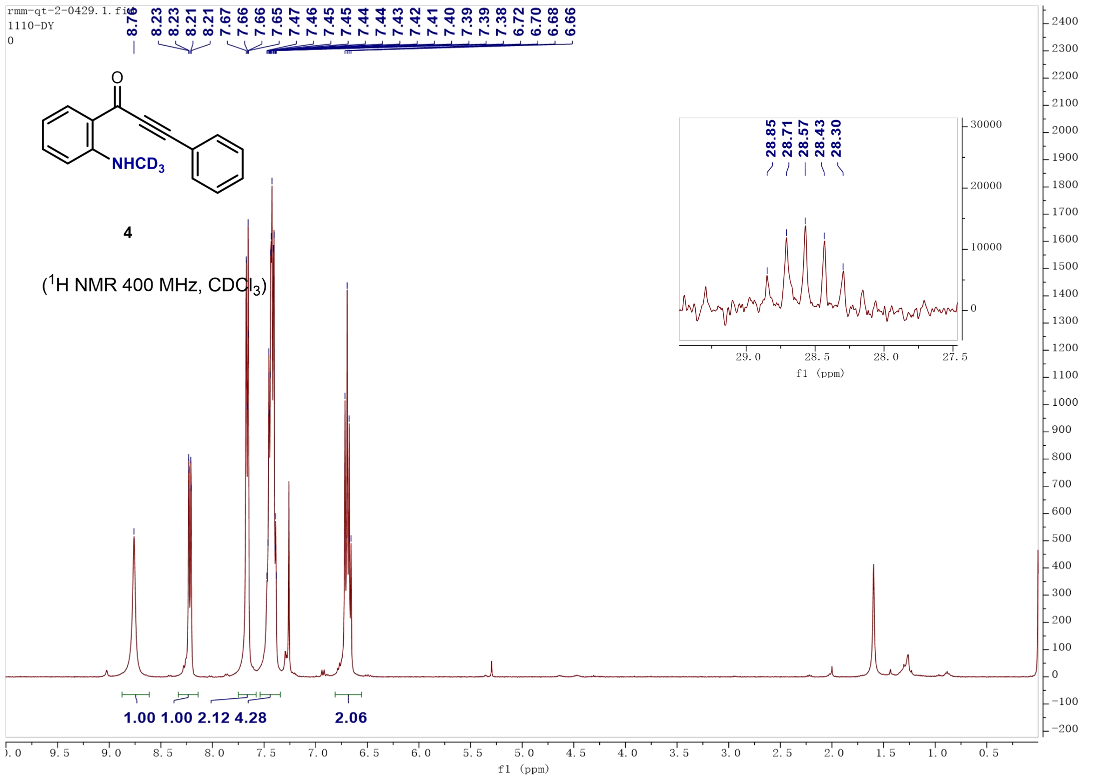
rmm-qt-2-0429.1.fid
1110-DY
8.76
8.23
8.23
8.21
8.21
7.67
7.66
7.66
7.65
7.47
7.46
7.45
7.45
7.44
7.44
7.43
7.42
7.41
7.40
7.39
7.38
6.72
6.70
6.68
6.66
NHCD3
4
(1H NMR 400 MHz, CDCl3)
28.85
28.71
28.57
28.43
28.30
1.00
1.00
2.12
4.28
2.06
f1 (ppm)

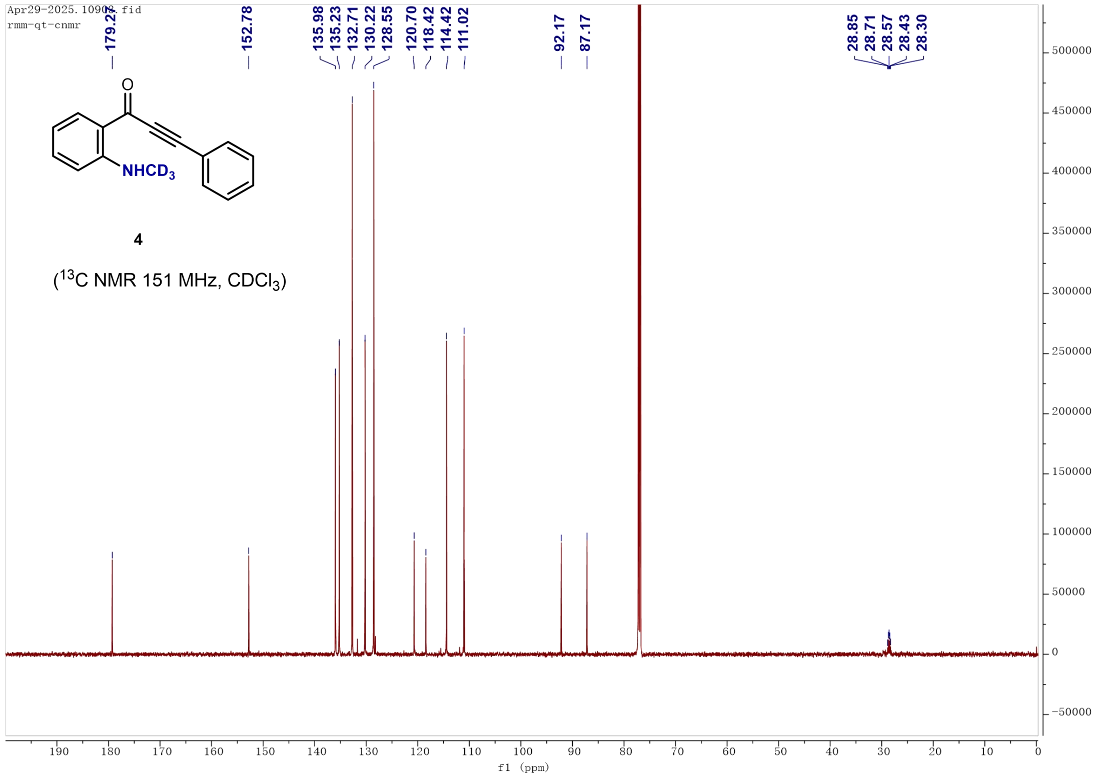
Apr29-2025.10908.fid
rmm-qt-cnmr
179.23
152.78
135.98
135.23
132.71
130.22
128.55
120.70
118.42
114.42
111.02
92.17
87.17
28.85
28.71
28.57
28.43
28.30
NHCD3
4
(13C NMR 151 MHz, CDCl3)
f1 (ppm)

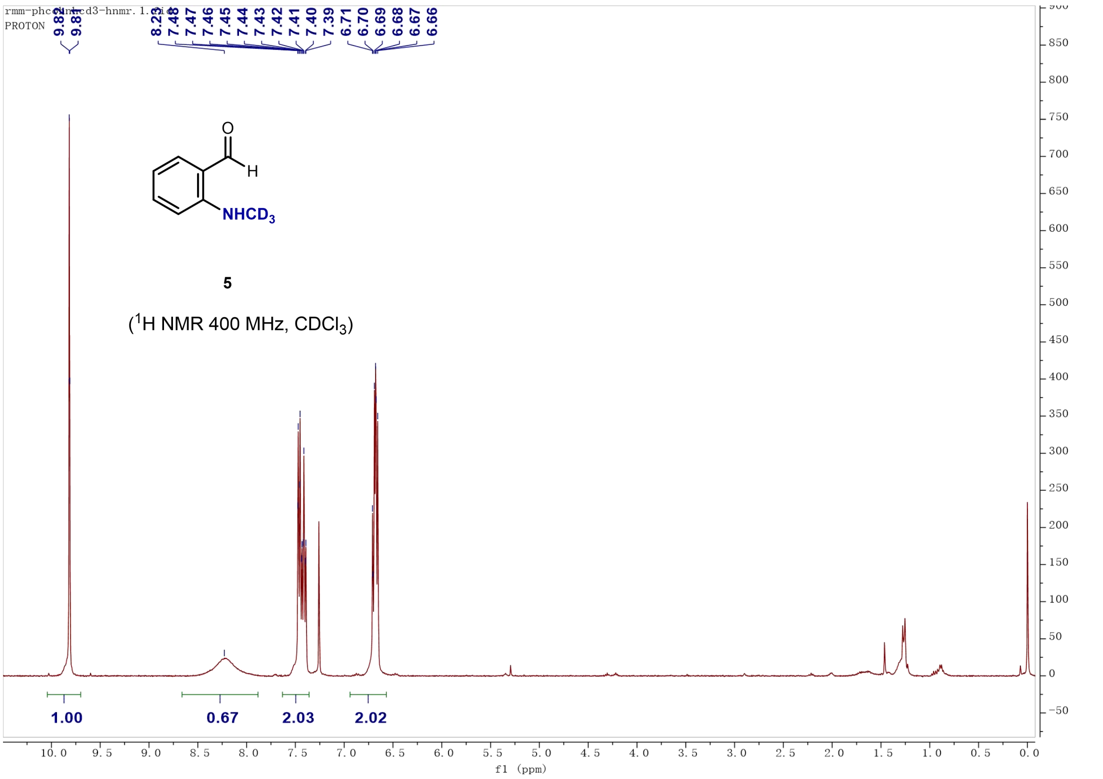
rmm-phcohnhcd3-hnmr.1.fid
PROTON
9.82
9.81
8.23
7.48
7.47
7.46
7.45
7.44
7.43
7.42
7.41
7.40
7.39
6.71
6.70
6.69
6.68
6.67
6.66
NHCD3
5
(1H NMR 400 MHz, CDCl3)
1.00
0.67
2.03
2.02
f1 (ppm)

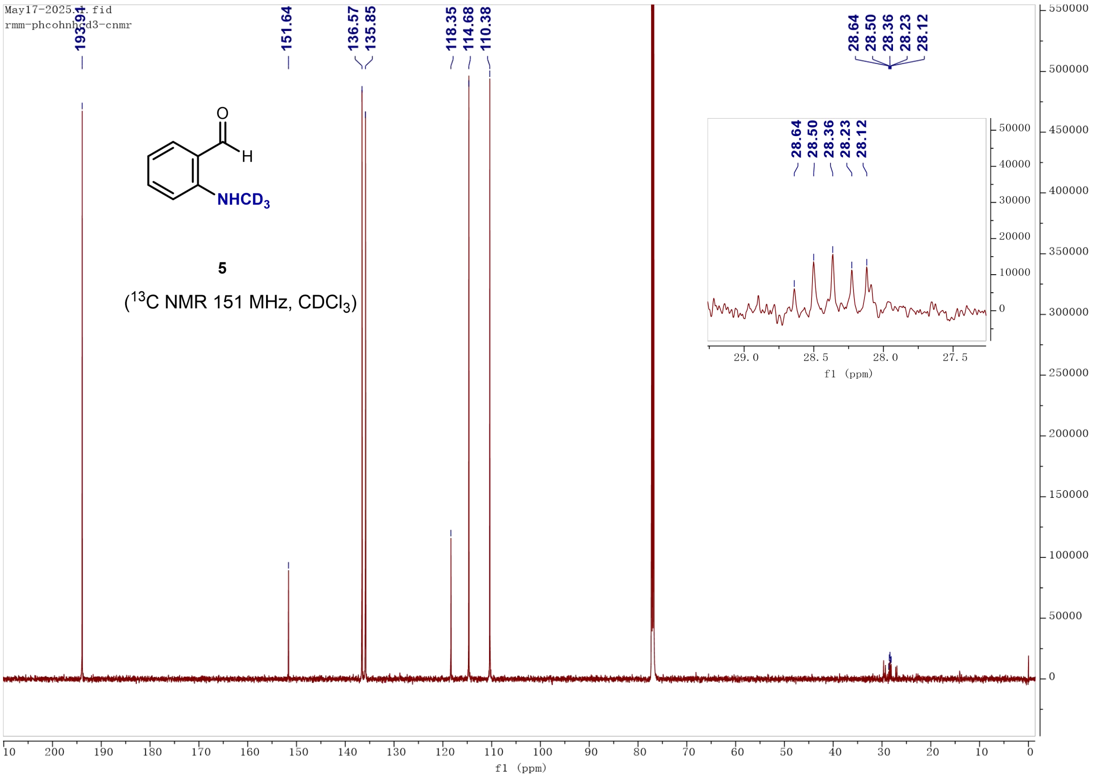
May17-2025.4.fid
rmm-phcohnhcd3-cnmr
193.91
151.64
136.57
135.85
118.35
114.68
110.38
28.64
28.50
28.36
28.23
28.12
NHCD3
5
(13C NMR 151 MHz, CDCl3)
f1 (ppm)

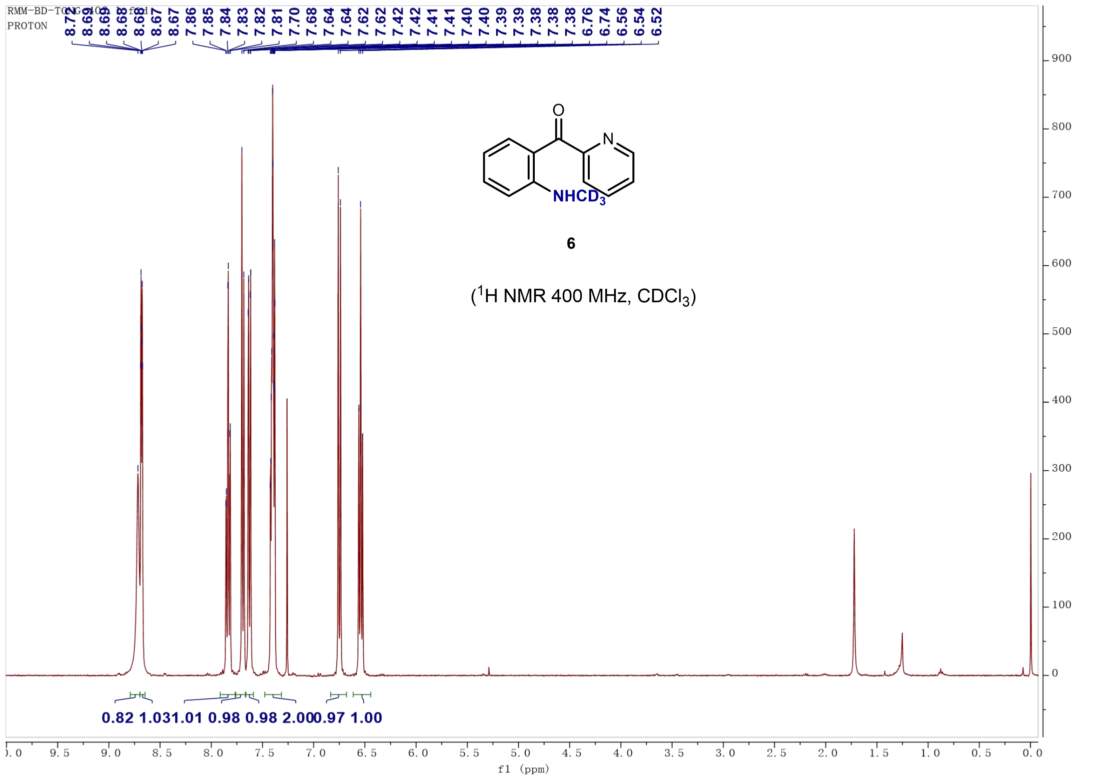
NHCD3
6
(1H NMR 400 MHz, CDCl3)
0.82 1.03 1.01 0.98 0.98 2.00 0.97 1.00
f1 (ppm)

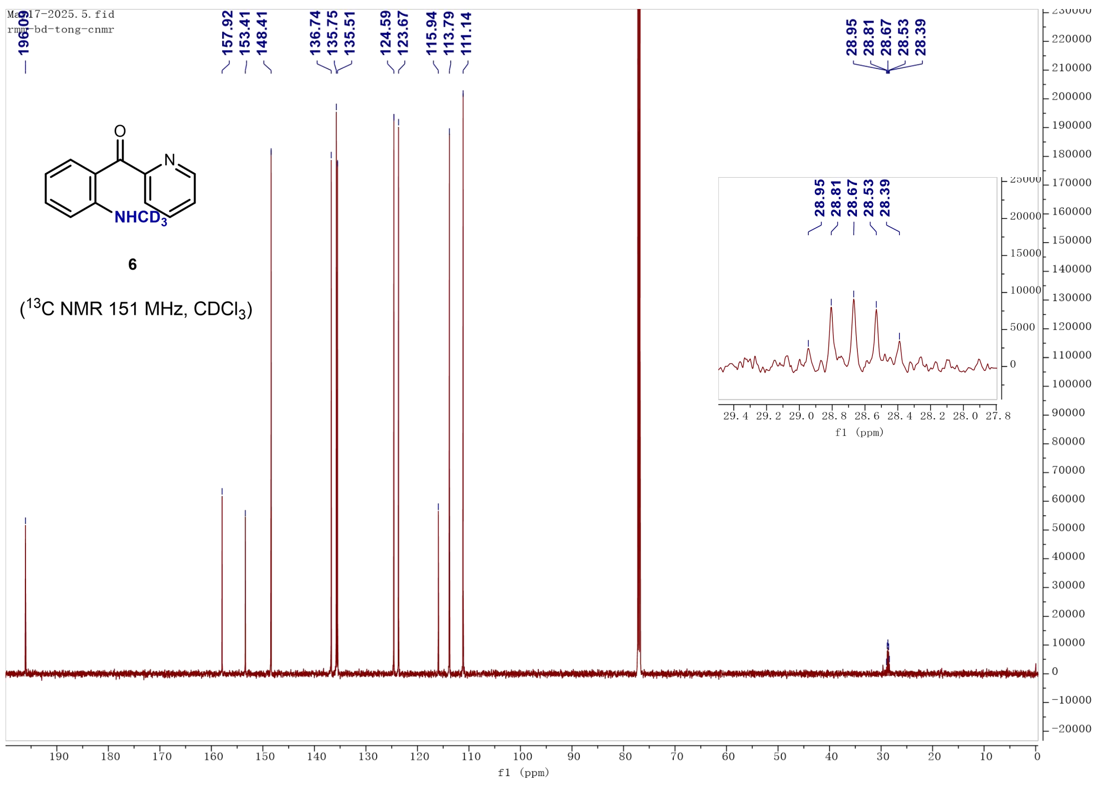
196.09
157.92
153.41
148.41
136.74
135.75
135.51
124.59
123.67
115.94
113.79
111.14
28.95
28.81
28.67
28.53
28.39
NHCD3
6
(13C NMR 151 MHz, CDCl3)
f1 (ppm)

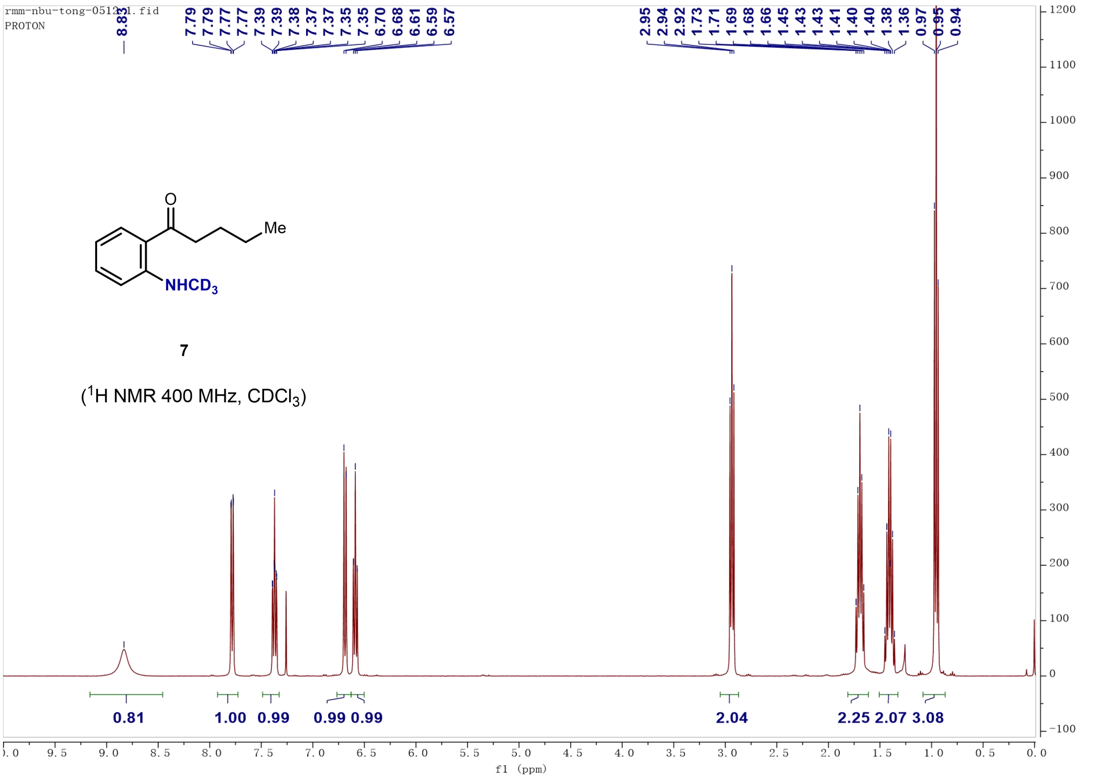
PROTON
8.83
7.79 7.79 7.77 7.77 7.39 7.39 7.38 7.37 7.37 7.35 7.35 6.70 6.68 6.61 6.59 6.57
2.95 2.94 2.92 1.73 1.71 1.69 1.68 1.66 1.45 1.43 1.43 1.41 1.40 1.40 1.38 1.36 0.97 0.95 0.94
O
Me
NHCD3
7
(1H NMR 400 MHz, CDCl3)
0.81 1.00 0.99 0.99 0.99 2.04 2.25 2.07 3.08
f1 (ppm)

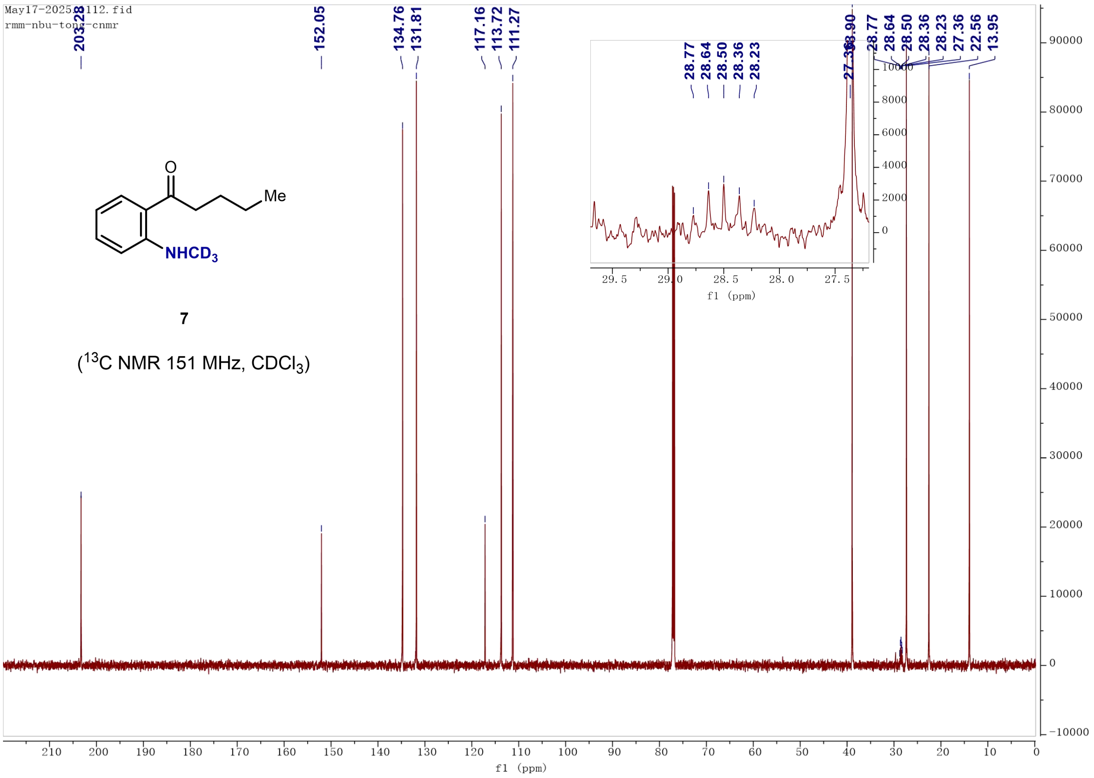
rmm-nbu-tong-cnmr
152.05
134.76 131.81
117.16 113.72 111.27
28.77 28.64 28.50 28.36 28.23 27.36 22.56 13.95
O
Me
NHCD3
7
(13C NMR 151 MHz, CDCl3)
f1 (ppm)

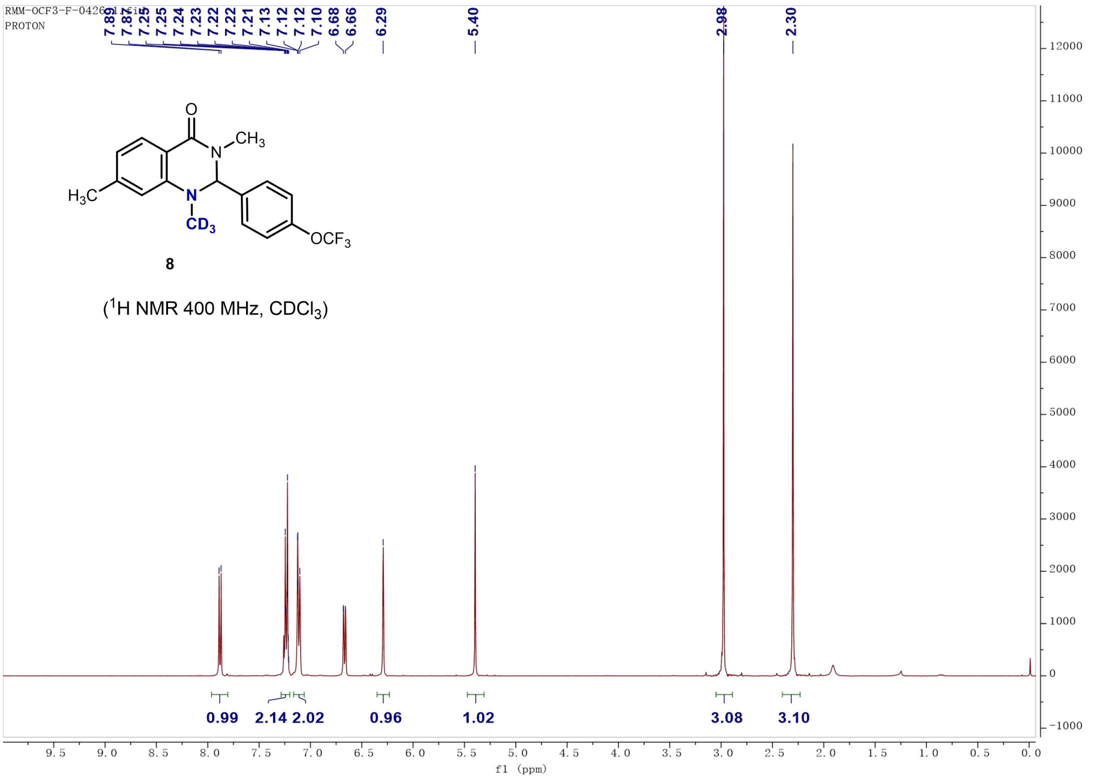

RMM-OCF3-F-0426
PROTON
7.89 7.87 7.25 7.25 7.24 7.23 7.22 7.22 7.21 7.13 7.12 7.12 7.10 6.68 6.66 6.29 5.40 2.98 2.30
8
(1H NMR 400 MHz, CDCl3)
0.99 2.14 2.02 0.96 1.02 3.08 3.10
f1 (ppm)

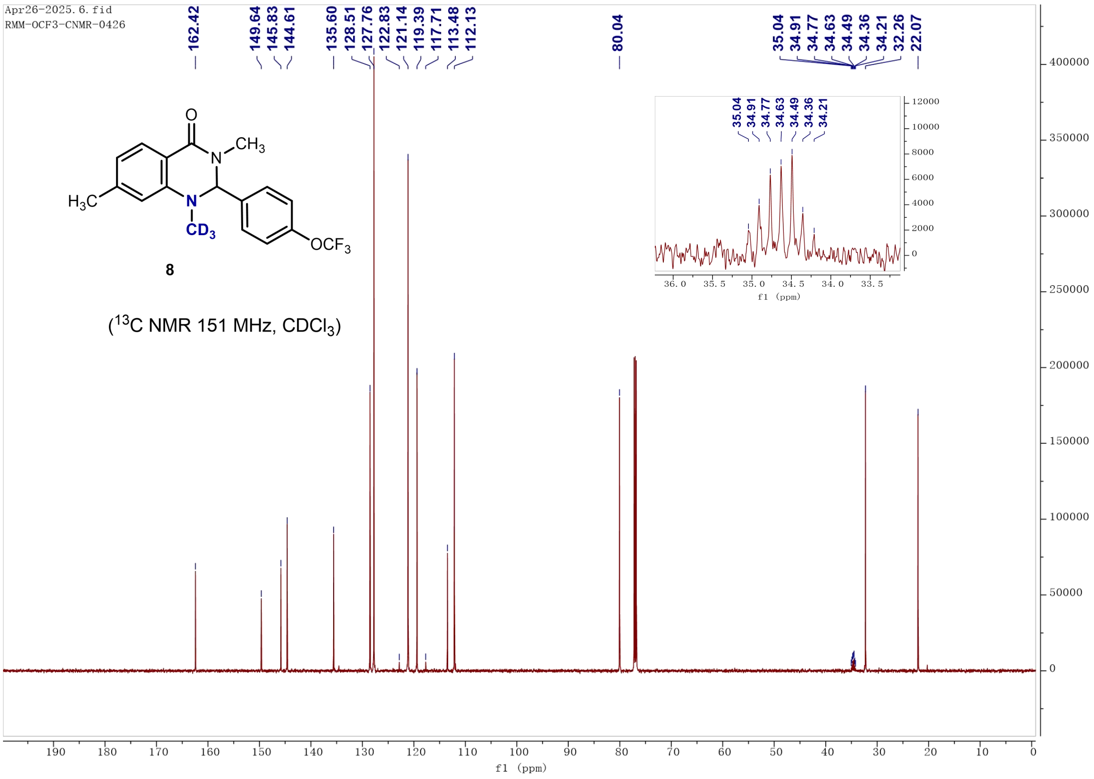

Apr26-2025.6.fid
RMM-OCF3-CNMR-0426
162.42 149.64 145.83 144.61 135.60 128.51 127.76 122.83 121.14 119.39 117.71 113.48 112.13 80.04 35.04 34.91 34.77 34.63 34.49 34.36 34.21 32.26 22.07
8
(13C NMR 151 MHz, CDCl3)
f1 (ppm)

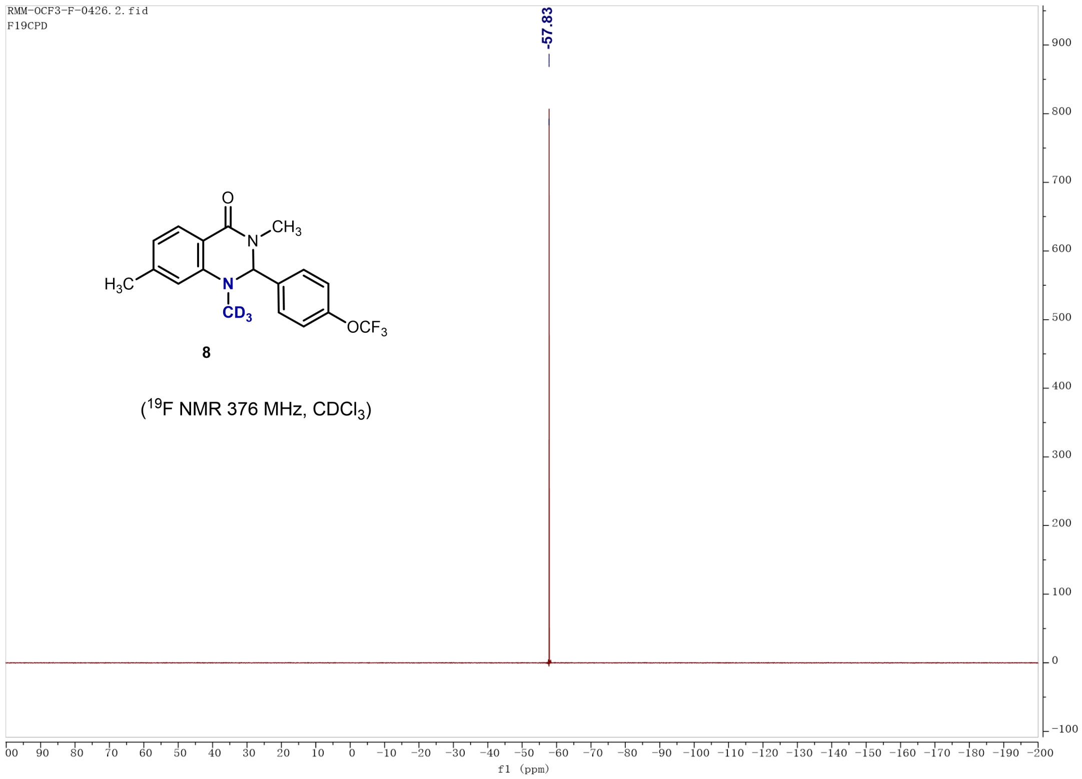
RMM-OCF3-F-0426.2.fid
F19CPD
-57.83
8
(^{19}F NMR 376 MHz, $CDCl_3$)
f1 (ppm)

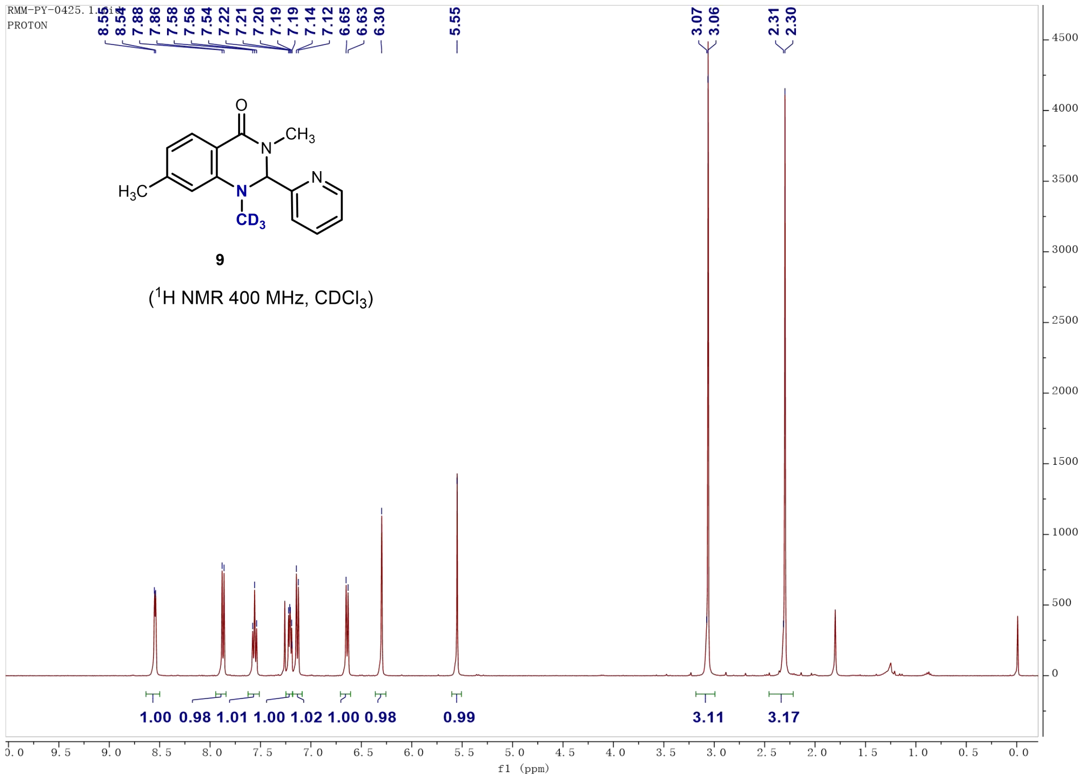
RMM-PY-0425.1.fid
PROTON
8.55 8.54 7.88 7.86 7.58 7.56 7.54 7.22 7.21 7.20 7.19 7.14 7.12 6.65 6.63 6.30
5.55
3.07 3.06
2.31 2.30
9
(^{1}H NMR 400 MHz, $CDCl_3$)
1.00 0.98 1.01 1.00 1.02 1.00 0.98 0.99 3.11 3.17
f1 (ppm)

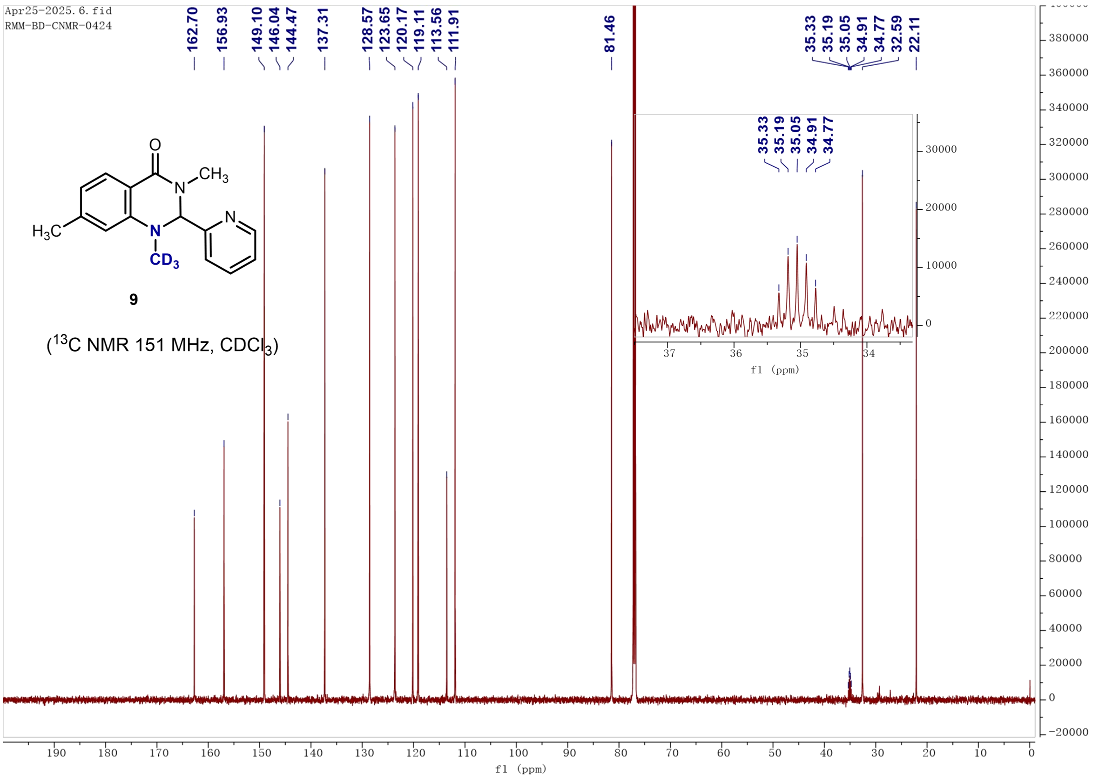
Apr25-2025.6.fid
RMM-BD-CNMR-0424
162.70 156.93 149.10 146.04 144.47 137.31 128.57 123.65 120.17 119.11 113.56 111.91 81.46 35.33 35.19 35.05 34.91 34.77 32.59 22.11
9
(^{13}C NMR 151 MHz, $CDCl_3$)
f1 (ppm)

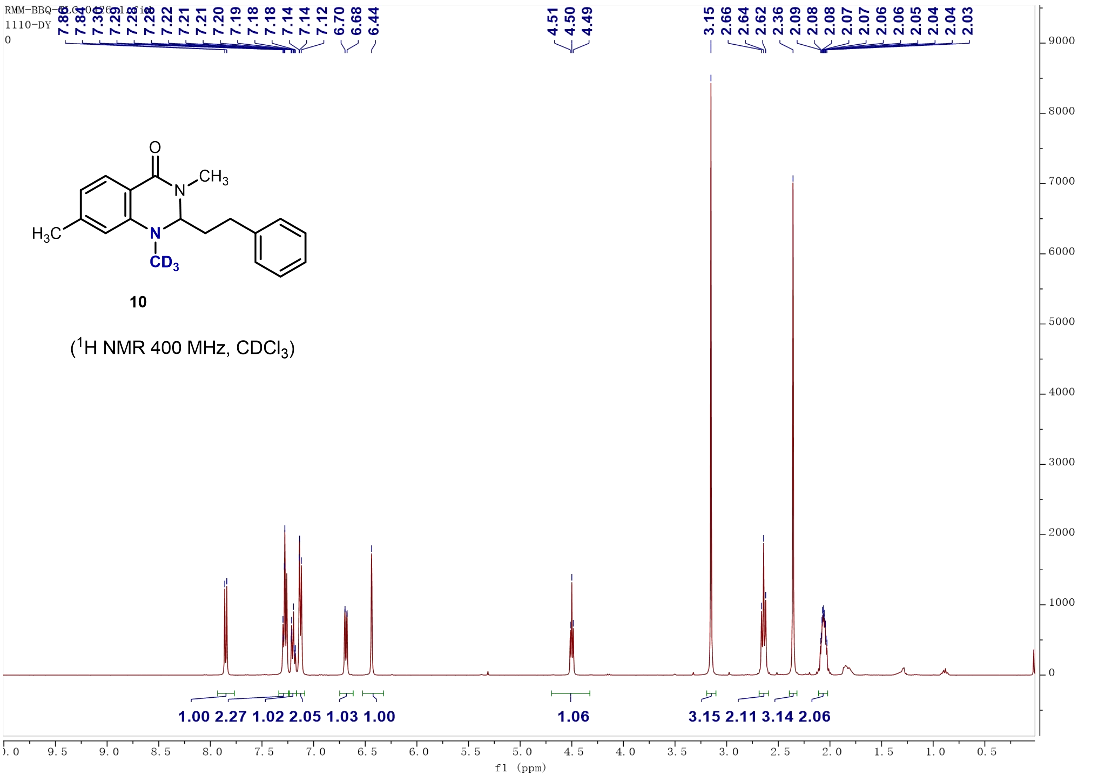
7.86 7.84 7.30 7.29 7.28 7.28 7.22 7.21 7.20 7.19 7.18 7.18 7.14 7.14 7.12 6.70 6.68 6.44 4.51 4.50 4.49 3.15 2.66 2.64 2.62 2.36 2.09 2.08 2.08 2.07 2.06 2.05 2.04 2.03
10
(^{1}H NMR 400 MHz, $CDCl_3$)
1.00 2.27 1.02 2.05 1.03 1.00 1.06 3.15 2.11 3.14 2.06
f1 (ppm)

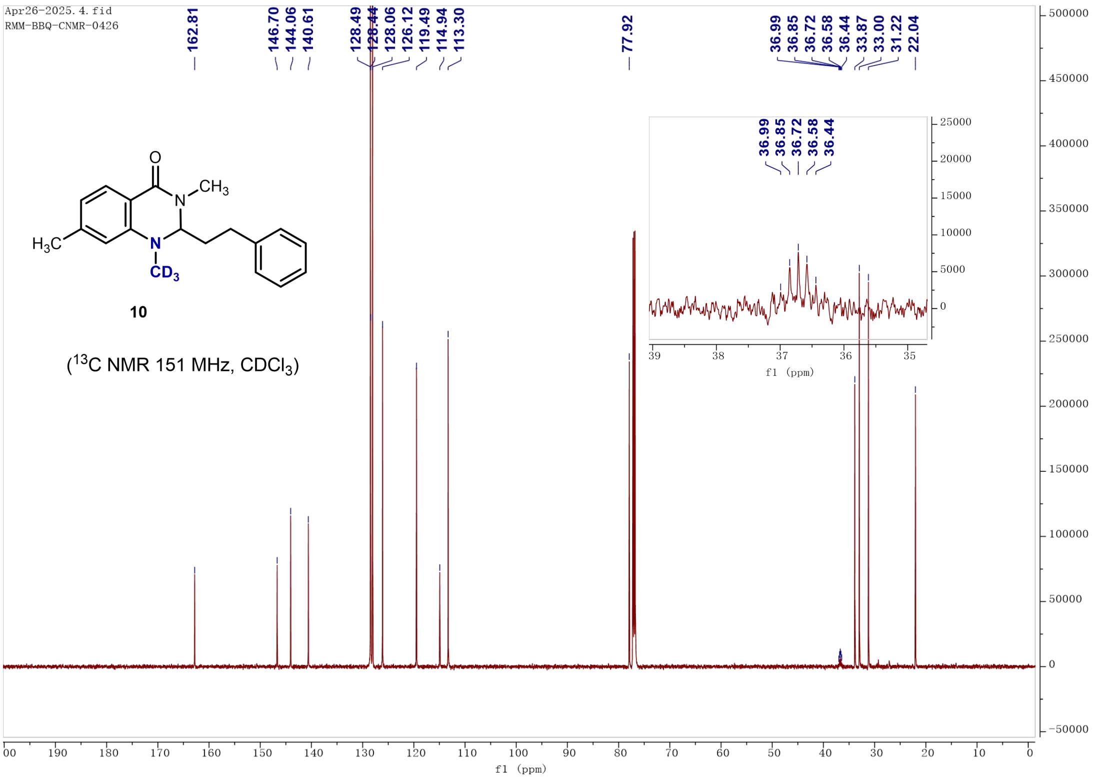
Apr26-2025.4.fid
RMM-BBQ-CNMR-0426
162.81
146.70
144.06
140.61
128.49
128.44
128.06
126.12
119.49
114.94
113.30
77.92
36.99
36.85
36.72
36.58
36.44
33.87
33.00
31.22
22.04
10
(13C NMR 151 MHz, CDCl3)
f1 (ppm)

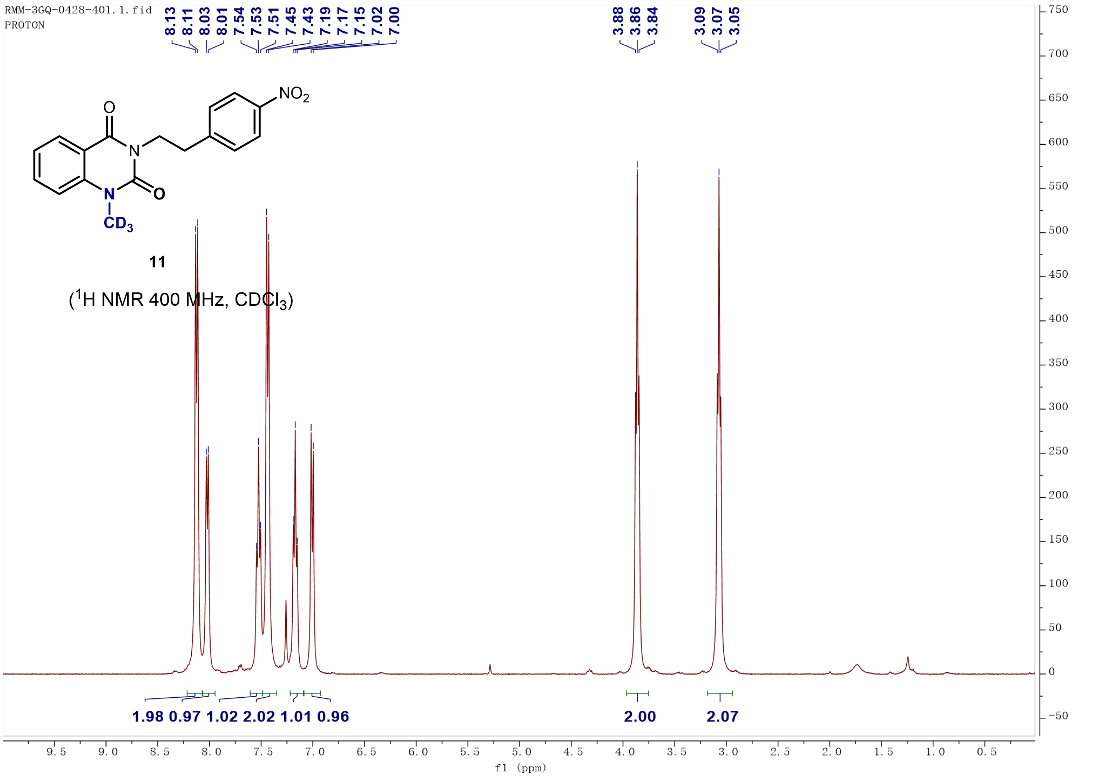
RMM-3GQ-0428-401.1.fid
PROTON
8.13
8.11
8.03
8.01
7.54
7.53
7.51
7.45
7.43
7.19
7.17
7.15
7.02
7.00
3.88
3.86
3.84
3.09
3.07
3.05
11
(1H NMR 400 MHz, CDCl3)
1.98 0.97 1.02 2.02 1.01 0.96
2.00
2.07
f1 (ppm)

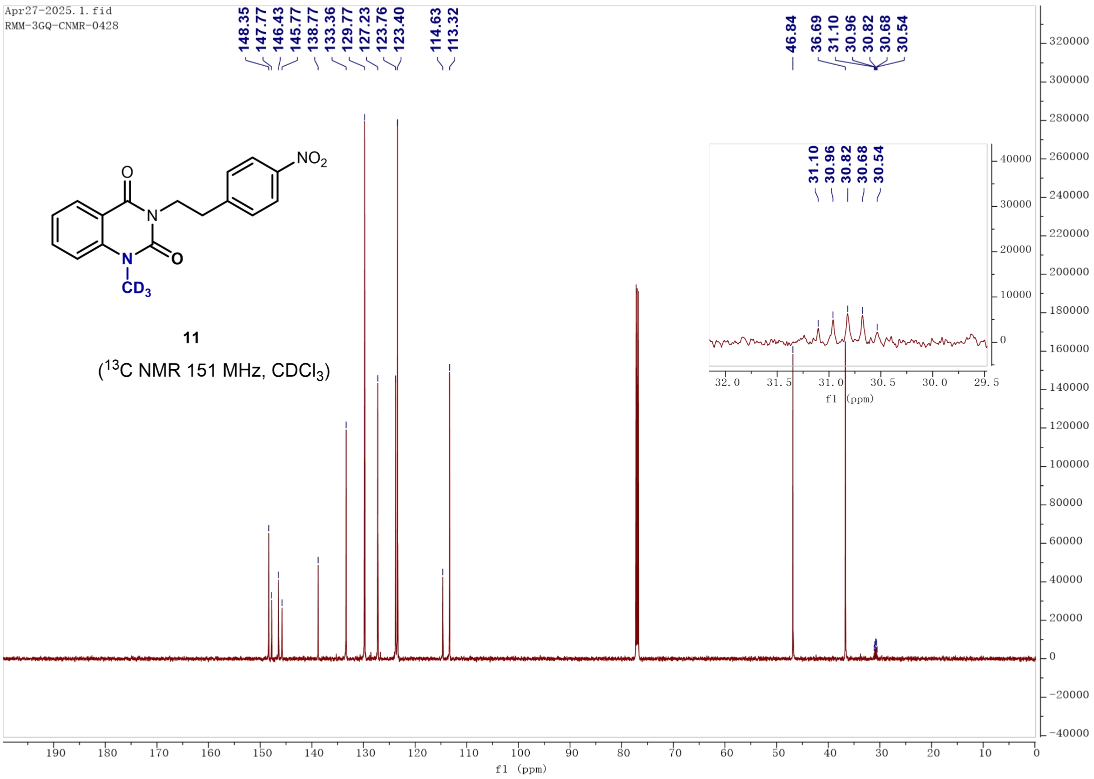

Apr27-2025.1.fid
RMM-3GQ-CNMR-0428
148.35
147.77
146.43
145.77
138.77
133.36
129.77
127.23
123.76
123.40
114.63
113.32
46.84
36.69
31.10
30.96
30.82
30.68
30.54
11
(^{13}C NMR 151 MHz, $CDCl_3$)
f1 (ppm)

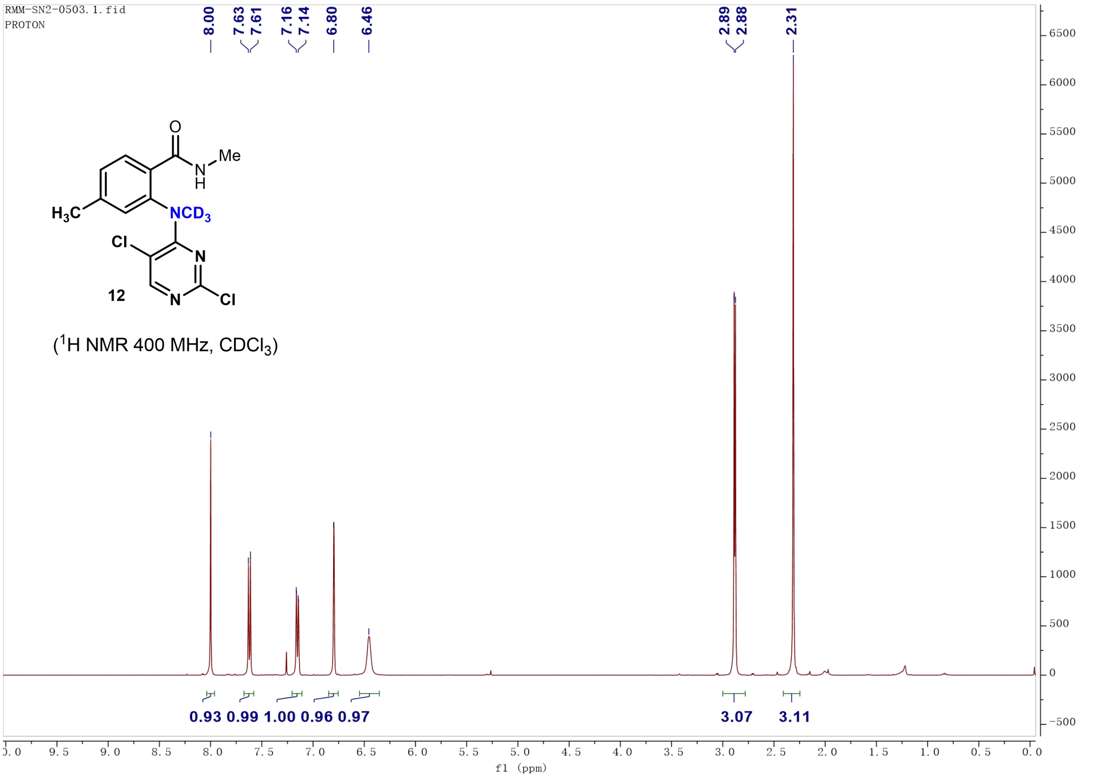

RMM-SN2-0503.1.fid
PROTON
8.00
7.63
7.61
7.16
7.14
6.80
6.46
2.89
2.88
2.31
12
(^{1}H NMR 400 MHz, $CDCl_3$)
0.93 0.99 1.00 0.96 0.97
3.07
3.11
f1 (ppm)

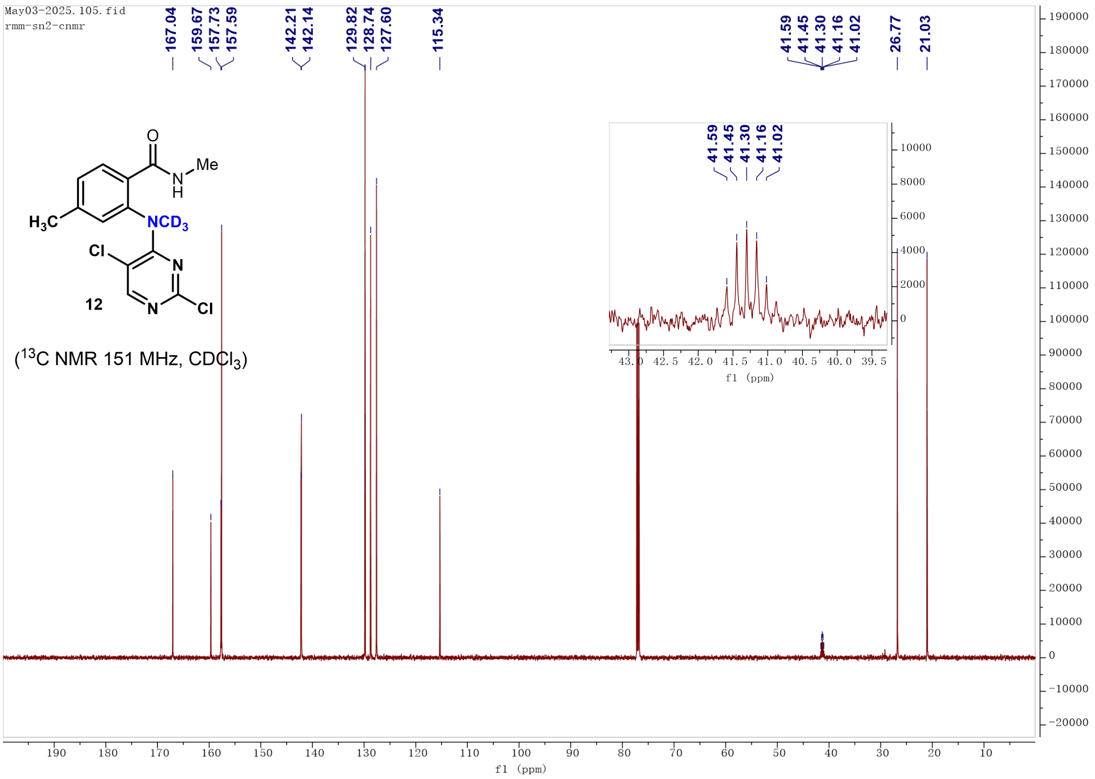
May03-2025.105.fid
rmm-sn2-cnmr
167.04
159.67
157.73
157.59
142.21
142.14
129.82
128.74
127.60
115.34
41.59
41.45
41.30
41.16
41.02
26.77
21.03
O
N
Me
H
H3C
NCD3
Cl
N
12
N
Cl
(13C NMR 151 MHz, CDCl3)
f1 (ppm)

12. References

1. E. Falk, S. Makai, T. Delcaillau, L. Gürtler, B. Morandi. *Angew. Chem. Int. Ed.* **2020**, *59*, 21064–21071.

2. E. Falk, V. C. M. Gasser, B. Morandi. *Org. Lett.* **2021**, *23*, 1422−1426.

3. P. G. K. Clark, M. Leina, R. A. Keyzers. *Org. Biomol. Chem.* **2012**, *10*, 1725.

4. M. Z. Liu, L. Xu, Y. Wei. *Chin. Chem. Lett.* **2022**, *33*, 1559–1562.

5. G. Pelletier, W. S. Bechara, A. B. Charette. *J. Am. Chem. Soc.* **2010**, *132*, 12817–12819.

6. J. Qi, C. Wang, G. Wang, P. O'Neill, S. R. Dubbaka, H. T. Ang, X. B. Chen, J. Wu. *Angew. Chem. Int. Ed.* **2025**, *64*, e202413723.

7. P. V. Ramachandran, H. J. Hamann, S. Choudhary. *Org. Lett.* **2020**, *22*, 8593−8597.

8. A. T. Nchinda, K. Chibale, P. Redelinghuysa, E. D. Sturrock. *Bioorg. Med. Chem. Lett.* **2006**, *16*, 4612–4615.

9. S. N. Rao, N. N. K. Reddy, S. Samanta, S. Adimurthy. *J. Org. Chem.* **2017**, *82*, 13632−13642.

10. X. Sun, W. Zhao, B. J. Li, *Chem. Commun.* **2020**, *56*, 1298−1301.

11. T. Yamaguchi, H. Ushirogochi, M. Hirai, Y. Imanishi, *Indazolepropionic acid amide compound.* U.S. Patent 20120016117, **2012**.

12. J. P. Jadav, J. K. Vankar, A. Gupta, G. N. Gururaja. *J. Org. Chem.* **2023**, *88*, 15551−15561.

13. P. C. Lian, K. F. Wang, H. Liu, R. Y. Li, M. G. Li, X. G. Bao, X. B. Wang, *Org. Lett.* **2023**, *25*, 7984−7989.

14. W. S. Ji , X. L. Li, Q. S. Huang, J. B. Xu, X. H. Li, F. Gao, *Synlett* **2025**; *36*, 1723-1728.

15. J. Templ, E. Gjata, F. Getzner, M. Schnürch. *Org. Lett.* **2022**, *24*, 7315−7319.

16. Y. M. Tian, X. Pu, A. H. Sánchez, W. Silva, R. M. Gschwind, B. Königa. *Adv. Synth. Catal.* **2025**, *367*, e202400547.

17. J. H. Schrittwieser, V. Resch, J. H. Sattler, W. D. Lienhart, K. Durchschein, A. Winkler, K. Gruber, P. Macheroux, W. Kroutil. *Angew. Chem. Int. Ed.* **2011**, *50*, 1068−1071.

18. Y. Wang, W. X. Zhang, Z. T. Wang, Z. F. Xi. *Angew. Chem. Int. Ed.* **2011**, *50*, 8122–8126.

19. J. I. Lee. Bull. Korean Chem. Soc. 2021, 42, 556–558.

20. S. Nahm, S. M. Weinreb. *Tetrahedron Lett.* **1981**, *22*, 3815–3818.

21. X. Fan, L.Z. Yu, Y. Wei, M. Shi. *Org. Lett*. **2017**, *19*, 4476–4479.

22. E. Sawatzky, A. Drakopoulos, M. Rölz, C. Sotriffer, B. Engels, M. Decker. Beilstein *J. Org. Chem.* **2016**, *12*, 2280–2292.

23. T. Inaba, T. Kaya, H. Iwamura. *Compounds and pharmaceutical use thereof.* U.S. Patent 2000006017, **2000**.

24. L. R. Weinberg, M. S. Albom, T. S. Angeles, J. Husten, J. G. Lisko, R. J. McHugh, K. L. Milkiewicz, S. Murthy, G. R. Ott, J. P. Theroff, R. Tripathy, T. L. Underiner, C. A. Zificsak, B. D. Dorsey. *Bioorg.Med.Chem.Lett.* **2011**, *21*, 164–167.
